# Tumor-targeted interferon-α gene therapy for glioblastoma: a phase 1 trial

**DOI:** 10.1038/s41591-026-04419-1

**Published:** 2026-06-01

**Authors:** Bernhard Gentner, Marica Eoli, Francesca Farina, Matteo Barcella, Alessia Capotondo, Stefania Mazzoleni, Valentina Brambilla, Antonio Francavilla, Mariagrazia Garramone, Matteo G. Carrabba, Valeria Ferla, Alessandro Bruno, Giorgia Alvisi, Nadia Coltella, Eugenio Montini, Leila Hadadi, Charlotte Capt, Valeria Cuccarini, Maria Grazia Bruzzone, Francesco Di Meco, Federico G. Legnani, Paolo Ferroli, Francesco Acerbi, Roberto Pallini, Quintino Giorgio D’Alessandris, Alessandro Olivi, Filippo Gagliardi, Silvia Snider, Pietro Mortini, Monica Patanè, Amleto Fiocchi, Valeria Berno, Pietro Luigi Poliani, Gaetano Finocchiaro, Carlo Russo, Fabio Ciceri, Luigi Naldini

**Affiliations:** 1 San Raffaele Telethon Institute for Gene Therapy (SR-TIGET), IRCCS San Raffaele Scientific Institute, Milan, Italy; 2 Hematology and Bone Marrow Transplantation Unit, IRCCS San Raffaele Hospital, Milan, Italy; 3 Ludwig Institute for Cancer Research and Department of Oncology, University of Lausanne (UNIL) and Lausanne University Hospital (CHUV), Lausanne, Switzerland; 4 Experimental Neuro-Oncology Unit, Fondazione IRCCS Istituto Neurologico Carlo Besta, Milan, Italy; 5 Genenta Science, Milan, Italy; 6 Neuroradiology Unit, Fondazione IRCCS Istituto Neurologico Carlo Besta, Milan, Italy; 7 Department of Neurosurgery, Fondazione IRCCS Istituto Neurologico Carlo Besta, Milan, Italy; 8 University of Milan, Department of Oncology and Hemato-oncology, Milan, Italy; 9 Department of Translational Research and New Technology in Medicine and Surgery, University of Pisa, Pisa, Italy; 10 Neurosurgery Unit, Azienda Ospedaliero Universitaria Pisana, Pisa, Italy; 11 Department of Neurosurgery, Fondazione Policlinico Universitario Agostino Gemelli IRCCS, Rome, Italy; 12 Department of Neurosurgery and Gamma Knife Radiosurgery, IRCCS San Raffaele Hospital, Milan, Italy; 13 Vita-Salute San Raffaele University, Milan, Italy; 14 Unit of Neuropathology, Fondazione IRCCS Istituto Neurologico Carlo Besta, Milan, Italy; 15 Animal Histopathology Core Facility, IRCCS San Raffaele Hospital, Milan, Italy; 16 Advanced Light and Electron Microscopy Bioimaging Center ALEMBIC, IRCCS San Raffaele Scientific Institute, Milan, Italy; 17 Pathology Unit, Department of Molecular and Translational Medicine, University of Brescia Medical School, Brescia, Italy; 18 Neurology Unit, IRCCS San Raffaele Scientific Institute, Milan, Italy

**Keywords:** Phase I trials, Cancer immunotherapy, Translational research, CNS cancer, Cancer immunotherapy

## Abstract

Glioblastoma (GBM) is an immunologically cold brain tumor with poor outcome, characterized by a myeloid-driven immunosuppressive microenvironment. Here we report an interim analysis of a first-in-human phase 1/2a dose-escalation study evaluating Temferon—a genetically engineered autologous stem cell transplant designed to deliver interferon-α2 by means of myeloid progeny recruited to the GBM TME and locally activate antitumor immunity. Twenty-four newly diagnosed patients with GBM and unmethylated *MGMT* promoter were treated across eight cohorts following surgical resection and radiotherapy, testing Temferon doses ranging from 0.5 × 10^6^ to 4.0 × 10^6^ CD34^+^ cells kg^−1^ and different conditioning regimens (BCNU or busulfan, with or without thiotepa). The primary endpoint was safety and tolerability within 90 days after infusion. Secondary endpoints included long-term safety, dose and conditioning regimen selection, Temferon engraftment, clinical response, quality of life and survival. No dose-limiting toxicities were observed up to the highest dose level of temferon tested. Busulfan conditioning was selected for further development. Adverse events included laboratory abnormalities, cytopenias and infections consistent with autologous stem cell transplant. Median overall survival and progression-free survival were 16.7 months and 8.1 months from diagnosis, respectively, with most patients maintaining good performance status and quality of life. Genetically engineered cells were detected long term in the bone marrow and the blood, where minimal amounts of interferon-α were measured. Temferon is a safe and tolerable immunotherapeutic strategy in patients with newly diagnosed GBM. ClinicalTrials.gov: NCT03866109.

## Abstract (web summary)

In an interim analysis of a phase 1/2 trial in 24 patients with newly diagnosed glioblastoma, a genetically engineered autologous stem cell transplant designed to deliver interferon-α to the tumor microenvironment was well tolerated, with stable engraftment and evidence for immune reprogramming.

## Main

Immunotherapy has become a principal pillar of cancer treatment, yet its efficacy is frequently limited by the TME, which is often dominated by myeloid cells. Tumor-associated macrophages (TAMs) promote tumor growth, angiogenesis, invasion, metastasis and therapy resistance, correlating with poor clinical outcome^1^. Owing to their marked phenotypic plasticity, TAMs can exert pro- and antitumoral functions and are increasingly recognized as critical components and therapeutic targets in the cancer ecosystem^2^. Although the therapeutic potential of inhibiting protumoral functions of TAMs has been broadly recognized, clinical breakthroughs comparable to T cell-directed therapies have not yet been achieved^3–5^.

Several factors may underlie these limitations. Broad depletion of TAMs may be undesirable, as macrophages can support antitumor immunity if reprogrammed away from immunosuppressive states. In addition, functional redundancy among suppressive myeloid populations and the dynamic, adaptive nature of the TME may necessitate precisely timed and context-dependent interventions. Although advances in monocyte/macrophage (MoMac) engineering have been made^6^, their application as cell therapies remains challenging due to their limited lifespan, restricted proliferation and poorly understood determinants of phenotypic stability after adoptive transfer.

Under nonhomeostatic conditions, including radiotherapy and chemotherapy, tissue macrophages can be replenished by hematopoietic stem/progenitor cell (HSPC)-derived monocytes^7,8^, which often become a principal source of TAMs during tumor progression^9–11^. This creates an opportunity to engineer HSPCs to steadily generate MoMac progeny endowed with built-in antitumor functions. Ex vivo lentiviral HSPC gene therapy has enabled stable, long-term engraftment in hundreds of patients with monogenic disorders^12^. Notably, durable restoration of enzyme activity in the central nervous system (CNS) of patients with lysosomal storage diseases demonstrates substantial microglia replacement after busulfan conditioning and infusion of genetically modified HSPCs, with effective biodistribution across the blood–brain barrier^7,13^.

GBM, characterized by highly invasive and plastic tumor cells and a profoundly immunosuppressive TME, remains one of the deadliest malignancies, with a median survival of 14–16 months and little therapeutic progress since the introduction of the Stupp regimen nearly two decades ago^14^. As a paradigmatic myeloid-dominant, immunologically ‘cold’ tumor refractory to immune checkpoint blockade^15,16^, GBM represents a compelling setting to evaluate TAM-directed cellular therapies capable of penetrating the CNS and reprogramming the TME towards immune activation.

We previously identified TIE2-expressing MoMacs (TEMs) as a proangiogenic TAM subset associated with tumor progression, relapse and metastasis in mice and humans^17–19^. To exploit TEMs as cellular vehicles to deliver anticancer cytokines locally within tumors, we designed TIA126—a lentiviral vector in which the *TIE2* enhancer/promoter drives interferon (IFN)-α2 (IFNα) expression, combined with microRNA-126 target sequences in the 3′ untranslated region to suppress transgene expression in HSPCs and prevent constitutive IFNα exposure in the bone marrow (BM)^20,21^. In murine models, HSPCs transduced with TIA126 showed stable engraftment and broad antitumor activity mediated by immune cell recruitment, angiogenesis inhibition and activation of innate and adaptive immunity, without systemic toxicity or impairment of physiological processes, such as wound healing or response to viral infections^21–24^. In an orthotopic, immunocompetent GBM model, TME-targeted IFN signaling induced myeloid reprogramming, reduced protumoral TAM subsets, enhanced antigen presentation and promoted the antitumoral response of endogenous and adoptively transferred T cells^25,26^.

Here we report a prespecified interim analysis from a first-in-human, phase 1/2a dose-escalation study of autologous TIA126-transduced HSPC (Temferon) administered after tumor resection, conformal focal radiotherapy (CFRT) and submyeloablative conditioning in patients with GBM harboring an unmethylated O^6^-methylguanine-DNA methyltransferase (*MGMT*) promoter. The study demonstrates feasibility and safety, durable BM engraftment, minimal systemic IFNα exposure, recruitment of Temferon progeny into the GBM TME and local IFNα-driven immune reprogramming of myeloid and T cell compartments associated with tumor control.

## Results

### Clinical trial design and patient disposition

**Fig. 1:**
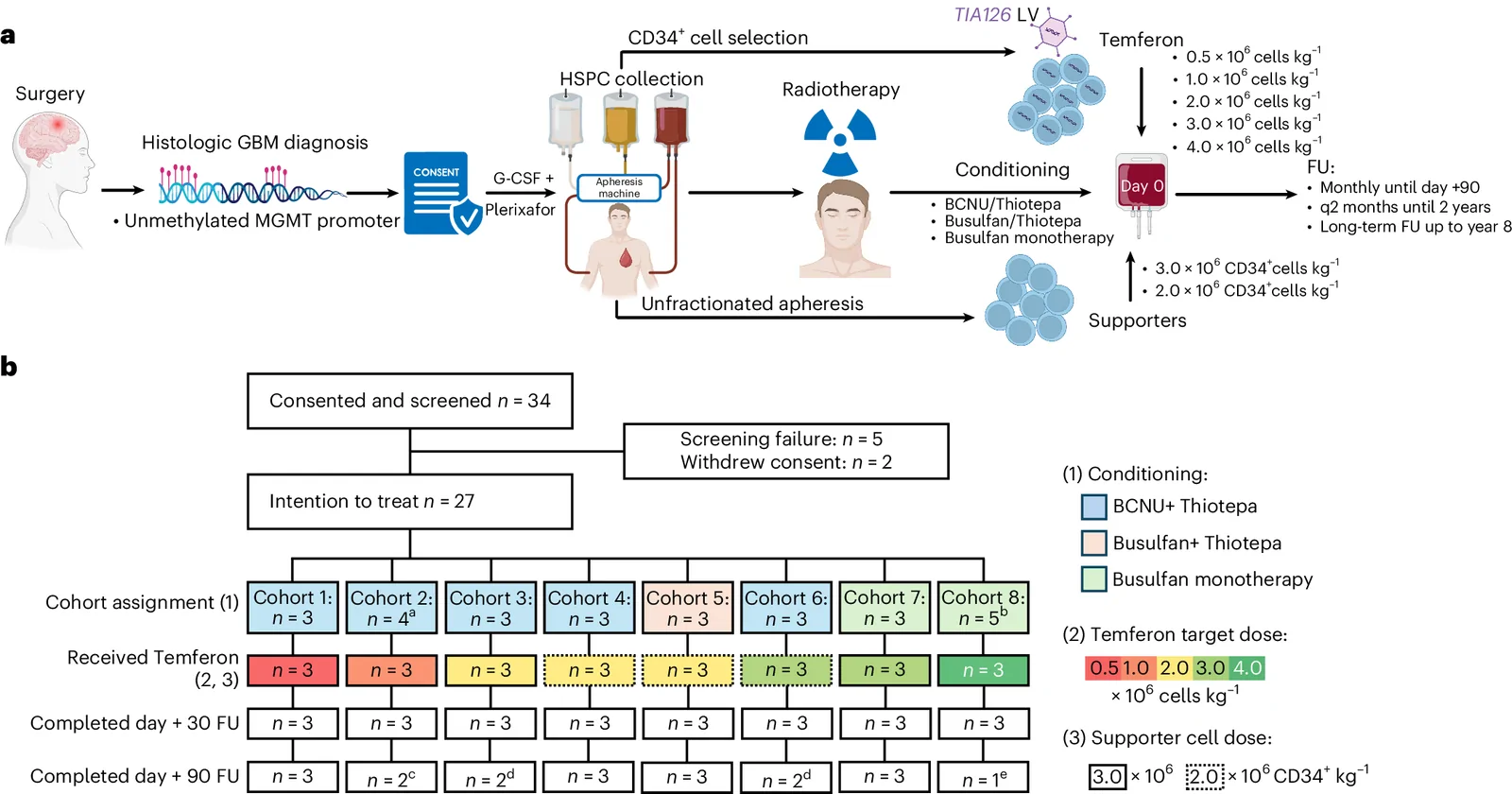
Patient disposition and study design. **a**, Schematic of the TEM-GBM_001 clinical trial enrolling patients with newly diagnosed, isocitrate dehydrogenase-wild-type GBM. **b**, CONSORT-style flow diagram showing the number of evaluable patients (*n*) at each trial stage up to day +90 FU post Temferon infusion. Patients were assigned to eight cohorts according to conditioning regimen (1), Temferon target dose (2) and Supporter CD34^+^ cell target dose (3). Postassignment attrition was due to the following: ^a^ineligibility due to poor condition associated with disease progression (*n* = 1); ^b^death before infusion or poor condition associated with disease progression (*n* = 2); ^c^loss to FU during the COVID-19 pandemic (*n* = 1);^d^early postinfusion death (*n* = 2); ^e^censoring at data cutoff (*n* = 2).

A phase 1/2a dose-escalation study (EudraCT 2018-001404-11) was initiated in March 2019 to evaluate the safety and efficacy of Temferon in patients with newly diagnosed GBM with unmethylated *MGMT* promoter (*MGMT*^unmeth^). The starting dose was set to reflect the minimum graft chimerism (>10%) associated with antitumor activity in mice^25^, with escalation planned up to 50% chimerism based on preclinical toxicity and biodistribution studies (Supplementary Figs. 1 and 2). The impact of three variables on in vivo chimerism—Temferon dose, CD34^+^ supporter cell dose from unfractionated apheresis and conditioning regimen—was investigated across eight cohorts of three patients each. Eligible patients had histologically confirmed GBM following surgical resection, no contraindication to adjuvant radiotherapy and a life expectancy ≥6 months. After informed consent, patients underwent HSPC mobilization and collection by one to two leukapheresis sessions (Fig. 1a). At least 5 × 10^6^ CD34^+^ cells kg^−1^ were cryopreserved as unfractionated apheresis, whereas the remaining cells were used for Temferon manufacturing under transduction conditions targeting a vector copy number (VCN) of ~1. Meanwhile, patients received CFRT (60 Gy over 6 weeks) omitting Temozolomide (TMZ) considering its limited benefit in *MGMT*^unmeth^ GBM. Temferon (0.5–4 × 10^6^ cells kg^−1^) was administered intravenously after submyeloablative conditioning, and ≥3 h after infusion of unfractionated apheresis (≥2–3 × 10^6^ CD34^+^ kg^−1^), sufficient to support hematologic recovery. Between 12 April 2019 and 26 February 2024, 34 patients were screened and allocated to eight cohorts (Fig. 1b). Twenty-seven patients (79.4%) constituted the intention-to-treat population, and 24 ultimately received Temferon. In cohorts 1–4 and 6, conditioning consisted of BCNU (carmustin) and thiotepa with Temferon dose escalation from 0.5 to 3 × 10^6^ cells kg^−1^ and de-escalation of supporter cells from 3 × 10^6^ (cohorts 1–3) to 2 × 10^6^ CD34^+^ cells kg^−1^ (cohorts 4 and 6). Cohorts were enrolled sequentially following independent data monitoring committee (DMC) review after completion of the day +30 visit by the third patient and absence of DLT. This regimen was selected based on established use in cerebral lymphoma, CNS penetration and potential effects on favoring macrophage turnover within the GBM TME. Cohort 5 received same cell doses as cohort 4, but was conditioned with busulfan plus thiotepa, to compare transduced HSPC engraftment. In cohorts 7 and 8, busulfan monotherapy was used to reduce toxicity and immunosuppression, with further Temferon escalation to 4 × 10^6^ cells kg^−1^ and reinstatement of 3 × 10^6^ CD34^+^ cells kg^−1^ as supporter cell dose per DMC recommendation. The total infused CD34^+^ cell dose ranged from ~4 × 10^6^ to 8 × 10^6^ cells kg^−1^, with a median estimated proportion of transduced cells increasing from 8% (cohort 1) to 33% (cohort 8). Patient, tumor and product characteristics are summarized in Table 1 and Supplementary Tables 1–3.

**Table 1:** Summary of patient characteristics

Patient characteristics	Temferon dosed (*n* = 24)	Temferon intention-to-treat (*n* = 27)
**Age**		
Median, years (range)	59 (29–67)	59 (29–67)
≥50 years, *n* (%)	17 (71%)	20 (74%)
<50 years, *n* (%)	7 (29%)	7 (26%)
**Gender**		
Male, *n* (%)	15 (62.5%)	17 (63%)
Female, *n* (%)	9 (37.5%)	10 (37%)
**Karnofsky performance status**		
100, *n* (%)	7 (29.2%)	9 (33.3%)
90, *n* (%)	3 (12.5%)	3 (11.1%)
80, *n* (%)	12 (50%)	13 (48.1%)
70, *n* (%)	2 (8.3%)	2 (7.4%)
**Primary tumor resection**		
Partial, *n* (%)	8 (33.3%)	8 (29.6%)
Complete, *n* (%)	16 (66.6%)	19 (70.4%)

### Safety and tolerability

**Fig. 2:**
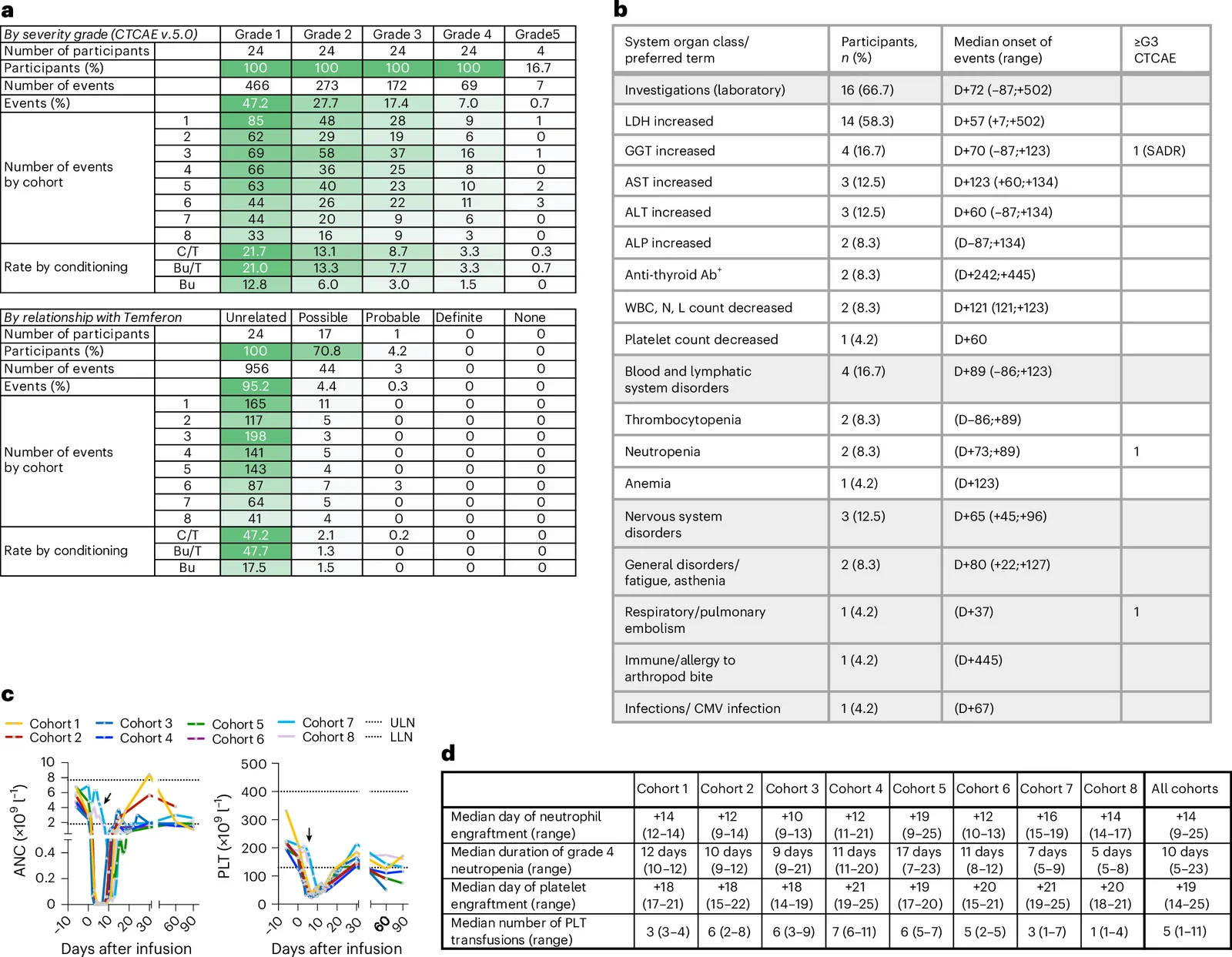
Safety endpoints. **a**, Summary of TEAEs stratified by severity (upper table) and relationship to Temferon (lower table). The rate by conditioning was calculated as the number of events divided by the number of patients per regimen: carmustine + thiotepa (C/T, *n* = 15), busulfan + thiotepa (Bu/T, *n* = 3) or busulfan alone (Bu, *n* = 6). Intensity of the green shade reflects AE frequency across a row (darker = more intense). **b**, AEs attributed to Temferon (ADRs) or to procedures related to HSPC collection or Temferon administration. Clinically significant organ toxicities were defined as CTCAE v.5.0 grade ≥3. Categories of nervous system disorders include the preferred terms ‘ageusia, dysgeusia, parosmia, dysaestesia, memory impairment and partial seizures.’ **c**, Hematologic recovery by cohort. ANC and PLT during the first 90 days after Temferon infusion (median values; *n* = 3 per cohort) measured by routine complete blood counts. Arrows indicate slower ANC and PLT decline in cohorts receiving busulfan-only conditioning. **d**, Neutrophil and platelet engraftment summarized by cohort, defined as the first of 3 consecutive days with ANC > 0.5 × 10^9^ l^−1^ and PLT > 20 × 10^9^ l^−1^ in the absence of transfusion for ≥7 consecutive days. ALP, alkaline phosphatase; ALT, alanine aminotransferase; AST, aspartate aminotransferase; D, day; GGT, gamma glutamyl transferase; L, lymphocyte; LDH, lactate dehydrogenase; LLN, lower limit of normal; N, neutrophil; SADR, severe ADR; ULN, upper limit of normal; WBC, white blood cell.

The primary endpoint was to assess the tolerability and safety of Temferon during the first 90 days after administration, with long-term safety (up to 2 years) reported as secondary endpoint. This interim analysis aimed to define the maximally tolerated dose after the third patient in cohort 8 completed the day +30 follow-up (FU). Dose-limiting toxicity (DLT) was defined as any grade 3–5 adverse event (AE) attributed to Temferon within 30 days of infusion. An overview of AEs by severity and attribution is shown in Fig. 2a and Supplementary Table 4. Temferon infusion was well tolerated, with no AEs attributed to the infusion itself. Complete listings of pretreatment AEs and treatment-emergent AEs (TEAEs) are provided in Supplementary Tables 5 and 6. All patients experienced TEAEs, most of which were mild or moderate (75%, CTCAE v.5 grade 1 or 2) and related primarily to conditioning chemotherapy consistent with expected toxicities of autologous stem cell transplant (ASCT). Events were distributed similarly across all cohorts and showed no association with Temferon dose. Patients receiving busulfan monotherapy experienced fewer TEAEs—particularly, fewer infections. Three patients, all treated with double-alkylator conditioning, died within 4 months from infectious complications, corresponding to a transplant-related mortality of 12.5%. Another patient died suddenly on day +402, probably due to disease progression. Apart from anti-thyroid antibodies detected in two asymptomatic patients >1 year after infusion, no autoimmune manifestations were observed. AEs related to HSPC collection, Temferon administration or Temferon exposure (adverse drug reactions (ADRs)) are summarized in Fig. 2b. Most events were mild and without relevant clinical impact, with transient alterations in blood chemistry being the most frequent findings. Three events met the protocol definition of significant organ toxicity. Following primary engraftment, patient G22 developed grade 3 neutropenia that resolved spontaneously. Patient G14 exhibited persistent liver enzyme elevations (>10× upper limit of normal), considered possibly related to Temferon. A liver biopsy showed mild acute toxic hepatitis, without important inflammatory infiltrate and absence of TEMs or CD34^+^ HSPC. The abnormalities were asymptomatic and resolved without intervention. Patient G34 developed pulmonary embolism secondary to deep vein thrombosis on day +37 in the context of several risk factors and was treated with therapeutic anticoagulation. No DLTs were observed at Temferon doses up to 4.0 × 10^6^ cells kg^−1^. Notably, no cytokine-related toxicities attributable to systemic IFNα exposure, or neurotoxicity syndromes typical of chimeric antigen receptor (CAR)-T cell therapies, were observed (Supplementary Table 6 and 7).

**Extended Data Fig. 1:**
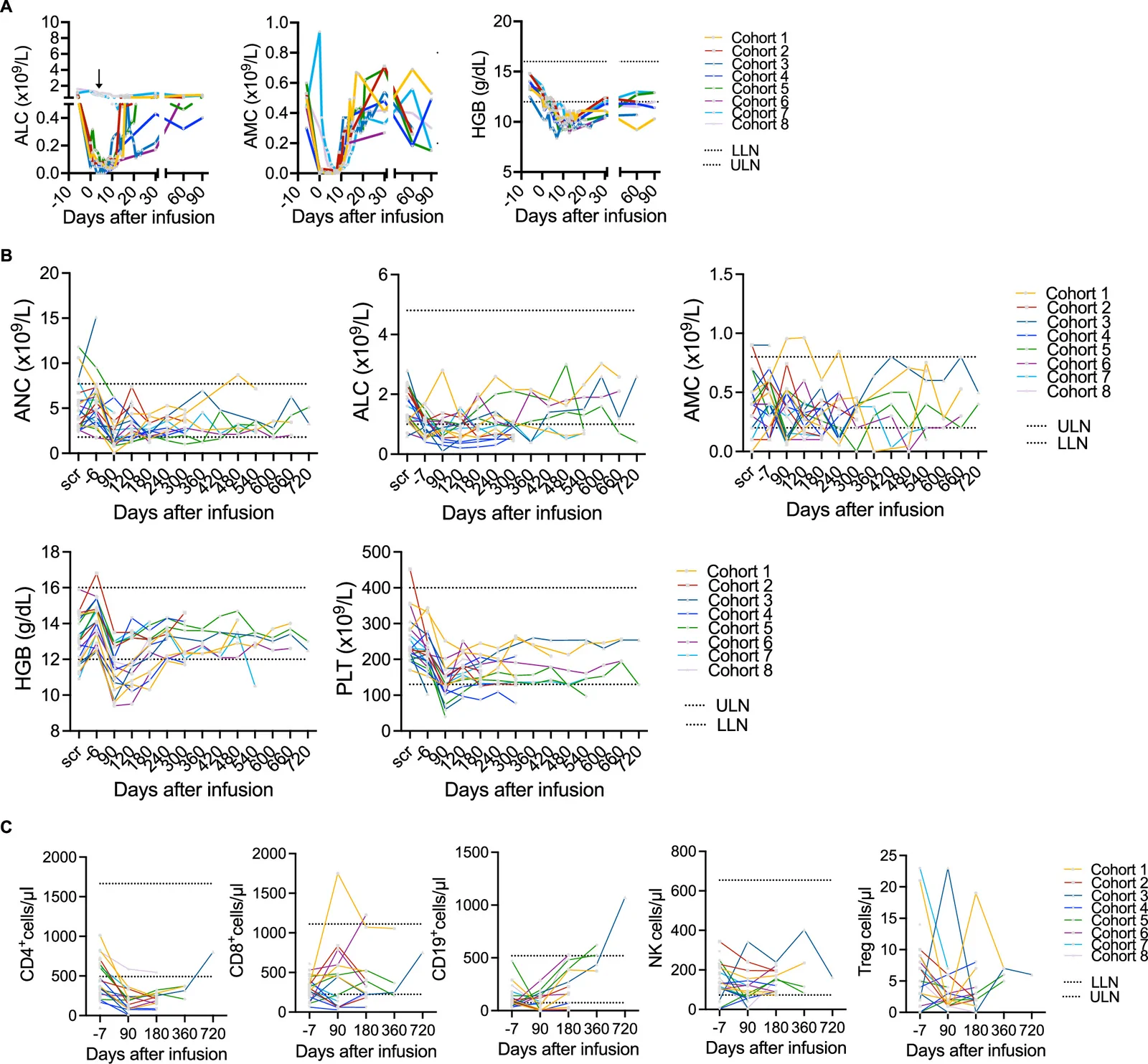
Hematologic recovery and immune reconstitution by cohort. (**A**) Absolute blood counts of lymphocytes (ALC), monocytes (AMC) and hemoglobin (HGB) levels during the first 90 days from Temferon infusion are shown (median, n = 3 per cohort), as determined by routine automated complete blood count. Arrow indicates stability of ALC in the cohorts receiving busulfan-only conditioning. ULN, upper limit of normal; LLN, lower limit of normal. (**B**) Absolute neutrophil counts (ANC), lymphocyte counts (ALC), monocyte counts (AMC), as well as hemoglobin levels (HGB) and platelet counts (PLT) in the blood during long-term follow up. ULN, upper limit of normal; LLN, lower limit of normal. (**C**) Lymphocyte subtyping in the blood by multicolor flow cytometry. Shown are the absolute counts of CD4 + T helper cells, CD8+ cytotoxic T lymphocytes, CD19 + B lymphocytes, natural killer (NK) cells (CD3-CD16+ or CD56+ within the lymphocyte gate), and CD4^+^CD25^high^CD127^−^ regulatory T cells (Tregs).

Hematologic recovery by day +30 was achieved in all patients and was consistent with ASCT including primary CNS lymphoma patients receiving BCNU/thiotepa conditioning (Fig. 2c,d). Median times to neutrophil and platelet recovery were days +14 and +19, respectively, with no differences across Temferon dose levels. Patients received, on average, five platelet transfusions to maintain platelet counts above 50 × 10^9^ l^−1^—a prudent threshold to prevent intracranial bleeding. In cohorts receiving busulfan monotherapy (cohorts 7 and 8), a delayed decline in neutrophil counts was observed, resulting in a shorter duration of grade 4 neutropenia, whereas total lymphocyte counts remained stable (Extended Data Fig. 1a) consistent with the known lack of acute lymphodepletion by busulfan^27^. Long-term blood counts and hemoglobin levels stabilized within the lower normal range across all Temferon dose levels, with CD4^+^ T cells taking longest to recover (Extended Data Fig. 1b,c). It is also noteworthy that patients who underwent second-line treatments or repeat craniotomy for tumor progression showed normal clinical courses, indicating no adverse effects of Temferon on BM regeneration or wound healing.

Longitudinal vector integration site analysis in peripheral blood (PB) and BM from Temferon-treated patients demonstrated a lentiviral insertion profile without evidence of insertional mutagenesis or clonal dominance (Supplementary Fig. 3).

### Secondary outcomes of efficacy

**Fig. 3:**
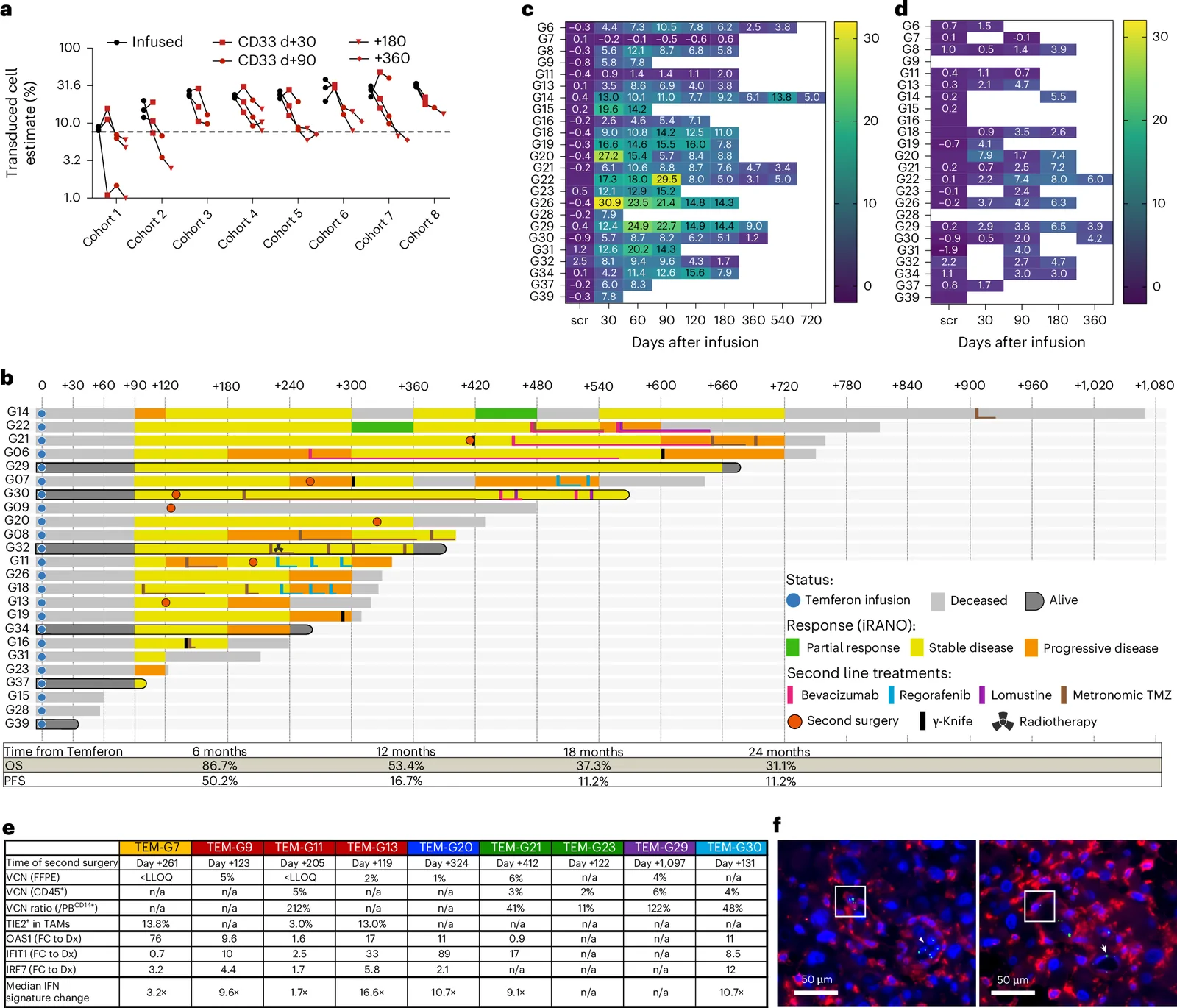
Clinical and biological efficacy endpoints. **a**, Engraftment of transduced BM CD33^+^ cells over time after Temferon infusion (red dots) estimated from VCN, compared with the infused input fraction of transduced CD34^+^ cells (black dots). The input fraction was calculated as: (CD34^+^ cells in the Temferon product × transduction efficiency in clonogenic progenitors)/(total CD34^+^ cells in Supporter plus Temferon products). **b**, Swim-lane plot ordered by OS (data cutoff, 20 June 2024), showing treatment timeline in days after Temferon infusion, radiologic response per iRANO criteria and additional treatments. γ-knife: stereotactic radiosurgery. **c**,**d**, IFNα levels in PB plasma (**c**) and CSF (**d**) measured longitudinally by ELISA. **e**, Summary of tumor biomarkers of Temferon activity. VCN was assessed in FFPE tissue or CD45^+^ tumor-infiltrating hematopoietic cells. VCN ratio indicates VCN in tumor CD45^+^ cells relative to VCN in CD14^+^ PB monocytes at the nearest matched timepoint. The proportion of TIE2^+^ TAMs (TEMs) was measured by flow cytometry (Extended Data Fig. 5). IFN-response genes (OAS1, IFIT1, IRF7) were quantified by qPCR on FFPE samples and expressed as 2^−ΔΔCt^ FC between second and first surgeries (FC to diagnosis (Dx)). The sample from TEM-G29 was collected after the interim analysis data cutoff. **f**, Representative immunofluorescence images from patient TEM-G09 at recurrence (two distinct tumor areas) showing CD14 (red) and RNAscope detection of TEK (TIE2) transcripts (green dots). Diagnosis-relapse sample pairs from three patients were analyzed, with similar findings (see also Extended Data Fig. 5e). Blue, nuclei; boxes, CD14^+^TEK^+^ TEMs; arrowheads, CD14^−^TEK^+^ cells; arrows, TEK^+^ endothelial cells; <LLOQ, below lower limit of quantification; n/a, not available (insufficient material); scr, baseline at screening.

**Extended Data Fig. 2:**
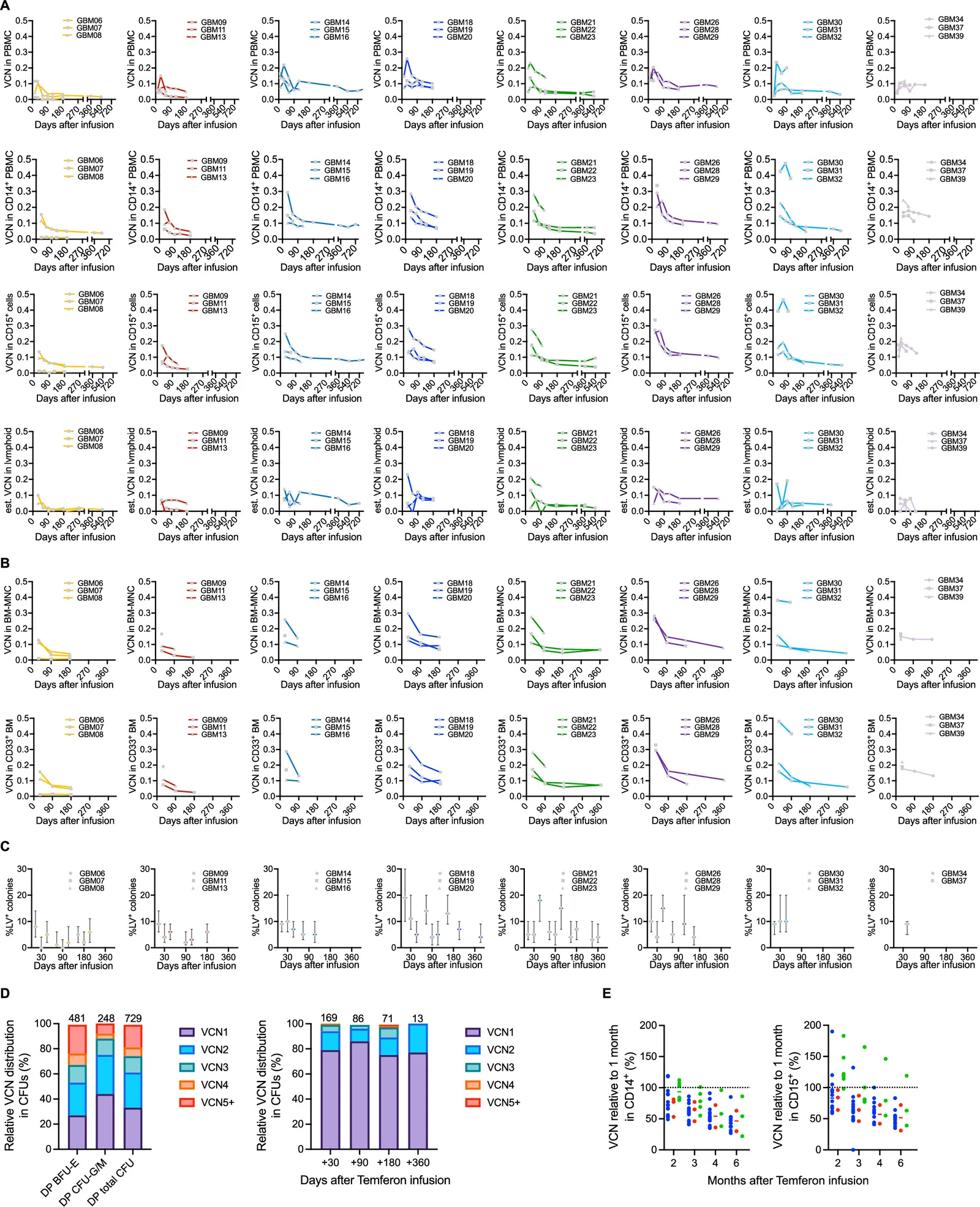
Engraftment of transduced cells after Temferon infusion. (**A**) Vector copy number (VCN) in peripheral blood cell subpopulations for individual patients, divided by cohort. PBMC, peripheral blood mononuclear cells. CD14^+^ cells, monocytes. CD15^+^ cells, granulocytes. VCN was estimated (est.) for lymphoid cells, as follows: est. VCN (lymphoid) = (VCN^PBMC^ - VCN^CD14+^ x F_CD14+_) / (1-F_CD14+_), whereby F_CD14+_ is the estimated fraction of CD14+ monocytes within PBMCs (absolute monocyte count/(absolute monocyte count + absolute lymphocyte count) at the given timepoint. (**B**) VCN in bone marrow mononuclear cells (BM-MNC) or BM CD33^+^ myeloid cells. (**C**) Engraftment of TIA126 LV-transduced clonogenic progenitors in the BM at the indicated timepoints post Temferon infusion. The symbols indicate the number of progenitor-derived colonies (CFU-G/M or BFU/CFU-E) tested positive for the LV (VCN > 0.5) divided by the total number of analyzed colonies. Error bars denote the 80% confidence interval calculated according to the method of Clopper and Pearson. An average of 35 individual CFU-G/M (minimum: 16) and 47 individual BFU/CFU-E (minimum: 38) were screened per patient and timepoint. (**D**) Vector copy number distribution within TIA126 LV+ (VCN > 0.5) colony-forming units (CFUs) from the drug products from all patients (left graph, total CFUs and split for erythroid BFU-E and myeloid CFU-G/M) and from BM CD34+ progenitors isolated at the indicated timepoints post Temferon infusion (right graph, shown for the total CFUs). The number above the bars indicate the number of individual TIA126 LV+ colonies analyzed. VCN 1 (0.5-1.4); VCN 2 (1.5-2.4); VCN 3 (2.5-3.4); VCN 4 (3.5-4.4); VCN5+ (4.5-20); VCN values > 20 were rejected because not plausible (likely technical error). (**E**) VCN in CD14+ monocytes and short-lived, CD15+ granulocytes at 2, 3, 4 and 6 months post Temferon infusion, normalized to the first in vivo datapoint at 1 month for each patient. Blue dots: Carmustin (BCNU) + thiotepa conditioning; Red dots: Busulfan + thiotepa; Green dots: Busulfan monotherapy.

All Temferon-treated patients engrafted with gene-marked cells in the PB and BM (Extended Data Fig. 2a–c). Engraftment, estimated by VCN in CD33 + BM progenitors, peaked at day +30 and closely matched the inferred input of TIA126-transduced CD34^+^ cells, indicating no competitive disadvantage relative to unmanipulated supporter cells (Fig. 3a). Transduced myeloid cells persisted in all patients declining over time and stabilizing at approximately half of the initial engraftment in most patients through the last FU. Because most engrafted clonogenic Temferon progenitors contained a VCN of ~1 throughout all timepoints (Extended Data Fig. 2d) and showed no selective disadvantage in xenograft experiments (Supplementary Fig. 2), the early decline observed between 1 and 3 months is probably attributable to submyeloablative conditioning, allowing recovery of endogenous HSCs that diluted the output of transduced progenitors. In vivo gene marking increased progressively from cohort 1 to 6 and then plateaued. Both BCNU and busulfan supported long-term engraftment, with a trend toward more stable early engraftment in patients receiving busulfan monotherapy (Extended Data Fig. 2e).

**Extended Data Fig. 3:**
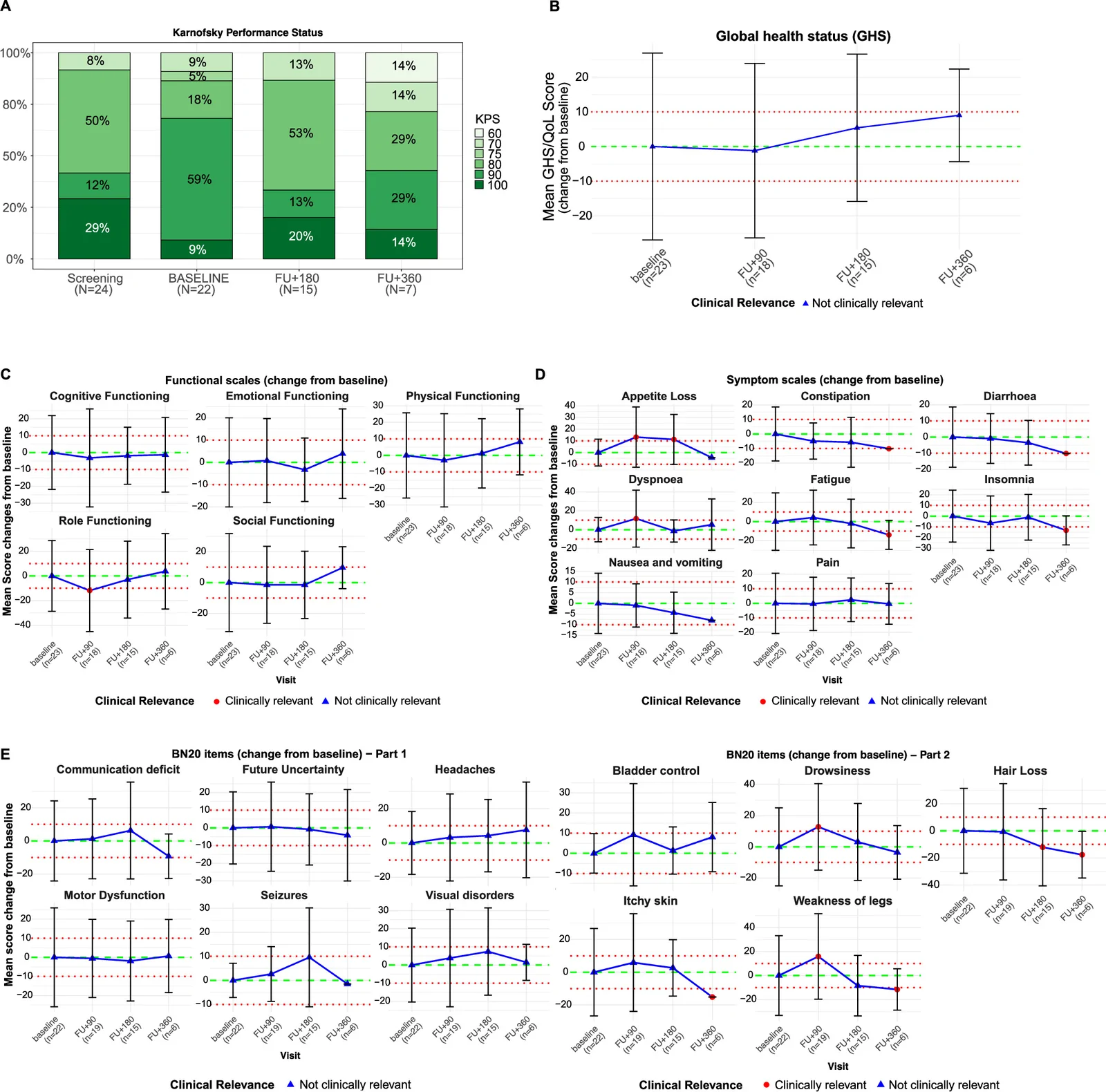
Performance status and quality of life after Temferon treatment. (**A**) Distribution of patients according to Karnofsky Performance Status at screening, baseline (after adjuvant radiotherapy, before the start of conditioning chemotherapy), day +180 and day +360 from Temferon administration. N: number of evaluable patients at the indicated timepoint. (**B-D**) Patient-reported, health-related quality of life assessed by the EORTC QLQ-C30 v.3.0 questionnaire. A change of ≧ 10 points as compared to baseline was considered clinically relevant. The graph shows the average change in score ± SD. The dotted green line indicates the mean value at baseline, while the red line shows the cutoff for clinically relevant changes ( ± 10 points). The number of evaluable patients (n) is indicated in the graph. Kruskal-Wallis test did not highlight statistically significant changes from baseline (p > 0.05). (**B**) Global health status (items 29, 30) at day +90, +180 and +360 as compared to baseline. Higher scores mean better quality of life. (**C**) Functional scales (items 1-7 and 20-27). Higher scores mean better functional performance. (**D**) Symptom scales (items 8-19 and 28). Higher scores mean more symptoms (and, hence, poorer quality of life). (**E**) Brain tumor module (BN20) from the EORTC QLQ-C30 v.3.0 questionnaire. The graphs show the average change in score ± SD in the indicated domain. The dotted green line indicates the mean value at baseline, while the red line shows the cut-off for clinically relevant changes ( ± 10 points). Higher scores mean more disability. Kruskal-Wallis test did not highlight statistically significant changes from baseline (p > 0.05).

At the data cutoff (20 June 2024), the median FU since Temferon infusion was 39 months. Only cohort 8 had less than 1 year FU (36–191 days). Applying immunotherapy response assessment for neuro-oncology (iRANO) criteria, we did not observe objective responses at +6 months, but most evaluable patients (75%, 12 out of 16) had stable disease, which persisted at +12 months in all nine evaluable patients (Fig. 3b). Notably, two long-term survivors achieved late partial responses at days +300 and +420, respectively, without additional therapy. Patient TEM-G14, who survived 39 months, had demonstrated early progression during adjuvant radiation therapy before Temferon infusion—a known adverse prognostic feature. These findings indicate that Temferon does not induce rapid tumor regression (as seen in patients receiving CAR-T therapies) but may promote prolonged disease stabilization. Median overall survival (OS) and progression-free survival (PFS) were, respectively, 16.7 and 8.1 months from diagnosis. OS and PFS rates, measured from the time of Temferon infusion, were 53.4% and 16.7% at 12 months and 31.1% and 11.2% at 24 months (Fig. 3b). Most of the patients (80%) maintained a stable Karnofsky performance status at the +180-day FU, which remained ≥80% in five out of seven evaluable patients on day +360 (Extended Data Fig. 3a). Quality-of-life assessments (EORTC QLQ-C30 v.3.0) showed stable global health and functional scales over 1 year, with a trend toward improvement in general symptom scores (Extended Data Fig. 3b–d). The brain-tumor-specific QLQ-BN20 module demonstrated overall neurological stability, aside from a trend toward more seizures at 6 months (Extended Data Fig. 3e).

These preliminary phase 1 data support the feasibility of Temferon and suggest sustained biological activity in GBM patients.

### Post hoc analyses show recruitment of Temferon progeny to the GBM TME and induction of local IFNα response

**Extended Data Fig. 4:**
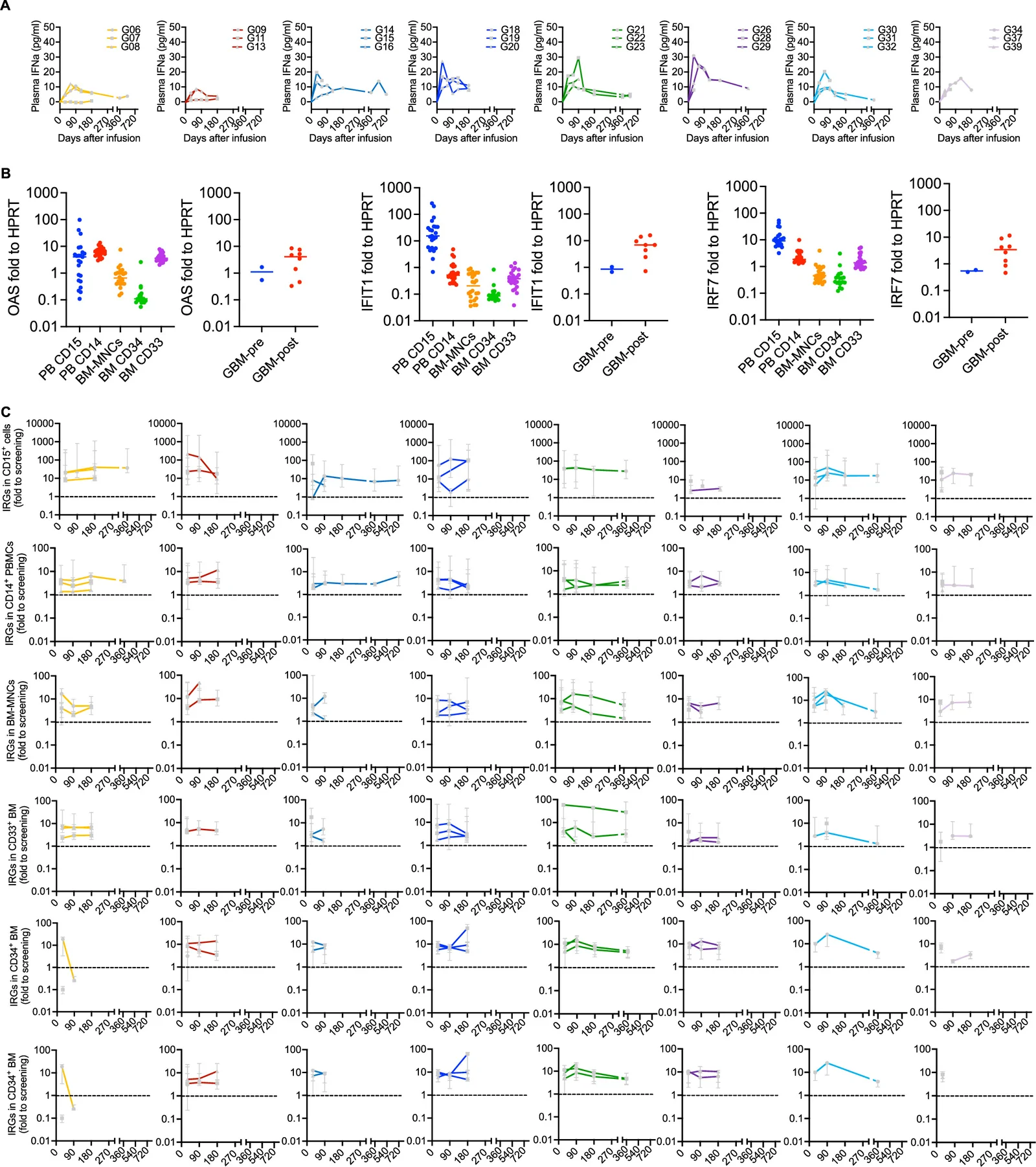
Biomarkers of Interferon activity. **A**) Interferon-alpha (IFN-α) concentrations over time in the plasma, measured by ELISA and divided by cohort. Individual patients are shown. (**B**) Transcript expression of 3 representative interferon response genes (IRGs) relative to the Hypoxanthine Phosphoribosyltransferase 1 (HPRT) housekeeping gene measured by digital droplet PCR in peripheral blood (PB) or bone marrow mononuclear cell (BM-MNC) subpopulations at screening, that is before Temferon treatment (*baseline expression*, left graphs, for each IRG), as well as in bulk tumor (right graphs, for each IRG) at first surgery (GBM-pre Temferon) or at second surgery (GBM-post Temferon). Each dot represents a patient, the line indicates the median. Note the cell population-intrinsic differences in baseline IRG expression. OAS1, Oligoadenylate Synthetase 1; IFIT1, Interferon Induced Protein With Tetratricopeptide Repeats 1; IRF7, Interferon Regulatory Factor 7. (**C**) Relative change of IRG expression in peripheral blood or bone marrow subpopulations at the indicated timepoint (days) after Temferon infusion, grouped by subpopulation and cohort. Shown is the median fold change of the OAS1/HPRT, IFIT1/HPRT and IRF7/HPRT values with respect to baseline (value at screening, see panel B) for individual patients, error bars indicate the range for the relative change of the 3 IRGs. Individual patients are shown.

Despite substantial in vivo gene marking with TIA126, reaching up to 40–50% of blood leukocytes in one patient from cohort 7, plasma IFNα levels remained consistently low (<31 pg ml^−1^) during the first 3 months and showed no clear correlation with VCN (Fig. 3c and Extended Data Fig. 4a). However, IFN-responsive gene (IRG) signatures were induced in PB/BM myeloid subpopulations following Temferon infusion, remained stable over time and did not differ between cohorts (Extended Data Fig. 4b,c). To assess CNS penetration and local activity, IFNα levels were measured in cerebrospinal fluid (CSF) (Fig. 3d). IFNα was undetectable at screening (<1.95 pg ml^−1^) in all but one patient but became measurable in 6 out of 12 (50%) evaluable patients at day +30, 11 out of 15 (73%) at day +90 and all patients at days +180 and +360. Peak CSF levels were observed frequently at day +180, consistent with progressive and sustained recruitment of genetically engineered hematopoietic cells into the TME.

Exploratory (post hoc) analyses were performed on tumor samples from nine patients, including one autopsy specimen and eight samples obtained at second surgery. Vector copies were detected in tumor-derived DNA from all evaluable samples (VCN range 1–6%), except for one patient from cohort 1 who had the lowest systemic engraftment (Fig. 3e). Comparison of VCNs in CD45^+^ tumor digests with matched PB CD14^+^ monocytes revealed that tumor leukocytes contained a median of 48% (range 11–212%) of the monocyte VCN, indicating substantial replenishment of TAMs from the Temferon graft, still detectable more than 3 years after treatment (TEM-G29).

**Extended Data Fig. 5:**
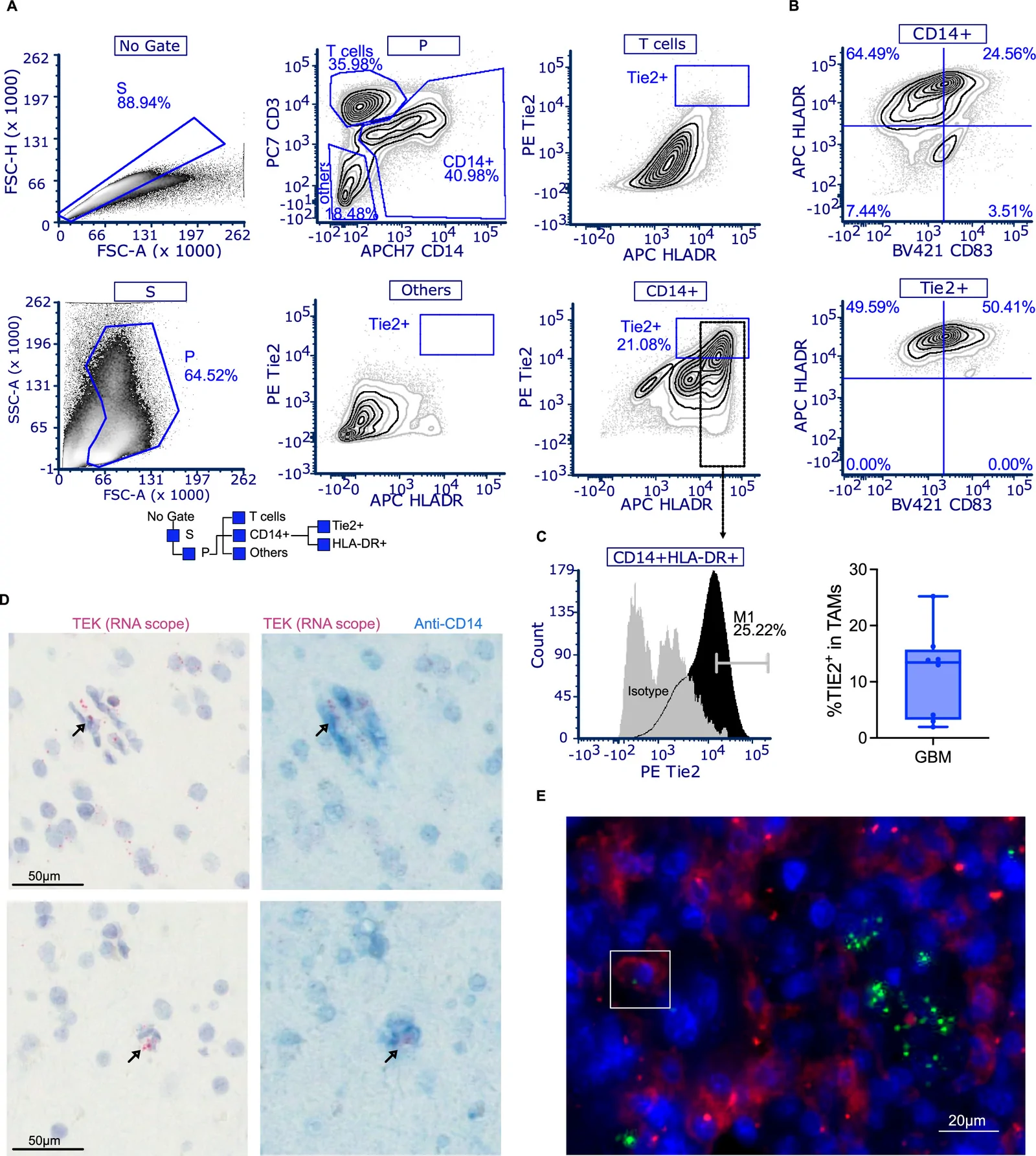
TEK (TIE2) expression in GBM-associated macrophages. (**A**) Flow cytometric analysis of CD45 + -enriched cells from dissociated GBM tumors. The gating strategy to identify TIE2-expressing monocytes/macrophages (TEMs) is illustrated for a representative sample. Note that TIE2 staining is limited to CD14 + HLA-DR+ myeloid cells; gates for TIE2+ staining were set based on an isotype control (background <1% within the CD14+ or CD14 + DR+ gate). (**B**) Expression of CD83, a molecule involved in antigen presentation and associated with inflammatory macrophages, on total CD14+ cells or TEMs. (**C**) (Left) Representative histograms showing TIE2 (TEK) expression (black) as compared to isotype control (grey) on CD14^+^ HLA-DR^high^ tumor-infiltrating myeloid cells. (Right) Quantification of TEMs within the total CD14^+^ HLA-DR^high^ tumor-infiltrating myeloid cell population (n = 8 samples, 4 from patients post Temferon treatment, and 4 from control GBM patients). Box and whisker plot shows the median, interquartile range and min/max values, each datapoint represents a sample. (**D**) Representative bright field images of combined immunostaining for TEK (pink-red dots, RNAscope, left) and CD14 protein (blue, right) from patient TEM-G20. The arrows show examples of TEMs. Similar findings have been obtained on 3 additional patients. (**E**) Representative immunofluorescence image of combined immunostaining for TEK (green dots, RNAscope), CD14 protein (red) and nuclei (DAPI, blue) from patient TEM-G09 at diagnosis. Diagnosis-relapse sample pairs from 3 patients have been analysed, with similar findings (see also Fig. 3f).

Surface antibody staining for TIE2 (TEK) identified a variable proportion (2–25%) of TEMs within the GBM TAM compartment (Fig. 3e and Extended Data Fig. 5a–c). RNAscope analysis confirmed the presence of TEK^+^ myeloid cells within GBM tissue sections (Fig. 3f and Extended Data Fig. 5d,e). Moreover, tumors obtained at second surgery exhibited up to tenfold higher IRG expression compared with matched primary resection samples (Fig. 3e and Extended Data Fig. 4b). Of note, the tumor from TEM-G11 that had the lowest proportion of TEMs showed the weakest IRG induction.

Collectively, these data demonstrate recruitment and persistence of Temferon progeny within the GBM TME and support the proposed mechanism of action involving local IFNα release by transduced TEMs.

### Temferon reprograms the GBM TME

**Fig. 4:**
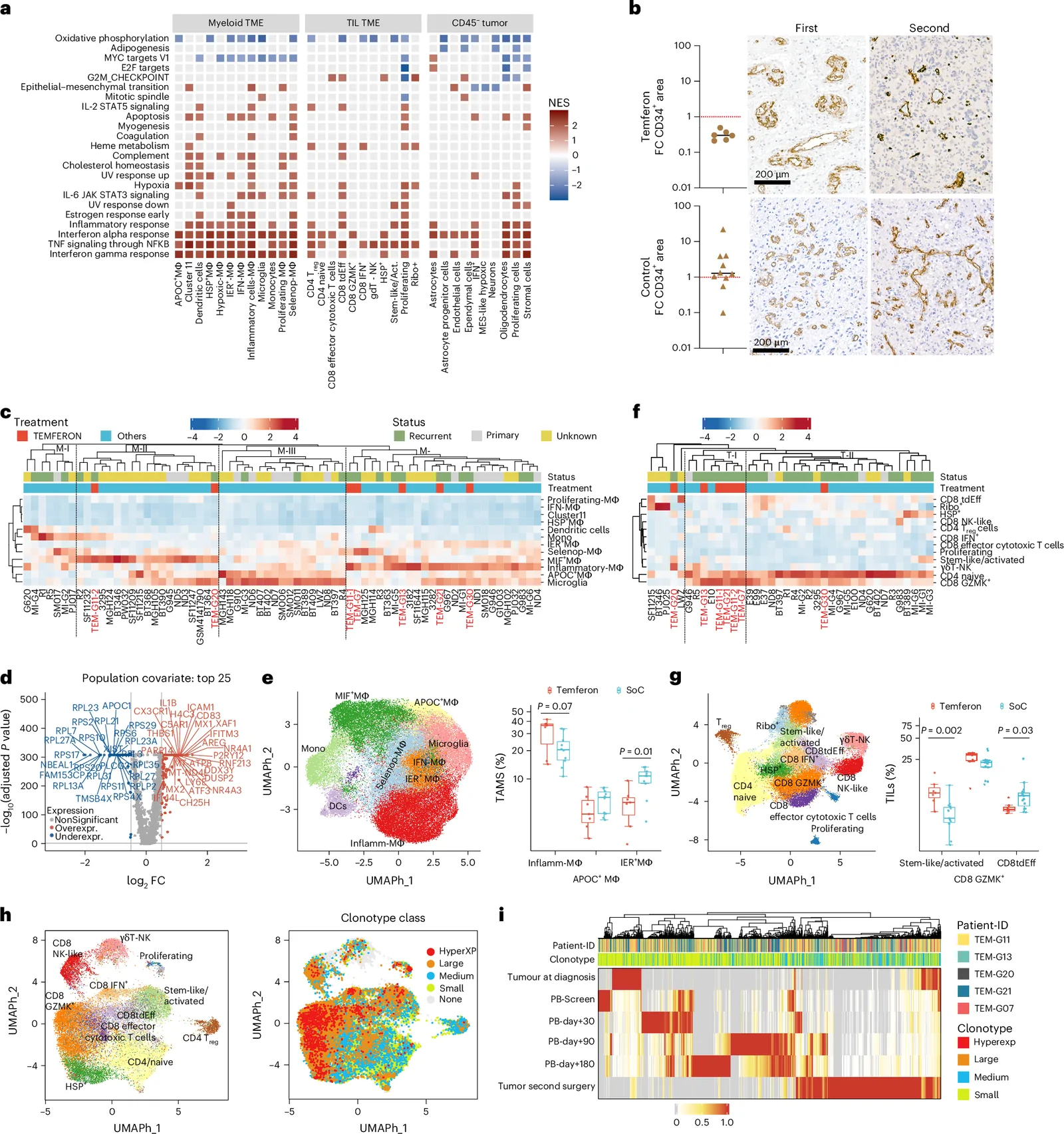
scRNA-seq analysis of the Temferon TME. **a**, Intracluster GSEA using preranked logFC gene lists from DEGs comparing Temferon-treated tumors (*n* = 7) with controls (extended dataset, *n* = 38–69; Supplementary Data 2). Normalized enrichment scores (NES) for significantly enriched or depleted MSigDB Hallmark pathways (Benjamini–Hochberg-adjusted *P* < 0.01) detected in at least two populations are shown. **b**, Change in blood vessel density (ΔCD34^+^ area) between diagnosis and recurrent tumors in the TEM-GBM_001 cohort (*n* = 6; TEM-G7, -G9, -G11, -G13, -G20, -G21) or matched controls (*n* = 10; MI-G2, -G6 and BS cohort; Supplementary Table 8). Each dot represents one patient. *P* = 0.008 (Mann–Whitney test, two-sided). Representative CD34 IHC images are shown. **c**,**f**, Heatmaps of myeloid (**c**) and lymphoid (**f**) subpopulation frequencies per sample in the extended dataset with unsupervised clustering. **d**,**e**,**g**, Comparison of post Temferon GBM samples (*n* = 7) with matched recurrent GBM controls (*n* = 10 for myeloid, *n* = 14 for lymphoid dataset; Supplementary Table 8). **d**, Volcano plot of DEGs in the myeloid TME (Temferon versus matched controls) using the MAST method with population as covariate (two-sided test, *P* values were adjusted for multiple testing using the Benjamini–Hochberg FDR). **e**, Myeloid subsets annotated by label transfer from the extended dataset; boxplots show subset frequencies, with significant differences or trends between Temferon (*n* = 7 biological replicate samples from six patients) and SoC (*n* = 10 patients) groups (two-sided Wilcoxon rank sum test; exact *P* values < 0.1 are shown). Shown are values for individual patients (dots), median, first and third quartile (boxplots) and minimum/maximum datapoints falling within 1.5× the interquartile range (whiskers). **g**, As in **e**, for TILs. SoC, *n* = 14 patients. **h**, Uniform manifold approximation and projection (UMAP) embedding of the TIL compartment (internal dataset, 37,392 cells; Supplementary Data 1) colored by T cell receptor clonotype frequency (hyperexpanded (HyperXP), 10–100%; large, 1–10%; medium, 0.1–1%; small, 0.01–0.1%; none, *n* = 12,549 cells lacking T cell receptor β sequences). **i**, Backtracking of scTCR-seq TIL clonotypes from Temferon-treated patients to bulk TCR-seq data from first or second tumor resections and PB at the indicated timepoints. MΦ, macrophages.

**Extended Data Fig. 6:**
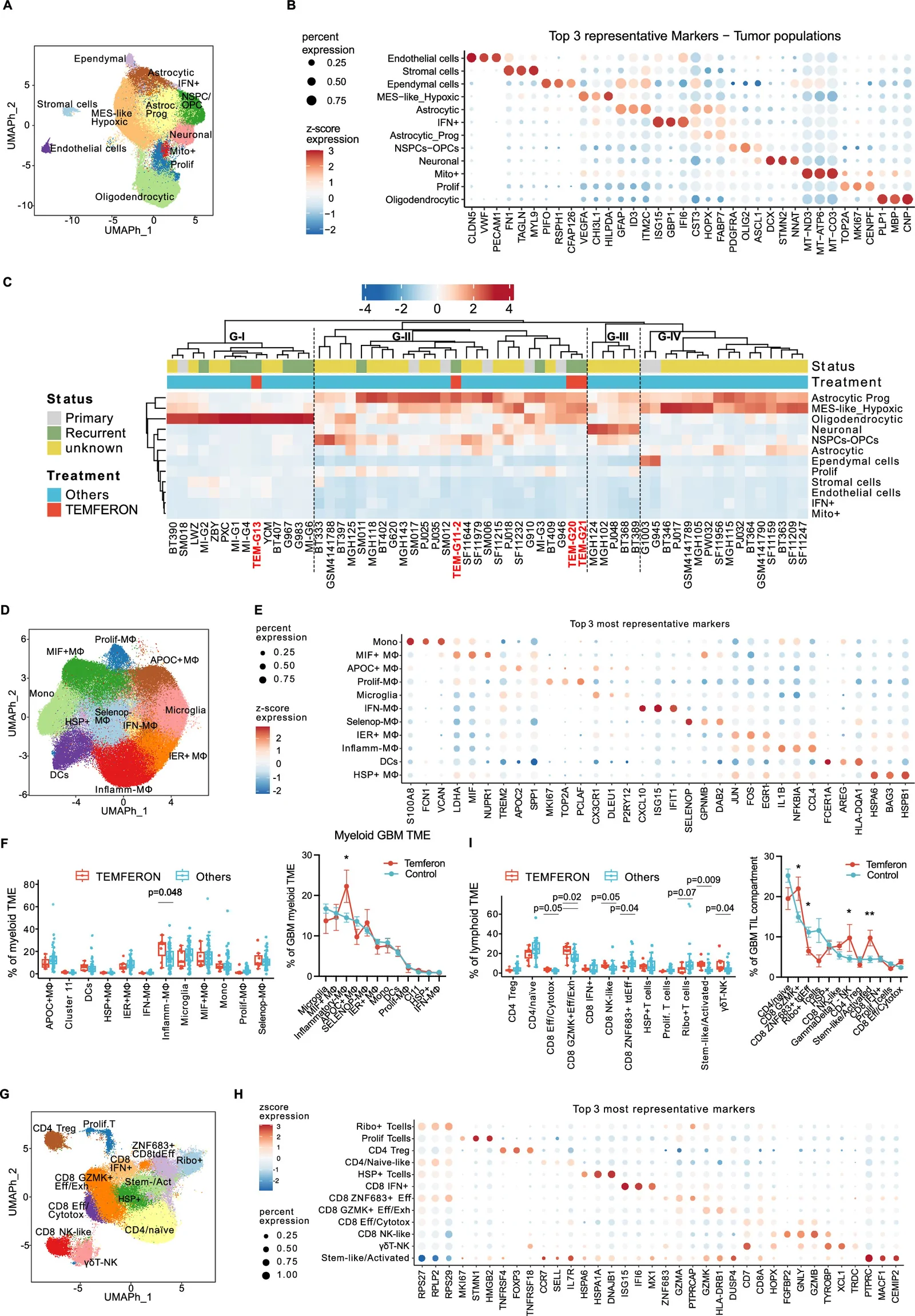
Integrated GBM landscape from an extended scRNA-seq dataset. (**A**) UMAP with annotation of the non-hematopoietic (CD45^neg^) cell compartment composed mainly of tumor cells (61 patients n = 142,173 cells). (**B**) Dot plot showing the top three marker genes for annotated CD45^neg^ tumor cell populations. (**C**) Heatmap of cell type frequencies per patient (n = 61), with unsupervised clustering into four GBM macro-clusters: G-I, oligodendrocytic; G-II, astrocytic; G-III, neural; G-IV, mesenchymal-like. Only samples contributing ≥100 cells were included. The heatmap shows column-scaled numbers per population and sample. (**D**) UMAP of tumor-associated myeloid cells in the GBM microenvironment (n = 69 samples, ~150,000 cells). The following major macrophage subpopulations were distinguished: MIF+ and APOC+ macrophages, which expressed several other genes associated with immune suppressive functions, such as SPP1 and TREM2; Inflammatory and intermediate early response (IER) gene macrophages/microglia, characterized by the expression of activation markers (JUN and FOS) and proinflammatory cytokines and signals (IL-1B, CCL4, NFKB1A); SELENOP+ macrophages, that showed an intermediate phenotype, between the above mentioned, more polarized subsets. (**E**) Dot plot of representative marker genes for annotated myeloid populations; dot size indicates the fraction of expressing cells and color indicates z-scored expression across populations. (**F**) Relative frequencies (% of tumor-associated myeloid cells) of each annotated cluster in Temferon-treated samples (n = 7 biological replicate samples from 6 patients) versus other treatments (controls; n = 62 patients). (**G**) UMAP of tumor-infiltrating lymphocytes (TILs; n = 38 samples, ~80,000 cells). (**H**). Dot plot of representative marker genes for annotated T cell populations. (**I**) Relative frequencies (% of TILs) of each annotated cluster in Temferon-treated samples (n = 7 biological replicate samples from 6 patients) versus other treatments (controls; n = 31 patients). (**F,I**) Box-and-whisker plots show values for individual patients (dots), median, 1^st^ and 3^rd^ quartile (boxplots) and minimum/maximum datapoints falling within 1.5x the interquartile range (whiskers). Significance was tested by two-sided Wilcoxon rank sum test, exact p values with p < 0.1 are shown. Line plots show mean ± s.e.m. *P < 0.05, **P < 0.01.

Next we performed single-cell RNA sequencing (scRNA-seq) on dissociated tumors from *n* = 6 trial patients after Temferon infusion, as well as *n* = 6 recurrent GBM from patients treated with standard of care (SoC) (Supplementary Table 8) and undergoing second surgery (‘internal dataset’; Supplementary Fig. 4 and Supplementary Data 1). To obtain a more comprehensive analysis, we integrated public scRNA-seq data to create an extended GBM dataset with a simplified, manually curated classification of cell populations (Extended Data Fig. 6 and Supplementary Data 2). Intracluster geneset enrichment analysis (GSEA) showed universal enrichment of IFN and inflammatory pathways across the entire tumor ecosystem in Temferon-exposed tumors, indicating pervasive IFNα activity (Fig. 4a). Temferon treatment was also associated with reduced oxidative phosphorylation and MYC signaling, increased apoptosis and hypoxia consistent with antiproliferative, cytotoxic and anti-angiogenic effects. Congruently, longitudinal quantification of the CD34^+^ blood vessel area from diagnosis to recurrence (Fig. 4b) showed a significant reduction in Temferon patients, but not in a matched control cohort (Supplementary Table 8).

**Extended Data Fig. 7:**
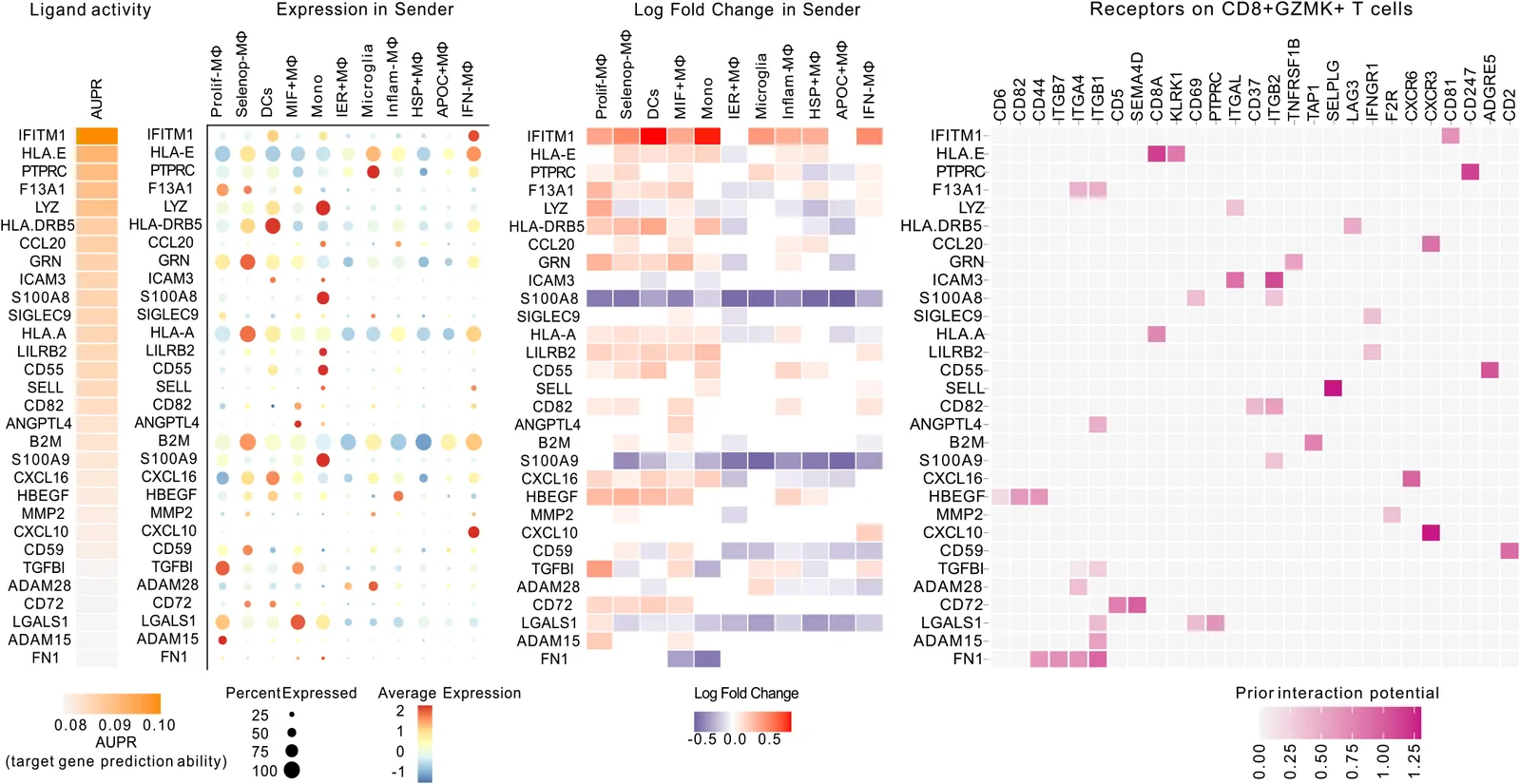
NicheNet analysis of interactions between GBM-associated myeloid cells and GZMK + CD8 effector T cells. Heatmaps show 30 prioritized ligands and their expression in GBM-associated myeloid cells (senders, left), log fold change (LFC) of ligand expression in Temferon-treated versus control samples (center), and interaction potential scores between prioritized ligands and downstream target genes in GZMK + CD8 T cells (right). Top-ranked upregulated ligand–receptor pairs were associated with IFN-α signaling (IFITM1–CD81), TNF signaling (GRN–TNFRSF1B), antigen presentation (HLA.E–CD8A/KLRK1, HLA-DRB5–LAG3, HLA.A–CD8A, B2M–TAP1), and T cell activation (CD55–ADGRE5). Chemokine axes linked to T cell infiltration and antitumor immunity, including CXCL16–CXCR6 and CXCL10–CXCR3, were also identified.

A focus on the myeloid landscape highlighted four macro-clusters: M-I, monocyte-rich, mostly seen in recurrent GBM; M-II, MIF+ macrophage-rich; M-III, microglia/APOC^+^ macrophage-rich; and M-IV, inflammatory macrophage-rich (Fig. 4c). Five out of seven post Temferon samples (71%) clustered in M-IV, whereas only two clustered in M-II. In contrast, only 34% of control cases clustered as M-IV, with most categorized as M-II or M-III. We then compared the seven Temferon samples with ten matched control cases of recurrent GBM (Supplementary Table 8 and Supplementary Data 3). Differential gene expression (DGE) analysis controlling for cell population highlighted upregulation of several IFN-stimulated genes and inflammatory activation markers, whereas APOC1 and ribosomal proteins were downregulated (Fig. 4d), consistent with a shift from M2-like macrophage populations to M1-like inflammatory subtypes (Fig. 4e), which was statistically significant in the extended dataset (Extended Data Fig. 6f). NicheNet analysis predicted several upregulated ligand/receptor pairs between inflammatory TAMs and tumor-infiltrating T lymphocytes (TILs) upon Temferon treatment (Extended Data Fig. 7) suggesting secondary reprogramming of the TIL compartment.

**Extended Data Fig. 8:**
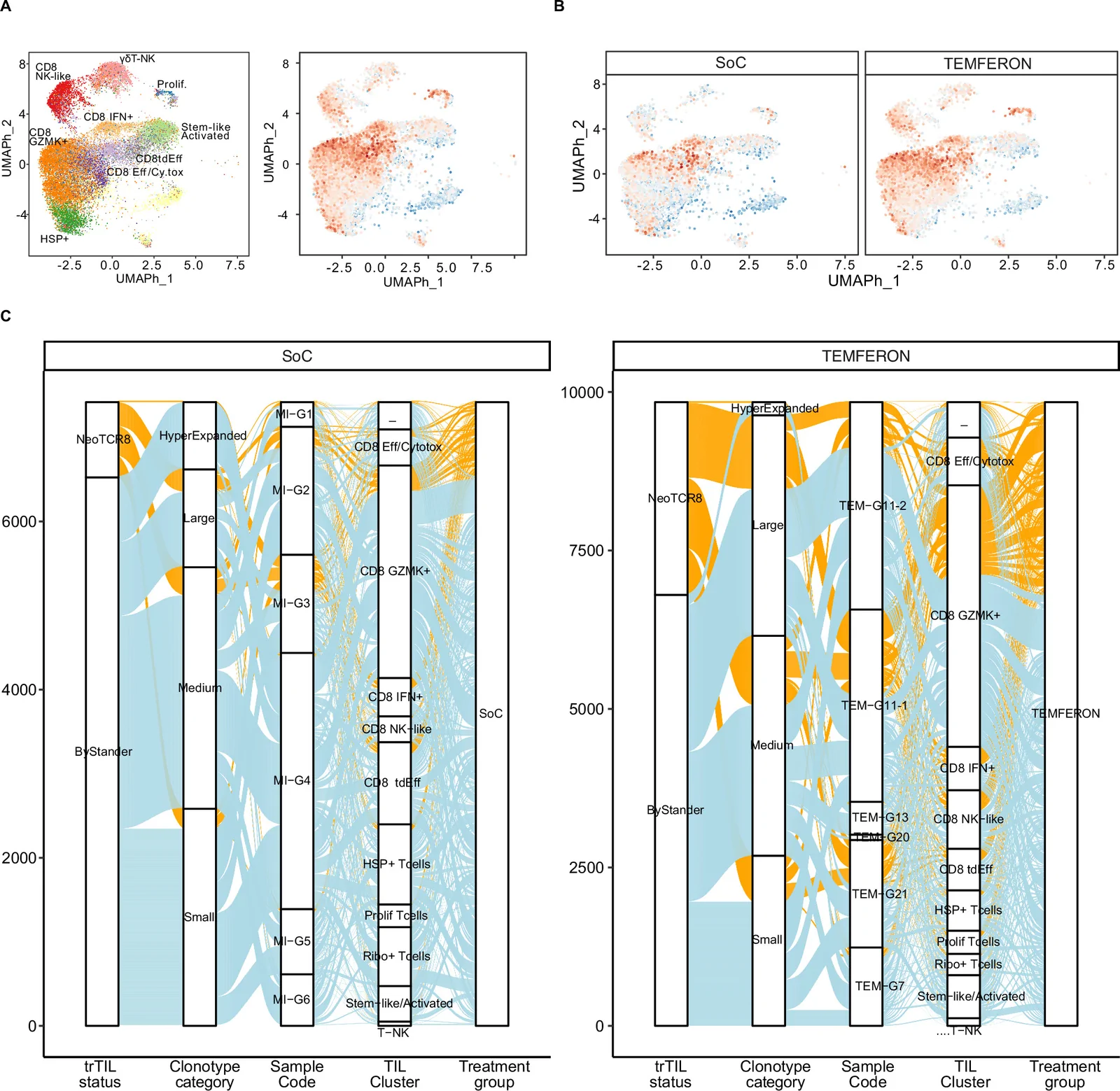
Identification of predicted tumor-reactive CD8 TILs. (**A**) Projection of predicted tumor-reactive TILs (trTILs), defined by the NeoTCR8 signature^28^, onto the CD8 T cell UMAP with internal dataset embeddings. (**B**) UMAPs split by standard-of-care (SoC) controls and Temferon-treated patients. (**C**) Alluvial plot of TCRβ clonotypes classified as trTILs (high NeoTCR8 signature, yellow) or bystander T cells (light blue), shown by sample origin (TEM-GXX, Temferon; MI-GX, SoC at recurrence), CD8 T cell subcluster, and clone size.

TILs in GBM were composed primarily of naive and CD4^+^ cells, as well as variable amounts of granzyme K (GZMK)^+^CD8 effector cells (Fig. 4f and Extended Data Fig. 6g,h). Strikingly, five out of seven Temferon GBM samples formed a nearly private macro-cluster (T-I), which differed from the most prevalent T-II cluster by a statistically significant increase in GZMK^+^CD8 effectors (Extended Data Fig. 6I). Comparing the Temferon samples with 14 immunotherapy-naive, matched controls (Supplementary Table 8 and Supplementary Data 3), we confirmed a trend towards more GZMK^+^CD8 T lymphocytes and significantly increased stem-like/ activated T cells (probably corresponding to antigen-experienced, metabolically repressed effector memory TILs) in Temferon-treated patients, whereas the cluster containing terminally differentiated effector CD8 cells (CD8tdEff) was decreased significantly (Fig. 4g). Single-cell T cell receptor (TCR) sequencing (scTCR-seq) on our comparably annotated internal dataset (Supplementary Fig. 4e) highlighted the presence of large and hyperexpanded clones within the CD8 effector compartment, particularly in the GZMK^+^CD8 subset (Fig. 4h). Several expanded clones were selectively enriched inside the tumor, as determined by bulk TCR-seq (immunoSEQ) of longitudinal tumor and blood samples, and most of them appeared de novo in the recurring lesion (Fig. 4I and Supplementary Data 1). Moreover, a pan-cancer CD8 T cell signature (neoTCR8) predicting tumor reactivity^28^ also mapped to the region encompassing the CD8 effector subsets and was particularly abundant in patients TEM-G11 and -G21 (Extended Data Fig. 8).

Overall, this granular analysis of the TME uncovers a specific impact of Temferon on the macrophage and TIL compartments favoring inflammatory activation of the former and induction and accumulation of T cell subpopulations with several features of tumor-reactive CD8 effector cells.

### Evidence for the development of antitumor immunity

**Fig. 5:**
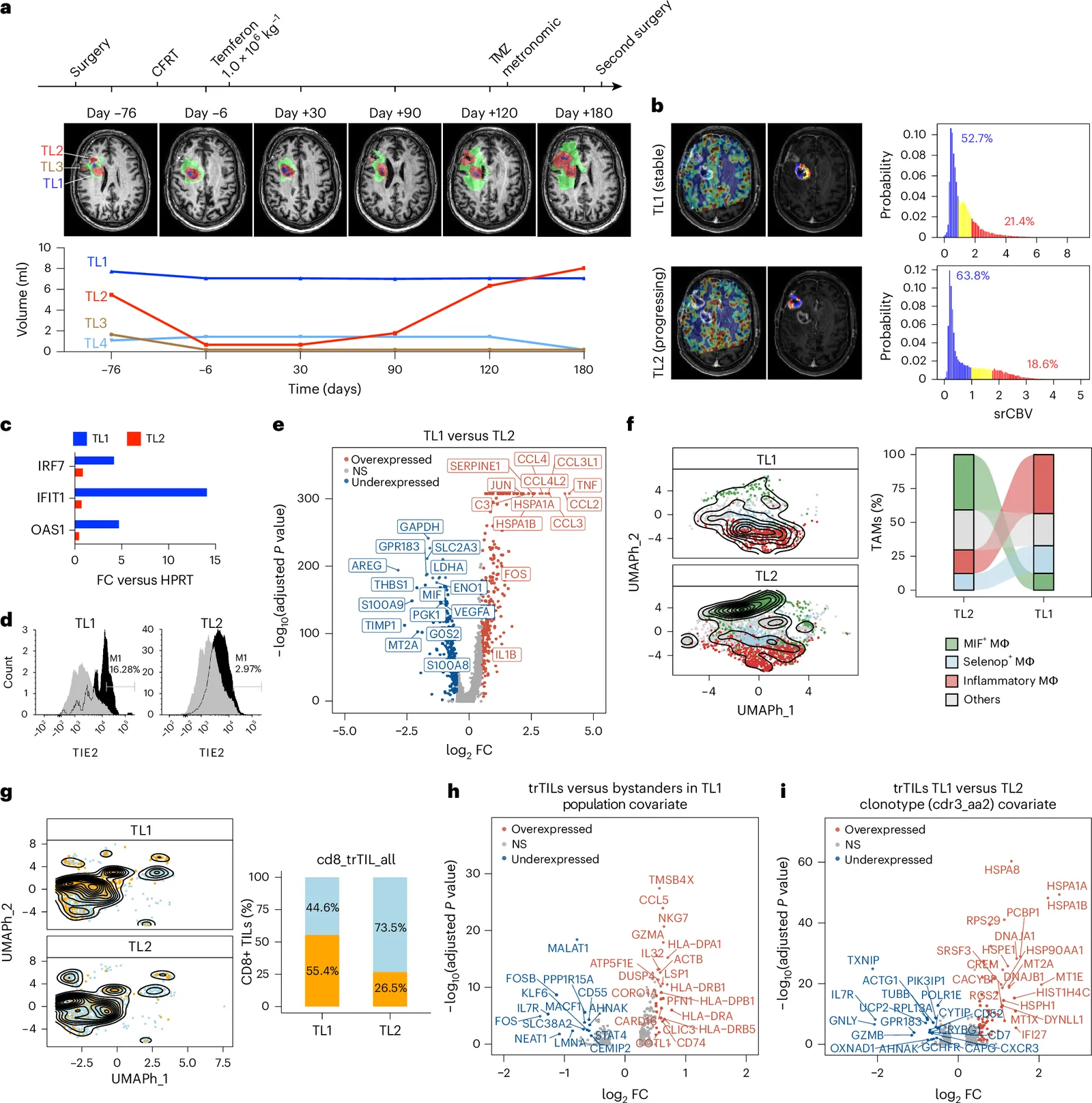
Intrapatient heterogeneity of Temferon response in patient TEM-G11. **a**, Radiologic evolution of four target lesions (TLs) over time, with treatment timeline between MRI assessments indicated above. Volumes of all TLs are shown; sagittal sections depict the three largest lesions (red: contrast-enhancing tumor; blue: necrosis; green: perilesional edema). **b**, Tumor blood perfusion (postcontrast T1 images) of TL1 and TL2 measured on day +120 as srCBV in enhancing tumor regions. Perfusion-based fractional tumor burden is classified as low (blue: rCBV < 1.0), high (red: rCBV > 1.75) or indeterminate (yellow). **c**, IRG expression (OAS1, IFIT1, IRF7; FC versus HPRT) measured by ddPCR in biopsies from stable TL1 (blue) and progressing TL2 (red). **d**, TIE2 surface expression on TAMs (CD45^+^CD14^+^HLA-DR^+^) from TL1 and TL2 assessed by flow cytometry, with isotype controls. **e**, DEGs in the myeloid compartment of TL1 versus TL2 identified from scRNA-seq data using a bulk analytical approach (Wilcoxon method). **f**, UMAP plots with simplified annotation of the macrophage compartment from TL1 and TL2, with relative population frequencies. **g**, UMAP projection and fraction of CD8^+^ T cells classified as tumor-reactive TILs (trTILs; yellow: NeoTCR8_ALL score above threshold) or bystander T cells (light blue: below threshold), with threshold defined by the minimum score of the top-scoring cluster. **h**, Volcano plot of DEGs (MAST method) between trTILs and bystander T cells in stable TL1, using T cell subpopulation as a covariate; top 20 genes by logFC are labeled. **i**, Volcano plot of DEGs (MAST method) between trTILs from stable TL1 and progressing TL2, using T cell receptor β clonotype as a covariate; top 20 genes by logFC are labeled. Two-sided tests were used in **e**, **h** and **i**, and *P* values were adjusted for multiple testing using Benjamini–Hochberg FDR. NS, nonsignificant.

We next analyzed target lesions (TLs) with divergent clinical trajectories in patient TEM-G11, enabling an intrapatient comparison of TME characteristics associated with stability versus progression. After radiotherapy and Temferon treatment, TL1 and TL4 remained stable and TL3 decreased, whereas TL2 progressed and led to reoperation and, ultimately, death on day +340 (Fig. 5a). Functional magnetic resonance imaging (MRI) at day +120 showed lower standardized relative cerebral blood volume (srCBV) in TL2 consistent with reduced perfusion and a more hypoxic TME (Fig. 5b).

**Extended Data Fig. 9:**
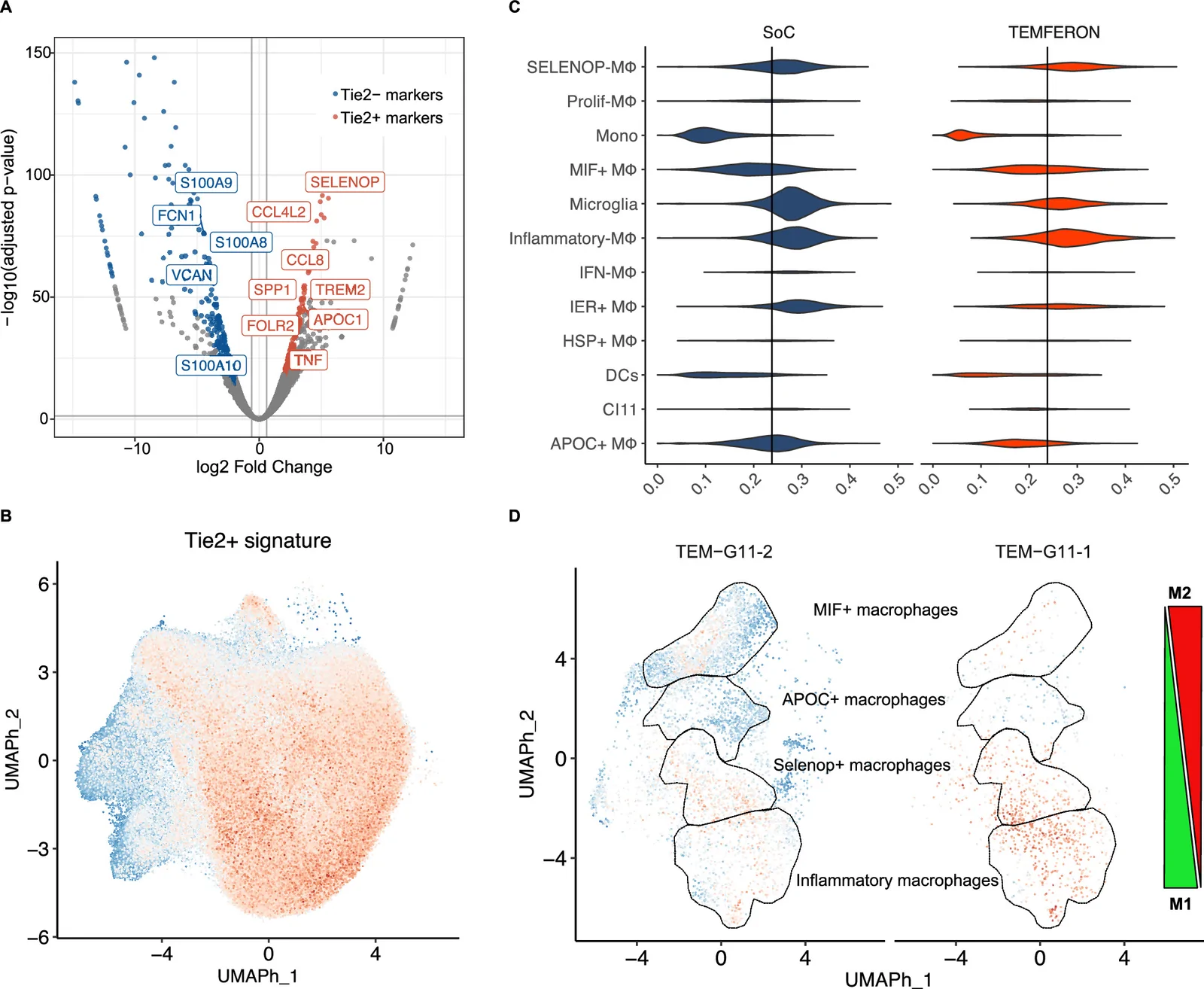
Definition of a human TIE2+ macrophage (TEM) transcriptional program in GBM. (**A**) A freshly resected GBM sample from a control patient was dissociated, enriched for human CD45^+^ cells, and sorted into CD14^+^HLA-DR^+^TIE2^+^ and CD14^+^HLA-DR^+^TIE2- fractions (gating in Extended Data Fig. 6). Volcano plot shows differentially expressed genes between fractions by low-input bulk RNA sequencing; selected genes up- or downregulated in TIE2-expressing macrophages (TEMs) are highlighted. This TEM signature captured a broad representation of genes marking immunosuppressive TAMs. No biological replicates were available, and a biological coefficient of variation (BCV) of 0.1 was assumed to estimate dispersion. EdgeR exactTest (two-sided) for two-group comparisons, p-values were adjusted for multiple testing using the Benjamini–Hochberg false discovery rate. (**B**) A human TEM gene signature derived from bulk RNA-seq was projected onto the myeloid compartment of the extended scRNA-seq dataset. (**C**) Distribution of TEM module scores across myeloid subpopulations is shown for Temferon-treated patients and controls, highlighting a decrease in the APOC^+^ macrophage subset in the Temferon group. (**D**) Projection of the TEM signature onto tumor-infiltrating myeloid cells from a stable (TL1) and a progressing (TL2) lesion in patient TEM-G11. TEM signature–positive cells (red) shift from the SELENOP^+^ macrophage compartment in the progressing lesion to the inflammatory macrophage compartment in the stable lesion (right), compatible with reprogramming towards an M1-like state. On the contrary, the progressing lesion (left) shows an accumulation of TEM signature–negative (blue) APOC^+^ and MIF^+^ M2-like macrophages.

The expression of IRGs was low in the progressing TL2, whereas the stable TL1 showed high IRG levels (Fig. 5c), correlating with an increased proportion of TEMs (16% versus 3%) and higher TIE2 promoter activity in TL1 (Fig. 5d). A TEM gene signature derived from bulk RNA-seq (Extended Data Fig. 9 and Supplementary Data 4) and projected into the scRNA-seq landscape of TL1 and TL2 not only marked a higher proportion of TEMs in TL1, but also showed skewing towards inflammatory TAMs supporting reprogramming of immunosuppressive macrophage states by exogenous TIE2 promoter-driven IFNα (Extended Data Fig. 9d).

DGE analysis between the myeloid TME of TL1 versus TL2 (Supplementary Data 5) showed reduced expression of genes involved in angiogenesis, cell adhesion, extracellular matrix remodeling and tumor progression (MIF, VEGFA, AREG, TIMP1, THBS1) and overexpression of proinflammatory cytokines (TNF, CCL3, CCL4, IL-1B), immediate early response (JUN, FOS) and stress-related genes (several HSPs) (Fig. 5e). Single-cell analysis also confirmed a different composition of the TAM compartment between these two TLs, with an inverted MIF^+^ to inflammatory macrophage ratio (Fig. 5f).

**Extended Data Fig. 10:**
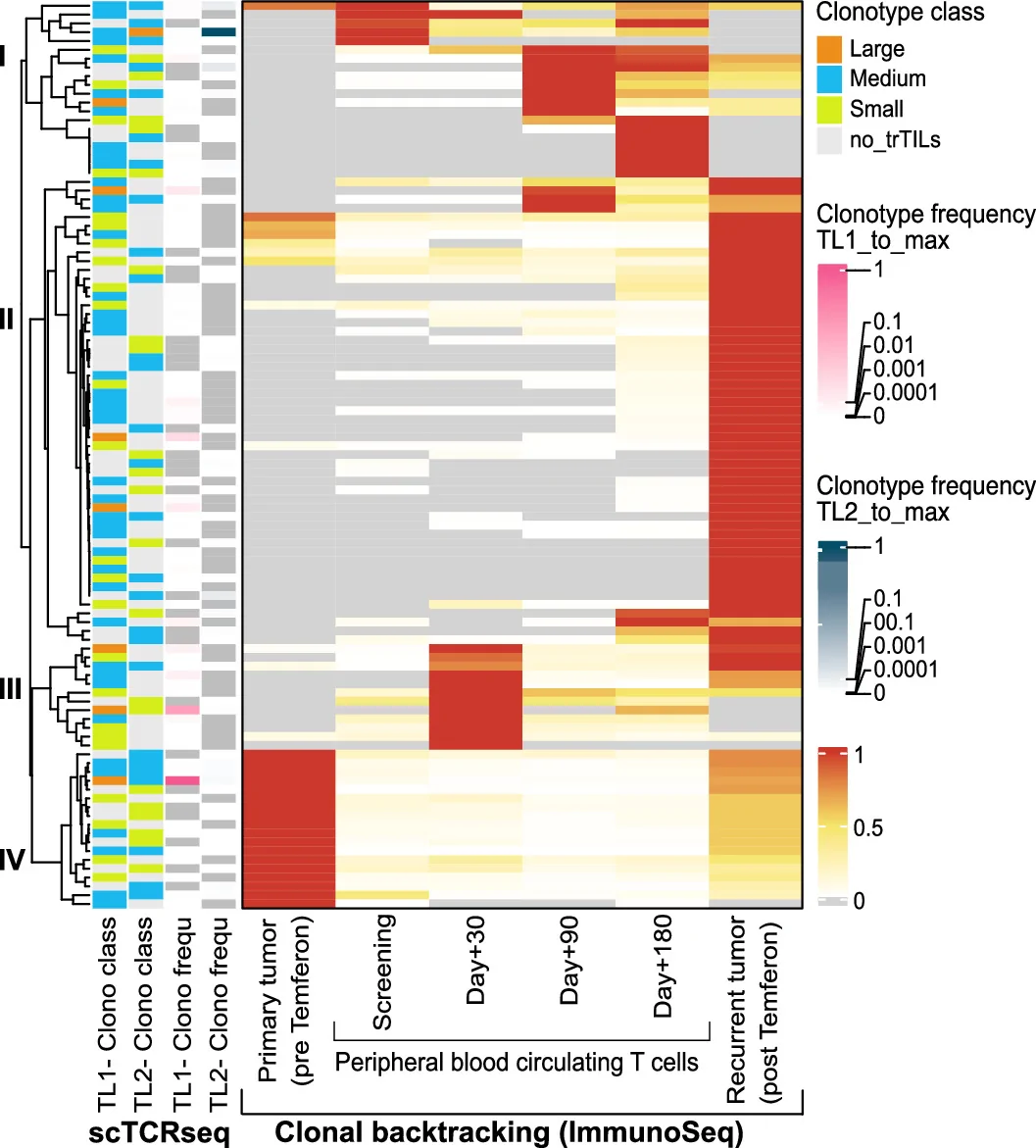
Longitudinal dynamics of predicted tumor-reactive TCR clonotypes. Putative tumor-reactive TILs (trTILs) were defined as T cells with a high NeoTCR8 score by scRNA-seq, a matched TCRβ sequence identified by scTCR-seq, and absent or low representation ( < 0.01%) of the same TCRβ clonotype in peripheral blood scTCR-seq at the time of second surgery. Left columns show the clonotype class (see Fig. 4e for definitions) and relative frequency of individual trTIL clonotypes within the stable (TL1) or progressing (TL2) lesion, as assessed by scTCR-seq. The heatmap (right) tracks these clonotypes in longitudinal bulk TCR sequencing (immunoSEQ) from blood and tumor across therapy time points, including first and second surgeries. Values represent clonotype frequencies scaled to the maximum observed across time points. Unsupervised clustering of bulk TCR data identified four trTIL macro-clusters (I–IV) with distinct blood and tumor dynamics. Expanded clones in the progressing lesion (TL2) were largely bystanders (I), whereas clonotypes enriched in the stable lesion (TL1) segregated into groups characterized by tumor restriction at second surgery (II), transient peripheral expansion at day +30 after conditioning-induced lymphodepletion followed by tumor enrichment (III), or persistence from diagnosis to second surgery (IV). These data indicate preferential expansion and maintenance of tumor-associated TCR clonotypes in stable lesions following Temferon treatment.

In the T cell compartment, the stable TL1 contained a higher proportion of neoTCR8^+^ putative tumor-reactive CD8 T cells (trTILs) (Fig. 5g), which upregulated proinflammatory chemokines, cytotoxic effector genes, HLA class II molecules and IRGs, consistent with sustained local activation (Fig. 5h). T cell receptor clonotype analysis revealed that top clonotypes in TL1 were predominantly tumor-restricted, whereas the larger clonotypes from TL2 were also expanded in blood, suggesting a predominance of bystander T cells (Extended Data Fig. 10). Finally, clonotype-controlled differential expression analysis showed that trTILs in TL1 exhibited a chronically activated state characterized by increases in IFN signaling, heat shock proteins and translational machinery, whereas trTILs in TL2 were enriched for genes associated with less differentiated, non-neoantigen-reactive states (Fig. 5i).

Together, these data link Temferon-induced myeloid reprogramming with the accumulation and activation of tumor-reactive CD8 T cells in a clinically stable lesion, providing granular evidence for coordinated innate and adaptive antitumor immunity within the same patient.

## Discussion

We report a first-in-human clinical trial demonstrating that genetically engineered HSPC can durably generate myeloid progeny that infiltrate the GBM TME, locally release IFNα without inducing systemic toxicity, and reprogram innate and adaptive immune cells toward activation and deployment of antitumor activity. The ability to achieve sustained in vivo production of engineered myeloid cells strongly supports targeting HSPCs rather than mature MoMacs for cytokine-based immunotherapy. With the limitations of a phase 1 study, our clinical results compare favorably with contemporary datasets reporting median OS of ~14 months for *MGMT*^unmeth^ GBM patients treated with SoC^29–31^. A randomized controlled study evaluating the addition of intravenous IFNβ to SoC during radiochemotherapy failed to show a survival benefit, although a trend was visible in younger, fit patients with *MGMT*^unmeth^ or *TP53*^mutant^ GBM^32^. Discontinuous dosing and systemic toxicity probably limited therapeutic efficacy. In contrast, Temferon enables continuous, low-level, tumor-restricted IFNα delivery, which may overcome these limitations by avoiding systemic exposure while sustaining immune activation within the TME.

Local activation of IFN and inflammatory pathways has long been pursued as a strategy to promote antitumor immunity in GBM and other cancers, using approaches including antibody–cytokine fusion proteins^33^, transgenic in vivo delivery^34,35^, STING agonists^36^, epigenetic modifiers^37^, tumor treating fields^38^, radiotherapy^39^ and oncolytic viruses^40,41^. Although several of these strategies activate adaptive immunity, they may also induce counterregulatory mechanisms, such as immune checkpoint upregulation, regulatory T cell expansion^42,43^ and T cell dysfunction. Notably, Temferon treatment was not associated with increased regulatory T cells in blood or tumor, or with widespread induction of immune checkpoint genes, possibly resulting from stable, low-level and local IFN exposure and avoidance of broader pathway activation^44^.

Functional assessment of TILs was limited by the constraints of intracranial tissue sampling. Nonetheless, analysis of recurrent tumor samples provided convergent evidence for enrichment of inflammatory TAMs and engagement and activation of putative tumor-reactive T cells, including by single-cell transcriptomic and bulk and single-cell T cell receptor clonotype profiling. These analyses probably underestimate immune engagement, as samples were obtained at progression, when tumors were escaping immune control, with the notable exception of patient TEM-G11. In this patient, parallel analysis of a stable responding lesion and a progressing lesion revealed a striking shift in the TAM compartment, from immunosuppressive MIF-1^+^ and APOC^+^ subsets toward inflammatory phenotypes. This transition recapitulates the mechanistic findings obtained in an orthotopic GBM mouse model^25^. Conversely, the progressing lesion may indicate exhausting efficacy because of declined levels of transduced monocytes in the blood and/or emergence of resistance mechanisms, including reduced TEM recruitment, diminished TIE2 promoter activity and attenuated response to IFN^45,46^.

Recent work has identified antigen-experienced GZMK^+^ effector T cells in GBM, which were expanded clonally in the TME but not blood^47^. Spatial analyses suggested that these cells are restrained by nearby CD163^+^MHCII^+^ myeloid cells. The observed increase in GZMK^+^ T cells following Temferon treatment supports partial relief of myeloid-mediated suppression. However, the low neoantigen burden of GBM probably limits spontaneous tumor rejection, suggesting that combination therapies might be required^48,49^. CAR-T cell therapies have shown encouraging but transient radiologic responses in GBM, in part due to antigenic heterogeneity^50–52^. Temferon provides a rational partner for such approaches. We have shown in preclinical models that the combination of a strongly tumor-reactive T cell population with a weaker but polyclonal repertoire of endogenous, antitumor T cells is synergistic following Temferon treatment^24^, including in GBM treated with CAR-T against a clinically relevant target^26^. Additional promising combinations include immune checkpoint inhibitors and anti-VEGF agents such as Bevacizumab, which was associated with durable responses in some Temferon patients.

Our data also show that a substantial fraction of tumor-infiltrating hematopoietic cells derive from nonresident populations. Although increased transduction efficiency or optimized radiotherapy could enhance recruitment of engineered cells^53–55^, higher intratumoral frequencies may not be necessary or may trigger toxicities. The HSPC-based platform is adaptable and may support delivery of other therapeutic payloads, including bispecific T cell engagers^56^ or macrophage-activating CARs^57,58^. Emerging chemotherapy-free conditioning regimens^59,60^ may further improve tolerability, particularly in solid tumor settings.

Our work has limitations inherent to phase 1 studies, namely enrollment bias, a small sample size, several cohorts testing several variables (Temferon/supporter cell dose and conditioning regimen), limited post-treatment tissue sampling and the lack of a matched control group to benchmark efficacy.

In conclusion, this interim analysis suggests that Temferon up to a dose of 4 × 10^6^ cells kg^−1^ is safe and feasible as part of a first-line treatment in patients with GBM. Cohort 8 may be extended by up to three additional patients to confirm the recommended phase 2 dose, and an updated analysis will be performed once the last patient has reached 2 years of FU (end of part A of the protocol). These findings warrant further evaluation in a phase 2 study and open the door to a new class of cellular immunotherapies addressing a central mechanism of resistance in solid tumors.

## Methods

### Clinical trial

#### Study design and regulatory oversight

The TEM-GBM_001 trial (EudraCT 2018-001404-11; NCT03866109, preregistered on 5 March 2019) was approved by the Italian Medicines Agency (AIFA) with technical evaluation input from the phase 1 commission of the Italian National Institute of Health (ISS). Ethical approval was obtained from Ethics Committees of San Raffaele Hospital (OSR) and Carlo Besta Neurological Institute (INCB) in Milan. Associated preclinical studies employing mouse models were approved by the Institutional Animal Care and Use Committee and Ethical Board of OSR. The study was conducted in accordance with the principles of Good Clinical Practice and the Declaration of Helsinki. The clinical trial protocol is provided as Supplementary Data 6. The research has included local researchers and clinicians from principal institutions across Italy, which have been included in the author list. The trial was open to participants of any gender involving the local population. Participants received a compensation for incurred travel expenses to attend medical appointments foreseen in the study.

This is a nonrandomized, open-label, multicenter, phase 1/2a, therapeutic-exploratory, dose-escalation, prospective study, involving a single injection of Temferon, an investigational ATMP consisting of autologous CD34^+^-enriched HSPCs transduced with a third generation, vesicular stomatitis virus G (VSV-G) pseudotyped lentiviral vector driving myeloid-specific IFNα expression (TIA126). The vector design has been described by Escobar and colleagues^21^, whereby the clinical construct contained two tandem repeats of microRNA-126 target sequences. The structure and sequence of TIA126 are shown in Supplementary Data 7. The current manuscript describes Part A (Phase 1) of the study, where Temferon was administered, in the period from 2019 to 2024, as part of the first-line treatment to 24 patients affected by GBM who have an unmethylated *MGMT* promoter. Patients were assigned to eight different cohorts, each including three patients and differing in the dose of Temferon and/or the Supporter cells or the conditioning regimen. Cohorts were recruited sequentially following review by an independent DMC, except for cohorts 4 and 5, which recruited in parallel administering the same cell doses after different conditioning regimens. Dose escalation was recommended if no DLT, defined as any adverse event related to Temferon with a CTCAE grade ≧3, was recorded within day +30 from infusion (DLT observation period). Any death attributed to Temferon occurring within day +90 or later represented a criterion to suspend study enrollment and/or obtain ad hoc DMC review. A summary of protocol amendments is provided in Supplementary Table 9 and at the end of the clinical trial protocol (Supplementary Data 6). Genenta Science is the study promoter and financial sponsor and agreed to the submission of the manuscript.

The experimental research readouts and their analysis were performed at the San Raffaele Telethon Institute for Gene Therapy (SR-TIGET). The use of patient data for scientific purposes was authorized through the signing of an informed consent form, in accordance with Article 13 of Legislative Decree No. 196/2003 and the EU General Data Protection Regulation (GDPR). Patients from the Milan control cohort (used exclusively for experimental research readouts conducted under the responsibility of SR-TIGET) have given their informed consent to biobanking and to the research use of excess tumor material deriving from routine procedures and not required for diagnostic purposes (protocol NCH06-2020). Another control cohort (for immunohistochemistry (IHC) evaluations) has been collected as follows: a SoC matched GBM control cohort of newly diagnosed pathologically confirmed GBM and the correspondent recurrent lesions (*n* = 8) were retrieved from the Institutional database of the Department of Pathology (Spedali Civili of Brescia) in compliance with the Declaration of Helsinki and with policies approved by the Ethics Board of Spedali Civili di Brescia, University of Brescia, for retrospective and exclusively observational study on archival material obtained for diagnostic purpose, for which patient consent was not needed (Delibera del Garante no. 52 del 24/7/2008 and DL 193/2003). The control cohort was selected according to the present clinical trial eligibility criteria, including age between 18 and 70 years; histologically confirmed newly diagnosed GBM with unmethylated *MGMT* gene promoter; complete or partial tumor resection; Karnofsky performance score ≥ 70. Information regarding clinical features, treatment and outcome were collected from the medical records. Clinical characteristics are reported in Supplementary Table 8.

#### Patients and procedures

Inclusion criteria were as follows (criteria (1)–(9) at screening; criteria (10) within day −20):

1. Patients aged between 18 years and ≤70 years.
2. Histologically confirmed, newly diagnosed supratentorial GBM with unmethylated *MGMT* gene promoter.
3. Patients have undergone complete or partial tumor resection.
4. Able and willing to provide written informed consent and comply with the study protocol and procedures.
5. Eligible for radiotherapy.
6. Life expectancy of 6 months or more at Screening.
7. Women of childbearing potential enrolled in the study must have a negative pregnancy test at screening and agree to use two distinct acceptable methods of contraception during the trial.
8. Men enrolled in the study with partners who are women of childbearing potential, must be willing to use an acceptable barrier contraceptive method during the trial or have undergone successful vasectomy at least 6 months before entry into the study. Successful vasectomy needs to have been confirmed by semen analysis.
9. Karnofsky performance score ≥ 70.
10. Adequate cardiac, renal, hepatic and pulmonary function (see the Clinical Trial Protocol in Supplementary Data 6 for details).

Exclusion criteria (assessed at screening) were as follows:

(11) Use of other investigational agents or procedures within 4 weeks before study enrolment (within 6 weeks if use of long-acting agents) or participation in a previous gene therapy study.
(12) Known hypersensitivity to carmustine (or any other nitrosurea), thiotepa, busulfan, lenograstim, plerixafor, or any excipients used in these products.
(13) Receipt of any oral or parenteral chemotherapy or immunotherapy within 2 years of screening.
(14) Previous allogeneic BM transplantation, kidney or liver transplant.
(15) Clinical evidence of persistent raised intracranial pressure following surgical resection.
(16) Clinically relevant active viral, bacterial, or fungal infection at eligibility evaluation.
(17) Active autoimmune disease or a relevant history of important autoimmune manifestations, in particular psoriasis, systemic lupus erythematosus, rheumatoid arthritis, vasculitis, immune-mediated peripheral neuropathies.
(18) History of sarcoidosis.
(19) History or current evidence of neuropsychiatric illness including depression, schizophrenia, bipolar disorders, impaired cognitive function, dementia or suicidal tendency.
(20) History of severe cardiovascular disease such as previous stroke, coronary artery disease requiring intervention or unresolved arrhythmias in the past 6 months.
(21) Evidence of any hematological neoplasm.
(22) Positivity for human immunodeficiency virus type 1 or 2 (HIV-1, HIV-2) (serology or RNA), and/or hepatitis B virus surface antigen (HbsAg) and/or hepatitis B virus (HBV) DNA and/or hepatitis C virus (HCV) RNA (or negative HCV RNA but on antiviral treatment) and/or Treponema Pallidum or Mycoplasma active infection.
(23) Active alcohol or substance abuse within 6 months of the study.
(24) Current pregnancy or lactation.
(25) Known bleeding diathesis or history of abnormal bleeding, or any other known coagulation abnormalities that would contraindicate lumbar puncture for CSF or future surgery.
(26) Use of immunosuppressants, with the exception of steroids. The maximum permitted dexamethasone (or equivalent) dose is 4 mg per day.

After informed consent was obtained and inclusion and exclusion criteria were verified, patients underwent leukapheresis on 1 or 2 consecutive days after HSPC mobilization with granulocyte colony-stimulating factor (10 μg per kilogram of bodyweight per day for 3.5–4.5 days) and plerixafor (0.24 mg per kilogram on the days of leukapheresis, ~5 h before the start of the procedure) to reach a drug product dose of at least 0.5–4 × 10^6^ cells kg^−1^ (depending on cohort) and a cryopreserved support or backup in several bags containing a minimum of 4–5 × 10^6^ CD34^+^ cells kg^−1^. CD34^+^-enriched cells were transduced with clinical-grade TIA126 LV and cryopreserved. Patients received conformal focal brain radiotherapy according to SoC. Patients received one of the following conditioning regimens: Cohorts 1–4 and 6: BCNU 400 mg per square meter on day −6, two doses (12 h apart) of thiotepa 5 mg kg^−1^ on day −5. Cohort 5: thiotepa 5 mg kg^−1^ on days −5 and −4, busulfan 3.2 mg kg^−1^ on day -4, with additional pharmokinetic-guided doses on day −3 and, potentially, day −2 towards an area under the curve target of between 40 and 60 mg × h l^−1^. Cohorts 7 and 8: busulfan 3.2 mg kg^−1^ on day −4, with additional pharmokinetic-guided doses on day −3 and, potentially, day −2 towards an area under the curve target of 60 mg × h l^−1^. On day 0, patients received first the infusion of supporter cells and, at least 3 h thereafter, the foreseen dose of Temferon (rounded to the nearest aliquot size, with a maximum tolerance of +10%), by intravenous infusion, preferably through a central venous catheter. Temferon dose adjustments were planned based on the VCN measured in the product, as follows: VCN < 0.4: 0.8 × 10^6^ cells kg^−1^ (cohort 1), 1.5 × 10^6^ cells kg^−1^ (cohort 2), 3.0 × 10^6^ cells kg^−1^ (cohorts 3, 4 and 5), ≥3.0 × 10^6^ cells kg^−1^ (cohort 6, 7), ≥4.0 × 10^6^ cells kg^−1^ (cohort 8); VCN 1.1–1.5: 0.4 × 10^6^ cells kg^−1^ (cohort 1), 0.8 × 10^6^ cells kg^−1^ (cohort 2), 1.5 × 10^6^ cells kg^−1^ (cohorts 3, 4 and 5), 2.3 × 10^6^ cells kg^−1^ (cohorts 6 and 7), 3.0 × 10^6^ cells kg^−1^ (cohort 8); VCN > 1.5: 0.3 × 10^6^ cells kg^−1^ (cohort 1), 0.5 × 10^6^ cells kg^−1^ (cohort 2), 1.0 × 10^6^ cells kg^−1^ (cohorts 3, 4 and 5), 1.5 × 10^6^ cells kg^−1^ (cohorts 6 and 7), 2.0 × 10^6^ cells kg^−1^ (cohort 8). In cohorts 4 and 5, if necessary, the supporter cell dose was adjusted to ensure the infusion of a minimum total of 4.0 × 10^6^ CD34^+^ cells kg^−1^.

During hospitalization, patients remained in an isolation unit and received supportive therapy according to local standards.

#### Endpoints

The primary endpoint was tolerability and safety of Temferon over the first 90 days following Temferon administration. Secondary endpoints included long-term tolerability and safety up to 2 years following Temferon administration, dose and conditioning regimen selection, Temferon engraftment, clinical response, quality of life and survival.

Tolerability and safety were evaluated through routine clinical and laboratory surveillance according to CTCAE v.5.0 criteria, with particular attention to significant organ toxicities (grade 3 or higher) attributable to Temferon or the harvesting of HSPCs, as well as autoimmune manifestations. A maximally tolerated dose was defined by the presence of a grade 3–5 adverse event that occurred within the first 30 days and was attributed to Temferon. Other secondary safety endpoints included the proportion of patients achieving hematologic recovery of neutrophil and platelet counts by day +30, defined as the first of 3 consecutive days of absolute neutrophil count (ANC) > 0.5 × 10^9^ l^−1^ and platelet count (PLT) > 20 × 10^9^ l^−1^, in the absence of transfusion for at least 7 consecutive days, and the tolerability and safety of the three different conditioning regimens, up to day +45 after Temferon administration.

Best response and disease progression were defined according to iRANO criteria.

The presence of transduced myeloid cells in the blood and BM was determined by measuring the lentiviral VCN on genomic DNA extracted from mononuclear cells (Ficoll) or magnetic-bead purified subpopulations (MACS microbeads, Miltenyi Biotec) using a validated digital droplet PCR (ddPCR) assay amplifying a vector-specific sequence (HIV) (Cat. no. 4332079 Custom TaqMan, Thermo Fisher Scientific) and a reference sequence within the TATA-Box Binding Protein Associated Factor 7 (TAF7) gene (cat. no. 4448491 TaqMan, Thermo Fisher Scientific) on the Bio-Rad QX200 system.

Transduction efficiency was measured in clonogenic progenitors by plating the drug product or the patient’s BM enriched for CD34^+^ cells in complete Methocult medium (CFU assay, Stem Cell Technologies). Single colonies (on average, 35 colony-forming unit–granulocyte–macrophage (CFU-GM) and 47 burst-forming unit-erythroid (BFU-E) per patient and timepoint) were picked, lysed and subjected to the above-described ddPCRs. Colonies with a VCN < 0.5 were considered nontransduced. Engraftment of transduced progenitors was calculated as the proportion of CFU-GM and BFU-E with a VCN ≥ 0.5 over the total number of colonies where the TAF7 signal was within the range of acceptance.

IFNα levels in the plasma and in CSF were measured by enzyme-linked immunosorbent assay (ELISA) using the Verikine-HS Human IFNα all subtype ELISA kit (cat. no. 41115, PBL Assay Science), according to the manufacturer’s instructions. IFN gene signatures were measured on RNA extracted from blood, BM subpopulations or tumor tissue using ddPCR assays for OAS1 (Hs00973637_m1), IFIT1 (Hs00356631_g1), IRF7 (Hs01014809_g1) and the housekeeping gene HPRT1 (Hs02800695_m1) (Thermo Fisher Scientific).

Lentiviral integration site retrieval was performed on genomic DNA using a sonication-based linker-mediated-PCR method, as described in refs. ^61–65^. Additional details can be found in Supplementary Methods.

The abovementioned nonroutine specialty tests were performed in the TIGET Clinical Laboratory and the Vector Integration Core, certified for phase 1 studies by the competent authorities.

#### Statistics and reproducibility

Patients were enrolled consecutively in cohorts of three at a time at a given dose level and conditioning regimen, with rule-based dose escalation (absence of DLTs in all three patients). No statistical method was used to predetermine sample size. The planned interim analysis (last patient from Part A reaching day +30 FU) reported here is descriptive, and no formal statistical testing was conducted. No randomization was performed. The investigators were not blinded to cohort allocation during outcome assessment. Individual values are shown, where appropriate. Where medians are shown, a minimum *n* = 3 was required for each individual datapoint. Unless otherwise indicated, data from the research endpoints are presented as mean and s.d. or median and range, as appropriate. See figure legends for details. No data were excluded from the analyses.

### Experimental study endpoints

#### Bulk TCR-seq

Human TCR characterization was performed using ImmunoSEQ (Adaptive Biotechnology). Exploratory analyses of T cell receptor β diversity by sequencing of the T cell receptor β locus were performed on genomic DNA extracted from tumor tissue and blood samples.

#### Tumor processing

Formalin-fixed, paraffin-embedded (FFPE) tumor blocks, prepared as per SoC from tumor material at primary or second surgery, were stored in the pathology departments of the referring centers. Sections from FFPE tumor material were used for DNA-based analyses, as follows: bulk TCR-seq analysis, performed by Adaptive biotechnologies (immunoSEQ); bulk VCN assessment by ddPCR, as described above, following DNA extraction with the QIAmp DNA FFPE Tissue kit (Qiagen). Expression of IFN response signature genes (data presented in Fig. 3e) was performed by Cell Carta (ex-Biogazelle) on tumor RNA, extracted with the miRNeasy FFPE Kit (Qiagen), using quantitative real-time PCR (qPCR). In brief, qPCR was performed in two replicates (5 μl reactions in 384-well format (cat. no. CFX384, Bio-Rad)) on the GAPDH, HMBS and TBP reference genes and on IFNA2, IRF7, OAS1 and IFIT1 target genes. Alternatively, IFN-response genes were assessed using the ddPCR assays described above (some of the data presented in Extended Data Fig. 4).

Fresh tumor material was obtained from some Temferon patients or other control patients treated according to SoC when undergoing second surgery for progressive disease. Part of the material was snap-frozen for DNA and RNA extraction followed by VCN analysis and IFN-response signature gene expression by ddPCR, respectively, as described above. Another part of the fresh tumor was dissociated using the ‘Tumor Dissociation Kit’ (Miltenyi Biotec), lysed to eliminate red blood cells with Ammonium Chloride Solution Lysis Buffer (ACK) (Stem Cell Technologies), purified using ‘Debris removal kit’ (Miltenyi Biotec) and ‘Dead cell removal kit’ (Miltenyi Biotec), and separated into a CD45^+^ and CD45^−^ fraction by human CD45 (TIL) MicroBeads (Miltenyi Biotec). Both fractions were used, either fresh or frozen, for 10× scRNA-seq (see below). Whenever enough material was available, DNA and RNA were extracted from the residual CD45^+^ fraction for VCN analysis and IFN-response signature gene expression (by ddPCR) on the purified hematopoietic tumor infiltrate.

#### FACS analysis and sorting for TEMs

Single-cell suspensions of CD45-enriched tumor fractions were resuspended in PBS and stained with ‘live and dead blue’ (Thermo Fisher) for 30 min. Cells were then washed and resuspended in MACS staining buffer (Miltenyi Biotec) at a concentration of ~10^6^–10^7^ cells ml^−1^. Staining with the following monoclonal antibodies was performed at 4 °C for 15–20 min:

CD14 (APC-H7) 1:100 (BD Biosciences, cat. no. 560180), HLA-DR (APC) 5:100 (BD Biosciences, cat. no. 559866), CD3 (PE-Cy7) 3:100 (BioLegend, cat. no. 300316), CD83 (BV421) 1:100 (BD Biosciences, cat. no. 562630), TEK (TIE2) (PE) 10:100 (R&D Systems, cat. no. FAB3131P), Isotype control (PE) 10:100 (R&D Systems, cat. no. IC002P).

Samples were acquired on an LSRII or Canto cytometer, or an FACS Aria Fusion Sorter equipped with Diva Software (all from Becton Dickinson), and data were analyzed with FCS Express (De Novo Software) following manual compensation.

#### Neuropathological analysis of human tumor cohorts

Histological diagnosis was reviewed according to the World Health Organization 2021 classification of CNS tumors and paraffin-embedded representative tissue sections were selected based on hematoxylin and eosin-stain confirmed tissue preservation. Immunohistochemical staining was performed as described previously^66^.

Expression of CD34 was assayed using an anti-CD34 antibody (1:50; Miltenyi, cat. no. 130-134-763 or Leica cat. no. END-L-CE) and detected using Dako EnVision+ Dual Link System Peroxidase (Dako Cytomation) followed by diaminobenzidine and hematoxylin counterstaining. Images were acquired with an Olympus BX60 microscope equipped with a DP70 camera and CellF imaging software (Soft Imaging System GmbH). Vessel distribution and density in viable tumor regions were scored semiquantitatively based on vessel size, presence of microvascular proliferation and CD34 staining intensity (0–3). Quantitative analysis was performed using Arivis Vision4D software (Zeiss). Regions of interest were delineated manually to define viable tumor areas. Diaminobenzidine signal (brown) was extracted by color deconvolution. For CD34-positive staining, a local adaptive threshold algorithm was applied. The percentage of CD34^+^ area per region of interest was calculated. All samples were processed using identical segmentation parameters.

Custom RNAscope LS Multiplex ISH/IF assay for Hs-TEK mRNA and CD14 protein codetection was performed on a Leica BOND RX platform using the RNAscope LS Multiplex Fluorescent kit (Advanced Cell Diagnostics, cat. no. 322800). 5 µm FFPE sections were pretreated (heat, protease) and hybridized with target probes. Signal was amplified and detected using TSA-fluorophore chemistry. Each sample was quality controlled for RNA integrity with PPIB/POLR2A probes and for background with bacteria-specific dapB probe. Signals were detected as punctate Opal650 fluorescence. For CD14 protein detection, anti-CD14 (Abcam EPR3653, cat. no. ab133335) with Alexa Fluor 594 secondary antibody (Goat Anti-Rabbit IgG H&L, Abcam, cat. no. ab150084) was used. Samples were counterstained with 4′,6-diamidino-2-phenylindole. Fluorescent images were acquired using a 3Dhistech Pannoramic SCAN II (×40 objective).

Combined IHC staining for CD14 and TEK was performed after completing the RNAscope for TEK detection by Advanced Cell Diagnostics (see above) with RNAscope 2.5 LS Reagent Kit – RED (cat. no. 322150). CD14 expression was detected using anti-CD14 (1:50; Miltenyi cat. no. 130-110-578) with MACH4 AP Polymer (Biocare) and Vector Blue substrate.

#### Single-cell library preparation and sequencing

Single-cell RNA and V(D)J libraries were prepared with the Chromium Next GEM Single-Cell 5′ and V(D)J Reagents Kits v.1.1 (10x Genomics). After CD45 selection, ≥900 cells were loaded according to the manufacturer instructions. 5′ gene expression library construction and sequencing were performed on Illumina NextSeq or NovaSeq S1 platforms.

#### scRNA-seq data processing

Sequenced libraries were processed by Cell Ranger (v.6.1.1) to generate unique molecular identifier (UMI) count matrices and V(D)J annotations. GRCh38 references were used. Data were analyzed in R (v.4.1.3) using several R and Bioconductor packages.

Preprocessing included removal of ambient RNA, doublets and cross-contaminating cells. Ambient RNA was removed using SoupX; Doublets were identified using scds (doublet score > outlier threshold: 75th quantile + 1.5 × interquartile range) and scDblFinder (built-in algorithm); cells flagged by either method (union) were removed. Identification of CD45^−^ and CD45^+^ fractions, and myeloid and T cell compartments within the CD45^+^ fraction, was performed (with removal of cells) where assignment to one of these compartments was ambiguous.

Seurat objects (v.4.1.1) were generated for CD45^+^ and CD45^−^ datasets applying custom parameterization strategies tailored to the specific dataset. Analyses were performed using Seurat workflows. Data were log-normalized (scale factor 10,000), variable genes selected (top 15%), scaled and corrected for mitochondrial content, UMI counts and cell cycle scores.

Dimensionality reduction was performed using principal component analysis (50–100 principal components; elbow selection), followed by Harmony batch correction (DonorID and sample). Clustering used the Louvain algorithm across several resolutions. Marker genes for each cluster and differential expression between conditions within clusters or cells of interest were computed using Seurat’s FindAllMarkers and FindMarkers functions, respectively.

#### Cell-type annotation and compartment definitions

Cell-type annotation was performed using SingleR (v.1.8.1) and manual curation based on canonical markers and reference datasets.

CD45^+^ cells were filtered (400–5,000 genes; <15% mitochondrial reads) and partitioned into myeloid, T and B cell compartments based on CD14, CD3 and CD79A expression.

Clusters were classified as myeloid, T, B or contaminant. Doublets and discordant annotations were removed. After filtering, 37,382 T or natural killer (NK) cells, 59,083 myeloid cells and 641 B cells were identified; B cells were excluded from downstream analyses due to their low abundance.

For manual annotation, clusters of the CD45^−^ tumor and nonhematopoietic stroma compartment were annotated using the Gbmap-core atlas^67^ -v.4 annotation label as a basis. Myeloid and T cell compartments were annotated manually based on optimal clustering resolution and top marker gene inspection. Details are provided in Supplementary Methods and the GitLab repository.

#### scTCR-seq data analysis

V(D)J data were processed using scRepertoire. Cells with valid T cell receptor β sequences were retained (~65%). Clonotypes were assessed according to the amino acid sequence of the T cell receptor β-chain only (cdr3_aa2 variable), due to high dropout of the T cell receptor α-chain. Clonotype frequencies were calculated by grouping for patients. Clonotype expansion classes were defined by percentages rather than absolute number. Frequency classes: hyperexpanded (>10%), large (1–10%), medium (0.1–1%) and small (<0.1%).

#### Tumor reactivity evaluation

Tumor reactivity was assessed using the NeoTCR8_ALL signature^28^ (UCell v.2.0.1). To define tumor-reactive TILs (trTILs), we performed *k*-means (*n* = 4) clustering to define a threshold (0.235). Cells above threshold were defined as tumor-reactive. Analyses focused on CD8 subsets, considering either cells harboring expanded clonotypes (frequency > 0.1%) or all cells. Alluvial plots to relate trTILs with single patients, CD8 TIL subcluster and clonotype expansion class were generated using the ggalluvial package.

#### Extended dataset: integration with Gbmap-core atlas

Our fully filtered internal dataset (CD45^+^ and CD45^−^ fractions) was integrated with the Gbmap-core atlas^67^. Gene IDs were standardized and shared genes retained. Data were filtered (300–6,000 expressed genes, 500–400,000 UMI counts; top 3,766 most variable genes selected, excluding T cell receptor genes), batch-corrected (Harmony) and clustered (resolution 1.2), and split into myeloid, T/NK and tumor compartments, which were reanalyzed independently. Cells from the Darmanis2017 and Yu2020 subdatasets were excluded due to the low number of cells. Ex novo re-analysis of the myeloid, T/NK and tumor compartments followed the same basic data analysis workflow used for the internal dataset. We chose a simplified resolution annotating each cluster by curated manual inspection of top markers using population labels defined in the internal dataset as drivers. DGE analysis between the TEMFERON group and Others was performed using the FindMarkers function To allow downstream analysis, such as GSEA, we tuned the function parameters (logFC = 0, min.pct = 0.1) to give the highest reliable number of genes, ensuring a minimal fraction of expression (10%) of the gene in one of the two groups.

#### Matched control dataset

Inclusion criteria were the following: scRNA-seq data acquired on the 10x Genomics platform, patients with *MGMT*^unmeth^ *IDH*^WT^ GBM undergoing second surgery for recurrent disease, who have not received previous immunotherapies (SoC control group). In addition to the three patients from our internal dataset (Milano; Supplementary Table 8) and selected cases from the extended dataset (Brussels^10^; BWH^68^) who satisfied these inclusion criteria, we identified other cases from publicly available data from the recent literature: McGill^69^, UCLA^70^ and BJH^47^. The data were processed starting from raw counts performing cluster annotation and identification of myeloid or T cells (McGill), or processed Seurat objects selecting the T cell containing clusters (UCLA, cluster 4 and BJH). Minimal Seurat objects from each public dataset with selected cells (myeloid or T cells) were produced encompassing a set of genes common to all datasets integrating eligible cases from the extended dataset (Milano, Brussels and BWH control cohorts). To annotate clusters that resemble the labels used in the extended dataset, we leveraged the labeling from already annotated cells and evaluated the frequency distribution of cell-type labels within each identified cluster (at resolutions 1.8 for T cells and 0.6 − subcluster for the Myeloid compartment). For the latter, we performed subclustering of cluster 0 and cluster 1 using default resolution, identifying four different cluster 0 subclusters (0_1 to 0_4) and four different cluster 1 subclusters (1_0 to 1_4). Closer investigation of the CD8 ZNF683^+^ cluster (defined in the extended dataset) pointed to a terminally differentiated effector CD8 state (CD8tdEff) and, as the ZNF683 marker gene was not found in samples from immunotherapy-naive patients of the matched control dataset, this CD8 ZNF683^+^ cluster was renamed as CD8tdEff T cells. Annotation was checked performing custom manual marker inspection obtained with the FindAllMarkers Seurat function, setting cell-type label as reference identity. Wilcoxon rank sum test was used to assess differences in frequency distribution between Temferon and the SoC group (for each cell type). To evaluate the global effect of Temferon in the myeloid compartment, we performed a DGE analysis following a bulk approach using the Model-based Analysis of Single-cell Transcriptomics (MAST) algorithm to compare Temferon versus SoC controlling for cell-type variable.

#### Downstream analysis: GSEA and ligand–receptor analyses

GSEA was performed using the GSEA function from the clusterProfiler package (v.1.18) starting from the preranked (logFC sorted) geneset lists obtained from the comparison of Temferon versus controls in each cell population. We relied primarily on the MSigDB hallmark geneset (v.7.4). Terms with a *P*_val.adj_ < 0.01 were considered significantly enriched. To evaluate the interplay between myeloid and T cells within the TME, we established a data analysis workflow based on the nichenet (v.2.0.4) R package. We evaluated cell-to-cell communication between several sets of myeloid and T cell populations, focusing primarily on the study of the interplay between all myeloid populations (defined as senders) and the CD8 GZMK^+^ Eff/Exh T cells (defined as the recipient population). For the identification of top ligands that differently (positively) modulated target genes in TEMFERON compared to the Others (control) group, we relied on the nichenet_seuratobj_aggregate function setting expression_pct (0.2), lfc_cutoff (0.25), top_n_ligands (30) and geneset = ‘up’. As ligand target matrix, ligand–receptor network and weighted networks, we relied on the ones provided by the software (available via Zenodo at https://doi.org/10.5281/zenodo.7074290 (ref. ^71^).

#### Transcriptomic characterization of trTILs in TEM-G11 lesions

For patient TEM-G11, scTCR-seq was also performed on PB samples drawn around the time of second surgery. We leveraged clonotypes detected at high frequency in PB to filter out putative bystander TILs detected in the second surgery clonotype analysis. The assumption is that a tumor-reactive TIL should be a nonexpanded clone in the blood but is prone to expand in the second surgery tumor. Hence, to focus on the characterization of putative tumor-reactive TILs, clonotypes defined as medium, large or hyperexpanded in PB were discarded from the TEM-G11 (both lesions) second surgery clonotypes set. We retained from PB samples only shared clonotypes with a frequency of less than 0.1% in PB samples, thereby including potential tumor expanded clonotypes circulating at low frequency in the blood. We subset the T cells from the internal dataset focusing only on cells that passed the above-described filter and belonged to the CD8 T cell population excluding the cluster encompassing CD4/CD8 naive T cells. Moreover, we discarded cells lacking a T cell receptor β clonotype (mostly NK cells). On this dataset, we evaluated both the transcriptional difference between putative trTILs and byStander cells within the TEM-G11-1 sample and the differences between trTILs from TL1 and TL2. For differential expression, we employed the MAST algorithm using as covariates the cell population and the clonotype (aa2), respectively, to mitigate cell of origin biases.

#### Clonotype backtracking across therapy timepoints

Clonal dynamics of tumor clonotypes identified by scTCR-seq at second surgery were tracked across clinical timepoints (tumor: first and second surgeries; PB: screening, baseline and days 30, 90 and 180) using bulk T cell receptor β sequencing (ImmunoSeq). Bulk clonotype frequencies (amino acid level) were integrated into scRNA-seq metadata using T cell receptor β as a key.

Analyses were restricted to CD8 T cell clonotypes. Clonal trajectories were inferred by unsupervised clustering of frequency matrices across timepoints for each patient. The same approach was applied to compare clonotype dynamics between paired lesions.

For lesion-specific analyses, putative trTILs were subsetted and clonotypes supported by a single cell were excluded. T cell receptor β frequencies were computed per lesion and normalized by maximum-frequency scaling across timepoints (first to second surgery).

#### Bulk RNA-seq analysis: Tie2 signature identification and mapping

Bulk RNA-seq was performed on the NextSeq platform (Illumina) after full length cDNA amplification using the SMART-Seq mRNA LP kit (Takara). Raw data were processed and trimmed using TrimGalore (v.0.5.0) and aligned to the GRCh38 reference using STAR (v.2.7.0d) with standard settings. For the TIE2^high^ versus TIE2^−^ comparison (only a single replicate available), we set up a biological variation coefficient of 0.1 to simulate gene expression dispersion. Gene counts were then normalized using the trimmed mean of M-values method and differential test was performed with the exactTest function provided by the edgeR package (v.3.32). Genes with an adjusted *P* value (Benjamini–Hochberg false discovery rate (FDR) method) < 0.05 were considered differentially expressed. To minimize false positive differentially expressed genes (DEGs) and to apply the signature to the single-cell data, we applied a filter on absolute gene expression, in terms of counts per million and magnitude of expression change, in terms of log fold change (FC) (logFC). We defined the TIE2^high^ signatures as genes with logFC > 2 and logCPM > 5, resulting in a 105 gene signature.

### Reporting summary

Further information on research design is available in the Nature Portfolio Reporting Summary linked to this article.

## Online content

Any methods, additional references, Nature Portfolio reporting summaries, source data, extended data, supplementary information, acknowledgements, peer review information; details of author contributions and competing interests; and statements of data and code availability are available at https://doi.org/10.1038/s41591-026-04419-1.

## Supplementary information

Supplementary Information Supplementary Tables 1–8 and Figs. 1–4.

Reporting Summary

Supplementary Data scRNA-seq of six Temferon and six internal control samples from recurrent GBM (internal dataset).

Supplementary Data Integrated scRNA-seq data characterizing GBM tumors (extended dataset).

Supplementary Data Integrated scRNA-seq data from Temferon samples and matched controls (recurrent GBM dataset).

Supplementary Data TIE2^+^ TAM signature.

Supplementary Data DEGs in patient TEM-G11, TL1 and TL2.

Supplementary Data Clinical trial protocol with amendment history.

Supplementary Data Structure and sequence of TIA126 lentiviral provirus.

## Data Availability

Raw fastq data as well as processed data including count and contig matrices of single-cell datasets were deposited in the ArrayExpress repository with the following IDs: E-MTAB-15962 (bulkRNAseq TIE2+vsTIE2-TAMs) and E-MTAB-16504 (scRNA-seq). All data and knowledge generated from the TEM-GBM_001 study refer to anonymized, individual-level clinical data, including but not limited to patient demographics, safety and efficacy outcomes, and associated clinical documentation. These data are the property of Genenta Science, which agrees to make them publicly available in accordance with applicable laws and regulations. Genenta Science is committed to sharing access to anonymized patient-level data and supporting clinical documents from eligible studies with qualified external researchers. Data will be made available following the execution of a Data Usage Agreement. Investigators and institutions who agree to the terms of the Data Usage Agreement—including the use of data solely for a defined research purpose, for noncommercial scientific use, and with strict measures to ensure confidentiality and prevent any reidentification of participants—will be granted access. Specific requests should be forwarded to info-trial@genenta.com. Genenta Science will review all requests and provide a response within 20 business days.

## Code availability

Code and workflow for producing plots and tables are stored in the gitlab repository as file or url link, available via http://www.bioinfotiget.it/gitlab/custom/gentner_temgbm_2025.

## References

[1] Cassetta, L. & Pollard, J. W. A timeline of tumour-associated macrophage biology. *Nat. Rev. Cancer* **23**, 238–257 (2023). 10.1038/s41568-022-00547-1 36792751

[2] Kloosterman, D. J. & Akkari, L. Macrophages at the interface of the co-evolving cancer ecosystem. *Cell* **186**, 1627–1651 (2023). 10.1016/j.cell.2023.02.020 36924769

[3] Mantovani, A., Allavena, P., Marchesi, F. & Garlanda, C. Macrophages as tools and targets in cancer therapy. *Nat. Rev. Drug Discov.* **21**, 799–820 (2022). 10.1038/s41573-022-00520-5 PMC9380983 35974096

[4] Barry, S. T., Gabrilovich, D. I., Sansom, O. J., Campbell, A. D. & Morton, J. P. Therapeutic targeting of tumour myeloid cells. *Nat. Rev. Cancer.* **23**, 216–237 (2023). 10.1038/s41568-022-00546-2 36747021

[5] Reiss, K. A. et al. CAR-macrophage therapy for HER2-overexpressing advanced solid tumors: a phase 1 trial. *Nat. Med.* **31**, 1171–1182 (2025). 10.1038/s41591-025-03495-z 39920391

[6] Sloas, C., Gill, S. & Klichinsky, M. Engineered CAR-macrophages as adoptive immunotherapies for solid tumors. *Front. Immunol.* **12**, 783305 (2021). 10.3389/fimmu.2021.783305 PMC8652144 34899748

[7] Gentner et al. Hematopoietic stem-and progenitor-cell gene therapy for Hurler syndrome. *N. Engl. J. Med.* **385**, 1929–1940 (2021). 10.1056/NEJMoa2106596 34788506

[8] Sailor, K. A. et al. Hematopoietic stem cell transplantation chemotherapy causes microglia senescence and peripheral macrophage engraftment in the brain. *Nat. Med.* **28**, 517–527 (2022). 10.1038/s41591-022-01691-9 35190726

[9] Akkari, L. et al. Dynamic changes in glioma macrophage populations after radiotherapy reveal CSF-1R inhibition as a strategy to overcome resistance. *Sci. Transl. Med.* **12**, eaaw7843 (2020). 10.1126/scitranslmed.aaw7843 32669424

[10] Pombo Antunes, A. R. et al. Single-cell profiling of myeloid cells in glioblastoma across species and disease stage reveals macrophage competition and specialization. *Nat. Neurosci.* **24**, 595–610 (2021). 10.1038/s41593-020-00789-y 33782623

[11] Casanova-Acebes, M. et al. Tissue-resident macrophages provide a pro-tumorigenic niche to early NSCLC cells. *Nature* **595**, 578–584 (2021). 10.1038/s41586-021-03651-8 PMC8923521 34135508

[12] Tucci, F., Galimberti, S., Naldini, L., Valsecchi, M. G. & Aiuti, A. A systematic review and meta-analysis of gene therapy with hematopoietic stem and progenitor cells for monogenic disorders. *Nat. Commun.* **13**, 1315 (2022). 10.1038/s41467-022-28762-2 PMC8921234 35288539

[13] Fumagalli, F. et al. Lentiviral haematopoietic stem-cell gene therapy for early-onset metachromatic leukodystrophy: long-term results from a non-randomised, open-label, phase 1/2 trial and expanded access. *Lancet* **399**, 372–383 (2022). 10.1016/S0140-6736(21)02017-1 PMC8795071 35065785

[14] Stupp, R. et al. Radiotherapy plus concomitant and adjuvant temozolomide for glioblastoma. *N. Engl. J. Med.* **352**, 987–996 (2005). 10.1056/NEJMoa043330 15758009

[15] De Groot, J. et al. Window-of-opportunity clinical trial of pembrolizumab in patients with recurrent glioblastoma reveals predominance of immune-suppressive macrophages. *Neuro. Oncol.* **22**, 539–549 (2020). 10.1093/neuonc/noz185 PMC7158647 31755915

[16] Reardon, D. A. et al. Effect of Nivolumab vs Bevacizumab in Patients with Recurrent Glioblastoma: The CheckMate 143 Phase 3 Randomized Clinical Trial. *JAMA Oncol* **6**, 1003–1010 (2020). 10.1001/jamaoncol.2020.1024 PMC7243167 32437507

[17] De Palma, M. et al. Tie2 identifies a hematopoietic lineage of proangiogenic monocytes required for tumor vessel formation and a mesenchymal population of pericyte progenitors. *Cancer Cell* **8**, 211–226 (2005). 10.1016/j.ccr.2005.08.002 16169466

[18] Venneri, M. A. et al. Identification of proangiogenic TIE2-expressing monocytes (TEMs) in human peripheral blood and cancer. *Blood* **109**, 5276–5285 (2007). 10.1182/blood-2006-10-053504 17327411

[19] Mazzieri, R. et al. Targeting the ANG2/TIE2 axis inhibits tumor growth and metastasis by impairing angiogenesis and disabling rebounds of proangiogenic myeloid cells. *Cancer Cell* **19**, 512–526 (2011). 10.1016/j.ccr.2011.02.005 21481792

[20] Gentner, B. et al. Identification of hematopoietic stem cell-specific miRNAs enables gene therapy of globoid cell leukodystrophy. *Sci. Transl. Med.* **2**, 58ra84 (2010). 10.1126/scitranslmed.3001522 21084719

[21] Escobar, G. et al. Genetic engineering of hematopoiesis for targeted IFN-α delivery inhibits breast cancer progression. *Sci. Transl. Med.* **6**, 217–3 (2014). 10.1126/scitranslmed.3006353 24382895

[22] De Palma, M. et al. Tumor-targeted interferon-α delivery by Tie2-expressing monocytes inhibits tumor growth and metastasis. *Cancer Cell* **14**, 299–311 (2008). 10.1016/j.ccr.2008.09.004 18835032

[23] Catarinella, M. et al. IFN α gene/cell therapy curbs colorectal cancer colonization of the liver by acting on the hepatic microenvironment. *EMBO Mol. Med.* **8**, 155–170 (2016). 10.15252/emmm.201505395 PMC4734840 26769348

[24] Escobar, G. et al. Interferon gene therapy reprograms the leukemia microenvironment inducing protective immunity to multiple tumor antigens. *Nat. Commun.* **9**, 2896 (2018). 10.1038/s41467-018-05315-0 PMC6057972 30042420

[25] Birocchi, F. et al. Targeted inducible delivery of immunoactivating cytokines reprograms glioblastoma microenvironment and inhibits growth in mouse models. *Sci. Transl. Med.* **14**, eabl4106–eabl4106 (2022). 10.1126/scitranslmed.abl4106 35857642

[26] Rossari, F. et al. A cross-talk established by tumor-targeted cytokines rescues CAR T cell activity and engages host T cells against glioblastoma in mice. *Sci. Transl. Med.* **17**, eado9511 (2025). 10.1126/scitranslmed.ado9511 40601779

[27] Sessa, M. et al. Lentiviral haemopoietic stem-cell gene therapy in early-onset metachromatic leukodystrophy: an ad-hoc analysis of a non-randomised, open-label, phase 1/2 trial. *Lancet* **388**, 476–487 (2016). 10.1016/S0140-6736(16)30374-9 27289174

[28] Lowery, F. J. et al. Molecular signatures of antitumor neoantigen-reactive T cells from metastatic human cancers. *Science* **375**, 877–884 (2022). 10.1126/science.abl5447 PMC8996692 35113651

[29] Alnahhas, I. et al. Characterizing benefit from temozolomide in MGMT promoter unmethylated and methylated glioblastoma: a systematic review and meta-analysis. *Neurooncol. Adv.* **2**, vdaa082 (2020). 10.1093/noajnl/vdaa082 PMC7596890 33150334

[30] Omuro, A. et al. Radiotherapy combined with nivolumab or temozolomide for newly diagnosed glioblastoma with unmethylated MGMT promoter: An international randomized phase III trial. *Neuro. Oncol.* **25**, 123–134 (2023). 10.1093/neuonc/noac099 PMC9825306 35419607

[31] Alafandi, A. et al. Association of pre-radiotherapy tumour burden and overall survival in newly diagnosed glioblastoma adjusted for MGMT promoter methylation status. *Eur. J. Cancer* **188**, 122–130 (2023). 10.1016/j.ejca.2023.04.021 37235895

[32] Natsume, A. et al. Genetic analysis in patients with newly diagnosed glioblastomas treated with interferon-beta plus temozolomide in comparison with temozolomide alone. *J. Neurooncol.* **148**, 17–27 (2020). 10.1007/s11060-020-03505-9 32367437

[33] Vogl, D. T. et al. Targeted interferon therapy with modakafusp alfa for relapsed or refractory multiple myeloma. *Blood* **145**, 944–955 (2025). 10.1182/blood.2024026124 PMC11969263 39630057

[34] Chiocca, E. A. Regulatable interleukin-12 gene therapy in patients with recurrent high-grade glioma: Results of a phase 1 trial. *Sci. Transl. Med.* **11**, eaaw5680 (2019). 10.1126/scitranslmed.aaw5680 PMC7286430 31413142

[35] Boorjian, S. A. et al. Intravesical nadofaragene firadenovec gene therapy for BCG-unresponsive non-muscle-invasive bladder cancer: a single-arm, open-label, repeat-dose clinical trial. *Lancet Oncol.* **22**, 107–117 (2021). 10.1016/S1470-2045(20)30540-4 PMC7988888 33253641

[36] Low, J. T. Understanding and therapeutically exploiting cGAS/STING signaling in glioblastoma. *J. Clin. Invest.* **134**, e163452 (2024). 10.1172/JCI163452 PMC10786687 38226619

[37] Zhai, Y. et al. T lymphocyte membrane-decorated epigenetic nanoinducer of interferons for cancer immunotherapy. *Nat. Nanotechnol.* **16**, 1271–1280 (2021). 10.1038/s41565-021-00972-7 34580467

[38] Chen, D. et al. Tumor Treating Fields dually activate STING and AIM2 inflammasomes to induce adjuvant immunity in glioblastoma. *J. Clin. Invest.* **132**, e149258 (2022). 10.1172/JCI149258 PMC9012294 35199647

[39] Awada, H., Paris, F. & Pecqueur, C. Exploiting radiation immunostimulatory effects to improve glioblastoma outcome. *Neuro. Oncol.* **25**, 433–446 (2023). 10.1093/neuonc/noac239 PMC10013704 36239313

[40] Nassiri, F. et al. Oncolytic DNX-2401 virotherapy plus pembrolizumab in recurrent glioblastoma: a phase 1/2 trial. *Nat. Med.* **29**, 1370–1378 (2023). 10.1038/s41591-023-02347-y PMC10287560 37188783

[41] Ling, A. L. et al. Clinical trial links oncolytic immunoactivation to survival in glioblastoma. *Nature* **623**, 157–166 (2023). 10.1038/s41586-023-06623-2 PMC10620094 37853118

[42] Benci, J. L. et al. Tumor interferon signaling regulates a multigenic resistance program to immune checkpoint blockade. *Cell* **167**, 1540–1554 (2016). 10.1016/j.cell.2016.11.022 PMC5385895 27912061

[43] Li, S. et al. STING-induced regulatory B cells compromise NK function in cancer immunity. *Nature* **610**, 373–380 (2022). 10.1038/s41586-022-05254-3 PMC9875944 36198789

[44] Wu, J., Dobbs, N., Yang, K. & Yan, N. Interferon-independent activities of mammalian STING mediate antiviral response and tumor immune evasion. *Immunity* **53**, 115–126 (2020). 10.1016/j.immuni.2020.06.009 PMC7365768 32640258

[45] Hodgins, J. J. et al. PD-L1 promotes oncolytic virus infection via a metabolic shift that inhibits the type I IFN pathway. *J. Exp. Med.* **221**, e20221721 (2024). 10.1084/jem.20221721 PMC11176258 38869480

[46] Feldman, L. et al. Hypoxia within the glioblastoma tumor microenvironment: a master saboteur of novel treatments. *Front. Immunol.* **15**, 1384249 (2024). 10.3389/fimmu.2024.1384249 PMC11238147 38994360

[47] Wang, A. Z. et al. Glioblastoma-infiltrating CD8^+^ T cells are predominantly a clonally expanded GZMK+ effector population. *Cancer Discov.* **14**, 1106–1131 (2024). 10.1158/2159-8290.CD-23-0913 PMC13480461 38416133

[48] Lawrence, M. S. et al. Mutational heterogeneity in cancer and the search for new cancer-associated genes. *Nature* **499**, 214–218 (2013). 10.1038/nature12213 PMC3919509 23770567

[49] Keskin, D. B. et al. Neoantigen vaccine generates intratumoral T cell responses in phase Ib glioblastoma trial. *Nature* **565**, 234–239 (2019). 10.1038/s41586-018-0792-9 PMC6546179 30568305

[50] Bernstock, J. D., Gerstl, J. V. E., Valdés, P. A., Friedman, G. K. & Chiocca, E. A. Next-generation CAR T cell therapies for glioblastoma. *Sci. Transl. Med.* **16**, eadp2660 (2024). 10.1126/scitranslmed.adp2660 39196960

[51] Monje, M. et al. Intravenous and intracranial GD2-CAR T cells for H3K27M^+^ diffuse midline gliomas. *Nature* **637**, 708–715 (2025). 10.1038/s41586-024-08171-9 PMC11735388 39537919

[52] Grewal, E. P. et al. Clinical progress in the development of CAR T cells to treat malignant glioma. *J. Neurooncol.* **171**, 571–579 (2024). 10.1007/s11060-024-04909-7 39695004

[53] Zonari, E. et al. Efficient ex vivo engineering and expansion of highly purified human hematopoietic stem and progenitor cell populations for gene therapy. *Stem Cell Rep.* **8**, 977–990 (2017). 10.1016/j.stemcr.2017.02.010 PMC5390102 28330619

[54] Petrillo, C. et al. Cyclosporine H overcomes innate immune restrictions to improve lentiviral transduction and gene editing in human hematopoietic stem cells. *Cell Stem Cell* **23**, 820–832.e9 (2018). 10.1016/j.stem.2018.10.008 PMC6292841 30416070

[55] Nishiga, Y. et al. Radiotherapy in combination with CD47 blockade elicits a macrophage-mediated abscopal effect. *Nat. Cancer* **3**, 1351–1366 (2022). 10.1038/s43018-022-00456-0 PMC9701141 36411318

[56] Gardell, J. L. et al. Human macrophages engineered to secrete a bispecific T cell engager support antigen-dependent T cell responses to glioblastoma. *J. Immunother. Cancer* **8**, e001202 (2020). 10.1136/jitc-2020-001202 PMC7597484 33122397

[57] Klichinsky, M. et al. Human chimeric antigen receptor macrophages for cancer immunotherapy. *Nat. Biotechnol.* **38**, 947–953 (2020). 10.1038/s41587-020-0462-y PMC7883632 32361713

[58] Chen, C. et al. Intracavity generation of glioma stem cell–specific CAR macrophages primes locoregional immunity for postoperative glioblastoma therapy. *Sci. Transl. Med.* **14**, eabn1128 (2022). 10.1126/scitranslmed.abn1128 35921473

[59] Czechowicz, A., Palchaudhuri, R. & Scheck, A. Selective hematopoietic stem cell ablation using CD117-antibody-drugconjugates enables safe and effective transplantation with immunity preservation. *Transplantation* **103**, 1526 (2019). 10.1038/s41467-018-08201-x PMC6365495 30728354

[60] Omer-Javed, A. et al. Mobilization-based chemotherapy-free engraftment of gene-edited human hematopoietic stem cells. *Cell* **185**, 2248–2264 (2022). 10.1016/j.cell.2022.04.039 PMC9240327 35617958

[61] Cesana, D. et al. A case of T-cell acute lymphoblastic leukemia in retroviral gene therapy for ADA-SCID. *Nat. Commun.* **10**, 3662 (2024). 10.1038/s41467-024-47866-5 PMC11061298 38688902

[62] Spinozzi, G. et al. VISPA2: A scalable pipeline for high-throughput identification and annotation of vector integration sites. *BMC Bioinformatics* **18**, 520 (2017). 10.1186/s12859-017-1937-9 PMC5702242 29178837

[63] Pais, G. et al. ISAnalytics enables longitudinal and high-throughput clonal tracking studies in hematopoietic stem cell gene therapy applications. *Brief. Bioinform.* **24**, bbac551 (2022). 10.1093/bib/bbac551 PMC9910212 36545803

[64] Berry, C. C. et al. Estimating abundances of retroviral insertion sites from DNA fragment length data. *Bioinformatics* **28**, 755–762 (2012). 10.1093/bioinformatics/bts004 PMC3307109 22238265

[65] Biffi, A. et al. Lentiviral vector common integration sites in preclinical models and a clinical trial reflect a benign integration bias and not oncogenic selection. *Blood* **117**, 5332–5339 (2011). 10.1182/blood-2010-09-306761 21403130

[66] Pagani, F. et al. Concurrent RB1 and P53 pathway disruption predisposes to the development of a primitive neuronal component in high-grade gliomas depending on MYC-driven EBF3 transcription. *Acta Neuropathol.* 10.1007/s00401-025-02845-y (2025). 39821672

[67] Ruiz-Moreno, C. et al. Harmonized single-cell landscape, intercellular crosstalk and tumor architecture of glioblastoma. Preprint at *bioRxiv* 10.1101/2022.08.27.505439 (2022).

[68] Mathewson, N. D. et al. Inhibitory CD161 receptor identified in glioma-infiltrating T cells by single-cell analysis. *Cell* **184**, 1281–1298 (2021). 10.1016/j.cell.2021.01.022 PMC7935772 33592174

[69] Miller, T. E. et al. Programs, origins and immunomodulatory functions of myeloid cells in glioma. *Nature* **640**, 1072–1082 (2025). 10.1038/s41586-025-08633-8 PMC12018266 40011771

[70] Lee, A. H. et al. Neoadjuvant PD-1 blockade induces T cell and cDC1 activation but fails to overcome the immunosuppressive tumor associated macrophages in recurrent glioblastoma. *Nat. Commun.* **12**, 6938 (2021). 10.1038/s41467-021-26940-2 PMC8626557 34836966

[71] Browaeys, R. NicheNet-v2: final networks and ligand-target matrices. *Zenodo*10.5281/zenodo.7074290 (2022).

