# Supplementary material for: Tumor-targeted interferon-α gene therapy for glioblastoma: a phase 1 trial
Source: Nat Med. 2026 Jun 1;32(6):2216–26. doi: 10.1038/s41591-026-04419-1 (PMC13768172; doi:10.1038/s41591-026-04419-1)
Supplement: Supplementary file 1 [file 41591_2026_4419_MOESM1_ESM.pdf]

nature medicine

Supplementary information

https://doi.org/10.1038/s41591-026-04419-1

Tumor-targeted interferon-α gene therapy for glioblastoma: a phase 1 trial

In the format provided by the authors and unedited

Supplementary Table 1: Baseline patient characteristics (TEM-GBM_01 study)

**Patient code**	**Sex**	**Age** (years)	**ECOG**	**KPS**	**Dexa at screening** daily equiv (mg)	**Dexa at baseline** daily equiv (mg)	**Clinically significant comorbidity**
TEM-G6	F	64	1	80	6	6	
TEM-G7	M	38	0	100	0	4	
TEM-G8	F	63	1	80	0	2	Bronchiectasis
TEM-G9	F	59	0	100	0	1	
TEM-G11	M	61	1	80	0	2	
TEM-G13	M	29	0	100	1.5	1	
TEM-G14	F	45	0	100	0	4	
TEM-G15	F	66	1	80	2	2.5	Hypertension
TEM-G16	M	56	0	90	0	16	Hypertension Hypercholesterolemia
TEM-G18	M	48	1	80	4	2	
TEM-G19	M	59	1	80	2	0.75	Prostate cancer, resected
TEM-G20	M	61	1	80	0	0.5	
TEM-G21	M	47	0	100	0	4	
TEM-G22	M	57	1	80	0	4	
TEM-G23	F	57	1	80	4	4	Metabolic syndrome
TEM-G26	F	64	1	80	0	0	
TEM-G28	M	52	1	70	0	4	Hypertension
TEM-G29	F	47	0	100	0	1	
TEM-G30	M	59	0	90	0	0	Ischemia
TEM-G31	M	65	1-2	70	0	8	Hypertension Hypercholesterolemia, arteriosclerosis
TEM-G32	M	45	1	80	1.5	1.5	
TEM-G34	M	61	1	80	0	2	Hypertension Hypercholesterolemia
TEM-G37	M	63	0	100	0	0	Atrial fibrillation (resolved)
TEM-G39	F	67	0	90	0	2	Hypertension Hypercholesterolemia
**Median**	9F/ 15M	**59**	**1**	**80**	**1.75**	**2.00**	

All patients were of European origin (White, non-Hispanic or -Latino)

Supplementary Table 2: Tumor characteristics at diagnosis

Patient Code	Tumor localization	#target lesions (TL)	Resection	PTEN	EGFR	EGFR vIII	P53	FGFR3	GFAP
TEM-G6	Left frontal lobe	1	complete	0	0	-	0	0	-
TEM-G7	Right insula	1	complete	-	-	-	0	-	1
TEM-G8	Left tempo-parietal	1	complete	-	0	-	1	-	1
TEM-G9	Right temporal lobe	1	partial	1	0	-	1	-	-
TEM-G11	Right frontal lobe	4	partial	0	1	-	1	-	1
TEM-G13	Right fronto-insular	0	complete	1	0	-	0	0	1
TEM-G14	Right parasagittal parietal	1	partial	-	0	-	0	-	1
TEM-G15	Right frontal lobe	0	complete	-	1	1	0	-	1
TEM-G16	Left frontal lobe	1	complete	0	1	1	0	-	-
TEM-G18	Left temporo-parietal	2	complete	-	1	1	0	-	-
TEM-G19	Left parietal lobe	0	complete	-	-	-	1	-	1
TEM-G20	Left parietal lobe	0	complete	-	1	1	0	-	1
TEM-G21	Left frontal lobe	1	partial	1	1	-	1	0	1
TEM-G22	Left temporo-parietal	2	partial	-	-	-	1	-	1
TEM-G23	Left frontal (multifocal)	1	partial	-	-	-	1	-	1
TEM-G26	Left temporal lobe	1	complete	-	0	-	1	-	0
TEM-G28	Left fronto-parietal	2	complete	-	1	-	0	-	1
TEM-G29	Left parieto-occipital	0	complete	-	-	-	0	-	1
TEM-G30	Right parietal lobe	1	complete	-	1	-	0	-	1
TEM-G31	Intraxial frontomesial left	2	complete	-	1	-	1	1	1
TEM-G32	Left parietal lobe	2	partial	-	-	-	1	-	1
TEM-G34	Left parietal lobe	0	complete	1	0	0	1	-	1
TEM-G37	Right frontal lobe	0	partial	-	1	-	0	-	1
TEM-G39	Fronto-temporal	0	complete	-	-	-	-	0	-

Supplementary Table 3: Temferon product characteristics

Patient code	Apheresis	Temferon DP characterization			Temferon infused	
	$x10^6$ $CD34^+$/kg	$\%CD34^+$	VCN/ genome	% LV^+ CFU	$x10^6$/kg total cells	$x10^6$/kg LV^+ cells
TEM-G6	4.9	100	0.6	63	0.5	0.3
TEM-G7	14.7	100	0.7	58	0.5	0.3
TEM-G8	11.4	100	0.38	35	0.8↑	0.3
TEM-G9	9	100	0.7	49	1.1	0.5
TEM-G10	Screening failure					
TEM-G11	8.1	100	0.5	49	1	0.5
TEM-G12	8.8	100	1	50	Withdrawn	
TEM-G13	11	100	0.9	45	1.1	0.5
TEM-G14	9.7	100	0.9	63	2.1	1.3
TEM-G15	7.5[2]	99	0.8	65	2	1.3
TEM-G16	12.4	100	0.8	58	2.1	1.2
TEM-G17	Screening failure					
TEM-G18	8.5[2]	99	0.9	49	1.5*	0.7
TEM-G19	8.4	100	1.1	59	1.6↓	0.9
TEM-G20	9.9[2]	98	0.5	45	2.2	1
TEM-G21	12.6	99	0.9	50	2.1	1.1
TEM-G22	10.9	100	0.8	53	2.2	1.2
TEM-G23	13.1	100	0.4	39	2.1	0.8
TEM-G24	Withdrawn					
TEM-G25	Screening failure					
TEM-G26	14.7	100	0.5	71	3.2	2.3
TEM-G27	Screening failure					
TEM-G28	16.3	100	0.5	51	3.3	1.7
TEM-G29	13	100	0.3	29	3.0	0.9
TEM-G30	10.7	100	0.5	51	3.2	1.7
TEM-G31	9.8	99	0.8	43	3.2	1.4
TEM-G32	7	100	1.6	>80	1.6↓	1.3
TEM-G33	Screening failure					
TEM-G34	8	99	0.5	63	4.3	2.7
TEM-G35	7.7	100	1.1	68	Withdrawn	
TEM-G36	9.6[2]	100	0.9	68	Withdrawn	
TEM-G37	11	100	0.6	56	4.4	2.5
TEM-G38	Withdrawn					
TEM-G39	9	100	0.8	67	4.3	2.9
**Mean** ±SD	**10.3** ±2.7	**99.7** ±0.5	**0.7** ±0.3	**54** ±11		

[2]: Two consecutive days of apheresis to obtain the target dose

*: Total Temferon dose was below the protocol-defined target, due to poor mobilization and below average process yield

↑: Total Temferon dose was increased based on low VCN, as defined in the protocol

↓: Total Temferon dose was reduced based on high VCN, as defined in the protocol

Supplementary Table 4: Overview of adverse events

Adverse Event Subcategory/ Attribution	Total		Cohort 1		Cohort 2		Cohort 3		Cohort 4		Cohort 5		Cohort 6		Cohort 7		Cohort 8		Events per subject		
	Frequency	No. Events	Frequency	No. Events	Frequency	No. Events	Frequency	No. Events	Frequency	No. Events	Frequency	No. Events	Frequency	No. Events	Frequency	No. Events	Frequency	No. Events	C/T	Bu/T	Bu
All Adverse Events (AEs)	100%	1004	100%	177	100%	122	100%	201	100%	146	100%	147	100%	97	100%	69	100%	45	49.5	49.0	19.0
Treatment Emergent Adverse Events (TEAEs)	100%	880	100%	151	100%	98	100%	170	100%	132	100%	136	100%	88	100%	64	100%	41	42.6	45.3	17.5
Severe Adverse Events (SAEs)	66.7%	32	100%	7	66.7%	2	100%	6	66.70%	2	100%	6	33.3%	6	66.7%	3	0%	0	1.5	2.0	0.5
Adverse Drug Reactions (ADRs)	70.80%	47	100%	11	66.7%	5	66.7%	3	66.7%	5	66.7%	4	66.7%	10	100%	5	33.3%	4	2.3	1.3	1.5
Serious ADRs	4.20%	1		0		0		1		0		0		0		0		0			
AEs attributed to conditioning regimen	100%	536	100%	65	100%	40	100%	107	100%	91	100%	88	100%	68	100%	40	100%	37	24.7	29.3	12.8
SAEs attributed to conditioning regimen	12.50%	5	33.3%	1		0		0		0		0	33.3%	3	33.3%	1		0			
AEs related to other concomitant medications/procedures	100%	286	100%	66	100%	53	100%	73	100%	26	100%	37	100%	8	100%	18	100%	5	15.1	12.3	3.8
AEs related to Temferon (IMP) infusion procedure	4.20%	1		0		1		0		0		0		0		0		0			
AEs related to harvesting of HSPCs	4.20%	4		0		0		0		4		0		0		0		0			
Severe AEs	100%	234	100%	38	100%	25	100%	54	100%	33	100%	33	100%	31	100%	11	100%	9	12.1	11.0	3.3
AEs leading to temporary and permanent discontinuation		0		0		0		0		0		0		0		0		0			
AEs leading to Death	16.70%	7	33.3%	1		0	33.3%	1		0	33.3%	2	33.3%	3		0		0			

Note 1: Adverse events with onset date/time >= date/time Temferon infusion are considered as TEAEs. Events identified as same as a previous pre-treatment event, and with Severity not worsened have been excluded and only reported as Pre-treatment events.
Note 2: SAE = Serious Adverse Event, ADR = Adverse drug reaction.
Note 3: Percentages (frequency) are calculated on the number of subjects (N).
Note 4: An event with different seriousness, severity, relationship and outcome is counted once per each subcategory. An event occurring during multiple time ranges is counted once per each time range.
Note 5: Events per subject (average) according to conditioning regimen: C/T: Carmustin/Thiotepa (cohorts 1-4, 6), n=15 ; Bu/T: Busulfan/Thiotepa (cohort 5), n=3; Bu: Busulfan only (cohorts 7,8), n=6

Supplementary Table 5: Pre-treatment adverse events

System Organ Class<br>Preferred Term	Number of subjects	Frequency	Number of events
**Any Pre-Treatment AE**	**22**	**91.70%**	**124**
**Blood and lymphatic system disorders**	**15**	**62.50%**	**39**
Lymphopenia	15	62.50%	32
Anaemia	2	8.30%	3
Thrombocytopenia	2	8.30%	3
Neutropenia	1	4.20%	1
**Nervous system disorders**	**10**	**41.70%**	**17**
Headache	6	25.00%	8
Dizziness	4	16.70%	6
Brain oedema	2	8.30%	2
Seizure	1	4.20%	1
**Gastrointestinal disorders**	**8**	**33.30%**	**13**
Nausea	7	29.20%	7
Constipation	2	8.30%	2
Vomiting	2	8.30%	2
Abdominal pain	1	4.20%	1
Diarrhoea	1	4.20%	1
**General disorders and administration site conditions**	**8**	**33.30%**	**10**
Asthenia	4	16.70%	4
Oedema peripheral	2	8.30%	2
Catheter site pain	1	4.20%	1
Fatigue	1	4.20%	1
Mucosal inflammation	1	4.20%	1
Pyrexia	1	4.20%	1
**Injury, poisoning and procedural complications**	**7**	**29.20%**	**8**
Infusion related reaction	7	29.20%	7
Facial bones fracture	1	4.20%	1
**Investigations**	**6**	**25.00%**	**11**
Alanine aminotransferase increased	2	8.30%	2
Blood lactate dehydrogenase increased	2	8.30%	2
Gamma-glutamyltransferase increased	2	8.30%	2
Aspartate aminotransferase increased	1	4.20%	1
Blood alkaline phosphatase increased	1	4.20%	1
Lymphocyte count decreased	1	4.20%	1
Staphylococcus test positive	1	4.20%	1
Urogram	1	4.20%	1

System Organ Class<br>Preferred Term	Number of subjects	Frequency	Number of events
**Metabolism and nutrition disorders**	**5**	**20.80%**	**8**
Decreased appetite	2	8.30%	2
Hypokalaemia	2	8.30%	2
Hyperglycaemia	1	4.20%	1
Hypoalbuminaemia	1	4.20%	1
Hypomagnesaemia	1	4.20%	1
Hyponatraemia	1	4.20%	1
**Psychiatric disorders**	**4**	**16.70%**	**5**
Insomnia	4	16.70%	4
Agitation	1	4.20%	1
**Vascular disorders**	**3**	**12.50%**	**3**
Hypertension	2	8.30%	2
Hypotension	1	4.20%	1
**Ear and labyrinth disorders**	**2**	**8.30%**	**2**
Ear disorder	1	4.20%	1
Tinnitus	1	4.20%	1
**Infections and infestations**	**2**	**8.30%**	**2**
Herpes simplex	1	4.20%	1
Upper respiratory tract infection	1	4.20%	1
**Cardiac disorders**	**1**	**4.20%**	**1**
Bradycardia	1	4.20%	1
**Eye disorders**	**1**	**4.20%**	**1**
Periorbital oedema	1	4.20%	1
**Immune system disorders**	**1**	**4.20%**	**1**
Hypogammaglobulinaemia	1	4.20%	1
**Musculoskeletal and connective tissue disorders**	**1**	**4.20%**	**1**
Bone pain	1	4.20%	1
**Respiratory, thoracic and mediastinal disorders**	**1**	**4.20%**	**1**
Hypoxia	1	4.20%	1
**Skin and subcutaneous tissue disorders**	**1**	**4.20%**	**1**
Rash	1	4.20%	1

Note 1: Adverse events with onset date/time < date/time Temferon infusion are presented in this table.
Note 2: Adverse events have been coded using the MedDRA dictionary (version 21.1). System Organ Class and Preferred Term are presented.
Note 3: Subjects are only counted once per cohort for each row in the column Number (%) of subjects.
Note 4: Percentages are calculated on the number of subjects (N).

Supplementary Table 6: Treatment emergent adverse events by system organ class and preferred term

	Number of subjects (%)	Number of events (days from Temferon)					% of subjects with TEAE by severity (CTCAE v5.0)					Distribution across cohorts								Frequency by conditioning		
		total	0-30	31-90	91-360	361-720	1	2	3	4	5	1	2	3	4	5	6	7	8	C/T	Bu/T	Bu
**Any TEAE**	**24 (100%)**	**847**	**427**	**220**	**157**	**43**	**100%**	**100%**	**100%**	**100%**	**17%**	**13%**	**13%**	**13%**	**13%**	**13%**	**13%**	**13%**	**13%**	**100%**	**100%**	**100%**
**Blood and lymphatic system disorders**	24	359	262	66	26	5	100%	96%	100%	100%	0%	13%	13%	13%	13%	13%	13%	13%	13%	100%	100%	100%
Anaemia	24	78	38	29	10	1	96%	54%	25%	0%	0%	13%	13%	13%	13%	13%	13%	13%	13%	100%	100%	100%
Neutropenia	24	83	67	11	5		67%	71%	88%	96%	0%	13%	13%	13%	13%	13%	13%	13%	13%	100%	100%	100%
Thrombocytopenia	24	111	93	14	3	1	79%	96%	83%	29%	0%	13%	13%	13%	13%	13%	13%	13%	13%	100%	100%	100%
Lymphopenia	21	65	45	10	8	2	46%	63%	63%	46%	0%	14%	10%	14%	14%	14%	5%	14%	14%	80%	100%	100%
Febrile neutropenia	16	21	19	2			0%	0%	58%	17%	0%	13%	13%	19%	13%	19%	19%	6%	0%	80%	100%	17%
Eosinophilia	1	1				1	4%	0%	0%	0%	0%	0%	0%	0%	0%	0%	100%	0%	0%	7%	0%	0%
**Investigations**	24 (100%)	219	62	69	65	23	96%	83%	38%	0%	0%	13%	13%	13%	13%	13%	13%	13%	13%	100%	100%	100%
Alanine aminotransferase increased	19	39	21	8	8	2	67%	42%	8%	0%	0%	11%	11%	11%	16%	11%	16%	16%	11%	80%	67%	83%
Aspartate aminotransferase increased	17	29	13	8	8		67%	17%	0%	0%	0%	6%	12%	6%	18%	6%	18%	18%	18%	67%	33%	100%
Gamma-glutamyltransferase increased	17	32	14	9	7	2	58%	33%	13%	0%	0%	6%	12%	12%	12%	18%	18%	12%	12%	67%	100%	67%
Blood lactate dehydrogenase increased	16	22	7	6	6	3	38%	0%	0%	0%	0%	19%	19%	13%	19%	13%	0%	13%	6%	73%	67%	50%
Lymphocyte count decreased	16	22		12	8	2	21%	42%	25%	0%	0%	13%	19%	6%	19%	13%	13%	6%	13%	73%	67%	50%
White blood cell count decreased	15	24		12	9	3	50%	33%	8%	0%	0%	13%	13%	7%	20%	13%	7%	13%	13%	60%	67%	67%
Platelet count decreased	10	13	1	8	1	3	42%	8%	0%	0%	0%	10%	20%	0%	20%	20%	0%	20%	10%	33%	67%	50%
Neutrophil count decreased	6	7		2	4	1	17%	13%	0%	0%	0%	17%	0%	0%	33%	33%	0%	17%	0%	20%	67%	17%
C-reactive protein increased	5	5	2		3		4%	0%	0%	0%	0%	20%	0%	20%	20%	0%	40%	0%	0%	33%	0%	0%
Blood alkaline phosphatase increased	4	7	2	1	3	1	17%	0%	0%	0%	0%	0%	25%	25%	0%	0%	50%	0%	0%	27%	0%	0%
Fibrin D dimer increased	4	5			2	3	0%	0%	0%	0%	0%	25%	0%	0%	0%	25%	0%	50%	0%	7%	33%	33%
Weight decreased	4	4		2	2		13%	4%	0%	0%	0%	50%	0%	0%	25%	0%	0%	25%	0%	20%	0%	17%
Anti-thyroid antibody positive	2	3			2	1	0%	0%	0%	0%	0%	0%	0%	0%	0%	0%	50%	50%	0%	7%	0%	17%
Blood cholesterol increased	1	1				1	4%	0%	0%	0%	0%	0%	0%	100%	0%	0%	0%	0%	0%	7%	0%	0%
ECOG performance status worsened	2	3	2	1			0%	0%	4%	0%	0%	50%	0%	0%	50%	0%	0%	0%	0%	13%	0%	0%
Blood corticotrophin abnormal	1	1				1	0%	0%	0%	0%	0%	0%	0%	100%	0%	0%	0%	0%	0%	7%	0%	0%
C-reactive protein	1	1			1		0%	0%	0%	0%	0%	0%	100%	0%	0%	0%	0%	0%	0%	7%	0%	0%
Oxygen saturation decreased	1	1			1		0%	0%	0%	0%	0%	0%	0%	100%	0%	0%	0%	0%	0%	7%	0%	0%
**General disorders and administration site conditions**	22 (91.7%)	47	19	16	10	2	75%	33%	4%	0%	8%	14%	9%	14%	14%	14%	14%	14%	9%	93%	100%	83%
Asthenia	12	12	5	4	2	1	33%	17%	0%	0%	0%	8%	17%	8%	25%	8%	17%	8%	8%	60%	33%	33%
Mucosal inflammation	12	8	8				42%	13%	4%	0%	0%	17%	8%	8%	17%	8%	0%	25%	17%	40%	33%	83%
Pyrexia	8	12	4	5	3		33%	0%	0%	0%	0%	38%	13%	25%	13%	13%	0%	0%	0%	47%	33%	0%
Fatigue	5	6	1	3	2		17%	8%	0%	0%	0%	20%	20%	0%	0%	40%	20%	0%	0%	20%	67%	0%
Hyperpyrexia	3	3		1	2		8%	0%	0%	0%	0%	33%	33%	33%	0%	0%	0%	0%	0%	20%	0%	0%
Chills	1	1	1				4%	0%	0%	0%	0%	100%	0%	0%	0%	0%	0%	0%	0%	7%	0%	0%
Multiple organ dysfunction syndrome	1	1		1			0%	0%	0%	0%	4%	0%	0%	0%	0%	0%	100%	0%	0%	7%	0%	0%
Oedema peripheral	1	1			1		4%	0%	0%	0%	0%	0%	0%	0%	0%	100%	0%	0%	0%	0%	33%	0%
Pain	1	2		2			0%	0%	0%	0%	0%	0%	0%	100%	0%	0%	0%	0%	0%	7%	0%	0%
Sudden death	1	1				1	0%	0%	0%	0%	4%	100%	0%	0%	0%	0%	0%	0%	0%	7%	0%	0%
**Nervous system disorders**	18 (75%)	37	4	18	14	1	42%	25%	21%	4%	0%	17%	6%	17%	17%	17%	17%	6%	6%	87%	100%	33%
Seizure	5	9	2	1	6		8%	13%	8%	0%	0%	20%	20%	0%	40%	0%	20%	0%	0%	33%	0%	0%
Dysgeusia	4	3		2	1		13%	4%	0%	0%	0%	50%	0%	0%	25%	0%	0%	0%	25%	20%	0%	17%
ICU acquired weakness	3	3	1	2			0%	4%	4%	0%	0%	0%	0%	67%	0%	33%	0%	0%	0%	13%	33%	0%
Somnolence	3	3		1	2		8%	0%	0%	0%	0%	0%	0%	33%	33%	0%	33%	0%	0%	20%	0%	0%
Dysaesthesia	2	2	1	1			4%	0%	0%	0%	0%	50%	0%	0%	0%	0%	0%	50%	0%	7%	0%	17%
Memory impairment	2	2		2			4%	0%	0%	0%	0%	0%	0%	0%	0%	50%	50%	0%	0%	7%	33%	0%
Partial seizures	2	2		1	1		0%	8%	0%	0%	0%	0%	0%	0%	50%	0%	50%	0%	0%	13%	0%	0%
Ageusia	1	1		1			4%	0%	0%	0%	0%	0%	0%	0%	0%	0%	100%	0%	0%	7%	0%	0%
Aphasia	1	1		1			4%	0%	0%	0%	0%	0%	0%	0%	100%	0%	0%	0%	0%	7%	0%	0%
Autonomic nerv. syst. imbalance	1	1		1			0%	0%	0%	0%	0%	0%	0%	100%	0%	0%	0%	0%	0%	7%	0%	0%
Dizziness	1	1		1			4%	0%	0%	0%	0%	100%	0%	0%	0%	0%	0%	0%	0%	7%	0%	0%
Epilepsy	1	1		1			0%	0%	4%	0%	0%	0%	0%	0%	0%	0%	100%	0%	0%	7%	0%	0%
Headache	1	1				1	4%	0%	0%	0%	0%	0%	0%	0%	0%	100%	0%	0%	0%	0%	33%	0%
Hemiparesis	1	2			2		0%	0%	4%	0%	0%	100%	0%	0%	0%	0%	0%	0%	0%	7%	0%	0%
Lhermitte's sign	1	1			1		4%	0%	0%	0%	0%	0%	100%	0%	0%	0%	0%	0%	0%	7%	0%	0%
Parosmia	1	1		1			0%	0%	0%	0%	0%	0%	0%	0%	0%	0%	0%	0%	100%	0%	0%	17%
Radiculopathy	1	1		1			4%	0%	0%	0%	0%	0%	0%	0%	100%	0%	0%	0%	0%	7%	0%	0%
Status epilepticus	1	1			1		0%	0%	0%	4%	0%	0%	0%	0%	0%	100%	0%	0%	0%	0%	33%	0%
Tremor	1	1		1			4%	0%	0%	0%	0%	0%	0%	0%	0%	0%	100%	0%	0%	7%	0%	0%
**Metabolism and nutrition disorders**	16 (66.7%)	25	12	7	4	2	54%	13%	4%	4%	0%	19%	13%	19%	19%	19%	0%	6%	6%	73%	100%	33%
Decreased appetite	10	12	8	3	1		33%	8%	4%	0%	0%	20%	10%	20%	20%	20%	0%	0%	10%	47%	67%	17%
Hypokalaemia	5	5	1	2	2		17%	4%	0%	0%	0%	20%	20%	0%	40%	20%	0%	0%	0%	27%	33%	0%
Hypoalbuminaemia	3	3	1	2			4%	13%	0%	0%	0%	67%	0%	33%	0%	0%	0%	0%	0%	20%	0%	0%
Diabetes mellitus	1	1	1				0%	4%	0%	0%	0%	0%	0%	100%	0%	0%	0%	0%	0%	7%	0%	0%
Hypertriglyceridaemia	1	1			1		4%	0%	0%	0%	0%	0%	0%	0%	0%	0%	0%	100%	0%	0%	0%	17%
Hypoglycaemia	1	1				1	0%	0%	0%	4%	0%	0%	0%	0%	0%	100%	0%	0%	0%	0%	33%	0%
Hyponatraemia	1	1	1				4%	0%	0%	0%	0%	100%	0%	0%	0%	0%	0%	0%	0%	7%	0%	0%
Vitamin D deficiency	1	1				1	4%	0%	0%	0%	0%	0%	0%	100%	0%	0%	0%	0%	0%	7%	0%	0%
**Gastrointestinal disorders**	15 (62.5%)	34	23	5	6		46%	29%	4%	0%	0%	13%	13%	20%	13%	20%	7%	7%	7%	67%	100%	33%
Diarrhoea	9	14	9	2	3		29%	17%	4%	0%	0%	22%	11%	0%	22%	22%	11%	11%	0%	40%	67%	17%
Nausea	6	7	5	1	1		13%	17%	4%	0%	0%	33%	0%	17%	17%	17%	0%	0%	17%	27%	33%	17%
Stomatitis	3	2	2				8%	0%	0%	0%	0%	33%	0%	0%	0%	67%	0%	0%	0%	7%	67%	0%
Vomiting	2	5	2	1	2		4%	8%	0%	0%	0%	50%	0%	0%	50%	0%	0%	0%	0%	13%	0%	0%
Abdominal pain upper	1	1	1				4%	0%	0%	0%	0%	0%	0%	100%	0%	0%	0%	0%	0%	7%	0%	0%
Anal incontinence	1	1		1			4%	0%	0%	0%	0%	0%	0%	100%	0%	0%	0%	0%	0%	7%	0%	0%
Constipation	1	0					4%	0%	0%	0%	0%	0%	0%	0%	0%	100%	0%	0%	0%	0%	33%	0%
Dysphagia	1	1	1				4%	0%	0%	0%	0%	0%	0%	100%	0%	0%	0%	0%	0%	7%	0%	0%
Haemorrhoids	1	1	1				0%	0%	0%	0%	0%	0%	0%	0%	0%	100%	0%	0%	0%	0%	33%	0%
Odynophagia	1	1	1				0%	0%	0%	0%	0%	0%	100%	0%	0%	0%	0%	0%	0%	7%	0%	0%
Oral pain	1	1	1				0%	4%	0%	0%	0%	0%	0%	0%	0%	100%	0%	0%	0%	0%	33%	0%

Note 1: Adverse events with onset date/time >= date/time study medication intake are presented in this table (TEAE, treatment emergent adverse events). Events identified as same as a previous pre-treatment event, and with Severity not worsened have been excluded and only reported as Pre-treatment events.

Note 2: Adverse events have been coded using the MedDRA dictionary (version 21.1). System Organ Class and Preferred Term are presented.

Note 3: If more than one adverse event occurred with the same Preferred Term for the same subject, the subject is counted only once for that Preferred Term for the “Number of subjects (%)” column, while he/she will be counted once for each reported severity for the CTCAE v5.0 grade columns.

Note 4: Percentages are calculated on the number of subjects (N), except for the "Distribution across cohorts" column, which shows the relative distribution of subjects with a given TEAE among the cohorts

Note 5: Frequency by conditioning refers to the % of subjects assigned to the indicated conditioning regimen, who experienced a given TEAE. C/T: Carmustin/Thiotepa; Bu/T: Busulfan/Thiotepa; Bu: Busulfan only

	Number of subjects (%)	Number of events (days from Temferon)					% of subjects with TEAE by severity (CTCAE v5.0)					Distribution across cohorts								Frequency by conditioning		
		total	0-30	31-90	91-360	361-720	1	2	3	4	5	1	2	3	4	5	6	7	8	C/T	Bu/T	Bu
**Infections and infestations**	14 (58.3%)	41	13	14	11	3	29%	25%	25%	4%	8%	21%	14%	21%	0%	21%	14%	7%	0%	67%	100%	17%
Pneumonia	6	6	1	3	2		0%	8%	8%	4%	8%	17%	0%	17%	0%	33%	17%	17%	0%	20%	67%	17%
Cytomegalovirus infection	5	9	3	5	1		8%	8%	4%	0%	0%	0%	20%	40%	0%	20%	20%	0%	0%	27%	33%	0%
Corona virus infection	3	4			3	1	4%	4%	0%	0%	0%	33%	33%	0%	0%	33%	0%	0%	0%	13%	33%	0%
Clostridium difficile infection	2	3	1	1	1		4%	8%	0%	0%	0%	0%	0%	50%	0%	50%	0%	0%	0%	7%	33%	0%
Skin infection	2	2	1	1			4%	0%	4%	0%	0%	0%	50%	0%	0%	0%	50%	0%	0%	13%	0%	0%
Bacteriuria	1	1				1	0%	0%	0%	0%	0%	100%	0%	0%	0%	0%	0%	0%	0%	7%	0%	0%
Brain abscess	1	1			1		0%	0%	4%	0%	0%	0%	100%	0%	0%	0%	0%	0%	0%	7%	0%	0%
Bronchitis	1	1			1		4%	0%	0%	0%	0%	100%	0%	0%	0%	0%	0%	0%	0%	7%	0%	0%
Candiduria	1	1	1				0%	0%	0%	0%	0%	0%	0%	100%	0%	0%	0%	0%	0%	7%	0%	0%
Citrobacter infection	1	1	1				4%	0%	0%	0%	0%	0%	0%	0%	0%	100%	0%	0%	0%	0%	33%	0%
Escherichia infection	1	1	1				4%	0%	0%	0%	0%	0%	0%	0%	0%	100%	0%	0%	0%	0%	33%	0%
Escherichia urinary tract infection	1	1		1			0%	4%	0%	0%	0%	0%	0%	100%	0%	0%	0%	0%	0%	7%	0%	0%
Lung infection	1	2		2			0%	0%	4%	0%	0%	100%	0%	0%	0%	0%	0%	0%	0%	7%	0%	0%
Oral herpes	1	1	1				4%	0%	0%	0%	0%	100%	0%	0%	0%	0%	0%	0%	0%	7%	0%	0%
Paronychia	1	1	1				4%	0%	0%	0%	0%	0%	100%	0%	0%	0%	0%	0%	0%	7%	0%	0%
Pharyngitis	1	1				1	0%	4%	0%	0%	0%	0%	0%	100%	0%	0%	0%	0%	0%	7%	0%	0%
Pneumonia streptococcal	1	1	1				0%	0%	4%	0%	0%	0%	0%	0%	0%	0%	0%	100%	0%	0%	0%	17%
Pseudomonas infection	1	1	1				0%	0%	4%	0%	0%	0%	0%	0%	0%	100%	0%	0%	0%	0%	33%	0%
Sepsis	1	1			1		0%	0%	4%	0%	0%	0%	0%	0%	0%	100%	0%	0%	0%	0%	33%	0%
Septic shock	1	2		1	1		0%	0%	0%	0%	4%	0%	0%	0%	0%	0%	100%	0%	0%	7%	0%	0%
Urinary tract infection	1	0					4%	0%	0%	0%	0%	0%	0%	100%	0%	0%	0%	0%	0%	7%	0%	0%
**Skin and subcutaneous tissue disorders**	11 (45.8%)	15	10	3	1	1	21%	21%	4%	0%	0%	9%	18%	0%	18%	18%	18%	9%	9%	47%	67%	33%
Rash	8	10	8	2			13%	17%	4%	0%	0%	13%	0%	0%	25%	25%	25%	0%	13%	33%	67%	17%
Erythema	1	2		1	1		4%	0%	0%	0%	0%	0%	100%	0%	0%	0%	0%	0%	0%	7%	0%	0%
Intertrigo	1	1	1				4%	0%	0%	0%	0%	0%	0%	0%	0%	0%	0%	100%	0%	0%	0%	17%
Palmar erythema	1	1				1	0%	0%	0%	0%	0%	0%	0%	0%	0%	100%	0%	0%	0%	0%	33%	0%
Urticaria	1	1	1				0%	4%	0%	0%	0%	0%	100%	0%	0%	0%	0%	0%	0%	7%	0%	0%
**Respiratory, thoracic and mediastinal disorders**	10 (41.7%)	17	9	5	3		38%	4%	8%	4%	4%	30%	30%	20%	0%	0%	0%	10%	10%	53%	0%	33%
Cough	4	4	1	1	2		17%	4%	0%	0%	0%	50%	25%	0%	0%	0%	0%	25%	0%	20%	0%	17%
Pulmonary embolism	3	3		2	1		0%	0%	8%	4%	0%	33%	0%	0%	0%	0%	0%	33%	33%	7%	0%	33%
Epistaxis	2	2	2				8%	0%	0%	0%	0%	0%	0%	100%	0%	0%	0%	0%	0%	13%	0%	0%
Oropharyngeal pain	2	2	2				8%	0%	0%	0%	0%	0%	100%	0%	0%	0%	0%	0%	0%	13%	0%	0%
Dysphonia	1	3	3				4%	0%	0%	0%	0%	0%	0%	100%	0%	0%	0%	0%	0%	7%	0%	0%
Dyspnoea	1	1		1			4%	0%	0%	0%	0%	100%	0%	0%	0%	0%	0%	0%	0%	7%	0%	0%
Hiccups	1	2	1	1			4%	0%	0%	0%	0%	0%	0%	0%	0%	0%	0%	100%	0%	0%	0%	17%
Respiratory failure	1	0					0%	0%	0%	0%	4%	0%	0%	100%	0%	0%	0%	0%	0%	7%	0%	0%
**Vascular disorders**	9 (37.5%)	10	4	4	1	1	8%	17%	13%	0%	0%	22%	11%	22%	22%	0%	11%	0%	11%	53%	0%	17%
Hypertension	4	4	3			1	4%	8%	8%	0%	0%	50%	0%	0%	25%	0%	25%	0%	0%	27%	0%	0%
Deep vein thrombosis	3	3	1	1	1		0%	8%	0%	0%	0%	0%	33%	67%	0%	0%	0%	0%	0%	20%	0%	0%
Embolism	1	1		1			0%	0%	4%	0%	0%	0%	0%	0%	0%	0%	0%	0%	100%	0%	0%	17%
Flushing	1	1		1			4%	0%	0%	0%	0%	100%	0%	0%	0%	0%	0%	0%	0%	7%	0%	0%
Haematoma	1	1		1			4%	0%	0%	0%	0%	0%	0%	0%	100%	0%	0%	0%	0%	7%	0%	0%
**Musculoskeletal and connective tissue disorders**	7 (29.2%)	10	1	3	5	1	13%	8%	0%	0%	0%	29%	14%	14%	14%	14%	14%	0%	0%	40%	33%	0%
Myopathy	2	2		2			4%	0%	0%	0%	0%	0%	50%	0%	50%	0%	0%	0%	0%	13%	0%	0%
Neck pain	2	3	1		1	1	4%	4%	0%	0%	0%	50%	0%	0%	0%	50%	0%	0%	0%	7%	33%	0%
Back pain	1	1			1		0%	4%	0%	0%	0%	100%	0%	0%	0%	0%	0%	0%	0%	7%	0%	0%
Bursitis	1	2			2		4%	0%	0%	0%	0%	0%	0%	100%	0%	0%	0%	0%	0%	7%	0%	0%
Joint swelling	1	1		1			4%	0%	0%	0%	0%	0%	100%	0%	0%	0%	0%	0%	0%	7%	0%	0%
Musculoskeletal pain	1	1			1		0%	0%	0%	0%	0%	0%	0%	0%	0%	0%	100%	0%	0%	7%	0%	0%
**Injury, poisoning and procedural complications**	6 (25.0%)	6	1	1	3	1	8%	8%	0%	4%	0%	33%	0%	17%	17%	17%	17%	0%	0%	33%	33%	0%
Fall	3	3	1		1	1	4%	4%	0%	0%	0%	33%	0%	33%	33%	0%	0%	0%	0%	20%	0%	0%
Contusion	1	1			1		4%	0%	0%	0%	0%	0%	0%	0%	0%	100%	0%	0%	0%	0%	33%	0%
Procedural complication	1	1			1		0%	4%	0%	0%	0%	100%	0%	0%	0%	0%	0%	0%	0%	7%	0%	0%
Transplant failure	1	1		1			0%	0%	0%	4%	0%	0%	0%	0%	0%	0%	100%	0%	0%	7%	0%	0%
**Renal and urinary disorders**	6 (25.0%)	6	2	3	1		21%	4%	0%	0%	0%	17%	17%	17%	17%	0%	0%	17%	17%	27%	0%	33%
Dysuria	2	2	1	1			4%	4%	0%	0%	0%	0%	0%	0%	50%	0%	0%	0%	50%	7%	0%	17%
Pollakiuria	2	2	1	1			8%	0%	0%	0%	0%	50%	0%	0%	0%	0%	0%	50%	0%	7%	0%	17%
Haemoglobinuria	1	1			1		4%	0%	0%	0%	0%	0%	100%	0%	0%	0%	0%	0%	0%	7%	0%	0%
Micturition urgency	1	1		1			4%	0%	0%	0%	0%	0%	0%	100%	0%	0%	0%	0%	0%	7%	0%	0%
**Psychiatric disorders**	5 (20.8%)	6	1	2	2	1	13%	4%	4%	0%	0%	20%	0%	0%	0%	40%	0%	40%	0%	7%	67%	33%
Depression	2	2	1		1		8%	0%	0%	0%	0%	50%	0%	0%	0%	0%	0%	50%	0%	7%	0%	17%
Insomnia	2	2		1	1		4%	0%	4%	0%	0%	0%	0%	0%	0%	50%	0%	50%	0%	0%	33%	17%
Agitation	1	1		1			0%	4%	0%	0%	0%	0%	0%	0%	0%	100%	0%	0%	0%	0%	33%	0%
Depressed mood	1	1				1	0%	0%	0%	0%	0%	0%	0%	0%	0%	100%	0%	0%	0%	0%	33%	0%
**Ear and labyrinth disorders**	4 (16.7%)	4	1	3			13%	0%	0%	0%	0%	25%	25%	0%	0%	25%	25%	0%	0%	20%	33%	0%
Tinnitus	3	3		3			13%	0%	0%	0%	0%	33%	33%	0%	0%	0%	33%	0%	0%	20%	0%	0%
Hypoacusis	1	1	1				0%	0%	0%	0%	0%	0%	0%	0%	0%	100%	0%	0%	0%	0%	33%	0%
**Endocrine disorders**	4 (16.7%)	4	2	1		1	4%	8%	0%	0%	0%	25%	0%	50%	0%	25%	0%	0%	0%	20%	33%	0%
Hypothyroidism	2	2	2				0%	8%	0%	0%	0%	0%	0%	50%	0%	50%	0%	0%	0%	7%	33%	0%
Glucocorticoid deficiency	1	1				1	0%	0%	0%	0%	0%	0%	0%	100%	0%	0%	0%	0%	0%	7%	0%	0%
Hyperthyroidism	1	1		1			4%	0%	0%	0%	0%	100%	0%	0%	0%	0%	0%	0%	0%	7%	0%	0%
**Neoplasms benign, malignant and unspecified (incl cysts and polyps)**	3 (12.5%)	3			3		-	-	-	-	-	33%	67%	0%	0%	0%	0%	0%	0%	20%	0%	0%
Tumour haemorrhage	3	3			3		-	-	-	-	-	33%	67%	0%	0%	0%	0%	0%	0%	20%	0%	0%
**Cardiac disorders**	2 (8.3%)	3	1		2		4%	4%	0%	0%	4%	0%	0%	50%	0%	50%	0%	0%	0%	7%	33%	0%
Bradycardia	1	1	1				4%	0%	0%	0%	0%	0%	0%	100%	0%	0%	0%	0%	0%	7%	0%	0%
Intracardiac thrombus	1	1			1		0%	0%	0%	0%	4%	0%	0%	0%	0%	100%	0%	0%	0%	0%	33%	0%
Myocardial fibrosis	1	1			1		0%	4%	0%	0%	0%	0%	0%	0%	0%	100%	0%	0%	0%	0%	33%	0%
**Immune system disorders**	1 (4.2%)	1				1	-	-	-	-	-	0%	0%	0%	0%	0%	100%	0%	0%	7%	0%	0%
Allergy to arthropod bite	1	1				1						0%	0%	0%	0%	0%	100%	0%	0%	7%	0%	0%

Notes: see page 1

Supplementary Table 7

Neurologic Assessment in Neuro-Oncology (NANO scale) until day +90 (doi: 10.1093/neuonc/nox029)

GAIT

Study Patient-ID	Screening	BASELINE	FU +30	FU +60	FU +90
TEM-G6	0	0	0	0	0
TEM-G7	0	0	0	0	0
TEM-G8	0	0	0	NA	0
TEM-G9	0	0	0	0	NA
TEM-G11	0	0	0	0	0
TEM-G13	0	0	0	0	0
TEM-G14	0	2	2	2	3
TEM-G15	0	1	NE	3	NA
TEM-G16	0	1	2	2	2
TEM-G18	0	0	0	0	0
TEM-G19	0	0	0	1	0
TEM-G20	0	0	0	0	0
TEM-G21	0	0	0	0	0
TEM-G22	0	0	0	0	0
TEM-G23	0	0	NE	NA	3
TEM-G26	0	0	0	0	0
TEM-G28	1	1	2	NA	NA
TEM-G29	0	0	0	0	0
TEM-G30	0	0	0	0	0
TEM-G31	1	2	2	2	1
TEM-G32	0	0	0	0	0
TEM-G34	0	1	1	1	1
TEM-G37	0	0	0	0	0
TEM-G39	0	0	0	NA	NA

0: normal; 1: walks w/o assistance; 2: with assistance; 3: unable to walk

STRENGHT

Study Patient-ID	Screening	BASELINE	FU +30	FU +60	FU +90
TEM-G6	0	0	0	0	0
TEM-G7	0	0	0	0	0
TEM-G8	0	0	0	0	0
TEM-G9	0	0	0	1	NA
TEM-G11	0	0	0	1	1
TEM-G13	0	0	0	1	0
TEM-G14	0	1	2	1	2
TEM-G15	0	0	2	2	NA
TEM-G16	0	1	1	0	0
TEM-G18	0	0	0	0	0
TEM-G19	0	0	0	1	0
TEM-G20	0	0	0	0	0
TEM-G21	0	0	0	0	0
TEM-G22	0	0	0	0	0
TEM-G23	0	0	2	NA	2
TEM-G26	0	0	0	0	0
TEM-G28	1	1	0	NA	NA
TEM-G29	0	0	0	0	0
TEM-G30	0	0	0	0	0
TEM-G31	0	0	0	1	1
TEM-G32	0	0	0	0	0
TEM-G34	0	1	1	1	0
TEM-G37	0	0	0	0	0
TEM-G39	0	0	0	NA	NA

0: normal; 1: decreased against resistance; 2: none against resistance; 3: none

ATAXIA

Study Patient-ID	Screening	BASELINE	FU +30	FU +60	FU +90
TEM-G6	0	0	0	0	0
TEM-G7	0	0	0	0	0
TEM-G8	0	0	0	0	0
TEM-G9	0	0	0	0	0
TEM-G11	0	0	0	0	0
TEM-G13	0	0	0	0	0
TEM-G14	0	0	1	NE	NE
TEM-G15	0	0	1	0	NA
TEM-G16	0	0	0	1	1
TEM-G18	0	0	0	0	0
TEM-G19	0	0	0	1	0
TEM-G20	0	0	0	0	0
TEM-G21	0	0	0	0	0
TEM-G22	0	0	0	0	0
TEM-G23	0	1	NE	NA	1
TEM-G26	0	0	0	0	0
TEM-G28	0	1	0	NA	NA
TEM-G29	0	0	0	0	0
TEM-G30	0	0	0	0	0
TEM-G31	0	0	0	0	1
TEM-G32	0	0	0	0	0
TEM-G34	1	1	1	1	1
TEM-G37	0	0	0	0	0
TEM-G39	0	0	0	NA	NA

0: Finger to nose w/o difficulty; 1: with difficulty; 2: unable

SENSATION

Study Patient-ID	Screening	BASELINE	FU +30	FU +60	FU +90
TEM-G6	0	0	0	0	0
TEM-G7	0	0	0	0	0
TEM-G8	0	0	0	0	0
TEM-G9	0	0	0	0	0
TEM-G11	1	0	0	1	1
TEM-G13	0	0	0	0	0
TEM-G14	0	1	1	1	1
TEM-G15	0	0	0	0	NA
TEM-G16	0	0	0	0	0
TEM-G18	0	0	0	0	0
TEM-G19	0	0	0	1	0
TEM-G20	0	0	0	0	0
TEM-G21	0	0	0	0	0
TEM-G22	0	0	0	0	0
TEM-G23	0	0	0	NA	1
TEM-G26	0	0	0	0	0
TEM-G28	0	0	0	NA	NA
TEM-G29	0	0	0	0	0
TEM-G30	0	0	0	0	0
TEM-G31	0	0	0	0	0
TEM-G32	0	0	0	0	0
TEM-G34	1	0	0	0	0
TEM-G37	0	0	0	0	0
TEM-G39	0	0	0	NA	NA

0: normal; 1: decreased but aware; 2: unaware

VISUAL FIELDS

Study Patient-ID	Screening	BASELINE	FU +30	FU +60	FU +90
TEM-G6	0	0	0	0	0
TEM-G7	0	0	0	0	0
TEM-G8	1	1	1	NA	1
TEM-G9	0	0	0	0	0
TEM-G11	0	0	0	0	0
TEM-G13	0	0	0	0	0
TEM-G14	0	0	0	0	0
TEM-G15	0	0	0	0	NA
TEM-G16	0	0	0	0	0
TEM-G18	1	1	1	1	1
TEM-G19	0	0	0	1	1
TEM-G20	2	2	2	2	2
TEM-G21	0	0	0	0	0
TEM-G22	3	0	2	2	2
TEM-G23	2	0	0	NA	3
TEM-G26	0	0	0	0	0
TEM-G28	0	0	0	NA	NA
TEM-G29	0	0	0	0	0
TEM-G30	0	0	0	0	0
TEM-G31	0	0	0	2	2
TEM-G32	0	0	0	0	0
TEM-G34	0	0	0	0	0
TEM-G37	0	0	0	0	0
TEM-G39	0	0	0	NA	NA

0: normal; 1: partial hemianopsia, inconsistent; 2: consistent; 3: complete hemianopsia

FACIAL STRENGTH

Study Patient-ID	Screening	BASELINE	FU +30	FU +60	FU +90
TEM-G6	0	0	0	0	0
TEM-G7	0	0	0	0	0
TEM-G8	0	0	0	0	0
TEM-G9	0	0	0	0	0
TEM-G11	0	0	0	0	0
TEM-G13	0	1	0	0	0
TEM-G14	0	1	1	1	0
TEM-G15	0	0	1	1	NA
TEM-G16	0	0	1	0	0
TEM-G18	0	0	0	0	0
TEM-G19	0	0	0	1	0
TEM-G20	0	0	0	0	0
TEM-G21	0	0	0	0	0
TEM-G22	0	0	0	0	0
TEM-G23	0	0	0	NA	0
TEM-G26	0	0	0	0	0
TEM-G28	0	0	0	NA	NA
TEM-G29	0	0	0	0	0
TEM-G30	0	0	0	0	0
TEM-G31	1	1	1	0	0
TEM-G32	0	0	0	0	0
TEM-G34	0	0	0	0	0
TEM-G37	0	0	0	0	0
TEM-G39	0	0	0	NA	NA

0: normal; 1: mild/moderate weakness; 3: severe weakness

LANGUAGE

Study Patient-ID	Screening	BASELINE	FU +30	FU +60	FU +90
TEM-G6	1	1	1	1	1
TEM-G7	0	0	0	0	0
TEM-G8	1	1	0	NA	1
TEM-G9	0	0	0	0	0
TEM-G11	0	0	0	0	0
TEM-G13	0	0	0	0	0
TEM-G14	0	0	0	0	NA
TEM-G15	0	0	1	0	NA
TEM-G16	0	0	0	0	0
TEM-G18	1	2	1	1	1
TEM-G19	1	1	1	0	0
TEM-G20	0	0	0	0	0
TEM-G21	0	1	1	0	0
TEM-G22	0	1	0	0	0
TEM-G23	0	0	0	NA	1
TEM-G26	1	0	0	0	1
TEM-G28	0	0	0	NA	NA
TEM-G29	0	0	0	0	0
TEM-G30	0	0	0	0	0
TEM-G31	0	0	0	0	1
TEM-G32	1	0	0	1	1
TEM-G34	0	0	0	0	0
TEM-G37	0	0	0	0	0
TEM-G39	0	0	0	NA	NA

0: normal; 1: abnormal, meaning conveyed easily; 2: with difficulty; 3: not conveyed

LEVEL OF CONSCIOUSNESS

Study Patient-ID	Screening	BASELINE	FU +30	FU +60	FU +90
TEM-G6	0	0	0	0	0
TEM-G7	0	0	0	0	0
TEM-G8	0	0	0	0	0
TEM-G9	0	0	0	0	0
TEM-G11	0	0	0	0	0
TEM-G13	0	0	0	0	0
TEM-G14	0	0	0	0	0
TEM-G15	0	0	1	1	NA
TEM-G16	0	1	0	0	0
TEM-G18	0	0	0	0	1
TEM-G19	0	0	0	1	0
TEM-G20	0	0	0	0	0
TEM-G21	0	0	0	0	0
TEM-G22	0	0	0	0	0
TEM-G23	0	0	0	NA	1
TEM-G26	0	0	0	0	0
TEM-G28	0	0	0	NA	NA
TEM-G29	0	0	0	0	0
TEM-G30	0	0	0	0	0
TEM-G31	0	0	0	0	0
TEM-G32	0	0	0	0	0
TEM-G34	0	0	0	0	0
TEM-G37	0	0	0	0	0
TEM-G39	0	0	0	NA	NA

0: normal; 1: drowsy; 3: somnolent; 4: unarousable/comatese

BEHAVIOUR

Study Patient-ID	Screening	BASELINE	FU +30	FU +60	FU +90
TEM-G6	0	1	0	0	0
TEM-G7	0	0	0	0	0
TEM-G8	0	0	0	0	0
TEM-G9	0	0	0	0	0
TEM-G11	0	0	0	0	0
TEM-G13	0	0	0	0	0
TEM-G14	0	0	0	0	0
TEM-G15	1	0	1	0	NA
TEM-G16	0	0	0	0	0
TEM-G18	0	0	0	0	0
TEM-G19	0	0	1	0	0
TEM-G20	0	0	0	0	0
TEM-G21	0	0	0	0	0
TEM-G22	0	0	0	0	0
TEM-G23	0	0	0	NA	1
TEM-G26	0	0	0	0	0
TEM-G28	0	0	0	NA	NA
TEM-G29	0	0	0	0	0
TEM-G30	0	0	0	0	0
TEM-G31	1	1	1	1	0
TEM-G32	0	0	0	0	0
TEM-G34	0	0	0	0	0
TEM-G37	0	0	0	0	0
TEM-G39	0	0	0	NA	NA

0: normal; 1: mild/moderate alteration; 2: severe alteration

NE: not evaluable; NA: not available

Supplementary Table 8: Characteristics of recurrence samples from control patients

Internal control cohort for CD34 immunohistochemistry staining (related to **Fig. 4B**):

**Patient code**	**Sex**	**Age** (years)	**Tumor location**	**MGMT***	**First line treatment**	**Second line**	**Time from diagnosis to 2nd surgery**
BS-#2	M	51	Right parietal	NM	Exeresis+RT/CT	-	4 months
BS-#3	M	65	Left fronto-parietal	NM	Exeresis+RT/CT	TMZ (6 cycle)	16 months
BS-#5	F	39	Fronto-parietal	NM	Exeresis+RT/CT	-	5 months
BS-#8	M	68	Left fronto-parietal	NM	Exeresis+RT/CT	-	8 months
BS-#9	M	50	Temporo-parietal	NM	Exeresis+RT/CT	TMZ (2 cycle)	12 months
BS-#10	M	55	Left frontal	NM	Exeresis+RT/CT	-	15 months
BS-#11	F	54	Left temporo-parietal	NM	Exeresis+RT/CT	TMZ (3 cycle)	17 months
BS-#12	M	55	Right fronto-parietal	NM	Exeresis+RT/CT	-	11 months

* NM: non-methylated MGMT promoter; RT/CT: adjuvant radiotherapy/chemotherapy according to the Stupp regimen; TMZ: Temozolomide

Internal control cohort for single cell RNA sequencing/ single cell TCR sequencing (related to **Fig. 4; Extended Data Fig. 6**; Supplementary Figure 4;):

**Patient code**	**Sex**	**Age** (years)	**Tumor location**	**MGMT***	**First line treatment**	**Second line**	**Time from diagnosis to 2nd surgery**
MI-G1	M	55	Right frontal	M	Exeresis+RT/CT	TMZ (1 cycle)	39 months
MI-G2	F	52	Right frontal	M/NM	Exeresis+RT/CT	Regorafenib	13 months
MI-G3	M	63	Right temporal	M	Exeresis+RT/CT	Regorafenib, TMZ	34 months
MI-G4	F	62	Left parieto-occipital	M	Exeresis+RT/CT	-	13 months
MI-G5	F	39	Left fronto-parietal	NM	Exeresis+RT/CT	-	9 months
MI-G6	M	83	Left frontal	NM	Exeresis+RT	-	14 months

*M: methylated; NM: non-methylated MGMT promoter; RT/CT: adjuvant radio- /chemotherapy according to the Stupp regimen; RT: radiotherapy; TMZ: Temozolomide

Matched control cohorts for single cell RNA sequencing (related to **Fig. 4D, E, G**):

Myeloid Cohort	Patient ID	Sex	Age (years)	Localisation	MGMT	Status	TTR (months)	Previous Treatments
Milano	MI-G2	F	52	R Frontal	NM	Recur	13	Ex+RT/CT+Reg
Milano	MI-G5	F	39	L FrontoPar	NM	Recur	9	Ex+RT/CT
Milano	MI-G6	M	83	L Frontal	NM	Recur	14	Ex+RT
McGill[69]	OPK369	F	74	L Temporal	NM	Recur	18	Ex+RT/CT
McGill[69]	OPK374	F	57	R Temporal	NM	Recur	20	Ex+RT/CT
McGill[69]	OPK389*(A)	M	58	R Parietal	NM	Recur	7	Ex+RT/CT
McGill[69]	OPK408	F	53	R Temporal	NM	Recur	7	Ex+RT/CT
McGill[69]	OPK419	M	74	L Temporal	NM	Recur	11	Ex+RT/CT
McGill[69]	OPK426	M	55	L Temporal	NM	Recur	7	Ex+RT/CT
Brussels[10]	R4	M	64	NA	NM	Recur	NA	Ex+RT/CT
**Controls**	**Median**	**5M/ 5F**	**57.5**		**10 NM**	**Recur**	**11.0**	**Ex+RT/CT**
**Temferon**	**Median (range)**	**6M**	**53 (29-61)**		**6 NM**	**Recur**	**11.3 (7-17)**	**Ex+RT/hdCT/TEM**

Lymphoid Cohort	Patient ID	Sex	Age (years)	Localisation	MGMT	Status	TTR (months)	Previous Treatments
Milano	MI-G2	F	52	R Frontal	NM	Recur	13	Ex+RT/CT+Reg
Milano	MI-G5	NA	NA	NA	NM	Recur	9	Ex+RT/CT
Milano	MI-G6	M	83	L Frontal	NM	Recur	14	Ex+RT
McGill[69]	OPK374	F	57	R Temporal	NM	Recur	20	Ex+RT/CT
McGill[69]	OPK389*(A)	M	58	R Parietal	NM	Recur	7	Ex+RT/CT
McGill[69]	OPK408	F	53	R Temporal	NM	Recur	7	Ex+RT/CT
McGill[69]	OPK419	M	74	L Temporal	NM	Recur	11	Ex+RT/CT
Brussels[10]	R4	M	64	NA	NM	Recur	NA	Ex+RT/CT
BWH[68]	E10	M	58	NA	NM	Recur	NA	Ex+RT/CT
BWH[68]	E100	M	33	NA	NM	Recur	NA	Ex+RT/CT+Opt
BJH[47]	GBM104/Re	M	33	NA	NM	Recur	NA	NA
UCLA[70]	LB3579T	NA	NA	NA	NM	Recur	NA	NA
UCLA[70]	LB3871T	NA	NA	NA	NM	Recur	NA	NA
UCLA[70]	LB4059T	NA	NA	NA	NM	Recur	NA	NA
**Controls**	**Median**	7M /3F	57.5		**14 NM**	**Recur**		
**Temferon**	**Median (range)**	**6M**	**53 (29-61)**		**6 NM**	**Recur**	**11.3 (7-17)**	**Ex+RT/hdCT/TEM**

Inclusion criteria: Tissue sample from recurrent $IDH^{WT}MGMT^{unmeth}$ GBM; immunotherapy-naïve; publicly available scRNAseq data run on the 10x Genomics platform. Source data, see reference list. F: female; M: male; NA: information not available; NM: non-methylated MGMT promoter; TTR: time to recurrence calculated from first surgery; Ex: Exeresis; RT/CT: adjuvant radio-/chemotherapy according to the Stupp regimen; RT: radiotherapy; hdCT: high dose ; TEM: Temferon; TMZ: Temozolomide; Reg: Regorafenib; Opt: Optune Tumor Treating Fields.

Supplementary Table 9: Summary of clinical trial protocol amendment version history (TEM-GBM_001)

Protocol version	Date issued	Purpose of modification
1.2	29 October 2018	• To modify the contact information for safety reporting (from Product Life Group in the UK to seQure, a specialized pharmacovigilance provider based in Italy).<br>• To reorganize the schedule of events in order to clarify the assessments being performed at the clinical centers involved in the study<br>• To correct typographical errors identified in the protocol<br>• Corrections have been made to the immunophenotype panels which had been incorrectly listed<br>• Greater clarity is provided on the assessments to be performed
2.0	05 February 2019	• Omission of temozolomide from the treatment regimen during the 6 weeks of radiotherapy<br>• Maintain blood platelet concentrations of at least 50 x 10^3/mm^3 during the hospitalization period post Temferon administration<br>• Alignment with approved changes to methods and procedures included in a parallel Temferon study in patients with multiple myeloma (EudraCT 2018-001741-14)<br>• To correct typographical errors identified in the protocol
3.0	27 April 2020	• Instructions on the follow up of patients during the COVID-19 pandemic<br>• Clarification on the required interval between patients in each cohort in Part A<br>• Instruct that peripheral blood be collected for the measurement of VCN at the time of hematological engraftment<br>• Clarify that dose adjustments may use data based on VCN expressed to 2 decimal places<br>• Permit the assessment of IFN-α concentrations and VCN in peripheral blood in the event of Serious Adverse Events, should these be clinically indicated<br>• Clarification on the routine analysis to be performed on cerebrospinal fluid and CSF collection at time of second surgery<br>• To correct typographical errors identified in the protocol and schedule of events
4.0	17 July 2020	• Recruitment and management of study patients in three centers in Italy (Ospedale San Raffaele, Istituto Neurologico Carlo Besta, Policlinico Universitario Fondazione Agostino Gemelli)<br>• Addition of 2 cohorts to Part A in order to evaluate the safety and hematologic engraftment associated with two conditioning regimens (BCNU and thiotepa, or busulfan and thiotepa) when

		administered prior to intravenous infusion of 2 x 10^6 non-modified $CD34^+$ cells/kg and Temferon Dose Level 3<br>• Exclusion at Screening from the study of patients with a known hypersensitivity to busulfan<br>• Permit additional leukapheresis to be performed after radiotherapy is completed in order to harvest sufficient non-modified $CD34^+$ cells to be used for administration prior to Temferon or as a backup
4.1	14 December 2020	• Prof Gaetano Finocchiaro has been appointed as Coordinating Principal Investigator based at Ospedale San Raffaele, Milan<br>• Dr. Marica Eoli has been appointed as the new Principal Investigator for Istituto Neurologico Carlo Besta<br>• Change of the company name of the contract manufacturing organization (CMO) selected for the production of the drug product
4.2	05 July 2021	• Change of the Medical Monitor<br>• Addition of 2 cohorts to Part A in order to continue dose escalation to Dose Level 4 (Cohort 6) and Dose Level 5 (Cohort 7), while administering the conditioning regimen as in Cohorts 1-4 (BCNU and thiotepa) and intravenous infusion of 2 x 106 non-modified $CD34^+$ cells/kg and Temferon Dose Level 4
5.0	21 October 2022	• Two cohorts to use busulfan monotherapy, each of 3 patients, in Part A. Both cohorts will utilize the same conditioning regimen of busulfan and the infusion of 3 x 10^6 non-modified CD34+ cells/kg. Patients in Cohort 7 will receive 3 x 10^6 transduced cells/kg, while patients in Cohort 8 will receive 4 x 10^6 transduced cells/kg. Following completion of the DMC review of data from both Cohorts 7 and 8, the DMC will advise on dose escalation (after Cohort 7) or dosing of an additional 3 subjects.

For more details about the implemented changes, please refer to paragraph 12.2 “Summary of Prior Protocol Amendments” of the provided study protocol TEM-GBM_001 (version amendment 5.0, 21 October 2022).

Supplementary Figure 1

A

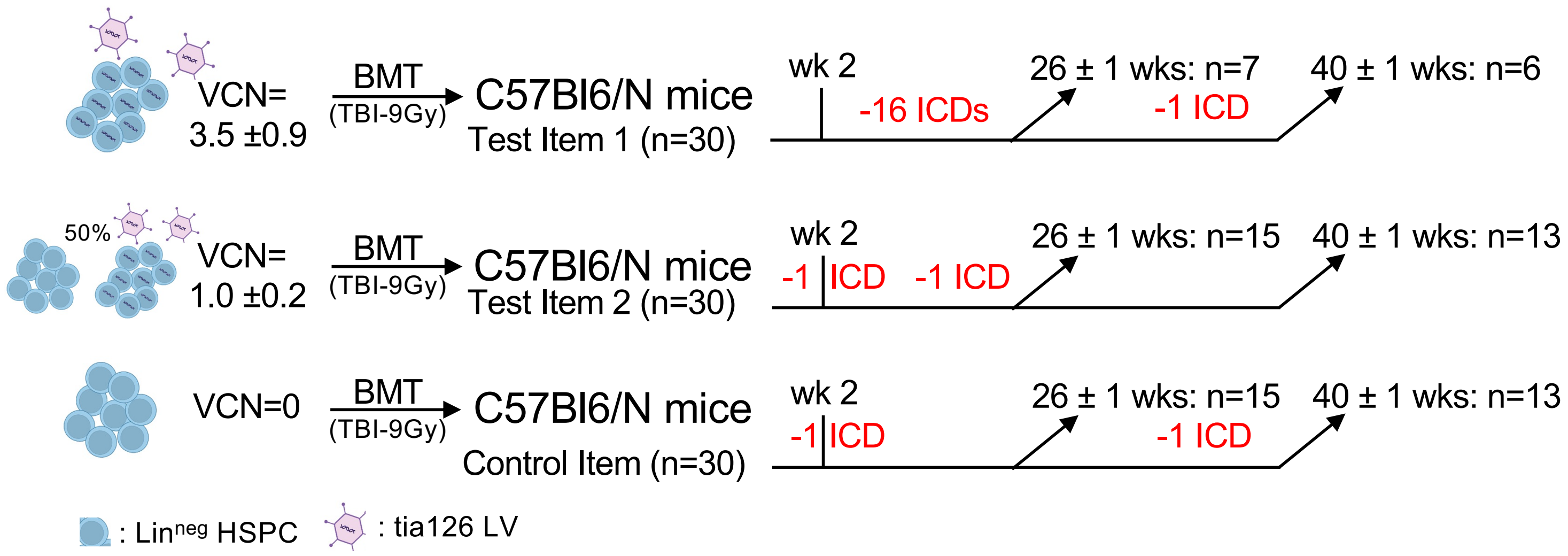

B

Timing of ICD	Cause of death	TI-1	TI-2	CI
Very early ICD (week 1-2)	irradiation toxicity	-	1 (3.3%)	1 (3.3%)
Early ICD (week 2-5)	engraftment failure	14 (46.7%)	-	-
Pathological findings:	*- moderate to severe decreased BM cellularity*			
	- lymphocyte depletion in the thymus and spleen			
	- focal/multifocal haemorrhage in the brain and/or heart			
Late ICD (>week 18)	various	3 (10%)	1 (3.3%)	1 (3.3%)
Pathological findings:	*- lymphoblastic lymphoma*			
	- spontaneous dental malocclusion			
	- skin/head inflammation			

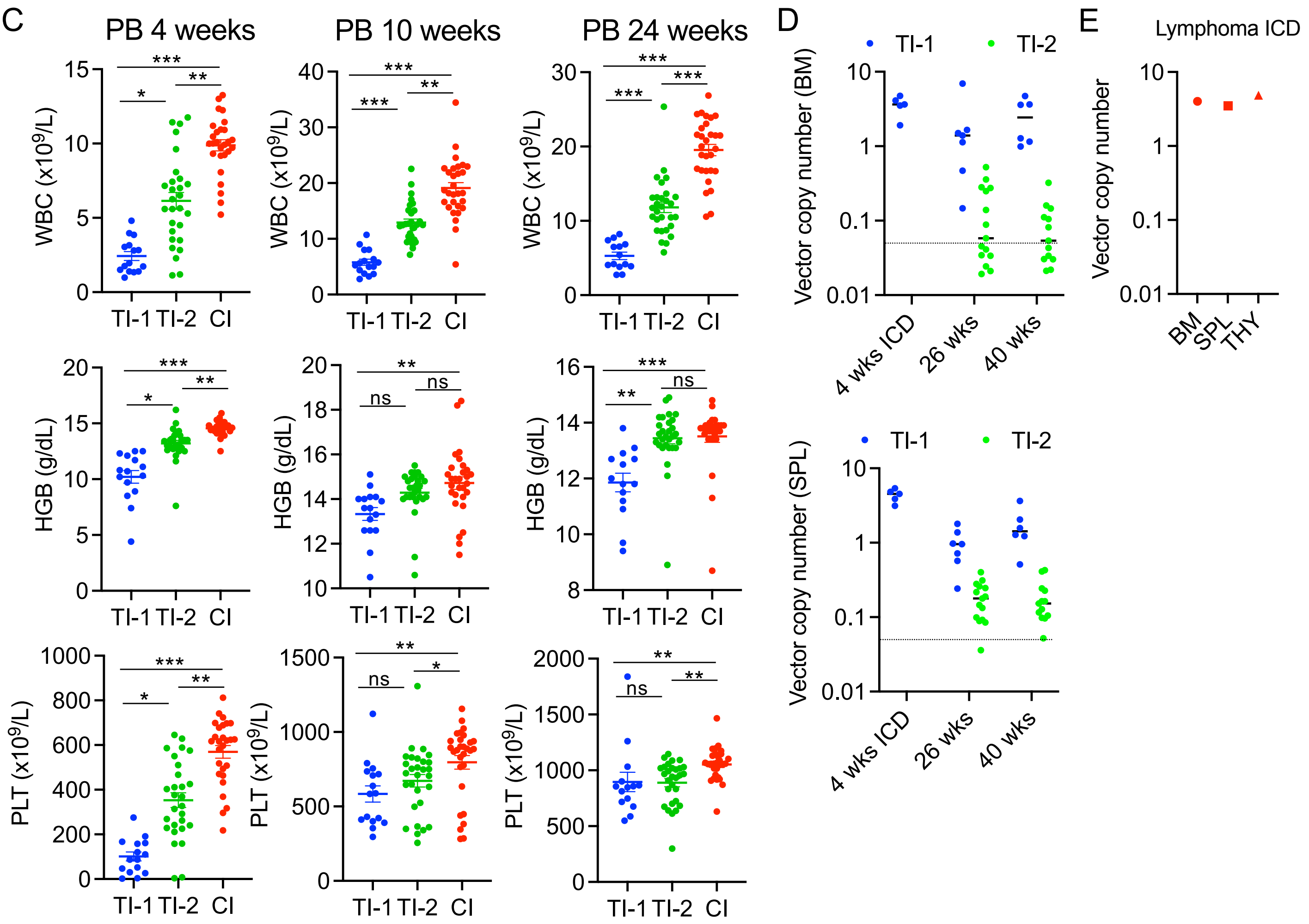

Supplementary Figure 1 | Toxicity studies in pre-clinical mouse models.

To evaluate the potential toxicity and tumorigenicity of Temferon, we transplanted C57/Bl6 mice with lineage marker negative bone marrow cells (Lin^{neg} HSPC) transduced with the murine version of the lentiviral construct (tia126 LV, multiplicity of infection 35) expressing Ifnα under the control of the Tie2 promoter/enhancer and miR-126, or mock-transduced, and followed them for 26 to 40 weeks. Mice were housed at 20-24°C, 40-70% humidity and 12hrs dark/light cycle. The study was conducted in compliance with Italian GLP regulations and OECD GLP principles. The study complied with the EMEA/CHMP/GTWP/125459/2006 guideline and was approved by the Animal Care and Use Committee and Ethical Board of Ospedale San Raffaele (IACUC 836) and notified to the national authorities in accordance with applicable Italian law.

(**A**) Experimental scheme. Bone marrow transplantation (BMT) with tia126 LV-transduced (Test item 1, TI-1), mock-transduced (Control Item, CI) or a 50:50 mixture of both cell populations (TI-2) was done into 6-9 weeks old, isogenic, female C57Bl6/N (CD45.1) mice (Charles River) at a dose of 1×10^6 cells per mouse following lethal conditioning with total body irradiation (TBI 900 cGy). VCN indicates the vector copy number of the 3 transplanted cell therapy products after 14 days of culture. Mice were analyzed at a predefined intermediate (26 weeks) and late (40 weeks) endpoint. ICD, intercurrent deaths. Readouts included mortality (from day +15), clinical observation, weekly body weight, hematology (weeks 4, 10, 24, 38), engraftment (flow cytometry and VCN), analysis of intercurrent deaths, and terminal procedures including necropsy and histopathology.

While transplanting highly tia126-transduced HSPCs (mean VCN: 3.5; n=30 mice) as a standalone graft resulted in dose-limiting toxicity, mainly due to delayed or failed hematopoietic recovery, co-transplanting a mix of 50% non-transduced HSPC with 50% tia126-transduced HSPC (mean VCN post mixing: 1.0; n=30 mice) fully rescued treatment-related toxicity.

(**B**) Table describing details of registered ICDs. Long-term follow-up over 40 weeks revealed, except for a moderate decrease in white blood cell counts (mostly due to lower B lymphocytes) and platelets, no significant differences in the TI-2 group as compared to the CI group.

(**C**) Peripheral blood (PB) counts and hemoglobin level over time. Mean ± SEM, each dot represents an individual mouse (4 weeks: TI1, n=15; TI2, n=28; CI, n=27; 10 weeks: TI1, n=16; TI2, n=29; CI, n=29; 24 weeks: TI1, n=14; TI2, n=29; CI, n=29). * $p<0.05$; ** $p<0.01$; *** $p<0.001$; Kruskall-Wallis test and Dunn's post-test; ns: not significant.

(**D**) VCN in bone marrow (BM) and spleen (SPL) at the predefined endpoints or at early ICDs (where available). The line represents the median, each dot refers to an individual mouse. A statistical analysis was not pre-planned in the study protocol.

(**E**) One mouse from TI-1 developed a donor-cell derived lymphoma, whose VCN was in line with the group's median arguing for neutral gene marking rather than tia126 LV-driven insertional mutagenesis.

Taken together, pre-clinical data in mouse models suggested a therapeutic dose window for Temferon in which no significant toxicity was observed (up to 50%, this study) and anti-tumor effect could be expected (prior efficacy studies) in the GBM model.[25,26]

Supplementary Figure 2

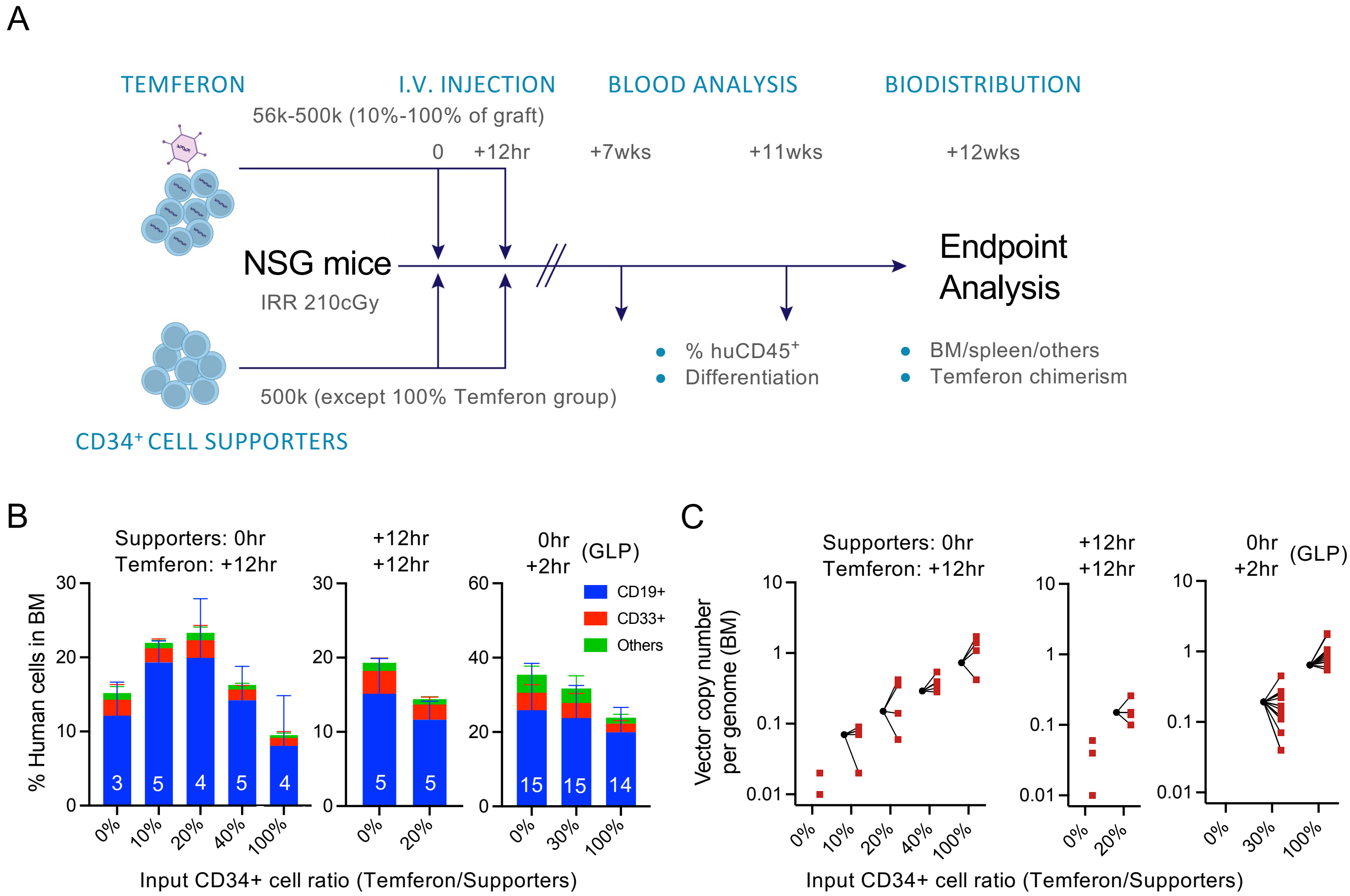

Supplementary Figure 2 | Biodistribution studies in pre-clinical mouse models.

We modeled the co-administration of TIA126 (humanized LV version)-transduced human mobilized peripheral blood HSPCs (Temferon) with non-cultured, non-transduced CD34+ HSPCs from the same donor (Supporters), either by single (mixed) or sequential injections (at 2-12hour intervals), in sub-lethally irradiated (210 cGy), 8-9 weeks old female NOD.Cg-*Prkdc*^{scid}*Il2rg*^{tm1Wjl}/SzJ (NSG) mice (Charles River Laboratories). Mice were housed at 20-24°C, 40-70% humidity and 12hrs dark/light cycle.

(**A**) Experimental scheme. First, a pilot study (internal code: R&D001_BG) was run testing a Temferon dose range from 0.56-5.0 x10^5 cells/mouse (drug product from the large scale manufacturing verification run M16102) infused concomitantly or sequentially with 5x10^5 autologous, uncultured human mobilized peripheral blood CD34+ cells/mouse (except the 100% Temferon condition). Then, a formal biodistribution study (internal code: TM042) was conducted in the SR-TIGET test facility, in compliance with the Italian Good Laboratory Practice Regulations (DL 50, 2 March 2007; G.U. 86, 13 April 2007) and the OECD Principles of Good Laboratory Practice (as revised in 1997). High (5×10^5 cells/mouse) and low (2.15×10^5 cells/mouse; sequential infusion after 5×10^5 $CD34^+$ cells, 30%) Temferon doses were compared to control mice receiving 5×10^5 autologous, uncultured $CD34^+$ cells. Human cell engraftment and biodistribution were measured by flow cytometry (humanCD45, CD19, CD33, CD3) and vector copy number analysis across multiple organs at 12 weeks, according to internal SOPs.

(**B**) Human CD45+ cell engraftment in the mouse BM at 12 weeks from transplantation, split into CD19+ B cells, CD33+ myeloid cells and others (CD45+CD19-CD33-). Shown are mean + SD, the number of mice per group is indicated in white color inside the bars. GLP, experiment performed under good laboratory practice conditions. The timing of Temferon and Supporter cell injection is indicated above each graph, the timepoint 0hr is defined as 240min from the end of total body irradiation. Human cell engraftment of both myeloid and lymphoid lineages was reduced when Temferon was administered without Supporters (100% Temferon input ratio), but it was not significantly different to the Supporter cells only group (0% ratio) when Temferon was injected in combination with Supporters. 2way ANOVA with Temferon input ratio as variable: Pilot study (left bar plot): p=0.007; (middle bar plot): p=0.07; GLP study (right bar plot): p=0.01; Dunnett's multiple comparison test: 0% vs. 30%: $p^{adjusted}$=0.5; 0% vs. 100%: $p^{adjusted}$ =0.006.

(**C**) Vector copy number (VCN) per human genomes in the infusion product (black) and in mouse BM at 12 weeks from transplantation (red). VCN was measured in the 100% Temferon product subjected to 2 weeks of in vitro culture. For the groups that received combinations of Temferon and Supporters, the input VCN of the total graft was extrapolated from the $VCN^{100\%Temferon}$ x the arithmetic proportion of Temferon within the total CD34+ cell dose. The mean *in vivo* VCN per human genome at the experimental endpoint matched the input value calculated from the *in vitro* Temferon VCN and the proportion of Temferon input to the total CD34+ cell dose, which argues against counterselection of TIA126-transduced HSPCs vs. non-cultured, non-transduced cells.

All experiments were approved by the Institutional Animal Care and Use Committee (IACUC license #749 and #836) and Ethical Board of the San Raffaele Scientific Institute, and notified to the competent national authorities.

Supplementary Figure 3

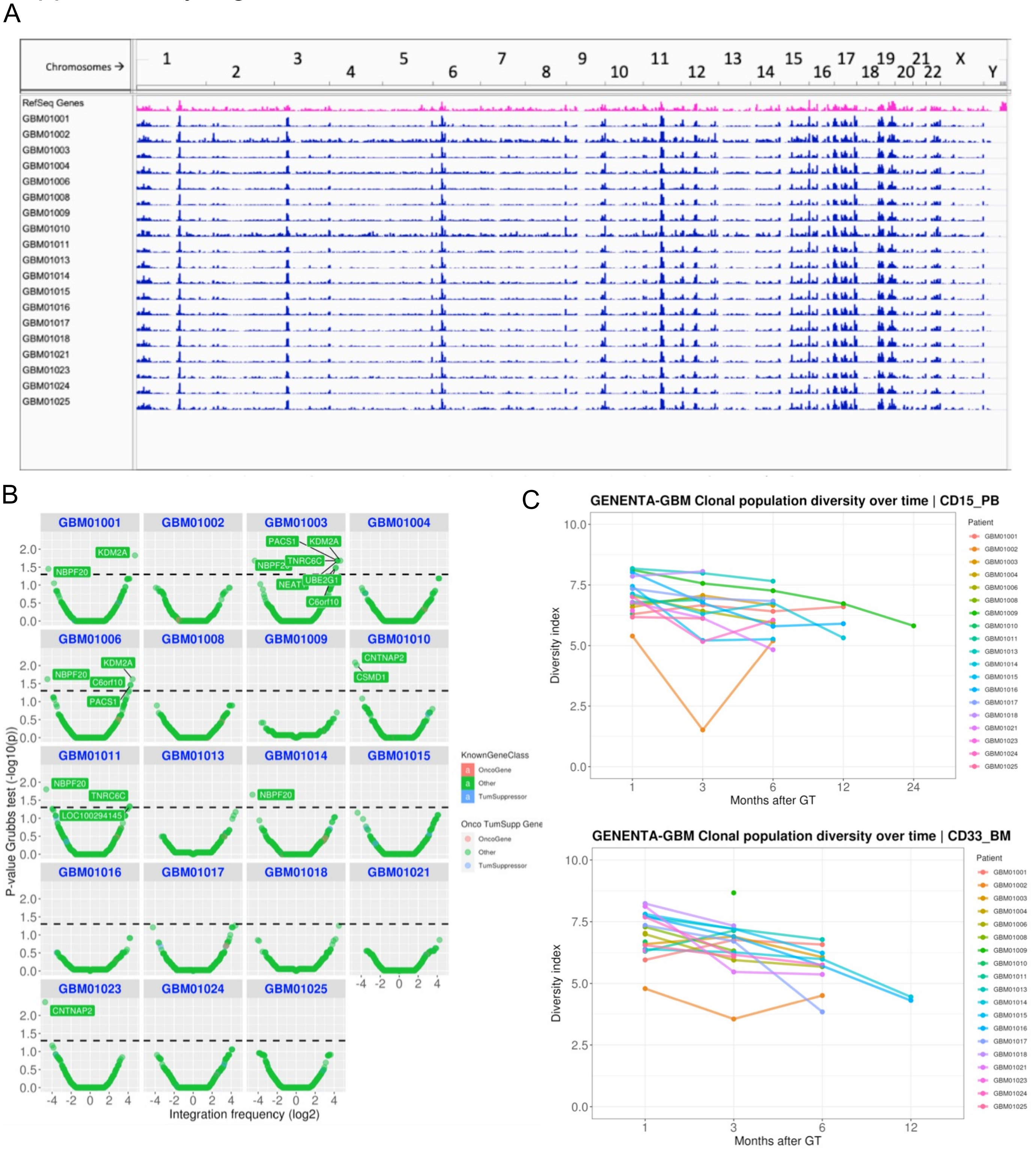

Supplementary Figure 3 | Lentiviral integration site analysis.

Lentiviral integration sites were retrieved by Sonication Linker-mediated-PCR (SLiM-PCR) from longitudinal blood and bone marrow DNA samples from patients that received Temferon.

(**A**) Distribution of lentiviral integrations sites across the human genome for each single patient. Note that the density peaks of integration sites corresponds well with gene density across the various chromosomes for all patients.

(**B**) Analysis of common integration sites. No statistically significant overrepresentation of integrations into known oncogenes or tumor suppressor genes was detected in any patient.

(**C**) Clonal diversity within CD15+ blood granulocytes (upper graph) or CD33+ bone marrow myeloid precursors (lower graph) over time, expressed as Shannon diversity index. Note that clonal diversity is decreasing over time, in line with the decreasing VCN, rather than expansion of dominant clones.

Lentiviral integration site retrieval was performed on genomic DNA using a sonication-based linker-mediated (SLiM)-PCR method, as described[61]. BSLiM-PCR products were sequenced by Illumina platform and sequences mapped and annotated by dedicated bioinformatic pipelines such as VISPA2 and ISAnalitics, as previously described[62,63]. Briefly, for each Illumina sequencing library, paired end reads undergo quality filtering, barcode identification for sample de-multiplexing, and vector sequence trimming. The remaining cellular genomic sequences are then aligned to the reference human genome (Human Genome GRCh37/hg19, Feb. 2019). To quantify the number of genomes representing each clone, we used SonicLength[64], a method that estimates clone abundance based on the count of distinct insertion site (IS) fragments. This approach ensures that each IS's abundance reflects the initial number of contributing cells, providing an estimate of clonal abundance in the original sample. The final list of unique IS consists of unambiguously mapped loci annotated with the nearest RefSeq gene.

For Common Insertion Site (CIS) analysis, we applied the Grubbs test for outliers as previously described[65]. Specifically, for each patient, we calculated the targeting frequency of each gene by counting IS within the gene body ±100 kbp, normalized by gene length. After log2 transformation of the gene frequency distribution, we performed the Grubbs test to identify genes with targeting frequencies significantly higher than the average.

Clonal diversity was assessed by the Shannon diversity index (H-index), which quantifies both species richness (number of distinct clones) and evenness (relative abundance distribution). The H-index is calculated as:

$$H' = -\sum_{i=1}^{R} p_i \ln p_i$$

i represents a clone (defined by an insertion site, IS), pi is the clonal abundance and R is the total set of clones.

Supplementary Figure 4

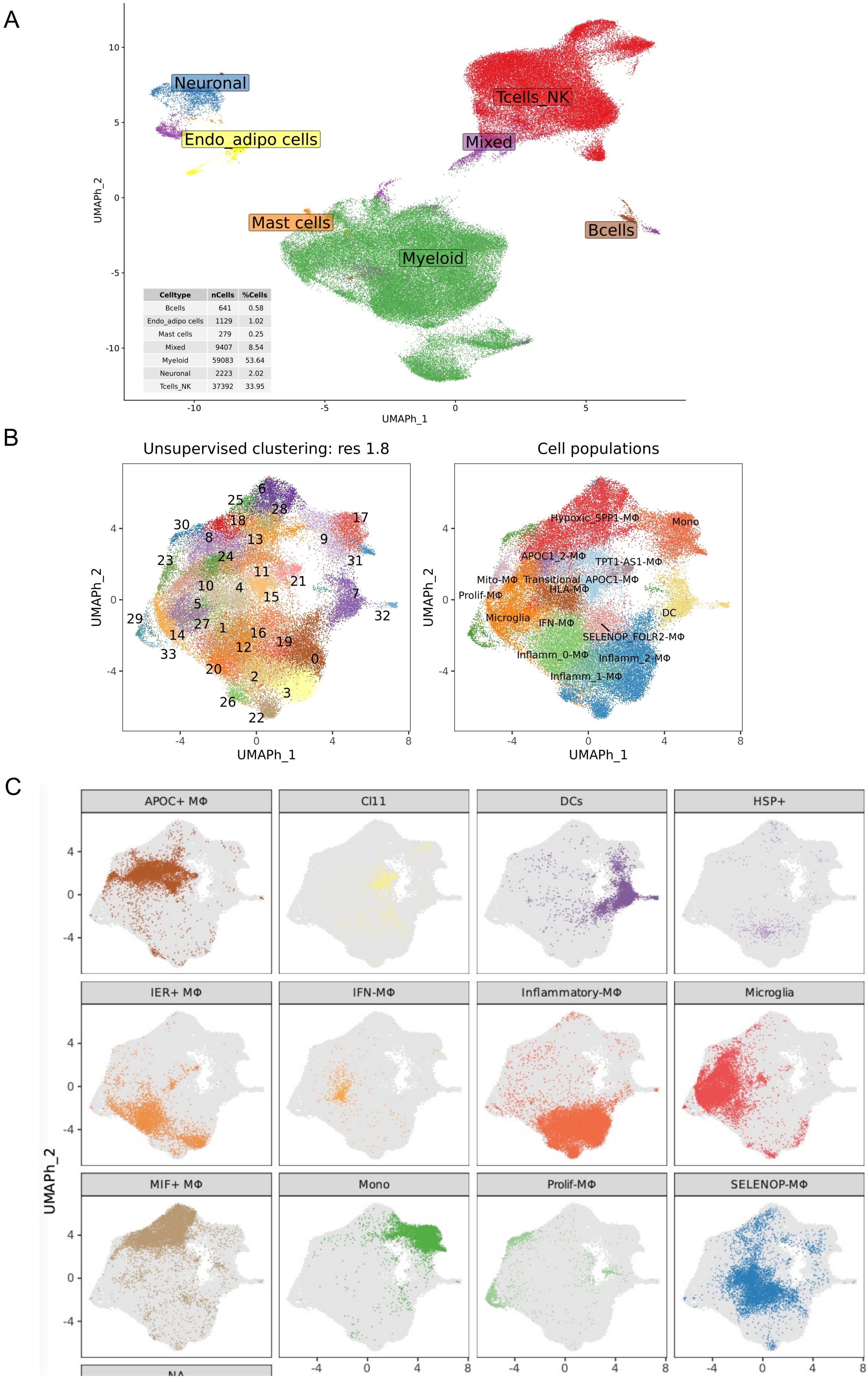

Celltype	nCells	%Cells
Bcells	641	0.58
Endo_adipo cells	1129	1.02
Mast cells	279	0.25
Mixed	9407	8.54
Myeloid	59083	53.64
Neuronal	2223	2.02
Tcells_NK	37392	33.95

Supplementary Figure 4 - *continued*

D

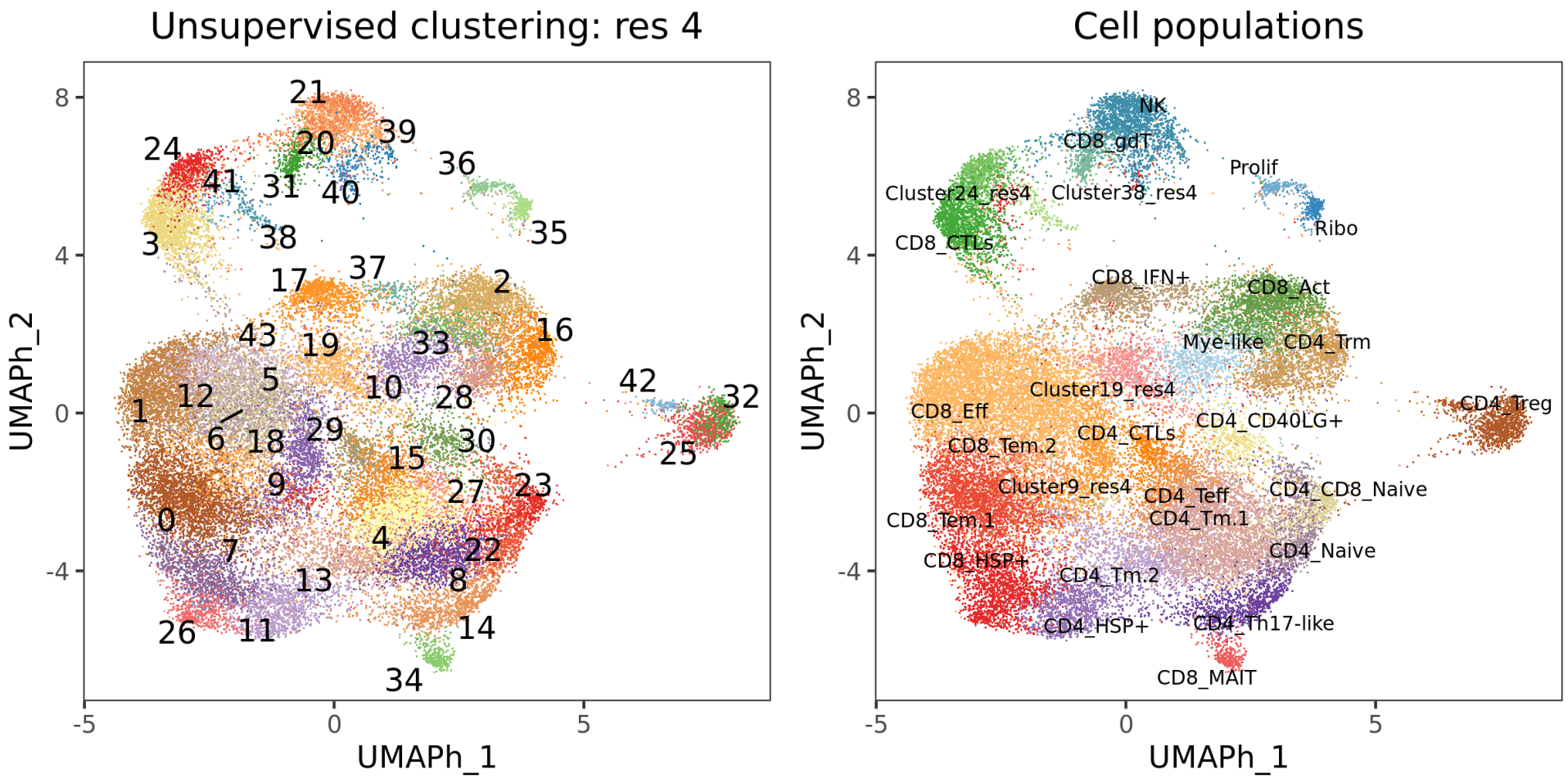

E

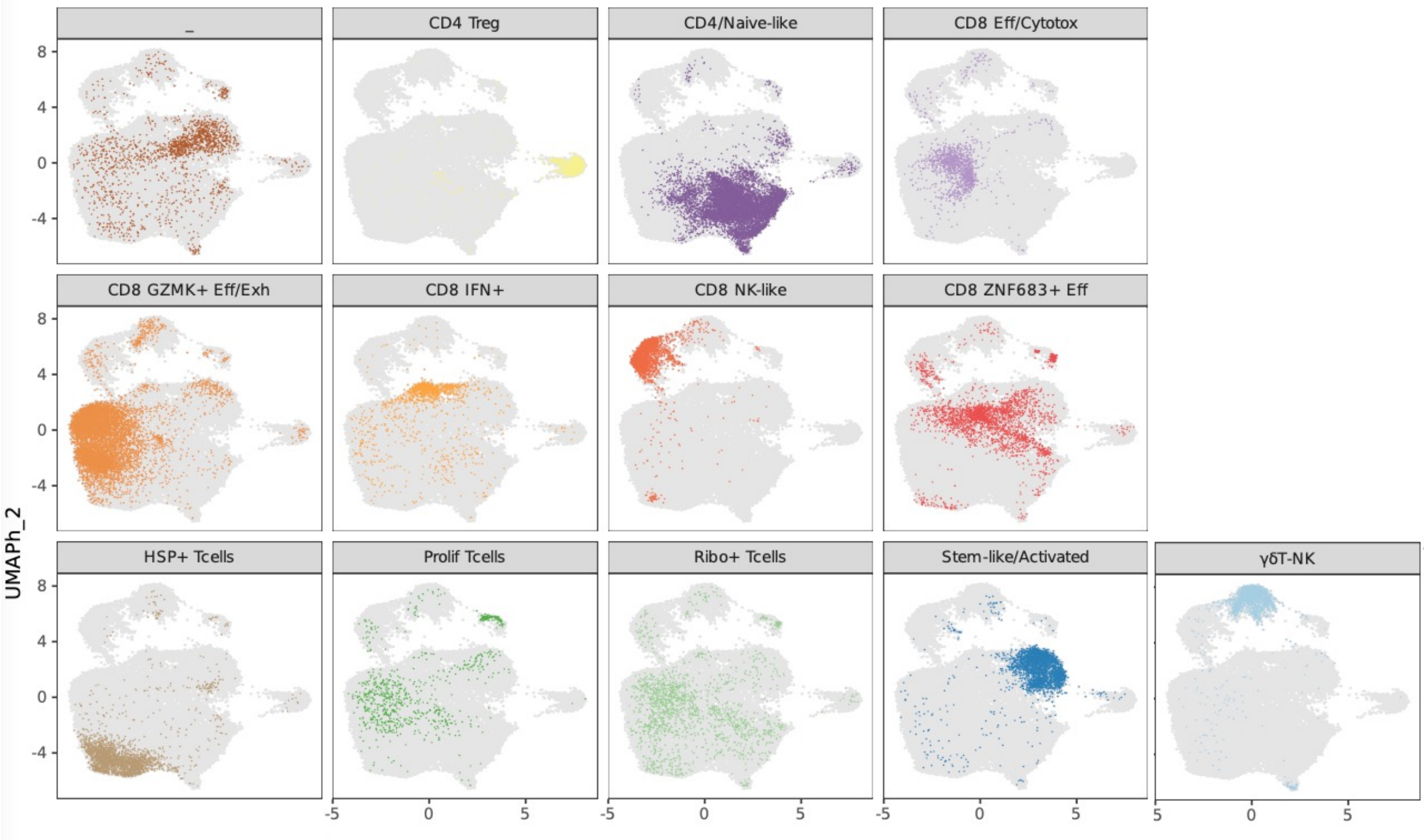

Supplementary Figure 4 | Internal scRNA-seq dataset.

Single cell RNA sequencing on GBM tumors has been performed on n=6 trial patients after Temferon infusion, as well as n=6 recurrent GBM tumors from patients treated with standard of care.

(**A**) UMAP projection of the merged object from the CD45+enriched tumor dissociates from these 12 samples (internal dataset). The insert shows the number and proportion of major cell types and lineages contained in the dataset. The 2 major components, i.e. myeloid cells (green, panels B/C) and T-/NK cells (red, panels D/E) were individually analysed in further detail.

(**B**) Unsupervised clustering (resolution: 1.8) of the tumor-associated myeloid compartment (left) and provisional classification (right) based on marker gene expression (see Supplementary Data 1).

(**C**) Projection of the refined myeloid population labels from the extended GBM single cell dataset (see Extended Data Fig. 6D-F and Supplementary Data 2) on the internal dataset.

(**D**) Unsupervised clustering at ultra-high resolution (res 4) of the tumor-associated T-/NK cell compartment (left) and provisional classification (right) based on marker gene expression (see Supplementary Data 1).

(**E**) Projection of the refined TIL population labels from the from the extended GBM single cell dataset (see Extended Data Fig. 6G,H and Supplementary Data 2) on the internal dataset.

For the myeloid dataset, we selected the top 43 principal components (PCs) and determined that resolution 1.8 was optimal to capture the dataset's heterogeneity. Manual inspection of top marker genes allowed annotation of each cluster. The annotation label was then simplified aggregating, if necessary, two or more clusters. On the final classification, we rerun marker identification using logFC threshold > 0.3 and minimal percentage of expression > 20%.

For the T cell compartment, we first removed the bias of TCR genes on cell clustering removing all TCR genes from the vector of most variable genes used for PCA dimensionality reduction. We performed clustering on the top 30 PCs, and selected a resolution of 4.0 as the optimum to describe T cell population variability. NK, CD4 and CD8 T cell subsets were assessed evaluating quantiles of $CD8A^{+}$, CD8+, $CD4^{+}$ and $CD56^{+}$ cells for each cluster at resolution 4.0. Clusters positive for both CD8s and CD4 were defined as $CD4^{+}$- $CD8^{+}$ T-cells. Details are provided in the GitLab repository.
