# Supplementary material for: Tumor-targeted interferon-α gene therapy for glioblastoma: a phase 1 trial
Source: Nat Med. 2026 Jun 1;32(6):2216–26. doi: 10.1038/s41591-026-04419-1 (PMC13768172; doi:10.1038/s41591-026-04419-1)
Supplement: Supplementary file 3 [file 41591_2026_4419_MOESM8_ESM.pdf]

DocuSign Envelope ID: A0702825-E0BD-46DB-A7D6-FEF901E11877

CLINICAL STUDY PROTOCOL

EudraCT Number 2018-001404-11

Protocol Number TEM-GBM_001

A phase I/IIa dose escalation study evaluating the safety and efficacy of autologous $CD34^+$-enriched hematopoietic progenitor cells genetically modified with a lentiviral vector encoding for the human interferon-α2 in patients with glioblastoma multiforme who have an unmethylated O-6-methylguanine-DNA methyltransferase gene promoter

The TEM-GBM_001 study will be conducted according to the protocol and in compliance with Good Clinical Practice, the ethical principles stated in the Declaration of Helsinki, and other applicable regulatory requirements.

CONFIDENTIALITY STATEMENT

Financial and nonfinancial support for this study will be provided by Genenta Science. The concepts and information contained in this document or generated during the study are considered proprietary and may not be disclosed in whole or in part without the expressed written consent of Genenta Science.

DocuSign Envelope ID: A0702825-E0BD-46DB-A7D6-FEF901E11877

**Study:**	TEM-GBM_001
**Sponsor:**	Genenta Science srl<br>via Olgettina 58<br>20132 Milano<br>Italy
**Sponsor Contact:**	[redacted]<br>Chief Medical Officer<br>Genenta Science<br>Telephone: [redacted]<br>e-mail: [redacted]
**Scientific Project Manager:**	[redacted]<br>Project Manager<br>Genenta Science<br>Telephone: [redacted]<br>e-mail: [redacted]
**Medical Monitor:**	[redacted]<br>Medical Monitor<br>Genenta Science<br>Telephone: [redacted]<br>e-mail: [redacted]
**Serious Adverse Event (SAE) Contact Information:**	Arithmos srl<br>Via Roveggia,122<br>37136 Verona ,<br>Italy<br>Email: [redacted]
**Version of Protocol:**	Amendment v5.0
**Date of Protocol:**	21st October 2022

genenta science

CONFIDENTIAL

Financial and nonfinancial support for this study will be provided by Genenta Science. Additional financial support and the investigational advanced therapy medicinal product (ATMP) will be provided by Genenta Science. The concepts and information contained in this document or generated during the study are considered proprietary and may not be disclosed in whole or in part without the expressed, written consent of Genenta Science. The study will be conducted according to the International Conference on Harmonisation harmonised tripartite guideline E6(R2): Good Clinical Practice.

Protocol Contributor	Role/ responsibility
Prof. Gaetano Finocchiaro	Coordinating Principal investigator, based at Ospedale San Raffaele, responsible for advising on the study design, eligibility criteria, neuro-oncology endpoints, and clinical monitoring from a neurological perspective
Prof. Fabio Ciceri	Principal investigator at Ospedale San Raffaele, responsible for developing the protocol regarding stem cell harvesting, non-myeloablative conditioning regimen, administration of ATMP (Temferon), monitoring for hematological recovery, and hematological endpoints
Dr. Marica Eoli	Principal investigator at Istituto Neurologico Carlo Besta and responsible for advising in study design, eligibility criteria, identification and management of potentially eligible patients
Prof. Alessandro Olivi	Principal investigator at Policlinico Universitario Fondazione Agostino Gemelli, responsible for advising on study design, identification and management of potentially eligible patients in Rome, and second surgical interventions if indicated
[redacted]	[redacted]
[redacted]	[redacted]
[redacted]	[redacted]
[redacted]	[redacted]

genenta science

	[redacted]
**Protocol Contributor**	**Role/ responsibility**
[redacted]	[redacted]
[redacted]	[redacted]
[redacted]	[redacted]
[redacted]	[redacted]
[redacted]	[redacted]
[redacted]	[redacted]
[redacted]	[redacted]

DocuSign Envelope ID: A0702825-E0BD-46DB-A7D6-FEF901E11877

Protocol Approval – Sponsor Signatory

**Study Title**	A phase I/IIa dose escalation study evaluating the safety and efficacy of autologous $CD34^+$-enriched hematopoietic progenitor cells genetically modified with a lentiviral vector encoding for the human interferon-α2 in patients with glioblastoma multiforme who have an unmethylated O-6-methylguanine-DNA methyltransferase gene promoter
**Protocol Number**	TEM-GBM_001
**Protocol Date**	21st October 2022

Protocol accepted and approved by:

[redacted]
Chief Medical Officer,
Genenta Science,
via Olgettina 58,
20132 Milano,
Italy

[redacted]

Signature

Date

DocuSign Envelope ID: A0702825-E0BD-46DB-A7D6-FEF901E11877

Protocol Approval – Coordinating Principal Investigator

**Study Title**	A phase I/IIa dose escalation study evaluating the safety and efficacy of autologous $CD34^+$-enriched hematopoietic progenitor cells genetically modified with a lentiviral vector encoding for the human interferon-α2 in patients with glioblastoma multiforme who have an unmethylated O-6-methylguanine-DNA methyltransferase gene promoter
**Protocol Number**	TEM-GBM_001
**Protocol Date**	21st October 2022

Protocol accepted and approved by:

Coordinating Principal Investigator
Prof. Gaetano Finocchiaro, MD
Head of Neuro-Oncology Unit
Ospedale San Raffaele,
via Olgettina 60,
20132 Milano,
Italy

[redacted] October 27, 2022

Signature Date

Declaration of Investigator

I have read and understood all sections of the protocol entitled "A phase I/IIa dose escalation study evaluating the safety and efficacy of autologous $CD34^+$-enriched hematopoietic progenitor cells genetically modified with a lentiviral vector encoding for the human interferon-α2 in patients with glioblastoma multiforme who have an unmethylated O-6-methylguanine-DNA methyltransferase gene promoter" and the accompanying investigator's brochure, version 5.0, dated 28th October 2022.

I agree to supervise all aspects of the protocol and to conduct the clinical investigation in accordance with the Amended Protocol Version 5.0, dated 21st October 2022, the International Conference on Harmonisation harmonised tripartite guideline E6(R2): Good Clinical Practice and all applicable government regulations. I will not make changes to the protocol before consulting with Genenta Science or implement protocol changes without independent ethics committee approval except to eliminate an immediate risk to patients. I agree to administer study treatment only to patients under my personal supervision or the supervision of a sub-investigator.

I will not supply the investigational drug to any person not authorized to receive it. Confidentiality will be protected. Patient identity will not be disclosed to third parties or appear in any study reports or publications.

I will not disclose information regarding this clinical investigation or publish results of the [redacted]horization from Genenta Science.

[redacted]vestigator

27th October 2022
Date

IRCCS OSPEDALE SAN RAFFAELE
PROF. FABIO CICERI
SPECIALISTA IN EMATOLOGIA
[redacted]

Printed Name of Principal Investigator

DocuSign Envelope ID: A0702825-E0BD-46DB-A7D6-FEF901E11877

Declaration of Investigator

I have read and understood all sections of the protocol entitled "A phase I/IIa dose escalation study evaluating the safety and efficacy of autologous $CD34^+$-enriched hematopoietic progenitor cells genetically modified with a lentiviral vector encoding for the human interferon-$\alpha 2$ in patients with glioblastoma multiforme who have an unmethylated O-6-methylguanine-DNA methyltransferase gene promoter" and the accompanying investigator's brochure, version 5.0, dated 28th October 2022.

I agree to supervise all aspects of the protocol and to conduct the clinical investigation in accordance with the Amended Protocol Version 5.0, dated 21st October 2022, the International Conference on Harmonisation harmonised tripartite guideline E6(R2): Good Clinical Practice and all applicable government regulations. I will not make changes to the protocol before consulting with Genenta Science or implement protocol changes without independent ethics committee approval except to eliminate an immediate risk to patients. I agree to administer study treatment only to patients under my personal supervision or the supervision of a sub-investigator.

I will not supply the investigational drug to any person not authorized to receive it. Confidentiality will be protected. Patient identity will not be disclosed to third parties or appear in any study reports or publications.

I will not disclose information regarding this clinical investigation or publish results of the investigation without authorization from Genenta Science.

[redacted]
Signature of Principal Investigator

27th October 2022
Date

MARICA EOLI
Printed Name of Principal Investigator

Declaration of Investigator

I have read and understood all sections of the protocol entitled "A phase I/IIa dose escalation study evaluating the safety and efficacy of autologous $CD34^{+}$-enriched hematopoietic progenitor cells genetically modified with a lentiviral vector encoding for the human interferon-α2 in patients with glioblastoma multiforme who have an unmethylated O-6-methylguanine-DNA methyltransferase gene promoter" and the accompanying investigator's brochure, version 5.0, dated 28th October 2022.

I agree to supervise all aspects of the protocol and to conduct the clinical investigation in accordance with the Amended Protocol Version 5.0, dated 21st October 2022, the International Conference on Harmonisation harmonised tripartite guideline E6(R2): Good Clinical Practice and all applicable government regulations. I will not make changes to the protocol before consulting with Genenta Science or implement protocol changes without independent ethics committee approval except to eliminate an immediate risk to patients. I agree to administer study treatment only to patients under my personal supervision or the supervision of a sub-investigator.

I will not supply the investigational drug to any person not authorized to receive it. Confidentiality will be protected. Patient identity will not be disclosed to third parties or appear in any study reports or publications.

I will not disclose information regarding this clinical investigation or publish results of the investigation without authorization from Genenta Science.

[redacted]

Signature of Principal Investigator

27th October 2022

Date

PROF. ALESSANDRO OLIVI

Printed Name of Principal Investigator

DocuSign Envelope ID: A0702825-E0BD-46DB-A7D6-FEF901E11877

Version History

Reserved to Sponsor

Protocol version N°	Date issued	Purpose of modifications	Regulatory Approvals
V1.0	03rd May 2018	Version submitted to the OSR Ethics Committee (EC) for CTA pre-submission review	N/A
V1.1	11th June 2018	Changes included changing the list of contributors to the protocol, clarification of exploratory endpoints, clarification on the role of the data monitoring committee, addition of prior hypersensitivity to protocol specified medications as an exclusion criterion, inclusion of the NANO scale as a clinical assessment, and minor typographical clarifications	Submitted to AIFA and OSR EC
V1.2	29th October 2018	Protocol amendment – See Appendix 12.2 for details	Approved by AIFA and OSR EC
V2.0	05th February 2019	Protocol amendment – See Appendix 12.2 for details	Approved by AIFA and OSR EC
V3.0	27th April 2020	Protocol amendment – See Appendix 12.2 for details	Approved by AIFA and OSR EC
V4.0	17th July 2020	Protocol amendment – See Summary of Changes Page 9 - 19	Approved by AIFA and OSR EC
V4.1	14th December 2020	Protocol amendment (Change in Principal Investigator at INCB); see Summary of Changes Page 9	Approved by AIFA and OSR EC
V4.2	05th July 2021	Protocol amendment (Changes in medical monitor and study outline); See Summary of Changes Page 9	Approved by AIFA and OSR EC
V5.0	21st October 2022	Protocol amendment (Changes in study design and conditioning); See Summary of Changes Page 9	

TEM-GBM_001 Clinical Protocol Amendment 7
SUMMARY OF CHANGES

This protocol (Version 5.0 of 21st October 2022) replaces previous version (Version 4.2 of 5th July 2021).

This amendment includes the following substantial changes to the protocol:

- Two cohorts to use busulfan monotherapy, each of 3 patients, in Part A. Both cohorts will utilize the same conditioning regimen of busulfan and the infusion of 3×10^6 non-modified $CD34^+$ cells/kg. Patients in Cohort 7 will receive 3×10^6 transduced cells/kg, while patients in Cohort 8 will receive 4×10^6 transduced cells/kg. Following completion of the DMC review of data from both Cohorts 7 and 8, the DMC will advise on dose escalation (after Cohort 7) or dosing of an additional 3 subjects.

Other administrative changes or corrections have also been performed, as detailed below.

(Note: added text is ***bold in italic***; deleted text is ~~strikethrough)~~

Location	Rationale	New (Version 5.0 of 21st October 2022)
Headers throughout the document, signature pages, page 2	Version update	***Amendment V5.0 of 21st October 2022*** ~~Amendment V4.2 of 5th July 2021~~
Medical Monitor, page 2	Change in Medical Monitor	[redacted]
SAE reporting, page 2<br><br>Section 6.3.3. page 140	Change in name of PV vendor	[redacted]

DocuSign Envelope ID: A0702825-E0BD-46DB-A7D6-FEF901E11877

Table of Contents

Table of Contents 10
List of Tables....................15
Protocol Synopsis....................16
List of Abbreviations....................45
1 Introduction 48
1.1 Sponsor and product background....................48
1.2 Glioblastoma multiforme and existing treatment modalities....................49
1.2.1 The disease burden and biological features of glioblastoma 49
1.2.2 The prognostic role for monocytes in GBM....................51
1.2.3 Treatment approaches for patients with newly diagnosed GBM....................52
1.3 Rationale for gene therapy approach in GBM 53
1.3.1 Tie2 expressing monocytes....................53
1.3.2 Relevance of human Tie2-expressing macrophages in sustaining glioma progression....................54
1.3.3 Interferons as anti-cancer drugs and our new gene delivery strategy......57
1.3.4 IFN-α and GBM....................59
1.3.5 Preclinical studies with Tie2 cassette in experimental tumor models 60
1.3.6 Clinical experience with lentiviral-mediated *ex-vivo* gene transfer into hematopoietic stem and progenitor cells 63
1.4 Clinical rationale for Temferon....................64
1.5 Dosage justification for Temferon 65
1.6 Benefit-risk assessment for Temferon....................69
1.6.1 Potential benefits....................69
1.6.2 Potential risks....................69
2 Study Objectives and Endpoints 82
2.1 Primary Objectives....................82
2.2 Secondary Objectives....................82
2.3 Primary Endpoint....................82
2.4 Secondary Endpoints 83

DocuSign Envelope ID: A0702825-E0BD-46DB-A7D6-FEF901E11877

2.5 Exploratory Endpoints 84
3 Investigational Plan 86
3.1 Study Design 86
3.2 Intervention Scheme 88
3.3 Rationale of Study Design 89
3.4 Study Phases 89
3.4.1 Enrolment 90
3.4.2 Screening 90
3.4.3 HSPC harvest 91
3.4.4 Temferon production 91
3.4.5 Temferon treatment 91
3.4.6 Follow-up 92
3.4.7 End of study / long term follow-up study 93
4 Patient Selection and Withdrawal Criteria 94
4.1 Selection of Study Population 94
4.1.1 Inclusion Criteria 94
4.1.2 Exclusion Criteria 95
4.2 Withdrawal of Patients from the Study 96
4.2.1 Reasons for Withdrawal/Discontinuation 97
4.2.2 Handling of Withdrawals 98
4.2.3 Replacements 98
4.2.4 Temporary suspension of the clinical study 99
5 Treatment plan 100
5.1 Method of Assigning Patients to Treatment Groups 100
5.2 HSPC mobilization and harvest 100
5.3 Lenograstim and plerixafor 102
5.4 Temferon manufacturing & characterization 102
5.4.1 Temferon definition 102
5.4.2 Drug substance and drug product manufacturing 102
5.5 Conditioning regimen 105
5.5.1 Busulfan & Thiotepa (Cohort 5) 105
5.5.2 Busulfan monotherapy (Cohorts 7 and 8) 106

DocuSign Envelope ID: A0702825-E0BD-46DB-A7D6-FEF901E11877

5.6 Infusion of non-manipulated, cryopreserved apheresis 108
5.7 Dosing of Temferon 109
5.7.1 Dose Levels and Dose Adjustments....................................109
5.7.2 Cohort Assignment 110
5.7.3 Dose-Limiting Toxicity (DLT) and Maximum Tolerated Dose (MTD) 111
5.8 Administration of Temferon....................................112
5.9 Treatment and Study Compliance....................................112
5.10 Overdose 112
5.10.1 Reporting of Overdose....................................112
5.10.2 Treatment of Overdose 113
5.11 Prior, Concomitant and Subsequent Therapy....................................113
5.11.1 Supportive therapy....................................113
5.11.2 Back-up stem cell infusion and rescue transplant....................................114
6 Study Assessments and Procedures....................................115
6.1 Schedule of Events....................................115
6.2 Assessments and Procedures....................................115
6.2.1 Eligibility Assessments....................................115
6.2.2 Safety Assessments....................................116
6.2.3 Efficacy Assessments....................................120
6.2.4 Exploratory Assessments 122
6.2.5 Clinical Laboratory Assessments....................................125
6.2.6 Emergency Assessments and Procedures during COVID-19 pandemic 127
6.3 Adverse Events 129
6.3.1 Definitions of Adverse Events 129
6.3.2 Eliciting and Documenting Adverse Events 130
6.3.3 Reporting Adverse Events 131
6.4 Data Monitoring Committee (DMC) 134
6.5 Pregnancy....................................134
6.6 Laboratory Analyses 135
6.7 Sample Collections 135
7 Statistical and Analytical Plan....................................136

DocuSign Envelope ID: A0702825-E0BD-46DB-A7D6-FEF901E11877

7.1 Sample Size Calculations....136
7.2 Analysis Sets....136
7.3 Description of Subgroups to be Analyzed....136
7.4 Statistical Analysis Methodology....136
7.4.1 Safety Analysis....137
7.4.2 Other Analyses....137
7.4.3 Interim Analyses....138
7.5 Data Quality Assurance....138
7.6 Data Management....139
8 Ethics....140
8.1 Independent Ethics Committee (IEC)....140
8.2 Ethical Conduct of the Study....140
8.3 Patient Information and Consent....140
8.4 Minimizing risk....141
9 Investigator's Obligations....142
9.1 Confidentiality....142
9.2 Financial Disclosure and Obligations....142
9.3 Investigator Documentation....142
9.4 Study Conduct....143
9.5 Adherence to Protocol....143
9.6 Adverse Events and Study Report Requirements....143
9.7 Investigator's Final Report....143
9.8 Records Retention....143
9.9 Liability and Insurance....144
9.10 Publications....144
10 Study Management....145
10.1 Monitoring....145
10.1.1 Clinical Study Monitor....145
10.1.2 Inspection of Records....145
10.2 Management of Protocol Amendments and Deviations....146

DocuSign Envelope ID: A0702825-E0BD-46DB-A7D6-FEF901E11877

10.2.1 Modification of the Protocol 146
10.2.2 Protocol Deviations 146
10.3 Study Termination 146
10.3.1 End of Study 147
10.3.2 Premature termination of the trial 147
10.4 Final Report 147
11 Reference List 149
12 Appendices 165
12.1 Appendix: Schedule of Events 165
12.2 Summary of Prior Protocol Amendments 180

DocuSign Envelope ID: A0702825-E0BD-46DB-A7D6-FEF901E11877

List of Tables

Table 5-1 Dosing scheme for Temferon.......... 109

Table 5-2 Temferon dose adjustments will be performed according to VCN.......... 109

Table 6-1 Assessment of AE severity.......... 132

Table 6-2 Assessment of AE causality.......... 133

Table 12-1 Schedule of Events (Screening to Day +29): INCB/OSR (Cohorts 1-6).......... 166

Table 12-2 Schedule of Events (Screening to Day +29): INCB/OSR (Cohorts 7 and 8).......... 169

Table 12-3 Schedule of Events (Day +30 to end of study): INCB/OSR.......... 172

Table 12-4 Schedule of Events (Screening to Day +29): PG.......... 174

Table 12-5 Schedule of Events (Day +30 to end of study): PG.......... 177

Protocol Synopsis

**Protocol Number:**	TEM-GBM_001
**Title:**	A phase I/IIa dose escalation study evaluating the safety and efficacy of autologous CD34$^+$-enriched hematopoietic progenitor cells genetically modified with a lentiviral vector encoding for the human interferon-α2 in patients with glioblastoma multiforme who have an unmethylated O-6-methylguanine-DNA methyltransferase gene promoter
**Sponsor:**	Genenta Science,<br>via Olgettina 58,<br>20132 Milano,<br>Italy
**Study Phase:**	I/IIa
**Study Sites:**	Ospedale San Raffaele and Istituto Neurologico Carlo Besta, Milan, Italy<br><br>Policlinico Universitario Fondazione Agostino Gemelli, Rome, Italy
**Indication:**	Glioblastoma Multiforme (GBM)
**Rationale:**	GBM remains the most common malignant primary brain tumor contributing to more than half of all gliomas and making up 16% of all primary brain tumors. Untreated, the median survival is only 3 months and treatments have resulted in improved survival but despite innovations elsewhere in the field of oncology, the prognosis for patients with GBM remains poor with median overall survival of approximately 13 to 15 months. Surgery remains the mainstay of initial treatment enabling not only histological confirmation of the diagnosis but also by reducing tumor burden and achieving complete resection, improves survival. The combination of radiotherapy and

DocuSign Envelope ID: A0702825-E0BD-46DB-A7D6-FEF901E11877

temozolomide (TMZ) is the standard of care following initial surgery. Nevertheless, particularly in patients who have an unmethylated O-6-methylguanine-DNA methyltransferase (MGMT) gene promoter who do not respond favourably to long term treatment with TMZ, there is a need for new treatment options. Reprogramming of the tumor microenvironment together with direct effects on tumor cells and neo-angiogenesis via a cell mediated IFN-α approach is an attractive proposition for further study.

Systemically or locally administered IFN-α is not currently standard of care in the management of patients with GBM although a large number of historical publications have reported clinical outcomes in patients with malignant brain tumors including GBM where they were treated with IFN-α injected either directly into the tumor or administered parenterally. A more recent phase 3 study comparing the combination of IFN-α and carmustine compared to carmustine alone following radiotherapy in patients with newly diagnosed high grade glioma, showed no difference between the groups in overall survival or progression free survival. However, a higher rate of withdrawal from the study due to poor tolerability was noted in patients treated with the combination. More recently two small single-arm studies evaluated the efficacy of IFN-α in combination with TMZ in patients with recurrent disease and both overall survival and progression free survival compared favorably with contemporary observational data in a large cohort with recurrent disease many of whom were treated with radiosurgery and second line agents. However, none of these studies specifically addressed the unmet need for patients with unmethylated MGMT promoter status.

The potential importance of IFN-α in GBM is also highlighted by other mechanistic data relating to IFN-α gene content in the tumor and deletion of the IFN gene locus (chromosome 9p). These observations suggest that a possible alternative source of IFN which minimizes systemic toxic effects and is used in combination with current standard of care, might be effective in GBM treatment. Furthermore, other

DocuSign Envelope ID: A0702825-E0BD-46DB-A7D6-FEF901E11877

factors such as a high tumor mutation and neo-antigen burden as well as defects in the DNA repair machinery and genomic instability may correlate with the response to immunotherapy and ionizing radiation, which is part of the standard of care for glioblastoma treatment, may also be associated with immuno-modulatory properties. Targeted release of IFN-α using a cell-based delivery mechanism is therefore an innovative and clinically relevant approach to modulating the immune microenvironment in GBM.

A subset of circulating and tumor-infiltrating bone marrow derived monocyte/macrophages characterized by the expression of the angiopoietin receptor Tie2 has been described. These Tie2-expressing macrophages (TEMs) have features of M2-polarized, tumor-associated macrophages (TAMs), promote both developmental and tumor angiogenesis, are required for the formation of tumor blood vessels and are infrequently found in normal organs. Notably, the Tie2 receptor is weakly expressed by the circulating monocytes but is significantly upregulated upon their homing to tumors. Human TEMs are often found at higher frequency in the blood of tumor-bearing individuals and colonize the stroma of many tumors. Several studies have shown that tumor infiltration by M2-polarized TAMs (including TEMs) is a negative prognostic factor, highlighting a likely role of these cells in fostering tumor invasion and growth, conceivably by promoting angiogenesis and suppressing anti-tumor immunity, suggesting that recruitment of these M2 macrophages to the tumor may be an obligate step for tumor progression and may represent a novel target for anti-tumor therapies. In this regard, TEMs represent a suitable carrier for the local and tumor-restricted release of an anti-tumor molecule thus enabling to combine the maximum therapeutic efficacy with a consistent reduction in the potential systemic toxicity. The selective expression of IFN-α in mouse and human TEMs has been demonstrated by transducing HSPCs with a third-generation lentiviral vector in which the mouse/human Tie2/TEK enhancer/promoter sequence has been cloned. The vector design for IFN-α gene therapy

DocuSign Envelope ID: A0702825-E0BD-46DB-A7D6-FEF901E11877

	has been optimized by adding target sequences recognized by microRNA-126 (miR-126) to the construct. This miRNA, being endogenously expressed to high levels in HSPCs and down-regulated in the relative differentiated progeny, provides the opportunity to combine a robust suppression of the transgene expression in human and mouse HSPCs with a strong expression in the differentiated progeny thus minimizing possible detrimental effects on the stem/progenitor cells.<br><br>Temferon, the investigational ATMP used in this study, consists of autologous $CD34^+$-enriched hematopoietic progenitor cells exposed in vitro to TIA126 lentiviral vector encoding for the human IFN-α2 gene. Its expression is tightly controlled by the human TIE2 enhancer/promoter sequence and by a post-transcriptional regulation layer represented by two target sequences for miR-126. This enables suppression of IFN-α expression in HSPCs, thereby further increasing the specificity of the delivery strategy for their Tie2 expressing myeloid cell progeny.
**Objectives:**	The primary objective of TEM-GBM_001 study is to evaluate the safety and tolerability of 5 escalating doses of Temferon, in up to 27 patients with GBM and an unmethylated MGMT promoter, following first line radiotherapy. Patients are assigned to 8 different cohorts, each including 3 patients. The <u>first 3 cohorts</u> studied Temferon dose escalation up to Dose Level 3 (2 x 10^6 CD34+ cells/kg). Cohorts 4 and 5 were infused with Temferon Dose Level 3, reducing the number of non-manipulated supporter cells to 2 x 10^6 CD34+ cells/kg and evaluating the safety, tolerability and long-term hematologic engraftment after 2 different conditioning regimens (BCNU & thiotepa vs Busulfan & thiotepa, respectively). Cohort 6 patients received Temferon Dose Level 4 (3 x 10^6 CD34+ cells/kg) and non-manipulated supporter cells to 2 x 10^6 CD34+ cells/kg which were administered after BCNU and thiotepa conditioning. Dose-limiting toxicities have not been observed in the 18 patients that were treated up to Temferon Dose Level 4. However, serious adverse events (CTCAE $\geq$ Grade 3)

	attributed to the conditioning regimen have occurred associated with hematologic complications (febrile neutropenia, secondary graft failure), venous thromboembolism, pneumonia and other infections. The majority of patients have also received dexamethasone during the post-transplant period as a treatment for radiotherapy associated cerebral edema. The combination of glioblastoma, brain irradiation, conditioning chemotherapy and dexamethasone has therefore contributed to complications resulting from immunosuppression.<br><br>In order to mitigate these recognised risks, the conditioning chemotherapy regimen will be modified in two additional cohorts to utilise busulfan monotherapy (total busulfan AUC target 60 mg·h/L). Thiotepa will be omitted. In addition, patients utilizing dexamethasone > 4mg qd within 20 days of Temferon administration will undergo frequent medical review over 3 weeks to reduce dexamethasone requirements to ≤ 4mg qd.<br><br>Cohorts 7 and 8 will recruit 3 patients each who will be administered busulfan monotherapy and an infusion of 3 x 10^6 non-modified $CD34^+$ cells/kg. Patients in Cohort 7 will receive 3 x 10^6 transduced cells/kg (Dose Level 4), while patients in Cohort 8 will receive 4 x 10^6 transduced cells/kg (Dose Level 5).<br><br>Following completion of dosing in Cohorts 7 and 8 and review of data by the DMC, an additional 3 patients will receive Temferon at a dose selected from either Cohort 7 or 8 in order to provide additional safety data before progression to Part B of the study.<br><br>The secondary objective of this study is to evaluate biological activity and efficacy of Temferon in patients with GBM and unmethylated MGMT when used following standard of care first line therapeutic approaches.
**Patient Population:**	Each patient must meet all of the following criteria to be enrolled in this study:

Inclusion Criteria at Screening:

- Patients aged between 18 and ≤ 70.
- Histologically confirmed, newly diagnosed supratentorial glioblastoma with unmethylated MGMT gene promoter.
- Patients have undergone complete or partial tumor resection.
- Able and willing to provide written informed consent and comply with the study protocol and procedures.
- Eligible for radiotherapy.
- Life expectancy of 6 months or more at Screening.
- Women of child-bearing potential enrolled in the study must have a negative pregnancy test at screening and agree to use two distinct acceptable methods of contraception during the trial.
- Men enrolled in the study with partners who are women of child-bearing potential, must be willing to use an acceptable barrier contraceptive method during the trial or have undergone successful vasectomy at least 6 months prior to entry into the study. Successful vasectomy needs to have been confirmed by semen analysis.
- Karnofsky performance score (KPS) ≥ 70.

Additional inclusion criteria to be assessed within 20 days of Temferon administration:

- Adequate cardiac, renal, hepatic and pulmonary function as evidenced by:
    - Left ventricular ejection fraction (LVEF) ≥ 45% by echo and normal electrocardiogram (ECG) or presence of abnormalities not significant for cardiac disease. Absence of severe pulmonary hypertension;

- Diffusing capacity of the lung for carbon monoxide (DLCO) > 50% and forced expiratory volume in 1 sec (FEV1) and forced expiratory vital capacity (FVC) > 60% predicted (if non cooperative: pulse oximetry > 95 % in room air);
- Serum creatinine < 2x upper limit normal and estimated glomerular filtration rate (eGFR) ≥ 30ml/min/1.73m^2;
- Alkaline phosphatase (ALP), alanine aminotransferase (ALT) and/or aspartate aminotransferase (AST) ≤ 2.5 x upper limit of normal (ULN), and total bilirubin ≤ 2.0 mg/dl.
- Hemoglobin ≥ 10 g/dL, platelet count ≥ 100000/mm^3, absolute neutrophil count > 1500/mm^3.

Exclusion criteria at Screening:

Patients meeting any of the following criteria will be excluded from the study:

- Use of other investigational agents or procedures within 4 weeks prior to study enrolment (within 6 weeks if use of long-acting agents) or participation in a previous gene therapy study.
- Known hypersensitivity to carmustine (or any other nitrosurea), thiotepa, busulfan, lenograstim, plerixafor, or any excipients used in these products.
- Receipt of any oral or parenteral chemotherapy or immunotherapy within 2 years of Screening.
- Previous allogeneic bone marrow transplantation, kidney or liver transplant.
- Clinical evidence of persistent raised intracranial pressure following surgical resection.

DocuSign Envelope ID: A0702825-E0BD-46DB-A7D6-FEF901E11877

- Clinically relevant active viral, bacterial, or fungal infection at eligibility evaluation.
- Active autoimmune disease or a relevant history of important autoimmune manifestations, in particular psoriasis, systemic lupus erythematosus (SLE), rheumatoid arthritis, vasculitis, immune-mediated peripheral neuropathies.
- History of sarcoidosis.
- History or current evidence of neuropsychiatric illness including depression, schizophrenia, bipolar disorders, impaired cognitive function, dementia or suicidal tendency.
- History of severe cardiovascular disease such as prior stroke, coronary artery disease requiring intervention or unresolved arrhythmias in the past 6 months.
- Evidence of any hematological neoplasm.
- Positivity for human immunodeficiency virus type 1 or 2 (HIV-1, HIV-2) (serology or RNA), and/or Hepatitis B Virus Surface Antigen (HbsAg) and/or Hepatitis B Virus (HBV) DNA and/or Hepatitis C virus (HCV) RNA (or negative HCV RNA but on antiviral treatment) and/or Treponema Pallidum or Mycoplasma active infection.
- Active alcohol or substance abuse within 6 months of the study.
- Current pregnancy or lactation.
- Known bleeding diathesis or history of abnormal bleeding, or any other known coagulation abnormalities that would contraindicate lumbar puncture for CSF or future surgery.
- Use of immunosuppressants with the exception of steroids. The maximum permitted dexamethasone (or equivalent) dose is 4 mg per day.

**Study Design:**	This is a non-randomized, open label, multicenter, phase I/IIa, therapeutic-exploratory, dose-escalation, prospective study, involving

DocuSign Envelope ID: A0702825-E0BD-46DB-A7D6-FEF901E11877

a single injection of Temferon, an investigational ATMP consisting of autologous CD34$^+$-enriched HSPCs exposed to transduction with a 3rd generation, vesicular stomatitis virus G (VSV-G) pseudo-typed lentiviral vector driving myeloid-specific IFN-α2 expression, which will be administered to up to 27 patients affected by GBM who have an unmethylated MGMT promoter. The study will recruit and follow up patients at specialist neurosurgical and neuro-oncology units in Italy. Administration of Temferon and hematological follow up will take place at specialist hematology and bone marrow transplantation units.

Potentially eligible patients will be identified immediately after surgical resection of GBM once the MGMT promoter methylator status is known. Once written, informed consent is obtained, and screening procedures have been completed, harvesting of HSPCs will occur. A standard of care regimen of radiotherapy without TMZ lasting approximately 6 weeks, will then take place. During this time, Temferon manufacturing will occur at [redacted]

Following completion of radiotherapy, patients will be admitted to the bone marrow transplant unit for receipt of a conditioning regimen. In Cohorts 1 to 4 conditioning consisted of BCNU and thiotepa. In Cohort 5, which ran in parallel with Cohort 4, patients were conditioned with busulfan and thiotepa before being administered with of non-manipulated HSPCs and Temferon. Patients in Cohort 6 received BCNU and thiotepa conditioning and an infusion of 2 x 10^6 non-modified CD34$^+$ cells/kg and 3 x 10^6 transduced cells/kg (Dose Level 4). Patients in Cohorts 7 and 8 will receive busulfan monotherapy as conditioning and an infusion of 3 x 10^6 non-modified CD34$^+$ cells/kg. Patients in Cohort 7 will receive 3 x 10^6 transduced cells/kg (Dose Level 4), while patients in Cohort 8 will receive 4 x 10^6 transduced cells/kg (Dose Level 5). In-patient monitoring will occur until hematological recovery occurs.

Thereafter, regular follow-up of patients will occur up to 2 years (+720 days) with the assessments and procedures performed within neurosurgery/oncology and hematology units. At the +720 day visit, patients will be invited to participate in a long term follow-up study (TEM-LT) which will last for an additional 6 years.

Escalating doses of Temferon, from Dose 1 to 5, were and will be administered to cohorts of 3 patients each as follows:

- Dose 1 – Cohort 1 (Completed)
- Dose 2 – Cohort 2 (Completed)
- Dose 3 – Cohorts 3 and 4, 5 (Completed)
- Dose 4 – Cohort 6 (Completed), and 7
- Dose 5 - Cohort 8

Cohorts 1 to 3 recruited and ran sequentially, while Cohorts 4 and 5 recruited and ran in parallel. Cohort 6 ran sequentially after Cohorts 4 and 5. Cohorts 7 and 8 will recruit and run sequentially.

Cohorts 1 to 3 received 3 x 10^6 non-modified $CD34^+$ cells/kg, while Cohorts 4 and 5 received 2 x 10^6 non-modified $CD34^+$ cells/kg. Cohorts 6 received 2 x 10^6 non-modified $CD34^+$ cells/kg. Cohorts 7 and 8 will receive 3 x 10^6 non-modified CD34+ cells/kg as in Cohorts 1 to 3.

Patients in Cohorts 7 and 8 will receive intravenous infusion of 3.0 x 10^6 Temferon cells/kg ($CD34^+$/kg) and 4.0 x 10^6 Temferon cells/kg ($CD34^+$/kg), respectively.

Following completion of dosing in Cohorts 7 and 8 and review of data by the DMC, an additional 3 patients will receive Temferon at a dose selected from either Cohort 7 or 8 in order to provide additional safety data before progression to Part B of the study.

DocuSign Envelope ID: A0702825-E0BD-46DB-A7D6-FEF901E11877

	In the event that GBM disease progression occurs, patients will be managed with second line therapies including second surgery, TMZ, BCNU, fotemustine or any other approved therapy for GBM.
**Study Phases**	Seven protocol phases are foreseen :<br><br>1. *Enrolment*, with signing of the informed consent form.<br><br>2. *Screening and eligibility-determining phase*, during which inclusion/exclusion criteria will be assessed (this will occur approximately 7 to 8 weeks prior to Temferon administration on Day 0).<br><br>3. *HSPC harvesting* (within 4 weeks following Screening), which will occur before standard of care treatment with radiotherapy administered for 6 weeks.<br><br>4. *Temferon production phase*, following HSPC harvest and up to Temferon release for clinical use by qualified person (QP). This will occur while standard of care treatment with radiotherapy occurs.<br><br>5. *Temferon treatment phase:* Once the Investigator receives confirmation that Temferon is available to administer to the patient, the conditioning regimen will be administered at Baseline (Cohorts 1 to 4 and Cohort 6: BCNU will be administered to the patient on Day -6, followed by thiotepa on Day -5; Cohort 5: Thiotepa on Day -5 and -4, Busulfan Day -4 to Day-2; Cohorts 7 and 8: Busulfan Day -4 to Day-2). Non-manipulated HSPCs and Temferon will be infused on Day 0.<br><br>6. *Follow-up phase:* from Temferon infusion until +720 days following experimental treatment.<br><br>7. *End of study phase:* At the end of the study (+720 days), patients will be invited to continue long term follow up as required by the authorities (up to 8 years in Italy after gene

DocuSign Envelope ID: A0702825-E0BD-46DB-A7D6-FEF901E11877

	therapy administration). Additional written informed consent will be required to participate in this long term follow up study (TEM-LT).
**Estimated Study Duration:**	The study will last approximately 2 years and 3 months from the first visit to obtain written informed consent to the final visit. A total of 20 visits will be required including assessments, day hospital visits and in-patient hospitalization. At the end of the study, patients will be invited to participate in a long term follow up (up to an additional 6 years) study requiring additional informed consent to be obtained.
**Primary Endpoint:**	The primary endpoint is to assess the tolerability and safety of Temferon over the first 90 days following Temferon administration, as evaluated by:<br>• Routine clinical and laboratory surveillance;<br>• Assessment of autoimmune manifestations;<br>• Significant organ toxicities (>grade 2 according to the National Cancer Institute Common Terminology Criteria for Adverse Events [NCI CTCAE v5.0] that are attributed to Temferon exposure or procedures related to the harvesting of HSPCs or administration of Temferon.
**Secondary Endpoints:**	• Long-term tolerability and safety of Temferon up to 2 years following Temferon administration, according to CTCAE v5.0 criteria as evaluated by:<br>o Routine clinical and laboratory surveillance;<br>o Assessment of autoimmune manifestations;<br>o Significant organ toxicities (>grade 2 according to CTCAE v5.0) that can be attributed to Temferon exposure.<br>• The proportion of patients achieving hematologic recovery by Day +30 following administration of non-manipulated autologous HSPC and Temferon. Hematologic recovery is

DocuSign Envelope ID: A0702825-E0BD-46DB-A7D6-FEF901E11877

defined as the first of at least 3 consecutive days with a neutrophil count > 0.5 x 10^9/L and platelet count > 20 x 10^9/L.

Safety (Part A only)

- Determine the maximum tolerated dose (MTD) of Temferon as defined by the presence of a CTCAE Grade 3-5 adverse event (AE) that occurs within the first 30 days and is attributed to Temferon.

- Assess the tolerability and safety of three conditioning regimens (BCNU and thiotepa, busulfan and thiotepa, or busulfan monotherapy), up to Day +45 following administration of Temferon, in patients with GBM.

Efficacy (Part A & Part B, if any)

- Identify the presence of transduced myeloid cells in bone marrow aspirate at Day +30 and +90 following administration of Temferon as determined by Vector Copy Number (VCN).
- Identify the presence of transduced myeloid cells in peripheral blood (PB) between Day +30 and +90 following administration of Temferon as determined by VCN.
- Evaluate persistent transduced myeloid cells in PB as assessed by VCN. Persistence is defined as a VCN above the level of detection evident for at least 12 weeks. Persistence according to the type of conditioning regimen used, will also be evaluated.
- Determine the proportions of patients achieving complete response (CR), partial response (PR), stable disease (SD), and progressive disease (PD) at 6 months (Day +180) following Temferon administration, using standard iRANO criteria.
- Clinical response will also be evaluated at Day +90, +120 and every 2 months until Day +720. Time to progression/progression-free survival (PFS) will be measured

DocuSign Envelope ID: A0702825-E0BD-46DB-A7D6-FEF901E11877

	from the first day following Temferon administration until unequivocal progression is identified.<br>• Overall Survival (OS) at various timepoints up to 2 years, will be calculated from the first day following Temferon administration.<br>• Changes over time (Day +30, +90, +180, +360, +540, +720) in functional status using the Eastern Cooperative Oncology Group Performance Status (ECOG) and Karnofsky Performance Status (KPS).<br>• Changes over time (Day +90, +180, +360, +720) in quality of life using the EORTC Quality of Life Questionnaires C30 and BN20.
**Exploratory Endpoints:**	• Imaging characteristics of the tumor over time according to MRI and additional functional imaging<br>• Subgroup analyses of clinical response and imaging parameters according to baseline characteristics.<br>• Immunohistochemistry characteristics of tumors at first surgery and following repeat surgery including proliferative and apoptotic index, type of vascularization, EGFR and EGFRvIII expression, evaluation of T cells and myeloid cells infiltrate including the presence of transduced Tie-2 expressing monocytes, and presence/absence of IFN-α gene expression<br>• Tumor infiltrating lymphocytes content analysis in tumor samples at diagnosis and correlation with clinical response.<br>• Evaluation of the T cell immune repertoire in tumor sample and PB at diagnosis, in tumors following repeat surgery and in PB over time after Temferon administration (Day -6, +30, +90, +180, +360 and +720).<br>• Changes in general immune system composition in peripheral blood over time.

DocuSign Envelope ID: A0702825-E0BD-46DB-A7D6-FEF901E11877

	• Characterization of IFN-α gene signatures in tumors following repeat surgery.<br>• Characterization of IFN-α gene signatures and mutations in cerebrospinal fluid (CSF) over time.<br>• Characterization of IFN-α gene signatures in bone marrow and peripheral blood over time.<br>• Characterization of genetic and epigenetic changes (MGMT methylation) in tumors at first surgery and following repeat surgery.<br>• Future proteomic and metabolomic analyses to identify biomarkers for clinical response.<br>• Concentration of IFN-α2 in peripheral blood and CSF over time.<br>• Concentration of cytokines in CSF over time.<br>• Identification of presence of transduced cells in CSF over time (as determined by VCN).
**Safety Assessments:**	Safety assessments will include hematologic recovery by Day +30 following Temferon infusion, defined by the absence of grade 3/4 neutropenia or grade 3/4 thrombocytopenia, as assessed by CTCAE v4.1, or recovery of pre-treatment blood counts. Complete blood counts will be performed daily after Temferon infusion until hematological engraftment and these data will be recorded in the eCRF. The occurrence of hematological engraftment is defined as the first of at least 3 consecutive days with a neutrophil count > 0.5 x 10^9/L and platelet count > 20 x 10^9/L (in the absence of transfusions).<br><br>Short-term tolerability (0-24 hrs from Temferon infusion) will be evaluated by AE reporting (any grade) and monitoring of the systemic reactions to cell injection (i.e. fever, tachycardia, nausea and vomiting, joint pain, skin rash). The tolerability and safety of Temferon over the first 90 days following Temferon administration will be evaluated by

routine clinical and laboratory surveillance, assessment of autoimmune manifestations and reporting of adverse events.

Safety assessments of the conditioning regimens utilized (thiotepa and BCNU for Cohorts 1-4 and 6, Cohort 5 busulfan and thiotepa, and Cohorts 7 and 8 busulfan only) will include AE reporting and routine clinical and laboratory surveillance performed during the admission to the bone marrow transplant unit for conditioning and administration of Temferon.

Absence of Replication Competent Lentivirus (RCL) will be defined as the absence of positive results of a) ELISA for HIV p24 antigen (serum); b) DNA PCR for VSV-G envelope (cells); c) RT-PCR for HIV-pol RNA (serum).

Absence of hematological malignancy resulting from treatment will include clinical examination to exclude lymphadenopathy and splenomegaly, and complete blood counts, bone marrow evaluations and chest x-ray will be performed according to the Schedule of Events (SOE) and as necessary based on clinical judgment to exclude myelodysplasia, acute myeloid leukemia, lymphomas and other hematologic malignancies.

Absence of abnormal clonal proliferation will be assessed every 6 months with clinical evaluation, blood count with differential to detect abnormal proliferation or cytopenia, routine biochemical tests to detect abnormal organ functions, flow cytometry on peripheral blood for lymphocyte subpopulations, and qPCR analysis for measuring vector copy number. Every year, bone marrow aspirates will be performed, including morphological examination, cytogenetic analysis, VCN and detailed immune phenotyping.

The safety of the myeloid-targeted IFN-α2 transgene product will be evaluated from hematologic engraftment to Day +720 by clinical and laboratory surveillance, including demonstration of 1) stable blood

DocuSign Envelope ID: A0702825-E0BD-46DB-A7D6-FEF901E11877

counts and absence of cytopenias > grade 2, not related to concomitant medications; 2) absence of autoimmune manifestations requiring treatment; 3) absence of organ toxicity (> grade 2 according to CTCAE v4.1) that can be ascribed to IFN exposure; 4) absence of systemic adverse reactions (e.g. flu-like symptoms) associated with IFN-α treatment.

Additional safety evaluations will include bone marrow and cerebrospinal fluid sample collections to evaluate vector copy number and IFN-α gene signatures.

All women of childbearing potential must undergo a serum beta-hCG test at Screening, and regularly throughout the study.

Data on the patient’s vital signs which will include body temperature, heart rate, normal oxygen saturation (SO_2) and systolic/diastolic blood pressure, will be recorded regularly throughout the study. In addition, physical and neurological examinations will be conducted regularly.

Diagnostic imaging and instrumental tests will include 12-lead ECG, echocardiography, lung function tests including diffusion capacity, and chest x-ray. A slit lamp examination performed by an ophthalmologist will take place periodically throughout the study to monitor potential ocular toxicity of IFN-α. Psychiatric assessments will also take place regularly throughout the study to monitor psychiatric toxicities associated with IFN-α, including depression and suicidal ideation.

DocuSign Envelope ID: A0702825-E0BD-46DB-A7D6-FEF901E11877

**ATMP, Dosage, and Route of Administration**	Temferon consists of autologous $CD34^+$-enriched hematopoietic progenitor cells exposed *in-vitro* to TIA126 LV encoding for the human interferon-α2 gene.<br><br>**HSPC mobilization**<br><br>Each subject will undergo Lenograstim and Plerixafor-mediated HSPC mobilization and harvest by leukapheresis per institutional practice:<br><br>Lenograstim 10 µg/kg/day divided in 2 administrations (5 µg/kg each) by subcutaneous injection (s.c.) until a sufficient number of $CD34^+$ cells is mobilized and the leukapheresis/-es has/have been concluded. Monitoring of $CD34^+$ cells in the PB will be performed.<br><br>Plerixafor 0.24 mg/kg s.c. 5 hours before commencement of leukapheresis.<br><br>If required, another leukapheresis will be performed the day after, preceded by a second plerixafor dose 5 hours before. Additional leukapheresis may be permitted, at the discretion of the Investigator, after completion of radiotherapy should additional non-manipulated $CD34^+$ HSPCs be required for co-administration or as a backup.<br><br>Mobilization target for Cohorts 6, 7 and 8 is $\geq 13 \times 10^6$ leukocytes/kg, of which a minimum of 7×10^6 $CD34^+$ cells/kg will be used for Temferon production, while a minimum of 6×10^6 leukocytes/kg will be frozen as non-manipulated apheresis product.<br><br>HSPCs will be sent to a CMO for the production of Temferon.<br><br>After transduction, Temferon will be stored frozen at ≤ -65ºC in a cryopreservative solution while aliquots are being tested for release.<br><br>Administration of Temferon will only occur once radiotherapy has completed and providing the daily requirements for dexamethasone (or another corticosteroid) is less than or equal to 4mg daily. In the event that dexamethasone (or equivalent) >4mg qd is required, the

DocuSign Envelope ID: A0702825-E0BD-46DB-A7D6-FEF901E11877

Investigator will have a period of 3 weeks from the time of receipt of the post-radiotherapy/baseline brain MRI images to control cerebral edema or manage clinical symptoms with alternative approved therapies (bevacizumab is prohibited) with the aim of reducing corticosteroid requirements to a dexamethasone (or equivalent) dose ≤4mg qd. In the event that this dose target cannot be reached, the Investigators will review the case with the Sponsor in order to discuss and agree the rationale for continuation of the patient in the study.

Experimental treatment with conditioning and infusion of Temferon:

Cohorts 1 – 4 and 6:

On Day -6, BCNU (carmustine) 400 mg/m^2 will be given by IV infusion over 1 hour.

On Day -5, thiotepa 5mg/kg will be given twice (once in the morning and once 12 hours later) as an IV infusion over 2 hours.

Cohort 5:

On Day -5 (once in the evening), thiotepa 5mg/kg will be given as an IV infusion over 2 hours. On Day -4, thiotepa 5mg/kg will be given as an IV infusion over 2 hours (once in the morning). In the evening, busulfan 3.2 mg/kg ideal body weight will be given by IV infusion over 3 hours. Therapeutic drug monitoring of busulfan will be performed with pharmacokinetic blood sampling occurring pre-dosing, 5 minutes after the end of busulfan infusion, and +2 hours, +4 hours and +6 hours after the end of the busulfan infusion. The pharmacokinetic busulfan data will be calculated. The total busulfan AUC target is between 40 to 60 mg·h/L. The busulfan dose on Day -3 may be adjusted and/or an additional dose of busulfan may be infused on Day -2 in order to ensure drug exposure within the target range.

Cohort 7 and 8:

DocuSign Envelope ID: A0702825-E0BD-46DB-A7D6-FEF901E11877

On Day -4, in the evening, busulfan 3.2 mg/kg ideal body weight will be given by IV infusion over 3 hours. Therapeutic drug monitoring of busulfan will be performed as described above for Cohort 5. Pharmacokinetic blood sampling will occur pre-dosing, 5 minutes after the end of busulfan infusion, and +2 hours, +4 hours and +6 hours after the end of the busulfan infusion. The pharmacokinetic busulfan data will be calculated. The total busulfan AUC target is 60 mg·h/L. The busulfan dose on Day -3 may be adjusted and/or an additional dose of busulfan may be infused on Day -2 in order to ensure drug exposure within the target range.

Non-manipulated $CD34^+$ HSPCs and Temferon Infusion:

On Day 0, patients receive an infusion of non-manipulated stem cell support as follows:

- Cohorts 1 to 3 were administered, at least 60 hours after completion of thiotepa infusion, with a dose of 3 x 10^6 $CD34^+$ of non-manipulated cells/kg.
- Cohort 4 was administered, at least 60 hours after completion of thiotepa infusion, with a dose of 2 x 10^6 $CD34^+$ of non-manipulated cells/kg.
- Cohort 5 was administered, at least 36 hours after completion of busulfan infusion, with a dose of 2 x 10^6 $CD34^+$ of non-manipulated cells/kg.
- Cohort 6 was administered, at least 60 hours after completion of thiotepa infusion, with a dose of 2 x 10^6 $CD34^+$ of non-manipulated cells/kg.
- Cohorts 7 and 8 will be administered, at least 36 hours after completion of busulfan infusion, with a dose of 3 x 10^6 $CD34^+$ of non-manipulated cells/kg.

After that, Temferon will be infused into a central vein or, if not feasible, into a large peripheral vein.

In Part A, the dose for Temferon is as follows:

- Dose level 1 (Cohort 1): 0.5 x 10^6 $CD34^+$ cells/kg body weight;
- Dose level 2 (Cohort 2): 1.0 x 10^6 $CD34^+$ cells/kg body weight;
- Dose level 3 (Cohorts 3, 4 and 5): 2.0 x 10^6 CD34+ cells/kg body weight;
- Dose level 4 (Cohort 6 and 7): 3.0 x 10^6 CD34+ cells/kg body weight;
- Dose level 5 (Cohort 8): 4.0 x 10^6 CD34+ cells/kg body weight.

The actual Temferon dose to be administered will be rounded to the nearest available aliquot size (maximum tolerance: +10%).

Temferon dose adjustments will be performed by the Sponsor, in agreement with the Principal Investigator, according to VCN in the DP:

**VCN**	**Dose Level 1** (cells/kg)	**Dose Level 2** (cells/kg)	**Dose Level 3** (cells/kg)	**Dose Level 4** (cells/kg)	**Dose Level 5** (cells/kg)
< 0.4	0.8 x10^6	1.5 x10^6	3.0 x10^6	≥3.0 x10^6	≥4.0 x10^6
0.4-1.0	0.5 x10^6	1.0 x10^6	2.0 x10^6	3.0 x10^6	4.0 x10^6
1.1-1.5	0.4 x10^6	0.8 x10^6	1.5 x10^6	2.3 x10^6	3.0 x10^6
>1.5	0.3 x10^6	0.5 x10^6	1.0 x10^6	1.5 x10^6	2.0 x10^6

Cohort 4/5 dose adjustments:

**VCN**	**Temferon Dose Level 3 (cells/kg)**	**Supporter $CD34^+$ cells (cells/kg)**
< 0.4	3.0 x10^6	≥1.5 x10^6
**0.4-1.0**	**2.0 x10^6**	**≥2.0 x10^6**
1.1-1.5	1.5 x10^6	≥2.5 x10^6
>1.5	1.0 x10^6	≥3.0 x10^6

No adjustment in the supporter cell dose is required for Cohorts 6, 7 or 8 as a minimum total $CD34^+$ cell dose > 4 x 10^6/kg is guaranteed in all circumstances.

Should the patient not have enough Temferon cells available for his planned dose level, the maximum number of available cells, but not less than 1.5 x 10^6/kg, will be infused.

DocuSign Envelope ID: A0702825-E0BD-46DB-A7D6-FEF901E11877

The Investigators will discuss with the Sponsor the possibility of replacing the patient in the original cohort.

In Part A, the first patient assigned to the first cohort received a single IV infusion of Temferon at Dose Level 1. Beginning on the day of treatment (Day 0), the patient was monitored for safety and treatment effects during protocol-specified study visits. For each cohort, a minimum of 30 days will separate dosing of the first patient and commencement of conditioning for the second patient within a cohort. Conditioning of the third patient will occur a minimum of 30 days after the second patient received Temferon.

Patients in Cohorts 3, 4 and 5 were administered with the same dose of Temferon (Dose Level 3). Interim data from Cohorts 1 to 3 were reviewed by the independent Data Monitoring Committee (DMC) and agreement was reached to progress to Cohorts 4 and 5. Both of these cohorts recruited patients and ran in parallel, using the same criteria as described above for between patient dosing intervals within each cohort.

Patients in Cohorts 6 utilized the same conditioning regimen (BCNU and thiotepa) and were administered with the same dose of unmodified cells as in Cohorts 4 and 5.

Patients in Cohorts 7 and 8 will use busulfan monotherapy as conditioning and will receive the same dose of unmodified cells as in Cohorts 1-3.

Prior to progression to Part B, an additional 3 patients will receive busulfan monotherapy as conditioning, the same dose of unmodified cells as in Cohorts 1-3, 7 and 8, and a dose of Temferon selected from Cohort 7 or Cohort 8.

DocuSign Envelope ID: A0702825-E0BD-46DB-A7D6-FEF901E11877

	If the decision will be to progress to Part B, the dose of Temferon to be evaluated will be determined from data obtained in Part A. Data from sequential patients will be reviewed in an ongoing manner to evaluate whether the Temferon dose should be modified for subsequent patients recruited according to the benefit:risk determination. Data to be used for this evaluation includes:<br>• Safety data according to CTCAE criteria<br>• Routine clinical and laboratory data<br>• *In vivo* engraftment of gene modified cells based on bone marrow and peripheral blood VCN<br>• Assessment of autoimmune manifestations attributed to use of Temferon<br>• Significant organ toxicities (≥ grade 3 according to CTCAE v5.0) that can be ascribed to IFN exposure that are attributed to Temferon exposure<br>• Extra-tumor detection of IFN-α gene signatures (PB, BM, CSF) and IFN-α concentrations (PB and CSF)<br>• Tumor specific criteria (imaging parameters, immunohistochemistry and IFN-α gene signatures obtained from samples at second surgery).
**Management of potential side effects related to Temferon:**	The cell based therapeutic approach that is being utilized in this study is designed to allow local, low dose IFN-α2 release at the tumor site, reducing the potential for systemic side effects. As a benchmark for a "worst case scenario", potential adverse drug reactions related to systemic IFN-α administration (well described in the Summary of Product Characteristics of approved medicines such as IntronA), are summarized below focusing on adverse reactions where warnings exist.<br>**Central Nervous System and psychiatric effects**<br>Severe central nervous system (CNS) effects, particularly depression, suicidal ideation and attempted suicide have been observed in some patients during IFN-α therapy, and even after treatment discontinuation

DocuSign Envelope ID: A0702825-E0BD-46DB-A7D6-FEF901E11877

mainly during the 6-month follow-up period. Other CNS effects including aggressive behaviour (sometimes directed against others such as homicidal ideation), bipolar disorders, mania, confusion and alterations of mental status have been observed with alpha interferons. Patients in this study should be closely monitored for any signs or symptoms of psychiatric disorders and will be reviewed periodically by a psychiatrist. If such symptoms appear, the potential seriousness of these undesirable effects must be borne in mind by the Investigator and the need for adequate therapeutic management should be considered. If psychiatric symptoms persist or worsen, or suicidal or homicidal ideation is identified, psychiatric intervention should occur as appropriate.

Hypersensitivity reactions

Acute hypersensitivity reactions (e.g., urticaria, angioedema, bronchoconstriction, anaphylaxis) to IFN-α have been observed rarely. If such a reaction develops in this study, institute appropriate medical therapy.

Abnormal liver function and prolongation of coagulation markers

IFN-α may increase the risk of hepatic decompensation and death in patients with cirrhosis. Regular monitoring of liver function tests will occur in this study. Any patient developing liver function abnormalities during treatment with IFN-α must be monitored closely. Elevation of alanine aminotransferase (ALT) $\geq$ 2 x upper limit of normal (ULN) should result in monitoring of ALT, prothrombin time, alkaline phosphatase, albumin and bilirubin every 2 weeks.

Myelosuppression

Clinically significant laboratory abnormalities, most frequently occurring at high systemic doses of IFN-α, include reduction in granulocyte and white blood cell counts, decreases in hemoglobin level and platelet count. Moderate and usually reversible pancytopenia has

been reported. Regular monitoring of complete blood counts will occur in this study.

Hypotension & need for adequate hydration

Hypotension may occur during IFN-α therapy and may require supportive treatment. Adequate hydration must be maintained since hypotension related to fluid depletion has been seen in some patients. Fluid replacement may be necessary.

Pyrexia

While pyrexia may be associated with the flu-like syndrome reported commonly during interferon therapy, other causes of persistent pyrexia must be ruled out.

Pulmonary conditions

Pulmonary infiltrates, pneumonitis, and pneumonia, occasionally resulting in fatality, have been observed rarely in IFN-α treated patients. The aetiology has not been defined. Regular monitoring of lung function via chest X-ray, lung function tests and diffusion capacity will occur in this study. Any patient developing pyrexia, cough, dyspnea or other respiratory symptoms must have a chest X-ray taken. If the chest X-ray shows pulmonary infiltrates or there is evidence of pulmonary function impairment, the patient is to be monitored closely. Treatment with corticosteroids may assist with the resolution of pulmonary adverse events.

Ocular adverse events

Ocular adverse events including retinal hemorrhages, cotton wool spots, serous retinal detachment, and retinal artery or vein obstruction have been reported in rare instances after treatment with IFN-α. All patients will undergo regular eye examinations using slit lamp ophthalmoscopy. Any patient complaining of changes in visual acuity or visual fields, or

DocuSign Envelope ID: A0702825-E0BD-46DB-A7D6-FEF901E11877

reporting other ophthalmologic symptoms during treatment, must have a prompt and complete eye examination.

Obtundation, coma and encephalopathy

More significant obtundation and coma, including cases of encephalopathy, have been observed in some patients, usually elderly, treated at higher doses of IFN-α. While these effects are generally reversible, in few patients full resolution took up to three weeks. Very rarely, seizures have occurred with high doses of IFN-α.

Patients with pre-existing cardiac abnormalities

Patients with significant cardiac abnormalities based on medical history, clinical examination or investigations including echocardiography, are excluded from the study. Cardiac arrhythmias (primarily supraventricular) have been reported with IFN-α and these usually respond to conventional therapy.

Hypertriglyceridemia

Hypertriglyceridemia and aggravation of hypertriglyceridemia, sometimes severe, have been observed. Monitoring of lipid levels will therefore occur in this study.

Patients with psoriasis and sarcoidosis

IFN-α may exacerbate pre-existing psoriatic disease and sarcoidosis. Patients with these conditions are excluded from participation in this study.

Kidney and liver graft rejection

Preliminary data indicates that IFN-α therapy may be associated with an increased rate of kidney graft rejection. Liver graft rejection has also been

DocuSign Envelope ID: A0702825-E0BD-46DB-A7D6-FEF901E11877

	reported. Patients with kidney or liver transplants are ineligible for the study and are excluded from this study.<br><br>**Auto-antibodies and autoimmune disorders**<br><br>The development of auto-antibodies and autoimmune disorders has been reported during treatment with IFN-α. Patients predisposed to the development of autoimmune disorders may be at increased risk. Auto-antibodies will be carefully monitored for the duration of the study.<br><br>**Thyroid disorders**<br><br>Infrequently, adult patients treated with IFN-α developed thyroid abnormalities, either hypothyroidism or hyperthyroidism. In clinical trials using IntronA therapy, 2.8% patients overall developed thyroid abnormalities. The abnormalities were controlled by conventional therapy for thyroid dysfunction. The mechanism by which IFN-α may alter thyroid status is unknown. Thyroid function will be monitored throughout the study and any thyroid abnormality detected during the study will be treated with conventional therapy. Discontinuation of IFN-α therapy has not reversed thyroid dysfunction occurring during treatment.<br><br>**Failure to achieve hematological recovery or severe and persistent side effects related to Temferon**<br><br>In the event that hematological recovery does not occur or patients have severe adverse events related to IFN-α, an autologous stem cell transplant using non-manipulated cells should be considered. All patients will retain a non-manipulated, autologous back-up of at least 2.0 x10^6 $CD34^+$ cells/kg which may be administered as rescue therapy in the form of an autologous stem cell transplant following ablation of genetically modified cells using high dose melphalan.
**Sample Size:**	Part A: up to 27 patients (8 cohorts, up to 3 patients per cohort, and an additional 3 patients prior to progression to Part B)

DocuSign Envelope ID: A0702825-E0BD-46DB-A7D6-FEF901E11877

	Part B: sample size will be defined once the data generated in Part A will be analysed.
**Data Monitoring Committee (DMC):**	An independent DMC constituted of a minimum of 3 members (2 hematologists and 1 neuro-oncologist) and up to 5 members (3 hematologists and 2 neuro-oncologists) with relevant medical and scientific expertise will be convened before the study start. A charter describing the composition and conduct of the DMC will be issued by the Sponsor and agreed to by all DMC members prior to the DMC's initial meeting. The names of the DMC members and the charter will be sent to the ethics committee before the first patient enters the study.<br><br>The DMC will review safety and efficacy data within a dosing cohort, together with cumulative safety data from preceding cohorts, and make a recommendation about proceeding to the next dosing cohort. Should there be insufficient Temferon engraftment in the bone marrow of the first 2 patients in a cohort (i.e. VCN < 0.07), the DMC will review the safety and efficacy data for the 2 subjects and make a recommendation about proceeding to the next dose level.<br><br>The dose escalation recommendation and minutes from the open session of the DMC meeting will be sent to the ethics committee, after each DMC meeting. In addition, a summary of the data reviewed by the DMC at completion of Part A, will be sent to the ethics committee before commencement of Part B, if any.
**Enrolment suspension criteria:**	In the event that a single death occurs within 90 days of Temferon administration that is attributed to Temferon, further enrolment will be suspended. All other SAEs that result in death and are attributed to Temferon, will be reviewed by the DMC who will advise on whether recruitment into the study should be suspended.
**Statistical Methods:**	This is a Phase I/IIa gene therapy study for which no formal statistical analysis is required. However, descriptive analyses will be performed on

DocuSign Envelope ID: A0702825-E0BD-46DB-A7D6-FEF901E11877

	all data collected in this study to gain further insight on safety and preliminary measures of efficacy.
**Date of Protocol:**	21st October 2022

DocuSign Envelope ID: A0702825-E0BD-46DB-A7D6-FEF901E11877

List of Abbreviations

Abbreviation	Definition
ABW	Adjusted body weight
AE	Adverse event
ALL	Acute lymphoblastic leukemia
ALT	Alanine aminotransferase
ATMP	Advanced therapy medicinal product
AUC	Area under the plasma concentration curve
BM	Bone marrow
CB	Cord blood
CFC	Colony forming cell
cGMP	Current Good Manufacturing Practice
CMO	Contract manufacturing organization
CMP	Common myeloid progenitor
CNS	Central Nervous System
CRC	Colorectal cancer
CRF	Case report form
CSF	Cerebrospinal fluid
DC	Dendritic cells
DMC	Data monitoring committee
DMSO	Dimethylsulfoxide
ECOG	Eastern Cooperative Oncology Group Performance Status
EMA	European Medicines Agency
EORTC	European Organization for Research and Treatment of Cancer
GCP	Good Clinical Practice
GFP	Green fluorescent protein
GrMP	Granulocyte and monocyte progenitor
GMP	Good Manufacturing Practice
gRV	Gamma retrovirus
HIV	Human immunodeficiency virus
HR	High risk
HSC	Hemopoietic stem cell
HSCT	Hemopoietic stem cell transplantation

DocuSign Envelope ID: A0702825-E0BD-46DB-A7D6-FEF901E11877

Abbreviation	Definition
HSPC	Hemopoietic stem and progenitor cell
IBW	Ideal body weight
ICF	Informed consent form
ICH	International Council for Harmonisation
IFN-α	Interferon alpha
INCB	Istituto Neurologico Carlo Besta
JACIE	Joint Accreditation Committee-ISCT & EBMT
KPS	Karnofsky Performance Status
LCMV	Lymphocytic choriomeningitis virus
LTR	Long terminal repeat
LV	Lentivirus
MEP	Megakaryocytic erythroid progenitors
MGMT	O6-methylguanine-DNA methyltransferase
MHC	Major histocompatibility complex
MIL	Marrow infiltrating lymphocytes
MLD	Metachromatic leukodystrophy
MOI	Multiplicities of infection
MPB	Mobilized peripheral blood
MRI	Magnetic resonance imaging
NK	Natural killer
NSG	NOD scid gamma
OS	Overall survival
OSR	Ospedale San Raffaele
OVA	Ovalbumin
PB	Peripheral blood
PD	Progressive disease
PFS	Progression free survival
PG	Policlinico Universitario Fondazione Agostino Gemelli
PGK	Phosphoglycerate kinase
PR	Partial response
PTI	Post tumor injection
QP	Qualified person
qPCR	Quantitative polymerase chain reaction

Abbreviation	Definition
RCL	Replication competent lentivirus
RCR	Replication competent retrovirus
RPA	RNase protection assay
SCID	Severe combined immunodeficiency
SO2	Oxygen saturation
SOM	Study operations manual
SR-TIGET	San Raffaele Telethon Institute for Gene Therapy
TAMs	Tumor associated macrophages
TDM	Therapeutic drug monitoring
TEMs	Tie2 expressing monocytes
Temferon	Autologous $CD34^+$ hematopoietic progenitor cells genetically modified with the TIA126-LV, encoding for the interferon-α2 gene
Tie2	Receptor tyrosine kinase with immunoglobulin and epidermal growth factor homology domain
TMZ	Temozolomide
ULN	Upper limit of normal
UTMO	Bone Marrow Transplantation Unit Ospedale San Raffaele
VCN	Vector copy number
WAS	Wiscott-Aldrich Syndrome
WHO	World Health Organization

DocuSign Envelope ID: A0702825-E0BD-46DB-A7D6-FEF901E11877

1 Introduction

1.1 Sponsor and product background

Genenta Science is an Italian biotechnology company founded in 2014 as an Ospedale San Raffaele (OSR) spin-off. OSR is a leading center for *ex-vivo* gene therapy using autologous hematopoietic stem and progenitor cells (HSPCs) genetically modified and re-administered to the patient in the context of a stem cell transplant. The San Raffaele Telethon Institute for Gene Therapy (SR-TIGET), a joint venture between OSR and the Telethon Foundation, has successfully conducted several clinical gene therapy studies using *ex-vivo* lentivirus (LV)-based technology to transfer a variety of transgenes into HSPCs in the field of monogenic diseases.

The LV-based technology used to transduce HSPCs with the IFN-α2 transgene for this clinical study is well established, having been used to develop Strimvelis, the first European Union approved gene therapy for the treatment of severe combined immunodeficiency due to adenosine deaminase deficiency[1]. Additionally clinical studies are ongoing at OSR using the same LV based technology but a variety of different transgenes, including for transfusion dependent beta-thalassemia (NCT02453477), metachromatic leukodystrophy (NCT01560182) and Wiskott-Aldrich syndrome (NCT01515462)[2–4]. As of March 2018, more than 50 patients with metachromatic leukodystrophy, Wiskott-Aldrich syndrome or transfusion-dependent beta thalassemia have been treated at OSR with lentivirally-transduced HSPCs. The benefit-risk of *ex-vivo* HSPC transduction using this specific LV-based technology continues to be highly positive and this approach minimizes the risks associated with direct infusion of transgene-carrying vectors into patients[5].

Temferon, a product consisting of autologous $CD34^+$ HSPCs transduced with the human interferon-α2 (IFN-α2) gene, builds on this experience with an *ex-vivo* HSPC gene therapy platform, and extends its application to the field of cancer. The immune system has a natural capacity to locate and destroy abnormal cells and the mechanisms by which tumor cells escape immune-surveillance have been the focus of scientific research for decades[6–8]. The fruits of such efforts are finally reaching patients, and there are several immunotherapy approaches achieving encouraging results. Based on more than 10 years of work in the laboratories of Professor Naldini and Dr. Gentner (OSR), a gene therapy strategy (Temferon), has been developed that could potentially inhibit patients' tolerance to tumor cells by exploiting a subset

DocuSign Envelope ID: A0702825-E0BD-46DB-A7D6-FEF901E11877

of circulating and tumor-infiltrating monocytes expressing the angiopoietin receptor Tie2. Our cancer gene therapy platform relies on the selective and local targeting of IFN-α2 to the tumor, achieved by bone marrow-derived myeloid cells characterized by TIE2 promoter activation, hereafter referred to as Tie2 expressing monocytes (TEMs). In order to reduce off-target and systemic effects, we exploit the endogenous activity of a specific microRNA, miR-126, that is strongly expressed in HSPCs, but down-regulated in the differentiated progeny. Adding recognition sequences for this endogenously expressed miR into the vector construct allows suppressing IFN-α expression specifically in the primitive HSPC compartment. By this novel strategy of tumor-selective IFN-α delivery by TEMs, antitumor activity has been demonstrated in several orthotopic, spontaneous, and metastatic pre-clinical animal models of oncogenesis [9–11].

The clinical study described in this protocol is the first to be conducted with Temferon in patients with glioblastoma multiforme (GBM) and will evaluate the safety, feasibility and biological activity of Temferon in patients where GBM cells have an unmethylated O-6-methylguanine-DNA methyltransferase (MGMT) gene promoter. The study will be conducted within a framework that potentially could provide therapeutic benefit that will be documented using widely accepted secondary endpoints. The experimental treatment is embedded in a treatment program delivering state-of-the-art therapy to all enrolled patients. Our intent is to potentially deliver therapeutic efficacy in the first patients treated in order to justify the risks associated with administration of a pre-transplant conditioning regimen and the first-in-human exposure to the investigational advanced therapy medicinal product (ATMP).

1.2 Glioblastoma multiforme and existing treatment modalities

1.2.1 The disease burden and biological features of glioblastoma

GBM is the most aggressive diffuse glioma of astrocytic lineage and is considered a grade IV glioma based on the World Health Organization (WHO) classification[12]. GBM is the most common malignant primary brain tumor making up 54% of all gliomas and 16% of all primary brain tumors[13]. Untreated, the median survival is only 3 months[14] and GBM remains an incurable tumor with a median survival of approximately 13 months[15].

The defining biological feature for GBM is a massive tumor cell infiltration. The neoplastic cells have the ability to travel long distances from the place of origin by tracking white matter

DocuSign Envelope ID: A0702825-E0BD-46DB-A7D6-FEF901E11877

structures. They may travel in the opposite hemisphere and may give rise to additional tumor foci (the so-called butterfly GBM). Due to this biological behavior, a complete microscopic resection can never be achieved, and remnant neoplastic cells are frequently the source for disease recurrence. Histologically, glioblastoma is composed of pleomorphic cells, mitotic activity, intravascular microthrombi, necrosis with or without cellular pseudopalisading, and/or microvascular proliferation[16].

GBMs can be classified into primary and secondary; primary GBM occurs de novo without evidence of a less malignant precursor, whereas secondary GBM develops from initially low-grade diffuse astrocytoma (WHO grade II diffuse astrocytoma) or anaplastic astrocytoma (Grade III). The majority of GBMs (90%) are primary, and patients with primary GBM tend to be older (mean age 55 years) than those with secondary GBM (mean age 40 years)[17]. GBMs have significant genetic heterogeneity and different tumor molecular subtypes characterized by distinct genetic alterations with a prognostic significance have been identified[18,19]. Genetic alterations that occur more frequently with primary GBM are EGFR overexpression, PTN mutation, and loss of chromosome 10, whereas genetic alterations more commonly seen in secondary GBM include IDH1 mutations, TP53 mutations, and 19q loss[20–22]. The classification of GBM based on gene expression also distinguishes between four subtypes including proneural, neural, classical, and mesenchymal[18,19].

In GBM genetic abnormalities translate into morphological changes such as infiltration, necrosis with pseudopalisading, and microvascular proliferation responsible for accelerated tumor growth and aggressive behavior. At the center of these morphological changes sit prothrombotic events that contribute to glioblastoma progression[23]. Unlike other cancers where inflammation plays an important role in promoting tumor development, GBM is relatively immunodeficient with any inflammation observed likely driven by tumor cell genetic alterations, changes in the tumor microenvironment, or by responses to therapy (e.g. necrosis following chemotherapy or radiotherapy accompanied by release of cytokines)[16].

GBM is a complex and heterogeneous disease encompassing a series of tightly interconnected cell signaling networks and it is likely that specific targeted therapies will be efficacious in molecularly defined subsets. The molecular classification of GBM based on the identification of markers that define subsets may be helpful in determining the utility of specific therapies.

1.2.2 The prognostic role for monocytes in GBM

Despite multimodal therapeutic intervention in GBM, clinical outcomes remain poor which is likely related to tumor heterogeneity, diffuse tumor cell infiltration into the normal brain parenchyma and the presence of a highly immunosuppressive tumor microenvironment (TME)[24]. The TME in gliomas is composed of many non-cancerous cell types[25] and a comprehensive analysis of immune infiltrate in GBM patients revealed that tumor-associated macrophages (TAMs) and microglia, the brain resident macrophages, are the major immune cell constituent representing up to 40% of immune cells in GBM[25,26] with monocytes/macrophages being more abundant compared to resident-microglia [27].

In experimental models, bone marrow (BM) transplantation can be used to distinguish the relative contribution to TME from both microglia and BM-derived macrophages from the peripheral circulation. In a murine GBM model, it was shown that BM-derived cells, containing Tie2$^+$, VEGFR1$^+$, CD11b$^+$, and F4/80$^+$ subpopulations, as well as endothelial and pericyte progenitor cells were actively recruited to the tumor in a HIF-1α/SDF-1-dependent manner[28]. Also in humans, there is compelling evidence that TAMs promote malignant glioma growth by creating a local immunosuppressive microenvironment, by secreting pro-angiogenic factors, by enhancing tumor cells invasion through the production of soluble factors such as transforming growth factor-β (TGF-β), interleukin-10 (IL-10), vascular endothelial growth factor (VEGF), and matrix metallopeptidase-9 (MMP-9)[29–34]. It is known that macrophages may differentiate into a range of phenotypes, conventionally classified according to two major polarization programs, M1 and M2[35,36]. The classically activated or M1-polarized macrophages are characterized by a pro-inflammatory phenotype and are responsible for the induction of Th1-mediated immune responses. On the other hand, M2-macrophages, which exhibit anti-inflammatory properties lead to an immunosuppressive state by enhancing the expression and the secretion of immunosuppressive cytokines, such as transforming growth factor TGF-β and IL-10 [37].

The extent of TAM infiltration has been associated with poor prognosis in patients with breast, prostate, bladder or cervical cancers[37–40]. In glioblastoma, the number of TAMs expressing markers of M2/alternative macrophage activation is higher compared to that observed in low-grade gliomas, and correlates with the vascular density within the tumor[41], increased tumor invasiveness, higher proliferation rates and poorer prognosis[42,43]. Accordingly, the frequency

DocuSign Envelope ID: A0702825-E0BD-46DB-A7D6-FEF901E11877

of circulating monocytes was higher in glioblastoma patients relative to healthy donors and patients with lower grade gliomas[44].

1.2.3 Treatment approaches for patients with newly diagnosed GBM

Surgery is the initial therapeutic approach for GBM and remains the standard of care in the treatment of malignant brain tumors. A more extensive surgical resection is associated with longer life expectancy, with the longest survival achieved in those patients who undergo total macroscopic resection followed by radiotherapy and temozolomide (TMZ). Data from a pivotal phase 3 trial showed an overall survival (OS) and progression free survival (PFS) improvement with the combination of radiotherapy and TMZ relative to radiotherapy alone (median OS 14.6 vs. 12.1 months; $P < 0.001$)[45]. These results were supported by other trials[46–49] and a meta-analysis revealed a median OS of 13.4–19.0 months in the combination treatment group, as opposed to 7.7–17.1 months in the radiotherapy group alone[50].

A number of additional factors have been identified as prognostic factors in GBM patients including age, neurological status , extent of surgical resection and methylation of the MGMT promoter region within the tumor[51,52]. MGMT is involved in DNA repair of O6-alkylating agents, including TMZ. MGMT promoter methylation has prognostic and predictive significance in patients with GBM, with longer survival rates in newly diagnosed patients treated with radiation and TMZ and subsequent adjuvant TMZ[53]. Unmethylated MGMT promoter status is identified in approximately 60% of GBM but despite the evidence suggesting that this group of patients do not benefit from adjuvant therapy with TMZ, the lack of alternative options means that these patients often receive TMZ in routine clinical practice. This approach may also be associated with harm, with worse outcomes in some patients with unmethylated MGMT compared to treating with radiotherapy alone [14,51,54]. Adding a new drug or agent on top of the previously established combined modality regimen may cause undue toxicity or drug interaction, thus requiring dose reduction and treatment with potentially subtherapeutic doses. Therefore in both recently published as well as in currently ongoing clinical studies in GBM patients with unmethylated MGMT, the standard approach is to omit TMZ in groups receiving investigational medicinal products[55–64].

There is therefore a need for new therapeutic options for patients with GBM and unmethylated GBM promoter. Reprogramming of the tumor microenvironment together with direct effects

DocuSign Envelope ID: A0702825-E0BD-46DB-A7D6-FEF901E11877

on tumor cells and neo-angiogenesis via a cell mediated IFN-α approach is an attractive proposition for further study.

1.3 Rationale for gene therapy approach in GBM

1.3.1 Tie2 expressing monocytes

A subset of circulating and tumor-infiltrating BM-derived monocyte/macrophages characterized by the expression of the angiopoietin receptor Tie2 has been described in mice[28,65–72]. These Tie2-expressing macrophages (TEMs) have features of M2-polarized, tumor-associated macrophages (TAMs)[73], promote both developmental and tumor angiogenesis[74,75], are required for the formation of tumor blood vessels[70,71] and are infrequently found in normal organs. Notably, the Tie2 receptor is weakly expressed by the circulating monocytes, but is significantly upregulated upon their homing to tumors[9,70,71].

Several studies indicated the occurrence of a population of monocyte/macrophages with corresponding features and activity also in humans[11,76–85]. Human TEMs are often found at higher frequency in the blood of tumor-bearing individuals and colonize the stroma of many tumors, including the group of hematologic malignancies such as multiple myeloma[86].

Several studies have shown that tumor infiltration by M2-polarized TAMs (including TEMs) is a negative prognostic factor, highlighting a likely role of these cells in fostering tumor invasion and growth, conceivably by promoting angiogenesis and suppressing anti-tumor immunity, suggesting that recruitment of these M2 macrophages to the tumor may be an obligate step for tumor progression and may represent a novel target for anti-tumor therapies[77,87–90]. In this regard, TEMs represent a suitable carrier for the local and tumor-restricted release of an anti-tumor molecule thus enabling to combine the maximum therapeutic efficacy with a consistent reduction in the potential systemic toxicity. Notably, we demonstrated that it is possible to achieve the selective expression of IFN-α in mouse and human TEMs by transducing HSPCs with a third-generation lentiviral vector in which we cloned the mouse/human Tie2/TEK enhancer/promoter sequence[9,10]. More recently, we improved the vector design for IFN-α gene therapy by adding target sequences recognized by microRNA-126 (miR-126) to the construct. This miRNA, being endogenously expressed to high levels in HSPCs and down-regulated in the relative differentiated progeny[91], gave us the possibility to combine a robust suppression of the transgene expression in human and mouse

DocuSign Envelope ID: A0702825-E0BD-46DB-A7D6-FEF901E11877

HSPCs with a strong expression in the differentiated progeny[10,92] thus reducing the risk of possible detrimental effects on the stem/progenitors cells.

1.3.2 Relevance of human Tie2-expressing macrophages in sustaining glioma progression

The first evidence of TEMs' role in sustaining glioma development and progression was published in 2005[71]. Murine Lin negative BM-derived cells, the equivalent of human $CD34^+$ cells which were either BM or mobilized peripheral blood (MPB)-derived, were transduced with the Tie2-Green Fluorescent Protein (GFP) or the ubiquitously expressed PGK-GFP LV and transplanted into nude mice to obtain respectively Tie2-GFP and Phosphoglycerate Kinase (PGK)-GFP mice. Eight weeks after the hematopoietic reconstitution mice were orthotopically injected with a glioblastoma-derived cancer stem cells line[93] that mimics a number of malignant traits of human GBM that may not be assessable in other models [93,94].

In brain tumors grown for 6 weeks in PGK-GFP mice, more than 85% of the monocyte/macrophage cells were GFP positive, whereas in the brain parenchyma only a small fraction of these cells was GFP positive. Moreover, within the tumors, all the GFP positive cells were hematopoietic ($CD45^+$). These findings underlined the recruitment of BM-derived macrophages into the tumor. In the brain of Tie2-GFP mice, TEMs were only located within the tumor, were never found in the non-infiltrated brain parenchyma and represented on average 9% of the total hematopoietic cells ($CD45^+$). Notably, the majority of TEMs expressed basic fibroblast growth factor (bFGF), a potent proangiogenic molecule, suggesting that TEMs might promote angiogenesis via the release of bFGF, and possibly other proangiogenic factors.

Angiogenesis is one of the key events in GBM development, and the histological diagnosis of GBM relies also on the presence of microvascular proliferation[95]. The vascularization in GBM is markedly higher compared to lower grade gliomas and is associated with worse prognosis[96]. One of the most potent activator of neo-angiogenesis in developing GBM is tissue hypoxia, which, through the stabilization of hypoxia-inducible factors (HIFs), regulates the expression of several pro-angiogenic genes, including VEGF, Ang-1, Ang-2, and Tie-2. While the binding to Ang-1 to Tie-2 promotes the survival of endothelial cells and stabilized their interaction with vascular pericytes, the binding of Ang-2 to Tie2 antagonizes Ang-1 binding and destabilizes endothelial cells from vessels wall, thus triggering the first and obligate step of new blood vessel sprouting[97]. Notably, Tie2 expression is increased in the vasculature of gliomas, its

DocuSign Envelope ID: A0702825-E0BD-46DB-A7D6-FEF901E11877

levels correlate with increasing tumor grade, and its inhibition results in decreased tumor growth in animal models[98–100]. Hypoxia is also responsible for Tie2 upregulation on TEMs [67,101].

To assess whether TEMs play a critical role during the angiogenic phase of glioma development we orthotopically injected the human U87 glioma cells[102] into the brain striatum of nude mice, which were previously transplanted with Tie2-thymidine-kinase transgenic BM-derived Lin-negative cells. The U87 glioblastoma cell line is among the most extensively employed GBM cell line models, and its molecular profile reveals that it belongs to the glioblastoma mesenchymal subtype (Galli et al., in preparation). The mesenchymal subtype is common in older adults, as the name suggests has mesenchymal differentiation, is associated with worse outcome, and is characterized by abnormal EGFR amplification/PTEN loss/NF1 mutations/Akt signaling [16].

One day after the injection of the U87 tumor cells, mice were randomly sorted into two groups which were either untreated or treated with ganciclovir daily for 12 days in order to achieve a thymidine kinase/ganciclovir-mediated "suicide" of TEMs. Consistent with the gliomas ability to initially grow by co-opting the preexisting vasculature, the destruction of TEMs using ganciclovit did not prevent tumor onset. However, ganciclovir treated tumors were on average smaller than the untreated tumors and displayed a markedly reduced contrast uptake, with mixed hyperintense and hypointense signal, indicating impaired perfusion and widespread cell loss. Magnetic resonance imaging (MRI) volumetry indicated that tumor necrosis was dramatically increased in ganciclovir treated as compared to untreated mice (>10-fold; $p < 0.02$). Accordingly, the hematoxylin/eosin staining of brain sections showed that ganciclovir treated gliomas were mostly made of nonviable tissue. Extensive necrosis was also prevalent at the tumor margins, a finding in sharp contrast with the untreated tumors. Moreover, the proliferation index of the ganciclovir treated tumors was extremely low, both in the inner tumor mass and at the tumor margins. Furthermore, ganciclovir treated tumors were almost avascular, whereas those of untreated mice were highly angiogenic. Interestingly, the rare blood vessels observed in ganciclovir treated tumors had regular shape and small diameter, were covered by pericytes, and thus likely represented residual coopted vessels. By contrast, the blood vessels of control tumors were abnormally enlarged and tortuous, contained many proliferating endothelial cells, and had loosely associated pericytes, all typical features of neo-angiogenesis.

Taken together, these data strongly suggest that gliomas of ganciclovir treated mice were regressing at the time of analysis and that the selective TEM ablation severely impaired the angiogenic phase in orthotopic gliomas. Ganciclovir effectively cleared GFP^+ TEMs from tumors but did not inhibit the recruitment of macrophages and granulocytes, indeed the large necrotic areas of treated tumors were heavily infiltrated by these scavenging cells. This observation indicated that tumor-infiltrating macrophages and granulocytes were not able to locally reconstitute the TEM cell pool or surrogate their function, supporting the notion that TEMs are a distinct proangiogenic lineage of HSPCs required for tumor vascularization.

After showing that mouse TEMs play a pivotal role in sustaining glioma progression, we investigated whether human TIE2 monocytes have pro-angiogenic activity[76]. To this end, we isolated $CD14^+TIE2^+$ and $CD14^+$TIE2- cells from human peripheral blood by cell sorting and co-injected these cells in increasing ratios with U87 human glioma cells subcutaneously in nude mice. As controls, we injected U87 cells alone and U87 cells together with unfractionated CD14 monocytes, which mostly comprise inflammatory monocytes. We assessed the tumor vascularization by analyzing CD31 expression 7 days after tumor injection.

The blood vessels within the inner tumor mass were scarce in animals injected with U87 alone, with few large blood vessels surrounding the tumors indicating that the neo-angiogenesis had not yet started at this early stage of growth. Tumors co-injected with unfractioned $CD14^+$ monocytes or $CD14^+$TIE2- were characterized by a small rim of blood vessels lining the tumor periphery, with a few spots of neo-angiogenic vessels within the inner mass. On the other hand, blood vessels of tumors co-injected with human $CD14^+TIE2^+$ monocytes had the typical morphology of neo-angiogenic vessels being large and tortuous. Moreover, these tumors were much more vascularized, with a profuse vascular framework extending from the tumor periphery toward the inner mass. The overall vascular area was significantly greater in tumors co-injected with $CD14^+TIE2^+$ monocytes than in control tumors and tumors co-injected with unfractioned CD14 cells or CD14 cells depleted of TIE2 positive monocytes.

These results indicate that among human blood monocytes, the $CD14^+TIE2^+$ subset was specifically endowed with the ability to enhance angiogenesis in a human tumor transplantation model relevant for GBM[76]. By utilizing TEMs, it is feasible to transport an immunomodulatory cytokine directly to growing GBM tumors and modulate the effects of TEMs and other cells. Data from preclinical studies using such an approach are discussed in Section 1.3.5.

DocuSign Envelope ID: A0702825-E0BD-46DB-A7D6-FEF901E11877

The totality of data supports the progression of a TEMs based gene therapy approach in GBM.

1.3.3 Interferons as anti-cancer drugs and our new gene delivery strategy

Type I interferons (IFN-α and -β) are highly pleiotropic cytokines discovered more than half a century ago as the key factors underlying viral interference[103]. Since their discovery, a variety of biological effects, in addition to the antiviral activity, have been described for IFNs, including antitumor effects, inhibition of angiogenesis and the modulation of the activity of several types of cells of the immune system[104]. Among the IFNs, IFN-α has the longest record of clinical use. It has been widely used and approved for the treatment of many types of cancer, including hematological malignancies, and selected solid tumors, such as malignant melanoma, metastatic renal-cell carcinoma, neuroendocrine tumours, ovarian carcinoma, AIDS-related Kaposi's sarcoma and severe, life-threatening hemangiomas[104–109]. While response rates to IFN therapy were low, a subset of patients was cured by this treatment, an outcome which is still hardly achievable even with the latest targeted agents[110]. As an example, about 15% of metastatic renal-cell carcinomas respond to treatment with IFN-α alone [111]. Despite the observation of tumor responses, however, the use of IFN-α has recently been significantly reduced because of the substantial toxicity observed upon systemic administration and the availability of alternative, targeted anticancer drugs offering a wider therapeutic window.

Our renewed interest in IFN as an anticancer treatment is based on the following aspects: **(1) Cell-based, specific delivery of IFN-α into the tumor microenvironment by TEMs is expected to relieve systemic symptoms**, overcoming the tolerability issues associated with systemic IFN treatment; **(2) IFN-α deployed locally in the tumor microenvironment may allow full exploitation of its pleiotropic anti-tumor activities** while limiting systemic adaptation to chronic IFN stimulation and immunoparalysis potentially associated to this phenomenon. We see a unique opportunity to overcome tolerability issues by administering HSPCs engineered to express IFN-α2 selectively in their monocyte/macrophage progeny, thus achieving a stable IFN-α source in the tumor which allows to exploit its anti-tumor activity and to avoid systemic toxicity.

Mechanisms of IFN anti-tumor activity

Concerning the mechanisms of antitumor activity, type I IFNs were long thought to act mainly by suppressing tumor cell proliferation and angiogenesis[112,113]. These cytokines can directly

DocuSign Envelope ID: A0702825-E0BD-46DB-A7D6-FEF901E11877

promote the apoptosis of tumor and stromal cells by the induction of proapoptotic genes and proteins or by the repression of antiapoptotic genes. Moreover, they may inhibit angiogenesis by down-regulating the expression of proangiogenic factors in tumors[105]. More recently, it has been established that IFN-α and -β also have important roles in regulating the innate and adaptive arms of the immune system, and recent studies have further shown that IFN responsiveness in the host hematopoietic compartment is necessary and sufficient to evoke antitumor activity, suggesting that the crucial targets of IFN responses are the host cells rather than the tumor cells[114]. IFN-α stimulates early innate immune responses and the subsequent adaptive immune response via multiple pathways and mechanisms, including: maturation and cross-priming capacity of dendritic cells (DCs); upregulation of the expression of tumor-associated surface antigens and major histocompatibility complex (MHC) class I molecules on tumor cells[115] and of MHC class I and II molecules on DCs[116], enhanced priming and survival of T cells; enhanced humoral immunity; increased cytotoxic activity of natural killer (NK) cells and macrophages, control of helper T-cell population balance (Th1=Th2), immunoglobulin class switching of B cells[117,118] and the regulation of $CD8^+$ cytotoxic T-lymphocyte responses[105]. Moreover, type I IFNs increase the susceptibility of target cells (both tumor and stromal cells) to the cytotoxic activities of immune effectors. Through these immunomodulatory functions, type I IFNs increase tumor immunogenicity, recruit and activate immune cells within the tumor milieu, and may eventually facilitate tumor rejection[114,119–123]. Notably, accumulating evidence indicates that the success of conventional chemotherapeutics, targeted anticancer agents, radiotherapy and immunotherapy relies on type I IFN signaling in malignant cells, tumor-infiltrating myeloid cells or within lymphoid organs[103]. Thus, IFNs appear to be a downstream effector pathway common to many currently applied anticancer treatments, and exogenously targeting these potent antitumor molecules to the right place, at the right dose and at the right time may potentiate anti-tumor responses, thereby increasing cure rates.

Importantly, in preclinical studies we have shown that our cell- and gene therapy delivery strategy may engage nearly all the above described activities of IFN at the tumor site, according to the tumor type. We have documented direct inhibition of tumor cell growth, inhibition of neo-angiogenesis, enhanced recruitment and activation of innate immune cells such as macrophages, dendritic cells and NK cells, and recruitment and activation of T-cells, including the priming and effective deployment of CD8 T-cells specifically recognizing cancer-associated antigens that play a dominant role in establishing tumor clearance (Escobar *et al.*, manuscript under revision for publication).

DocuSign Envelope ID: A0702825-E0BD-46DB-A7D6-FEF901E11877

1.3.4 IFN-α and GBM

Systemically or locally administered IFN-α is not currently standard of care in the management of patients with GBM although a large number of historical publications have reported clinical outcomes in patients with malignant brain tumors including GBM where they were treated with IFN-α injected either directly into the tumor or administered parenterally[124–142]. Most of these were cohort studies performed in small numbers of patients. Furthermore, the patients included in the studies varied according to tumor histology, early versus recurrent disease, degree of surgical resection of the tumor, use of radiotherapy, coadministration of cytotoxic agents, and route of IFN-α administration. A large phase 3 trial comparing the combination of IFN-α and carmustine compared to carmustine alone following radiotherapy in patients with newly diagnosed high grade glioma, showed no difference between the groups in overall survival or progression free survival[142]. However, a higher rate of withdrawal from the study due to poor tolerability was noted in patients treated with the combination. More recently two small single-arm studies evaluated the efficacy of IFN-α in combination with temozolomide in patients with recurrent disease[140]. The data were encouraging with 53-57% of patients achieving stable disease, 6 months progression free survival observed in 31-38% of patients and median overall survival of 7.2-10 months. In a large cohort with recurrent disease, many of whom were treated with radiosurgery and second line agents such as nitrosourea based chemotherapy, temozolomide, mitoxantrone, fotemustine and bevacizumab, median survival was 8.9 months[15].

The potential importance of IFN-α in GBM is also highlighted by other mechanistic data. Retrospective clinical analyses have suggested that clinical response to IFN-α treatment may be inversely correlated to IFN-α gene content in the tumor and response to IFN-α treatment[143]. Furthermore, the deletion of the IFN gene locus (chromosome 9p) has been reported both in glioma tissue samples and in glioma cell lines and may be associated with high grade tumors[144–147]. Tumors may be both hemi- or nullizygous for interferon but nullizygosity occurs exclusively among high-grade gliomas (e.g. glioblastoma) and with high frequency (>40%)[146,147]. This last observation suggests that IFN locus loss is linked to the acquisition of more malignant traits and that a possible alternative source of IFN which minimizes systemic toxic effects and is used in combination with current standard of care, might be effective in GBM treatment.

DocuSign Envelope ID: A0702825-E0BD-46DB-A7D6-FEF901E11877

Increasing evidence suggests that a high tumor mutation and neo-antigen burden as well as defects in the DNA repair machinery and genomic instability correlate with the response to immunotherapy, which implies that these factors are important targets for T cells in cancer immunity[148–150]. Specifically, accumulating data on glioblastoma somatic alterations achieved through whole-genome, exome, and RNA sequencing as well as copy-number, transcriptomic, epigenomic, and targeted proteomic profiling[151–153] highlighted that this type of tumor is characterized by a substantial load of the above mentioned key players in the tumor immune response[154].

Furthermore, it has also been reported that ionizing radiation, which is part of the standard of care for glioblastoma treatment[45] may be associated with immuno-modulatory properties. While low single doses impact on tumor vascularization and immune cell infiltration, higher doses were linked to the induction and production of type I interferons and both were able to trigger immunogenic cancer cell death[155]. The dispersion of radiotherapy-induced immune-stimulating tumor antigens released from dying tumor cells into the surrounding milieu[156], contributes to increasing of neo-antigen load.

1.3.5 Preclinical studies with Tie2 cassette in experimental tumor models

The non-clinical pharmacology studies supporting the clinical use of Temferon in non-glioma solid tumor and hematopoietic cancer models are described in detail in the Investigator Brochure.

Briefly, the expression pattern of the Tie2-driven vector cassette in the hematopoietic compartment after hematopoietic reconstitution was assessed with the help of a green fluorescent protein (GFP) marker gene. Mice reconstituted with murine HSPC transduced with the mouse Tie2.GFP lentiviral construct showed almost no GFP marking of the myeloid populations replaced in organs such as the liver, lung and brain, whereas GFP positive monocyte/macrophages were found to comprise a minor but well detectable proportion of the hematopoietic infiltrate of tumor allografts or of spontaneously grown tumors (breast cancer - PyMT mice) in the transplanted mice[70,71,157]. The selective expression of the Tie2-driven reporter gene in the hematopoietic tumor infiltrate is favored by 1) preferential contribution of the transplanted HSPCs engrafted in the bone marrow to the myeloid infiltrate of tumors as compared to the resident pools of macrophages found in other organs; 2) preferential recruitment of TEMs and/or skewed polarization towards M2 phenotype of

DocuSign Envelope ID: A0702825-E0BD-46DB-A7D6-FEF901E11877

monocyte/macrophages recruited in tumors as compared to the other organs; 3) upregulation of Tie2 promoter-driven expression in tumor-infiltrating macrophages, likely in response to cytokines, ischemia and other factors found in the tumor microenvironment. This specificity provided the basis for developing a new gene and cell-based strategy for targeted delivery of a transgene product, such as IFN-α, to tumors by HSPC gene therapy. With the limitations of lineage commitment biases and incomplete differentiation of human HSPCs in xenograft models, a myeloid-specific and monocyte-tilted expression profile, with protection of the primitive HSPC compartment by the miRNA target sequences, was observed in human cord blood (CB) cells transduced with the human Tie2.GFP.miRT construct and transplanted into NOD scid gamma mice (NSG) mice[10].

To assess whether the TEMs mediated gene therapy may be effective in counteracting glioma progression, we cloned the mouse Ifna1 gene and separately the reporter gene GFP, used as a control, in a third-generation lentiviral vector[157]. Transgene expression was controlled by the Tie2 promoter/enhancer sequences (Tie2-IFN LV or Tie2-GFP LV). As controls, the IFN gene or the reporter gene GFP were also cloned under the control of the ubiquitously active PGK promoter (PGK-IFN LV or PGK-GFP LV). Transduced HSPCs were then obtained from the bone marrow of CD1 athymic (nu/nu) mice and transplanted the cells into lethally irradiated mice to obtain mice with TEM-specific or pan-hematopoietic IFN-α or GFP expression, respectively.

As a consequence of the different promoters used to guide the transgene expression, HSPCs transduced with the Tie2-IFN LV expressed IFN-α to a much lower extent compared to PGK-IFN LV-transduced cells. HSPCs transduced with mTie2-IFNa LV showed long-term hematopoietic reconstitution ability (average vector copy number [VCN] per cell 0.6 ± 0.2) and recovered normal blood cell and platelet counts, hemoglobin and hematocrit. However, the robust and pan-hematopoietic expression of IFN-α, achieved through the ubiquitously expressed PGK promoter, caused primary graft failure resulting in overt BM aplasia and mouse death, as expected from the well-known myelotoxicity of high dose IFN, thus demonstrating the crucial importance of a moderate and selective expression of this transgene for its safe, gene therapy-based administration.

Eight weeks after HSPCs transplantation, the U87 glioma cell line was inoculated intracranially and glioma growth was monitored by MRI for up to 5 weeks post tumor injection (PTI) in Tie2-IFN mice, Tie2-GFP mice and PGK-GFP mice. At three weeks PTI, tumor

DocuSign Envelope ID: A0702825-E0BD-46DB-A7D6-FEF901E11877

volume reached 24.6 ± 4.3 mm^3 in control mice (including Tie2-GFP and PGK-GFP mice), whereas it was only 3.8 ± 2.1 mm^3 in Tie2-IFN mice. Of note, the majority of Tie2-IFN mice were either tumor-free or had tumors barely detectable by MRI at 4 or 5 weeks PTI, whereas all control mice carried large tumors and had to be euthanized by this time. Despite their small size, tumors detectable in Tie2-IFN mice appeared more necrotic than those of control mice as measured by MRI at 3 weeks PTI. The Tie2-IFN tumors that grew sufficiently to be analyzed displayed decreased cell proliferation and greater apoptosis (assessed by Ki-67 and cleaved caspase-3 immunostaining, respectively) as compared to the control tumors.

In agreement with previous findings[67,71], in Tie-GFP mice, GFP positive TEMs surrounded angiogenic blood vessels. Mononuclear cells positive for IFN-α were detected in the gliomas of Tie2-IFN mice, but not of control mice. The frequency and distribution of IFN positive cells in Tie2-IFN tumors were similar to those of GFP^+ TEMs in control tumors, suggesting that the IFN positive cells represent bona fide IFN-producing TEMs. Notably, Tie2-IFN tumors, but not control tumors, were heavily surrounded and infiltrated by cells expressing Iba1, a monocyte/macrophage/microglia protein upregulated upon cell activation and involved in cell migration and phagocytosis[158]. As previously shown, the wide majority of glioma-infiltrating hematopoietic cells are of BM origin and express F4/80, thus representing tumor-associated macrophages[71]. Together, these results suggest that the Iba1-expressing cells in Tie2-IFN tumors represent macrophages activated by IFN signaling.

The expression of a panel of IFN-inducible genes was then analyzed in the tumors. RNase protection assays (RPA) performed using a set of mouse-specific probes showed that IFN-responsive genes were strongly upregulated in the gliomas of Tie2-IFN mice as compared to control tumors or brain tissue obtained from the contralateral, non-injected brain hemisphere. In particular, 2’5’-oligoadenylate synthetase (Oas1a), tumor necrosis factor-α (Tnfa), and interleukin-1a and -1b (Il1a/b), all typical indicators of type I IFN-induced responses, were significantly upregulated in Tie2-IFN tumors. These findings indicate expression of biologically active concentrations of IFN-α at the tumor site in Tie2-IFN mice. As IFN proteins are species specific, no significant expression of IFN-inducible genes was detected in Tie2-IFN gliomas when RPA was performed using human-specific probes, indicating that the IFN pathway was minimally activated in the tumor cells of human origin. These results indicate that an IFN response targeted to the host-derived components of the tumor is capable of prospectively inhibiting human glioma growth in the mouse brain.

DocuSign Envelope ID: A0702825-E0BD-46DB-A7D6-FEF901E11877

There were striking differences in the vascularization of control and Tie2-IFN gliomas. Whereas the blood vessels of control tumors were notably enlarged and tortuous, those of Tie2-IFN tumors were small, had a regular profile, and were richly covered by $NG2^+$ pericytes, all typical features of quiescent or ''normalized'' blood vessels. The relative vascular area of Tie2-IFN tumors was only 33% of that of control tumors, and the blood vessels of Tie2-IFN tumors contained fewer proliferating endothelial cells and increased numbers of apoptotic cells compared to those of control tumors. Together, these findings strongly suggest that blood vessels of Tie2-IFN gliomas are in a quiescent, non-angiogenic state.

Finally, to compare the safety and the efficacy of our IFN-α delivery strategy to a model of systemic IFN-α administration, mice were intravenously injected with PGK-IFNα LVs thus establishing a stable source of IFN-α from transduced cells in the liver and spleen. Mean IFN-α plasma concentration was 418 ± 124 pg/ml in these mice, which failed to inhibit glioma growth and caused myelotoxicity with marked thrombocytopenia.

Upon orthotopic transplantation into C57Bl/6 mice, mGB2 cells gave rise to high-grade gliomas that faithfully recapitulated the histopathological features, invasiveness and immune cell infiltration characteristic of certain types of human glioblastoma[15959]. In this mGB2 mouse model, IFN-a delivery by TEMs delayed or suppressed GBM growth, whereas in the GL261 preclinical model, low circulating amounts of IFN-a were insufficient to elicit antitumor responses. The antitumor activity was exerted through the reprogramming of the myeloid compartment associated with a shift in the M2 to M1 macrophage balance in favor of pro-inflammatory macrophages (M1) with IFN-a mediated angiogenesis inhibition. Furthermore, there was activation of the adaptive and innate immune response as showed by the reduced percentage of exhausted CD8+ T cells, the immunoglobulin switch from IgM to IgG, the activation of tumor-infiltrating DCs, a positive enrichment of M1 genes and negative enrichment for M2 genes[16060].

1.3.6 Clinical experience with lentiviral-mediated *ex-vivo* gene transfer into hematopoietic stem and progenitor cells

Up to now, *ex-vivo* hematopoietic stem cell gene therapy has mainly been aimed at correcting genetic defects in patients affected by monogenic diseases. In recent years, successful clinical trials, based on lentiviral vector gene transfer, applied this strategy to the prevention and treatment of some severe inherited disorders[161–163]. Genetic modification of self-maintaining

DocuSign Envelope ID: A0702825-E0BD-46DB-A7D6-FEF901E11877

multipotent HSPCs ensures a steady supply of their gene-corrected progeny to the body and therefore represents a unique treatment option or a potentially safer alternative treatment to conventional allogeneic transplantation. In terms of long-term safety and therapeutic benefits, the overall results from past and ongoing clinical trials of lentiviral HSPC gene therapy have been compelling. With respect to prior experience at SR-TIGET, all treated patients affected by Wiskott-Aldrich syndrome (WAS) have shown substantial and clinically meaningful improvements[161]. Similarly, all the patients affected by metachromatic leukodystrophy (MLD) and treated by HSPC gene therapy before or very early after disease onset have shown marked benefits, such as disease prevention or halted progression[162,163]. Several non-clinical and clinical studies, conducted on patients enrolled in HSPC gene therapy trials have shown that lentiviral vectors constitute a valuable and safer alternative to gamma-retroviral vectors (gRV), enabling more efficient gene transfer into HSPCs, a robust and long-term transgene expression in their progeny[164–170] and an alleviated risk of genotoxicity, both because of the vector design, which comprises a self-inactivating long terminal repeat (LTR) and a moderately strong internal promoter driving transgene expression, as well as their integration site bias, which prefers gene bodies and not promoters, as gamma-retroviral vectors instead do[161,171–173]. Overall, these findings are reassuring and support the safety of lentiviral gene transfer into HSPCs, with no evidence of adverse events related to vector insertion in almost a hundred treated patients followed up for up to 9 years in the earliest treated patients. Given the positive safety and efficacy results obtained to date with lentiviral HSPC gene therapy, the translation of this approach to other diseases characterized by high unmet medical need, like GBM, represents a promising option to open new treatment opportunities for affected patients.

1.4 Clinical rationale for Temferon

As discussed previously, the prognosis for most patients with GBM and particularly those with an unmethylated MGMT promoter remains poor despite progress in the management of patients. Use of adjuvant TMZ has not been shown to be of benefit to this subgroup of patients and in some cases may be harmful. Although the introduction of anti-angiogenic therapies with bevacizumab being the lead drug in class initially appeared to be a paradigm shift, subsequent clinical trial experience has been disappointing although it may ameliorate radiation induced cerebral edema and mass effect[174–176]. Investigators are exploring a range of translational medicine approached for recurrent and newly diagnosed GBM including molecular targeted therapeutics, immunotherapies and somatic gene therapies[177]. A significant unmet medical need continues to exist in patients with GBM.

DocuSign Envelope ID: A0702825-E0BD-46DB-A7D6-FEF901E11877

The scientific rationale for evaluating the role of a targeted gene therapy IFN-α approach allowing modulation of the GBM tumor microenvironment is supported by an extensive body of evidence and this has been summarized in **Section 1.3.3** and **1.3.4.** Administering Temferon following surgical resection of the tumor and completion of radiotherapy may be the optimal timepoint for direct inhibition of residual tumor cell growth, inhibition of neo-angiogenesis, enhanced recruitment and activation of innate immune cells, and recruitment and activation of T-cells, in order to maximize the inhibition of disease progression. This approach is justified in patients with unmethylated MGMT promoter where adjuvant use of TMZ has not been shown to be beneficial. The clinical experience with locally injected and systemically administered IFN-α also supports a positive benefit-risk evaluation for some patients with GBM but clearly there are issues relating to systemic toxicities from parenterally administered interferon and tumor necrosis when injected directly into the tumor. The Temferon approach is likely to avoid the systemic toxicities associated with IFN-α reported in previous studies.

1.5 Dosage justification for Temferon

In preclinical mouse models, significant tumor growth inhibition has been observed in experiments with an average VCN as low as 0.08 copies (corresponding to a percentage of transduced cells of about 8%) of Tie2.IFNa vector per human diploid genomes in the bone marrow[178]. The term 'chimerism' is hereafter used to describe the proportion of cells that carry the transgene compared to those that do not. We will use VCN as a surrogate marker for Temferon chimerism in this clinical trial. The VCN quantifies the average ratio of vector genomes per diploid human genomes and cannot resolve multiple integrations in a single cell. It may therefore overestimate the transduced cell chimerism, considering that a fraction of transduced cells may have two Tie2.IFNa.126T vector integrations. For example, if a third of Temferon cells were transduced with 1 copy of Tie2.IFNa.126T, another third with 2 copies and the remaining third with 0 copies, vector copy number would result as 100%, while the fraction of transduced cells would be 66%. Assuming a similar copy number distribution in the Drug Product (DP), a vector copy number of 0.08 would correspond to a transduced cell chimerism of 5%. Given that the degree of overestimation is limited by the transduction protocol (tuned towards avoiding multiple integrations per cell), the release criteria (upper VCN limit of 2 in the DP) and evidence from the mouse model that HSPCs with more than 2 integrations of the Tie2.IFNa vector do not engraft, we will from now apply the VCN definition for *in vivo* Temferon chimerism.

DocuSign Envelope ID: A0702825-E0BD-46DB-A7D6-FEF901E11877

Extrapolating our preclinical data to humans, we expect a lower threshold of efficacy when an average Temferon chimerism of 5%-10% is reached in the myeloid cell compartment *in vivo*. The no observed adverse effect level supported by the preclinical studies is for a chimerism of 50%, suggesting a potential therapeutic window of 5%-50%. *In vivo* chimerism depends on multiple variables including Temferon dose, dose of supporting non-manipulated HSPCs and conditioning regimen. Therefore the objectives for this phase I/IIa dose escalation study are to determine the safety and tolerability of 5 escalating doses of Temferon in the context of conditioning regimens used in bone marrow transplantation and a fixed dose of non-manipulated HSPCs, and to assess the biological activity of Temferon.

The need for Temferon transduced cells to engraft in bone marrow requires a conditioning regimen. Ideally the requirements for such a regimen include achieving rapid and stable engraftment of transduced HSPCs with long term differentiation of myeloid progeny and mobilisation to the tumor tissue of TEMs carrying the IFN-α2 transgene, a direct cytotoxic effect on glioma cells, and minimizing the risk of complete myeloablation which is associated with potentially significant risks relating to infection, anemia, bleeding and end organ damage. A number of options have been considered including high dose carmustine (BCNU) as utilized in a previous gene therapy study in GBM[179], a combination of carmustine and thiotepa[180] or busulfan and thiotepa[181–183] as utilized in primary central nervous system lymphoma, busulfan as used in many completed and ongoing gene therapy studies[163,184–190], and other low intensity conditioning regimens [191,192]. A non-myeloablative conditioning regimen is proposed for initial use in this study using a single dose of carmustine (400mg/m^2) administered intravenously (IV) 6 days prior to Temferon and thiotepa 5mg/kg given twice IV, 5 days prior to Temferon. This conditioning approach is likely to minimize the potential risk for clinically significant myelosuppression[193].

An alternative conditioning approach using busulfan, instead of carmustine, in combination with thiotepa was also evaluated in Cohort 5. The aim was to evaluate whether conditioning with busulfan (Cohorts 5) (instead of carmustine - Cohorts 1-4) in combination with thiotepa may result in a more stable engraftment of Temferon cells that ultimately leads to a greater number and potentially longer term differentiation of transduced myeloid progenitors into IFN-α2 expressing TEMs that migrate into the residual tumor. Busulfan has direct effects on myeloid cell turnover in the brain and may potentiate engraftment of HSPC derived cells in the brain[194,195]. Importantly, historical data confirm that, even at doses higher than those proposed for this study, busulfan when administered as a monotherapy for conditioning, has

DocuSign Envelope ID: A0702825-E0BD-46DB-A7D6-FEF901E11877

greater specificity for myeloid precursors with relative sparing of lymphocytes[196]. Recent findings from ongoing gene therapy studies support this observation (Dr. Bernhard Gentner – Personal Communication) and this is an important consideration for achieving efficacy with Temferon taking into account the known sequestration of T cells in the bone marrow in GBM[197]. Using therapeutic drug monitoring (TDM), complete myeloablation may be avoided by targeting busulfan exposure to achieve an area under the plasma concentration curve (AUC) of 40-60 mg·h/L. Busulfan will be administered IV 4 days prior to Temferon at a dose of 3.2 mg/kg ideal body weight, with a further TDM adjusted dose administered 24 hours (and if necessary 48 hours) later.

The combination of busulfan and thiotepa for conditioning is important particularly when considering early homing of Temferon derived TEMs to the brain tumor in tandem with the pro-inflammatory tumor environment that is likely to exist after radiotherapy[198]. An engraftment peak of transduced progenitors at Day +30 could be beneficial and early engraftment has typically been associated with inclusion of thiotepa as part of conditioning regimens[199–201]. Another argument in favour of inclusion of thiotepa as part of the conditioning regimen is that pre-formed tumor-specific $CD8^+$ T cells may undergo homeostatic expansion following mild lymphopenia [202,203]. Although a reduced lymphocyte repertoire and low $CD4^+$ count post-thiotepa may impair in the short-term the *de novo* generation of tumor-specific T cells, at this point the relative clinical importance of these two thiotepa associated effects on T cells remains unclear in the context of Temferon treatment for GBM. Clinical safety with the combination of thiotepa and busulfan is well established not only in the field of haploidentical HSCT[199–201] but also historically for cerebral lymphoma[181–183], where higher doses have been administered than those proposed for inclusion in this study.

Considering that the current transduction protocol yielded an average VCN of 0.7 ± 0.3 in the test/verification production runs, this may translate into a theoretical *in vivo* chimerism of 14-35% (±1SD 8%-50%) if only Temferon is administered in the absence of supportive, non-manipulated HSPCs after a non-myeloablative conditioning regimen. As this is a first-in-man study for GBM, patients in Cohorts 1-3 received stem cell support using $3x10^6$ non-manipulated $CD34^+$ cells/kg body weight transplanted before Temferon, sufficient to allow full hematologic recovery following the conditioning regimen with either carmustine and thiotepa. Our preclinical biodistribution studies show that Temferon cells successfully engrafted and differentiated into mature progeny when transplanted alone or co-transplanted with non-transduced HSPC but when Temferon cells were transplanted as a standalone graft, they

DocuSign Envelope ID: A0702825-E0BD-46DB-A7D6-FEF901E11877

showed a lower total and myeloid engraftment compared to the control group or to the co-transplanted groups. Sequential infusion resulted in an *in vivo* chimerism that may be predicted based on the ratio of the transplanted $CD34^+$ cells doses. In the first dose cohort, Temferon constituted 14% of the total dose of infused $CD34^+$ cells (0.5 x 10^6 Temferon cells/kg and 3.0 x 10^6 non-manipulated $CD34^+$ cells/kg), with a dose correction scheme in case the measured VCN in the DP deviates more than 1 standard deviation from the average measured in the test runs. Dose Cohort 2 received 1.0 x 10^6 Temferon cells/kg (25% of the total dose of infused $CD34^+$ cells), and Dose Cohort 3 received 2.0 x 10^6 Temferon cells/kg (40% of the total dose of infused $CD34^+$ cells).

Cohorts 4 and 5, in the absence of 2 or more dose-limiting toxicities in Cohort 3, employed 2.0 x 10^6 Temferon cells/kg with a reduced number of non-manipulated $CD34^+$ cells (2.0 x 10^6/kg) (i.e. 50% of the total dose of infused $CD34^+$ cells). Cohort 4 received as conditioning regimen carmustine and thiotepa, whereas Cohort 5 received busulfan and thiotepa.

Dose-limiting toxicities have not been observed in the 9 patients that were treated at Temferon Dose Level 3, while engraftment of genetically engineered cells stabilised around the 10% mark, with little difference between the 2 conditioning regimens. Based on these observations and the possibility that higher engraftment may translate into a higher likelihood of therapeutic efficacy, the study enrolled an additional Temferon Dose Level, conditioned with the BCNU & Thiotepa regimen used for Cohorts 1-4. Patients in Cohort 6 received 3.0 x 10^6 Temferon cells/kg (Dose Level 4), with the same number of non-manipulated $CD34^+$ cells (2.0 x 10^6/kg) (i.e. 60% of the total dose of infused $CD34^+$ cells). No dose limiting toxicity was observed in this Cohort. However, review of safety data from all 6 cohorts showed that serious adverse events (CTCAE $\geq$ Grade 3) attributed to the conditioning regimen have occurred associated with hematologic complications (e.g. febrile neutropenia, secondary graft failure), venous thromboembolism, pneumonia and other infections. The majority of patients have also received dexamethasone during the post-transplant period as a treatment for radiotherapy associated cerebral edema. The combination of glioblastoma, brain irradiation, conditioning chemotherapy and dexamethasone has therefore contributed to complications resulting from immunosuppression.

In order to mitigate these recognised risks, the conditioning chemotherapy regimen will be modified in two additional cohorts (Cohorts 7 and 8) to utilise busulfan monotherapy (total busulfan AUC target 60 mg·h/L). Thiotepa will be omitted. In addition, patients utilizing

dexamethasone >4mg qd within 20 days of Temferon administration will undergo frequent medical review over 3 weeks to reduce dexamethasone requirements to ≤4mg qd.

Cohorts 7 and 8 will recruit 3 patients each who will be administered busulfan and an infusion of 3 x 10^6 non-modified $CD34^+$ cells/kg. Patients in Cohort 7 will receive 3 x 10^6 transduced cells/kg (Dose Level 4), while patients in Cohort 8 will receive 4 x 10^6 transduced cells/kg (Dose Level 5) (i.e. 50% and 57%, respectively, of the total dose of infused $CD34^+$ cells).

1.6 Benefit-risk assessment for Temferon

1.6.1 Potential benefits

Our renewed interest in IFN as an anticancer treatment is based on the potential advantages associated with a cell-based, specific delivery of IFN-α into the tumor microenvironment by TEMs. We anticipate that serious systemic symptoms will be avoided, overcoming the tolerability issues associated with systemic IFN treatment. Furthermore, IFN deployed locally in the tumor microenvironment may allow full exploitation of its pleiotrophic anti-tumor activities described in previous sections while limiting systemic adaptation to chronic IFN stimulation and immunoparalysis potentially associated with this phenomenon. The timing of Temferon administration after completion of radiotherapy, which in itself has immunomodulatory properties, is also important and Temferon may have a synergistic effect to increase PFS and OS following optimal standard of care treatment in patients with GBM who have an unmethylated MGMT promoter.

This study will be evaluating a new cell and gene-based strategy that specifically delivers IFN-α to the tumor microenvironment, allowing persistent, therapeutically relevant IFN-α activity at the tumor sites without the need of chronic exogenous administration of the product introducing a new line of maintenance treatment.

1.6.2 Potential risks

Whereas our gene therapy strategy is based on a novel and yet unexplored approach for cell- and gene-based delivery of a biological therapeutic, the drug itself is not novel, and represents a normal human cytokine mediating inflammatory/immune response, whose physiological activity and pharmacological usage are well known. This knowledge helps to address the concerns associated with the first-in-human testing of our strategy and the intrinsic limitations

DocuSign Envelope ID: A0702825-E0BD-46DB-A7D6-FEF901E11877

of pre-clinical toxicity testing in mouse models, which may fail to comprehensively interrogate the potential adverse effects of a pleiotropic cytokine such as IFN-α.

In recent years, high constitutive expression of IFN-stimulated genes (ISGs) has been associated to a family of rare monogenic, Mendelian inherited, autoinflammatory diseases named type I interferonopathies[204]. The phenotypic spectrum of the heterogeneous group of genetically determined type I interferonopathies is broad, ranging from severe neurological impairment to mild cutaneous disease. Systemic inflammation and autoimmunity are commonly shared features of these pathologies. Signs common to many of these diseases are skin vasculopathy with chilblains and livedo reticularis, interstitial lung disease, panniculitis, recurrent fever, arthropathies, autoimmune manifestations, malaise, growth failure, glomerulonephritis and neurologic signs (encephalopathy, seizures, intracranial calcifications).

The emerging knowledge from these diseases in combination with the well-established safety profile of approved IFN-α therapies has two important implications for the first-in-human testing of our gene therapy strategy in GBM patients. The signs and symptoms reported in patients affected by type I interferonopathies and the adverse effects recorded following IFN-α systemic administration highlights some of the potential target organs/toxicities to be carefully monitored in our study and the maximal level of severity to be expected even in the worst-case scenario of uncontrolled systemic IFN expression.

1.6.2.1 Vector mobilization and recombination

Upon transduction, the vector becomes stably integrated as a provirus within the chromatin of target cells. Because of the replication-defective nature of the vector, there is no possibility of spontaneous mobilization of the provirus from the transduced cells unless helper functions are also provided in the transduced cells either by recombination during vector production or by super-infection with wild-type human immunodeficiency virus (HIV) in an infected host. Both of these possibilities are extremely unlikely, because: 1) the multi-split design of the packaging system and the lack of homology between the envelope and transfer vector genome make it extremely unlikely that a replication-competent recombinant is generated during production; 2) the self-inactivating feature of the vector LTR prevents transcription from the proviral LTR and thus prevents vector mobilization even in the case of recombination with the packaging constructs or superinfection of the transduced cell by wild-type HIV-1. Moreover, contamination by replication competent lentivirus (RCL) in lentiviral vector lots is excluded.

No case of mobilization or recombination has been observed in OSR-TIGET trials, and neither has such a phenomenon been reported in any other gene therapy trials using the lentiviral vector platform.

1.6.2.2 Transduction of bystander cells

The risk that some infectious vector particles remain associated with the transduced cells and can be subsequently released to transduce bystander cells is also very limited using purified LV vectors. First, the amount of vector being released is conceivably limited, and thus the chance of off-target transduction is low. Second, we have conducted experiments specifically aimed at assessing the extent of lentiviral vector release from previously transduced human HSPCs. Our results with the LV platform showed that: 1) vector release and bystander transduction can occur, but at substantially lower level when using vector stocks from the large-scale production and purification process used for Good Manufacturing Practice (GMP) production, as compared to laboratory grade stocks[205]; 2) exposure of the transduced HSPCs to autologous human plasma significantly inhibits bystander transduction, becoming virtually undetectable when using large scale LV stocks. These findings suggest that in the clinical setting, where the transduced cells circulate in the bloodstream of patients, the potential occurrence of bystander cell transduction is negligible[205].

A Good Laboratory Practice (GLP) biodistribution study for Temferon (TM042) has not shown evidence for *in vivo* transduction of mouse tissues, in line with other biodistribution studies performed at SR-TIGET[206]. Vector DNA was associated in a stable ratio with the genome of human cells in all mouse organs and tissues.

1.6.2.3 Germline transmission

Because the risk of inadvertent germ line transmission associated with the administration of *ex vivo* transduced human cells is considered “low” or “minimal” according to the European Medicine Agency (EMA) and the International Council for Harmonisation (ICH), respectively, and animal testing of human cells may be difficult or not meaningful, non-clinical germline transmission studies of human genetically modified cells are not recommended (EMA Guideline on non-clinical testing for inadvertent germline transmission of gene transfer vectors, EMA/273974/2005 and CHMP/ICH/469991/2006)[207,208].

DocuSign Envelope ID: A0702825-E0BD-46DB-A7D6-FEF901E11877

Moreover, no evidence of germline transmission nor of transgene expression in germ cells after *in vivo* delivery of gRV and LV into experimental animals have been reported to date. In a study of testicular tubule injection of LV in mice, the sperm collected from recipient testes were able to generate normal pups and none of the offspring carried the transgene[209]. Rat spermatogonial stem cells, however, can be effectively transduced *ex vivo* by LV and generate viable transgenic offspring upon re-implantation into rat testes. Moreover, LV can transduce *in vitro* fertilized eggs from a variety of species, including mouse, rat and pig. Indeed, LV are becoming common tools for the generation of transgenic animals by *in vitro* oocyte transduction followed by in utero implant[210].

In the biodistribution study (Study TM042), Temferon transplanted alone (test item at a high dose, TI high) or combined with a 2.3 fold excess of non-manipulated $CD34^+$ cells (30% Temferon, test item at a low dose, TI low) in NSG mice, the presence of LV genome in the gonads was assessed at the time of sacrifice, 12 weeks post-transplant. For this experiment, DNA was extracted from the gonads of perfused mice and quantitative polymerase chain reaction (qPCR) was performed for vector and human genome content. In 64% of TI high and 93% of TI low mice, the ovaries were negative or below the limit of quantification for VCN analysis. In the remaining mice, measured VCN values were in the range of VCN measured in other samples from the same mouse, consistent with incomplete perfusion and contamination of the sample with residual human blood cells containing the TIA126 vector.

1.6.2.4 Genotoxicity and carcinogenicity

Insertional oncogenesis is a possible consequence of semirandom vector integration into the human genome. Several decades of studies of gamma- or oncoretrovirus-induced lymphomas/leukemias[211–213] in mice have shown that proviral insertions near or within proto-oncogenes can trigger their overexpression and/or aberrant transcription and lead to cell transformation. Both mechanisms of oncogene activation are dependent on the strong transcriptional enhancer/promoter elements contained in the viral LTR. In clinical trials using gRV, activation of *MECOM, LMO2,* and other oncogenes resulting in myelodysplasia and/or leukemogenesis has been reported in 6 out of 20 X-linked SCID patients treated with HSPC gene therapy[214,215], in 7 out of 10 patients treated with gRV WAS gene therapy[216] and 4 chronic granulomatous disease patients treated with gRV HSPC gene therapy[217,218]. A better understanding of the risks associated with gRV and LV in HSPC gene therapy has emerged from a growing number of pre-clinical studies which have modeled genotoxicity in *in vitro* cell

DocuSign Envelope ID: A0702825-E0BD-46DB-A7D6-FEF901E11877

immortalization assays and in *in vivo* murine models of leukemia/lymphoma development, and from transcriptional studies of gene de-regulation at the vector integration sites in cells transduced *in vitro* or retrieved from transplanted mice and patients[219,220]. Direct comparison of gRV and LV in these assays demonstrated that prototypical LV has a substantially lower oncogenic potential than gRV[221–223]. Important features contributing to the lower genotoxicity of LV as compared to conventional gRV are thought to be: 1) the self-inactivating design, which prevents LTR-dependent effects on the transcription of genes neighboring the integration site; 2) the use of a vector internal promoter to drive transcription of the therapeutic transgene; 3) different integration preferences of LV vs. gRVs. LV favor gene bodies and introns rather than promoters and CpG islands of actively transcribed genes thereby making a transactivation event less likely.

The safety data from gene therapy clinical trials conducted at OSR-TIGET in MLD, WAS and β-thalassemia[191,224–226] as well as the extensive preclinical testing of the vector proposed for clinical use[223,227,228] have provided evidence for the safe profile of LV integration at high vector doses, as requested for clinical efficacy in these pathologies. Vector integration analyses showed stable polyclonal hematopoietic repopulation by the transduced HSPCs and no evidence for skewing in genomic distribution or specific gene classes by comparing vector integration sites in pre- and post-transplant samples[161–163,226]. These data provide compelling evidence that LV allow efficient, safe and long-term marking of human hematopoiesis.

The vectors tested in the above mentioned clinical trials were third-generation LV similar to the one proposed for this study, except for the transgene, the internal promoter and the presence of the miR-126 targeted sequences in the 3’UTR. The internal Tie2 enhancer/promoter has weak and lineage-specific transcriptional activity, reducing even more the concerns on its transactivation potential as compared to ubiquitous promoters currently employed in LV-based gene therapy trials. The target sequences recognized by microRNA-126 in HSPCs are unlikely to interfere with the regulation of endogenous microRNA-126 targets, due to perfectly complementary nature of the target sequences and the low dose of transcripts containing the miRNA targets that accumulate in HSPCs as a consequence of Tie2-driven expression[10,91,92,229–231]. Given that miR-126 favors the development and persistence of leukemia[232,233], its inhibition by an excessive amount of TIA126 transcripts would reduce the risk of oncogenesis while having benign effects on HSPC such as a modest expansion of their numbers[231], in the highly unexpected case that the expression of the miRNA targets interfered with the regulation of endogenous miR-126 targets. The transgene product, IFN-α2, has not

DocuSign Envelope ID: A0702825-E0BD-46DB-A7D6-FEF901E11877

been associated with an increased carcinogenic risk in patients, as witnessed by a huge body of literature describing decades of experience with its clinical use in a multitude of disease contexts, including infectious, autoimmune and oncologic indications. In synthesis, the TIA126 LV construct carries a comparably low genotoxic risk even when benchmarking it against state-of-the-art vectors currently used in other *ex vivo* gene therapy protocols.

A theoretical risk inherent to this protocol is the use of HSPCs from comparably old individuals (up to 70 years of age). These cells may show age-associated alterations such as reduced self-renewal and reconstitution potential, myeloid differentiation bias, increasing mutational burden and reduced clonality up to clonal hematopoiesis[234]. Although an increased risk of secondary hematologic malignancies consequent to the engineering of aged HSPC with TIA126 cannot be excluded, a more likely scenario is reduced engraftment and premature exhaustion of Temferon, with consequent loss of potential therapeutic efficacy.

1.6.2.5 Risks related to autologous HSPC collection, conditioning chemotherapy and autologous HSPC infusion

Autologous HSPC collection/harvesting, administration of conditioning chemotherapy to provide sufficient space in the BM for Temferon cells to engraft, and administration of autologous HSPCs to ensure that full hematological recovery occurs quickly, is not standard of care for patients with GBM. Nevertheless, the potential benefit to patients of receiving Temferon outweighs the risks associated with the procedures required to prepare and administer Temferon to patients. HSPC harvest will likely be performed using leukapheresis via a large peripheral venous cannula. However, if deemed necessary by the Investigator, a central venous catheter may be required. Standard procedural risks associated with implantation of a central venous catheter for collection of HSPCs by means of leukapheresis are to be taken into consideration[235,236]. In particular, the risks of central catheter implantation include bleeding, formation of hematoma, pain, infections, thrombosis with risk of pulmonary embolism and requirement for anticoagulant treatment, and rarely pneumothorax. Adverse reactions to leukapheresis can derive from transient volume loss and lowering of calcium levels by the citrate anticoagulant.

The use of the stem cell mobilization agents lenograstim (granulocyte colony stimulating factor, G-CSF) and plerixafor may be associated with specific, usually mild, side effects. These include, in the case of lenograstim, fever, musculoskeletal pain and rarely allergic-type

DocuSign Envelope ID: A0702825-E0BD-46DB-A7D6-FEF901E11877

reactions and capillary leak syndrome. Very rarely, lenograstim has been associated to pulmonary events such as interstitial pneumonitis. Overall, the safety record of lenograstim is good; nevertheless, there is a rare risk of cardiovascular and thromboembolic events, as well as splenic rupture as a consequence of extreme leukocytosis under specific circumstances. Plerixafor is registered for use in adults. Common adverse reactions reported in patients in combination with lenograstim include insomnia, dizziness, headache, diarrhea, nausea, vomiting, abdominal pain, stomach discomfort, dyspepsia, abdominal distention, constipation, flatulence, oral hypoaethesia, dry mouth, hyperhidrosis, erythema, arthralgia, musculoskeletal pain, injection and infusion site reactions, fatigue and malaise. Further information for both lenograstim and plerixafor can be found in the respective Summary of Product Characteristics included in the Study Operations Manual (SOM).

Risks associated with the infusion of cryopreserved autologous HSPCs (relevant for both the non-manipulated stem cell support and Temferon) are associated with the presence of dimethylsulfoxide (DMSO) as a cryoprotectant. DMSO may lead to the following findings and side effects upon infusion: appearance of a garlic-like odor/taste which may last for several days, histamine release leading to nausea, vomiting, diarrhea, headache, flushing, fever, chills, dyspnea, anaphylaxis, vasodilatation and hypotension, pulmonary or abdominal complaints; neurotoxicity manifested by complex reactions of cognition and emotion; cardiotoxicity manifested by for example bradycardia, liver and kidney injury. It should be noted, though, that the side effects reported to be associated with DMSO often have a multifactorial cause, and DMSO exposure will be spread over a time window of up to 12 hours reducing to a minimum the likelihood of severe reactions. The highest DMSO exposure will come from infusion of non-manipulated supporter cells not selected for the $CD34^+$ fraction. Three million $CD34^+$cells/kg usually contain 15-30 ml of DMSO, while the DMSO contained in Temferon is generally less than 2 ml. The total DMSO infused is therefore at the lower end of what patients typically receive during an ASCT [237].

Regarding infusion reactions, the DP consists of autologous cells that have been extensively washed after the transduction procedure, making short-term toxicity directly linked to the genetically engineered cells unlikely. Potential reactions associated with the infusion of the DP may include fever, tachycardia, nausea and vomiting, joint pain, skin rash.

Carmustine (BCNU) and thiotepa were utilized as a conditioning regimen in Cohorts 1-4 and Cohorts 6 prior to infusion of non-manipulated supporter cells and Temferon. Very common

DocuSign Envelope ID: A0702825-E0BD-46DB-A7D6-FEF901E11877

adverse reactions that may occur with a single 400 mg/m^2 dose of BCNU include myelosuppression, ataxia, dizziness, headache, transient conjunctival flushing and blurred vision, retinal hemorrhages, hypotension (due to alcohol content of diluent), phlebitis, nausea and vomiting, and transient hyperpigmentation of the skin associated with accidental skin contact. Infusion reactions have also been reported.

For thiotepa, the most commonly reported adverse reactions are nausea and dizziness. Bone marrow depression, including thrombocytopenia, and increased susceptibility to infections have been reported. Other adverse reactions relevant to the proposed use as part of a conditioning regimen includes allergic reactions, headache, dizziness, nausea, vomiting, diarrhea, abdominal pain, oral mucositis, rash, contact dermatitis, alopecia, hemorrhagic cystitis, fatigue, febrile reaction, pain at the injection site, skin discoloration and irritation following topical exposure.

Busulfan and thiotepa was utilized as a conditioning regimen in Cohort 5 prior to infusion of non-manipulated supporter cells and Temferon. Very common adverse reactions that may occur with busulfan include myelosuppression and infections, which reflect the immune status of patients during conditioning. Gastrointestinal symptoms such as nausea and vomiting, stomatitis, and diarrhea have also been reported very commonly. Hepatic veno-occlusive disease, acute respiratory distress syndrome and seizures have also been reported as serious adverse reactions. Impaired fertility commonly occurs after use of busulfan.

Following review of safety data from Cohorts 1-6, serious adverse events (CTCAE ≥ Grade 3) attributed to the conditioning regimen have occurred associated with hematologic complications (febrile neutropenia, secondary graft failure), venous thromboembolism, pneumonia and other infections. The majority of patients have also received dexamethasone during the post-transplant period as a treatment for radiotherapy associated cerebral edema. The combination of glioblastoma, brain irradiation, conditioning chemotherapy and dexamethasone has therefore contributed to complications resulting from immunosuppression.

In order to mitigate these recognised risks, and following discussion and agreement with the Data Monitoring Committee (DMC), the conditioning chemotherapy regimen will be modified in two additional cohorts (Cohorts 7 and 8) to utilise busulfan monotherapy (total busulfan AUC target between 40 to 60 mg·h/L). Thiotepa will be omitted. In addition, patients utilizing

DocuSign Envelope ID: A0702825-E0BD-46DB-A7D6-FEF901E11877

dexamethasone > 4mg qd within 20 days of Temferon administration will undergo frequent medical review over 3 weeks to reduce dexamethasone requirements to ≤ 4mg qd.

Further information for BCNU, thiotepa and busulfan can be found in the respective Summary of Product Characteristics included in the SOM.

1.6.2.6 Interferon-alpha related side effects

The cell based therapeutic approach that is being utilized in this study is designed to allow local, low dose IFN-α2 release at the tumor site, reducing the potential for systemic side effects. As a benchmark for a "worst case scenario", potential adverse drug reactions related to systemic IFN-α administration (well described in the Summary of Product Characteristics of approved medicines such as Intron A[238]), are summarized below focusing on adverse reactions where warnings exist.

1.6.2.6.1 Central Nervous System and psychiatric effects

Severe central nervous system (CNS) effects, particularly depression, suicidal ideation and attempted suicide have been observed in some patients during IFN-α therapy, and even after treatment discontinuation mainly during the 6-month follow-up period. Other CNS effects including aggressive behavior (sometimes directed against others such as homicidal ideation), bipolar disorders, mania, confusion and alterations of mental status have been observed with alpha interferons. Patients in this study should be closely monitored for any signs or symptoms of psychiatric disorders and will be reviewed periodically by a psychiatrist. If such symptoms appear, the potential seriousness of these undesirable effects must be borne in mind by the Investigator and the need for adequate therapeutic management should be considered. If psychiatric symptoms persist or worsen, or suicidal or homicidal ideation is identified, psychiatric intervention should occur as appropriate.

1.6.2.6.2 Hypersensitivity reactions

Acute hypersensitivity reactions (e.g., urticaria, angioedema, bronchoconstriction, anaphylaxis) to IFN-α have been observed rarely. If such a reaction develops in this study, institute appropriate medical therapy.

1.6.2.6.3 Abnormal liver function and prolongation of coagulation markers

IFN-α may increase the risk of hepatic decompensation and death in patients with cirrhosis. Regular monitoring of liver function tests will occur in this study. Any patient developing liver function abnormalities during treatment with IFN-α must be monitored closely. Elevation of alanine aminotransferase (ALT) ≥ 2 x upper limit of normal (ULN) should result in monitoring of ALT, prothrombin time, alkaline phosphatase, albumin and bilirubin every 2 weeks.

1.6.2.6.4 Myelosuppression

Clinically significant laboratory abnormalities, most frequently occurring at high systemic doses of IFN-α, include reduction in granulocyte and white blood cell counts, decreases in hemoglobin level and platelet count. Moderate and usually reversible pancytopenia has been reported. Regular monitoring of complete blood counts will occur in this study.

1.6.2.6.5 Hypotension & need for adequate hydration

Hypotension may occur during IFN-α therapy and may require supportive treatment. Adequate hydration must be maintained since hypotension related to fluid depletion has been seen in some patients. Fluid replacement may be necessary.

1.6.2.6.6 Pyrexia

While pyrexia may be associated with the flu-like syndrome reported commonly during interferon therapy, other causes of persistent pyrexia must be ruled out.

1.6.2.6.7 Pulmonary conditions

Pulmonary infiltrates, pneumonitis, and pneumonia, occasionally resulting in fatality, have been observed rarely in IFN-α treated patients. The aetiology has not been defined. Regular monitoring of lung function via chest X-ray, lung function tests and diffusion capacity will occur in this study. Any patient developing pyrexia, cough, dyspnea or other respiratory symptoms must have a chest X-ray taken. If the chest X-ray shows pulmonary infiltrates or there is evidence of pulmonary function impairment, the patient is to be monitored closely. Treatment with corticosteroids may assist with the resolution of pulmonary adverse events.

1.6.2.6.8 Ocular adverse events

Ocular adverse events including retinal hemorrhages, cotton wool spots, serous retinal detachment, and retinal artery or vein obstruction have been reported in rare instances after treatment with IFN-α. All patients undergo regular eye examinations using slit lamp ophthalmoscopy. Any patient complaining of changes in visual acuity or visual fields, or reporting other ophthalmologic symptoms during treatment, must have a prompt and complete eye examination.

1.6.2.6.9 Obtundation, coma and encephalopathy

More significant obtundation and coma, including cases of encephalopathy, have been observed in some patients, usually elderly, treated at higher doses of IFN-α. While these effects are generally reversible, in few patients full resolution took up to three weeks. Very rarely, seizures have occurred with high doses of IFN-α.

1.6.2.6.10 Patients with pre-existing cardiac abnormalities

Patients with significant cardiac abnormalities based on medical history, clinical examination or investigations including echocardiography, are excluded from the study. Cardiac arrhythmias (primarily supraventricular) have been reported with IFN-α and these usually respond to conventional therapy.

1.6.2.6.11 Hypertriglyceridemia

Hypertriglyceridemia and aggravation of hypertriglyceridemia, sometimes severe, have been observed. Monitoring of lipid levels will therefore occur in this study.

1.6.2.6.12 Patients with psoriasis and sarcoidosis

IFN-α may exacerbate pre-existing psoriatic disease and sarcoidosis. Patients with these conditions are excluded from participation in this study.

1.6.2.6.13 Kidney and liver graft rejection

Preliminary data indicates that IFN-α therapy may be associated with an increased rate of kidney graft rejection. Liver graft rejection has also been reported. Patients with kidney or liver transplants are ineligible for the study and are excluded from this study.

1.6.2.6.14 Auto-antibodies and autoimmune disorders

The development of auto-antibodies and autoimmune disorders has been reported during treatment with IFN-α. Patients predisposed to the development of autoimmune disorders may be at increased risk. Auto-antibodies will be carefully monitored for the duration of the study.

1.6.2.6.15 Thyroid disorders

Infrequently, adult patients treated with IFN-α developed thyroid abnormalities, either hypothyroidism or hyperthyroidism. In clinical trials using IntronA therapy, 2.8% patients overall developed thyroid abnormalities. The abnormalities were controlled by conventional therapy for thyroid dysfunction. The mechanism by which IFN-α may alter thyroid status is unknown. Thyroid function will be monitored throughout the study and any thyroid abnormality detected during the study will be treated with conventional therapy. Discontinuation of IFN-α therapy has not reversed thyroid dysfunction occurring during treatment.

1.6.2.7 Environmental risks

The gene therapy planned for this clinical trial does not involve particular risks in terms of public health. The cell injection will occur in a dedicated room of the authorized area (Impianto Area for MOGM Class -MI/IC/IMP.2/01-002 Rev.2) in the Hematology and Bone Marrow Transplantation Unit (UTMO) at Ospedale San Raffaele, which has been notified and approved for the use of the specific genetically modified micro-organisms.

The procedures and programs for the cleaning, disinfection and decontamination of the ward, the operating theatre (if it is used) and the equipment do not differ from the standard ones in force for the management of patients who undergo bone marrow transplantation, according to Institutional standard operating procedures.

All the solid and liquid waste contaminated by biological and/or potentially infected material are disposed of in compliance with the hospital regulations in force. All members of the staff, wearing gloves and a protection mask, are adequately educated and trained. In particular, staff are instructed on how to apply the regulations in force to patients who receive multiple transfusions, are immunosuppressed, and at high risk of infections.

In a review of the literature, no replication competent retrovirus (RCR) was detected in 103 patients treated with *ex vivo* retrovirally transduced cells, nor in 342 patients treated *in vivo* with retroviral vectors[239]. Overall, in more than 18 years of clinical trials, there has been no report of cultural or molecular RCR test positivity, in more than 300 clinical trials conducted worldwide with retroviral vectors and a very high number of people considering healthcare staff, family members and other people. In addition, no RCL has been detected in the clinical trials currently conducted with self-inactivating lentiviral vector[167]. Even in the unlikely event of RCL, the risk of transmission is expected to be typical of a lipid membrane enveloped virus. Therefore, risks for healthcare staff and others should be mostly limited to accidental venipuncture and accidental mucosal or parenteral exposure to body fluids. An emergency plan is active in Ospedale San Raffaele for this circumstance.

The patients will be followed to exclude the possible development of RCL infection (see above). Samples will be collected at Screening, and at Day +90, +360 and +720 and yearly thereafter, as suggested by FDA and EMA guidelines (Guidance for Industry, November 2006; Guideline on follow-up of patients administered with gene therapy medicinal products, EMEA/CHMP/GTWP/60436/2007)[240,241]. In case the samples collected during the first two years result always negative for the screening tests (ELISA for HIV p24 antigen in serum), the subsequent yearly samples will be stored, but not analysed. Of note, HIV-derived RCL would conceivably be sensitive to, and treatable by, conventional anti-HIV therapy targeting all *pol*-derived enzymes (RT, protease and integrase).

2 Study Objectives and Endpoints

2.1 Primary Objectives

The primary objective of TEM-GBM study is to evaluate the safety and tolerability of 5 escalating doses of Temferon in up to 27 patients with GBM who have an unmethylated MGMT promoter, following first line radiotherapy. Cohorts 1-3, and 6 each included 3 patients and evaluated the safety, tolerability and long-term hematologic engraftment after a conditioning regimen with BCNU and thiotepa, followed by Temferon Dose Level 1-4. Cohorts 4 and 5, each included 3 patients and evaluated the safety, tolerability and long-term hematologic engraftment after 2 different conditioning regimens (BCNU and thiotepa and thiotepa and busulfan), followed by Temferon Dose Level 3. Cohorts 7 and 8 will receive busulfan as a single agent (monotherapy) conditioning, and the same dose of unmodified cells as of Cohorts 1-3, i.e. $3x10^6$ non-manipulated $CD34^+$cells/kg. Patients in Cohort 7 will receive Temferon Dose Level 4, while patients in Cohort 8 will receive Dose Level 5.

2.2 Secondary Objectives

The secondary objective of this study is to evaluate biological activity and efficacy of Temferon in patients with GBM and unmethylated MGMT when used following standard of care first line therapeutic approaches.

2.3 Primary Endpoint

The primary endpoint is to assess the tolerability and safety of Temferon over the first 90 days following Temferon administration, as evaluated by:

- Routine clinical and laboratory surveillance;
- Assessment of autoimmune manifestations;
- Significant organ toxicities (>grade 2 according to the National Cancer Institute Common Terminology Criteria for Adverse Events [NCI CTCAE v5.0] that are attributed to Temferon exposure or procedures related to the harvesting of HSPCs or administration of Temferon.

2.4 Secondary Endpoints

- Long-term tolerability and safety of Temferon up to 2 years following Temferon administration, according to CTCAE v5.0 criteria as evaluated by:
  - Routine clinical and laboratory surveillance;
  - Assessment of autoimmune manifestations;
  - Significant organ toxicities (> grade 2 according to CTCAE v5.0) that can be attributed to Temferon exposure.
- The proportion of patients achieving hematologic recovery by Day +30 following the administration of non-manipulated autologous HSPC and Temferon. Hematologic recovery is defined as the first of at least 3 consecutive days with a neutrophil count > 0.5 x 10^9/L and platelet count > 20 x 10^9/L.

Safety (Part A only)

- Determine the maximum tolerated dose (MTD) of Temferon as defined by the presence of a CTCAE Grade 3-5 adverse event (AE) that occurs within the first 30 days and is attributed to Temferon.

- Assess the tolerability and safety of three conditioning regimens (BCNU and thiotepa, busulfan and thiotepa, or busulfan monotherapy), up to Day +45 following administration of Temferon, in patients with GBM.

Efficacy (Part A & Part B, if any)

- Identify the presence of transduced myeloid cells in bone marrow aspirate at Day +30 and +90 following administration of Temferon as determined by VCN.
- Identify the presence of transduced myeloid cells in peripheral blood (PB) between Day +30 and +90 following administration of Temferon as determined by VCN.
- Evaluate persistent transduced myeloid cells in PB as assessed by VCN. Persistence is defined as a VCN above the level of detection evident for at least 12 weeks. Persistence according to the type of conditioning regimen used, will also be evaluated.
- Determine the proportions of patients achieving complete response (CR), partial response (PR), stable disease (SD), and progressive disease (PD) at 6 months (Day +180) following Temferon administration, using standard iRANO criteria[242].

- Clinical response will also be evaluated at Day +90, +120 and every 2 months until Day +720. Time to progression/progression-free survival (PFS) will be measured from the first day following Temferon administration until unequivocal progression is identified.
- Overall Survival (OS) at various timepoints up to 2 years, will be calculated from the first day following Temferon administration.
- Changes over time (Day +30, +90, +180, +360, +540, +720) in functional status using the Eastern Cooperative Oncology Group Performance Status (ECOG) and Karnofsky Performance Status (KPS).
- Changes over time (Day +90, +180, +360, +720) in quality of life using the EORTC Quality of Life Questionnaires C30 & BN20.

2.5 Exploratory Endpoints

- Imaging characteristics of the tumor over time according to MRI and additional functional imaging.
- Subgroup analyses of clinical response and imaging parameters according to baseline characteristics.
- Immunohistochemistry characteristics of tumors at first surgery and following repeat surgery including proliferative and apoptotic index (Ki-67, Cleaved Caspase 3, Tunel assay), type of vascularization (CD31 or vWF), EGFR and EGFRvIII expression, evaluation of T cells and myeloid cells infiltrate including the presence of transduced Tie-2 expressing monocytes, and presence/absence of IFN gene expression.
- Tumor infiltrating lymphocytes content analysis in tumor samples at diagnosis and correlation with clinical response.
- Evaluation of the T cell immunorepertoire in tumor sample and PB at diagnosis, in tumors following repeat surgery and in PB over time after Temferon administration (-6, +30, +90, +180, +360, and +720).
- Changes in general immune system composition in peripheral blood over time.
- Characterization of IFN-α gene signatures in tumors following repeat surgery (including Ifn2a, Irf7, Oas1a, Ifit1).

DocuSign Envelope ID: A0702825-E0BD-46DB-A7D6-FEF901E11877

- Characterization of IFN-α gene signatures and mutations in CSF over time (including Ifn2a, Irf7, Oas1a, Ifit1).
- Characterization of IFN-α gene signatures in bone marrow and peripheral blood over time.
- Characterization of genetic (including Mutation/Deletion PTEN, P53, IDH1/2, EGFR amplification) and epigenetic changes (MGMT methylation) in tumors at first surgery and following repeat surgery.
- Future proteomic and metabolomic analyses to identify biomarkers for clinical response.
- Concentration of IFN-α2 in peripheral blood and CSF over time.
- Concentration of cytokines in CSF over time (including IL-10).
- Identification of presence of transduced cells in CSF over time (as determined by VCN).

DocuSign Envelope ID: A0702825-E0BD-46DB-A7D6-FEF901E11877

3 Investigational Plan

3.1 Study Design

This is a non-randomized, open label, multicenter, phase I/IIa, therapeutic-exploratory, dose-escalation, prospective study, involving a single injection of Temferon, an investigational ATMP consisting of autologous CD34$^+$-enriched HSPCs exposed to transduction with a 3rd generation, vesicular stomatitis virus G (VSV-G) pseudo-typed lentiviral vector driving myeloid-specific IFN-α2 expression, which will be administered to up to 27 patients affected by GBM who have an unmethylated MGMT promoter. The study will recruit and follow-up patients at specialist neurosurgical and neuro-oncology units in Italy. Administration of Temferon and hematological follow up will take place at specialist hematology and bone marrow transplantation units.

Potentially eligible patients will be identified immediately after surgical resection of GBM once the MGMT promoter methylator status is known. Once written, informed consent is obtained, and screening procedures have been completed, harvesting of HSPCs will occur. A standard of care regimen of radiotherapy lasting approximately 6 weeks, will then take place. During this time, Temferon manufacturing will occur [redacted]. Following completion of radiotherapy, patients will be admitted to the bone marrow transplant unit for receipt of a conditioning regimen which in Cohorts 1-4 and 6 consisted of BCNU and thiotepa, and in Cohorts 5 consisted of busulfan and thiotepa. The conditioning regimen in Cohorts 7 and 8 will be busulfan monotherapy. The conditioning is followed by administration of non-manipulated HSPCs and Temferon. In-patient monitoring on the bone marrow transplant unit will occur daily until hematologic recovery occurs and these data will be recorded in the eCRF. Thereafter, regular follow-up of patients will occur up to 2 years (Days +720) with the majority of assessments and procedures being neuro-oncological with some hematological assessments/procedures. At the +720 day visit, patients will be invited to participate in a long term follow-up study (TEM-LT) which will last for an additional 6 years.

Escalating doses of Temferon from Dose 1 to 5 were and will be administered to cohorts of 3 patients each as follows:

- Dose 1 – Cohort 1 (Completed)
- Dose 2 – Cohort 2 (Completed)

DocuSign Envelope ID: A0702825-E0BD-46DB-A7D6-FEF901E11877

- Dose 3 – Cohort 3 and 4, 5 (Completed)
- Dose 4 – Cohort 6 (Completed) and 7
- Dose 5 - Cohort 8

Cohorts 4 and 5, which recruited and ran in parallel, each included 3 patients who received the same dose of Temferon as in Cohort 3, but a lower dose of non-manipulated $CD34^+$ cells compared to Cohorts 1 to 3. Furthermore, patients in Cohort 5 received a different conditioning regimen, busulfan and thiotepa), compared to patients enrolled in Cohort 1-4.

Cohort 6 ran sequentially after Cohort 4 and included 3 patients. The conditioning regimen in Cohorts 6 was the same as in Cohorts 1-4 (BCNU and thiotepa). The dose of Temferon in Cohort 6 was 3×10^6 per kg body weight.

Cohorts 7 and 8 will recruit and run sequentially and will include 3 patients each. The conditioning regimen will be busulfan monotherapy. The dose of Temferon in Cohort 7 will be 3×10^6 per kg body weight, while the dose of Temferon in Cohort 8 will be 4×10^6 per kg body weight.

Following completion of dosing in Cohorts 7 and 8 and review of data by the DMC, an additional 3 patients will receive Temferon at a dose selected from either Cohort 7 or 8 in order to provide additional safety data before progression to Part B of the study.

In the event that GBM disease progression occurs, patients will be managed with second line therapies including second surgery, TMZ, BCNU, fotemustine or any other approved therapy for GBM.

DocuSign Envelope ID: A0702825-E0BD-46DB-A7D6-FEF901E11877

3.2 Intervention Scheme

Surgical resection of tumor and confirmation of MGMT unmethylated promoter status

Informed consent & eligibility criteria

HSPC mobilization and harvest (approximately 5-7 days duration)

Lenograstim 10 µg/kg/day (divided in 2 doses) s.c. for 3 days. CD34+ cell counts will be checked daily during leukapheresis
Plerixafor 0.24 mg/kg s.c. at 5h before commencement of leukapheresis

Radiotherapy for 6 weeks

Conditioning regimen (Cohorts 1-4 and 6)*

Day -6: BCNU 400mg/m^2 given by IV infusion over 1 hour

Day -5: thiotepa 5mg/kg will be given twice (once in the morning and once 12 hours later) as an IV infusion over 2 hours.

Conditioning regimen (Cohort 5)*

Day -5: thiotepa 5mg/kg will be given twice (once in the evening and once 12 hours later on Day -4) as an IV infusion over 2 hours.

Day -4: busulfan 3.2 mg/kg ideal body weight given by IV infusion over 3 hours

Day -3: busulfan dose determined according to TDM

Day -2: if necessary, based on D-3 TDM, another busulfan dose may be administered

Conditioning regimen (Cohort 7 and 8)*

Day -4: busulfan 3.2 mg/kg ideal body weight given by IV infusion over 3 hours

Day -3: busulfan dose determined according to TDM

Day -2: if necessary, based on D-3 TDM, another busulfan dose may be administered

Experimental treatment (Cohorts 1-3)

Day 0: Infuse 3 x 10^6 non-manipulated HSPCs/kg followed by Temferon (Dose Level 1-3)

Experimental treatment (Cohorts 4 & 5)

Day 0: Infuse 2 x 10^6 non-manipulated HSPCs/kg followed by Temferon (Dose Level 3)

Experimental treatment (Cohort 6)

Day 0: Infuse 2 x 10^6 non-manipulated HSPCs/kg followed by Temferon (Dose Level 4)

Experimental treatment (Cohort 7 and 8)

Day 0: Infuse 3 x 10^6 non-manipulated HSPCs/kg followed by Temferon (Cohort 7: Dose Level 4) (Cohort 8: Dose Level 5)

Follow up

Day +30: Hematologic recovery assessment
Day +60, +90, +120 and every 60 days thereafter up to Day +720:
Clinical assessments for safety, GBM progression and biological activity

*Conditioning regimen, dose of non-manipulated cells and dose of Temferon for Part B will be discussed and agreed following completion of Part A with the DMC

3.3 Rationale of Study Design

The subgroup of GBM patients with unmethylated MGMT promoter represents a suitable clinical setting for a first-in-human testing of IFN-α gene therapy as it is an area of high unmet medical need. It allows data to be acquired on safety, feasibility and biological activity of the investigational ATMP, within a framework that potentially could provide therapeutic benefit that will be documented using widely accepted secondary endpoints. The experimental treatment is embedded in a treatment program delivering state-of-the-art therapy to all enrolled patients. We propose a phase I/IIa study in GBM patients undergoing first-line treatment, designed according to principles already employed for the first testing of HSPC gene therapy in rare genetic diseases, where the clinical protocols were designed to potentially deliver therapeutic efficacy already in the first treated patients in order to justify the risks associated with administration of a pre-transplant conditioning regimen and the first-in-human exposure to the ATMP.

3.4 Study Phases

Seven protocol phases are foreseen:

1. *Enrolment*, with signing of the informed consent form.

2. *Screening and eligibility-determining phase*, during which inclusion/exclusion criteria will be assessed (this will occur approximately 7 to 8 weeks prior to Temferon administration on Day 0).

3. *HSPC harvesting* (within 4 weeks following Screening), which will occur before standard of care treatment with radiotherapy administered for 6 weeks.

4. *Temferon production phase*, following HSPC harvest and up to Temferon release for clinical use by qualified person (QP). This will occur while standard of care treatment with radiotherapy occurs.

5. *Temferon treatment phase:* Once the Investigator receives confirmation that Temferon is available to administer to the patient, the conditioning regimen will be administered at Baseline (Cohorts 1 to 4 and 6: BCNU administered to the patient on Day -6, followed by thiotepa on Day -5; Cohort 5: Thiotepa on Day -5 (evening) and -4 (morning), Busulfan on Day -4 (evening) to Day -2); Cohorts 7 and 8, Busulfan

monotherapy on Day -4 (evening) to Day -2. Non-manipulated HSPCs and Temferon will be infused on Day 0.

6. *Follow-up phase:* from Temferon infusion until Days +720 following experimental treatment.

7. *End of study phase:* At the end of the study (+720 days), patients will be invited to continue long term follow up (TEM-LT) as required by the authorities (up to 8 years in Italy after gene therapy administration). Additional written informed consent will be required to participate in this long term follow up.

3.4.1 Enrolment

The Investigator will select potential eligible patients, inform them about the study and obtain their written informed consent. Patients will be provided adequate time to ask questions about the study, including the treatment and the required procedures, before obtaining their informed consent. The Investigator must not start any study-specific procedure before the Informed Consent Form (ICF) has been signed and dated by both the patient or legal representative (and impartial witness, if applicable) and the Investigator.

The consent process will be performed in accordance with ICH Good Clinical Practice (GCP), local applicable regulations and institutional practice.

Patients considered by the Investigator to be eligible and for who written informed consent has been obtained will enter the Screening phase.

3.4.2 Screening

Patients who are considered by the Investigator to be eligible and for whom written informed consent has been provided will enter the Screening Phase (approximately 7-14 days duration) and undergo the tests and procedures necessary to confirm study eligibility. Inclusion and exclusion criteria will be checked.

Patients who are confirmed to be eligible, based on screening assessments, will proceed to HSPC harvesting.

DocuSign Envelope ID: A0702825-E0BD-46DB-A7D6-FEF901E11877

3.4.3 HSPC harvest

Patients will undergo HSPC mobilization with lenograstim and plerixafor and harvest by leukapheresis, as per institutional practice. The harvested cells will be selected for the $CD34^+$ marker to enrich for HSPCs. The mobilization target for Cohorts 6 and 7 is $\geq 12 \times 10^6$ $CD34^+$ cells/kg, of which 7×10^6 $CD34^+$ cells/kg will be used for Temferon production, while a minimum of 5×10^6 $CD34^+$ cells/kg will be frozen as non-manipulated apheresis product. In patients who mobilise poorly additional leukapheresis may be permitted, at the discretion of the Investigator, after completion of radiotherapy should additional non-manipulated $CD34^+$ HSPCs be required for co-administration or as a backup. If the target number of cells cannot be collected after at least 2 attempts at HSPC collection, then the patient may be withdrawn from the study.

Following HSPC harvest, patients will receive treatment with radiotherapy but without TMZ for approximately 6 weeks. This treatment is not part of this research protocol and is not discussed further but adverse events that occur during this time will be recorded as discussed in Section 6.3.

3.4.4 Temferon production

The freshly harvested cells obtained at leukapheresis will be transported to the CMO [redacted] for $CD34^+$ cells enrichment. After purification, autologous $CD34^+$ cells will be exposed to transduction with the TIA126 LV and stored frozen in cryopreservative solution while aliquots are being tested for release.

3.4.5 Temferon treatment

At least 7 to 28 days following completion of radiotherapy, a number of assessments should be completed prior to administration of conditioning to ensure the patient is still eligible for the study. A time window of up to 14 days prior to Baseline visit (defined as the first day of conditioning) has been provided to ensure these assessments are performed (See Table 12-1), and for Cohorts 7 and 8 this has been extended to 21 days (Table 12-2).

Administration of Temferon will only occur once radiotherapy has completed and providing the daily requirements for dexamethasone (or another corticosteroid at an equivalent dose) is less than or equal to 4mg daily. In the event that the dexamethasone dose (or equivalent corticosteroid dose) > 4mg qd is required, the Investigator will have a period of 3 weeks from

DocuSign Envelope ID: A0702825-E0BD-46DB-A7D6-FEF901E11877

the time of receipt of the post-radiotherapy/baseline brain MRI images to control cerebral edema or manage clinical symptoms with alternative approved therapies (bevacizumab is prohibited) with the aim of reducing corticosteroid requirements to a dexamethasone (or equivalent) dose ≤4mg qd. In the event that this dose target cannot be reached, the Investigators will review the case with the Sponsor in order to discuss and agree the rationale for continuation of the patient in the study. Experimental treatment (Baseline through Day 0) will consist of reduced intensity conditioning; Cohorts 1-4 and 6 with BCNU (Day -6, 400mg/m^2 IV) and thiotepa (Day -5, two doses of 5mg/kg IV, once in the morning and once 12 hours later); Cohort 5 with thiotepa (Day -5 evening, 5mg/kg IV, Day -4 morning, 5mg/kg IV) and busulfan (Day -4 evening, busulfan 3.2 mg/kg ideal body weight IV, with TDM performed to adjust, if necessary, the dose of busulfan to administer on Day -3 and/or, if required, on Day -2). Cohorts 7 and 8 will undergo conditioning with busulfan monotherapy (Day -4 evening, busulfan 3.2 mg/kg ideal body weight IV, with TDM performed to adjust, if necessary, the dose of busulfan to administer on Day -3 and/or, if required, on Day -2). On Day 0, infusion of autologous non-manipulated HSPCs will occur followed by infusion of Temferon (within 3h to 12h of autologous non-manipulated HSPCs infusion). Patients will be monitored in-hospital until hematological recovery occurs.

3.4.6 Follow-up

Patients will enter the follow-up phase from Day +1 until Day +720 post Temferon infusion for safety, efficacy and exploratory assessments. During this period patients will be carefully monitored for GBM disease progression. Once progression is confirmed, second line treatment including second surgery or chemotherapy may be commenced according to Investigator's choice.

It is essential that daily complete blood counts are performed from D+1 until the day of discharge from the transplant unit following ASCT and these data are recorded in the eCRF.

At Day +60, +90 and +120, a time window of ±7 days is permitted to conduct the scheduled visit. Following Day +120, patients will be reviewed every 60 days and a time window for each scheduled visit is ±30 days to allow for sufficient time for repeat brain imaging to assess disease progression. Any scheduled visit occurring outside of the permitted window should be recorded as a protocol deviation.

DocuSign Envelope ID: A0702825-E0BD-46DB-A7D6-FEF901E11877

3.4.7 End of study / long term follow-up study

Upon completion of the TEM-GBM_001 study at Day +720 post-Temferon infusion, patients will be invited to participate in a separate long-term follow-up study, TEM-LT (according to applicable regulations) to further assess the long-term safety and efficacy of Temferon treatment.

DocuSign Envelope ID: A0702825-E0BD-46DB-A7D6-FEF901E11877

4 Patient Selection and Withdrawal Criteria

4.1 Selection of Study Population

Approximately 27 patients will be treated in Part A and followed up at two centers in Milan (INCB and OSR). A center in Rome will participate for Part B of the study. INCB is a specialist referral center for GBM in Italy and therefore patients may be resident outside the Milan or Lombardy region. OSR is the Italian leading center for rare disease gene therapy, and the OSR neurosurgical team may also identify patients with GBM. Policlinico Universitario Fondazione Agostino Gemelli (PG) in Rome has been selected as this center has both neurosurgical and transplant hematology expertise for this study. Patients will be assigned to study treatment only if they meet all the inclusion criteria and none of the exclusion criteria.

Deviations from the inclusion and exclusion criteria are not allowed because they can potentially jeopardize the scientific integrity of the study, regulatory acceptability, or patient safety. Therefore, adherence to the criteria as specified in the protocol is essential.

4.1.1 Inclusion Criteria

Each patient must meet all of the following criteria to be enrolled in this study:

At Screening:

- Patients aged between 18 and ≤ 70.
- Histologically confirmed, newly diagnosed supratentorial glioblastoma with unmethylated MGMT gene promoter.
- Patients have undergone complete or partial tumor resection.
- Able and willing to provide written informed consent and comply with the study protocol and procedures.
- Eligible for radiotherapy.
- Life expectancy of 6 months or more at Screening.
- Women of child-bearing potential enrolled in the study must have a negative pregnancy test at screening and agree to use two distinct acceptable methods of contraception during the trial.

DocuSign Envelope ID: A0702825-E0BD-46DB-A7D6-FEF901E11877

- Men enrolled in the study with partners who are women of childbearing potential, must be willing to use an acceptable barrier contraceptive method during the trial or have undergone successful vasectomy at least 6 months prior to entry into the study. Successful vasectomy needs to have been confirmed by semen analysis.
- Karnofsky performance score (KPS)≥70.

Within 20 days of Temferon administration:

- Adequate cardiac, renal, hepatic and pulmonary function as evidenced by:
    - Left ventricular ejection fraction (LVEF) ≥ 45% by echo and normal electrocardiogram (ECG) or presence of abnormalities not significant for cardiac disease. Absence of severe pulmonary hypertension;
    - Diffusing capacity of the lung for carbon monoxide (DLCO) >50% and forced expiratory volume in 1 sec (FEV1) and forced expiratory vital capacity (FVC) > 60% predicted (if non cooperative: pulse oximetry > 95 % in room air);
    - Serum creatinine < 2x upper limit normal and estimated glomerular filtration rate (eGFR) ≥ 30ml/min/1.73m^2;
    - Alkaline phosphatase (ALP), alanine aminotransferase (ALT) and/or aspartate aminotransferase (AST) ≤ 2.5 x upper limit of normal (ULN), and total bilirubin ≤ 2.0 mg/dl.
- Hemoglobin ≥10 g/dL, platelet count ≥100000/mm^3, absolute neutrophil count >1500/mm^3.

4.1.2 Exclusion Criteria

Patients meeting any of the following criteria will be excluded from the study:

- Use of other investigational agents or procedures within 4 weeks prior to study enrolment (within 6 weeks if use of long-acting agents) or participation in a previous gene therapy study.
- Known hypersensitivity to carmustine (or any other nitrosurea), thiotepa, busulfan, lenograstim, plerixafor, or any excipients used in these products.

DocuSign Envelope ID: A0702825-E0BD-46DB-A7D6-FEF901E11877

- Receipt of any oral or parenteral chemotherapy or immunotherapy within 2 years of Screening.
- Previous allogeneic bone marrow transplantation, kidney or liver transplant.
- Clinical evidence of persistent raised intracranial pressure following surgical resection.
- Clinically relevant active viral, bacterial, or fungal infection at eligibility evaluation.
- Active autoimmune disease or a relevant history of important autoimmune manifestations, in particular psoriasis, systemic lupus erythematosus (SLE), rheumatoid arthritis, vasculitis, immune-mediated peripheral neuropathies.
- History of sarcoidosis.
- History or current evidence of neuropsychiatric illness including depression, schizophrenia, bipolar disorders, impaired cognitive function, dementia or suicidal tendency.
- History of severe cardiovascular disease such as prior stroke, coronary artery disease requiring intervention or unresolved arrhythmias in the past 6 months.
- Evidence of any hematological neoplasm.
- Positivity for human immunodeficiency virus type 1 or 2 (HIV-1, HIV-2) (serology or RNA), and/or Hepatitis B Virus Surface Antigen (HbsAg) and/or Hepatitis B Virus (HBV) DNA and/or Hepatitis C virus (HCV) RNA (or negative HCV RNA but on antiviral treatment) and/or Treponema Pallidum or Mycoplasma active infection.
- Active alcohol or substance abuse within 6 months of the study.
- Current pregnancy or lactation.
- Known bleeding diathesis or history of abnormal bleeding, or any other known coagulation abnormalities that would contraindicate lumbar puncture for CSF or future surgery.
- Use of immunosuppressants with the exception of steroids. The maximum permitted dexamethasone (or equivalent) dose is 4 mg per day.

4.2 Withdrawal of Patients from the Study

Each patient's study participation duration is expected to be approximately 26 months from the date signed written informed consent is provided through the last follow-up visit on Day

DocuSign Envelope ID: A0702825-E0BD-46DB-A7D6-FEF901E11877

+720 post Temferon infusion. After completion of the study, patients will be invited to consent for a long-term follow-up study of another 6 years, to reach up to 8 years total follow-up post-Temferon infusion.

4.2.1 Reasons for Withdrawal/Discontinuation

Patients may withdraw from the study at any time and for any reason without prejudice to their future medical care by the Investigator or at the study site. Should a patient withdraw from the study, the Investigator should ensure that the patient's clinical care is continued. Every effort should be made to keep patients in the study in order to monitor their safety following administration of Temferon. Should patients withdraw their consent for specific assessments following Temferon infusion (e.g. bone marrow biopsy, blood sampling, etc.), a protocol deviation should be recorded but other assessments should be completed as specified by the protocol.

Under these circumstances the documentation should be completed as thoroughly as possible, meaning that a final examination and documentation according to the protocol should be done if feasible. Reasons for premature study termination should be listed and documented on the case report form (CRF) and in the medical record. Appropriate further treatment and follow up outside the study should be ensured. The patient's general practitioner should be informed about the termination if necessary, provided the patient agrees. An attempt should be made at least to assess the patient's survival status by telephone follow-up unless informed consent for documentation has been withdrawn.

In case a patient's study participation has been stopped prematurely, the CRF pages must be completed until the date of termination.

The reasons for patients not completing the study will be recorded.

A patient may be withdrawn from the study for any of the following reasons:

1. Does not meet the protocol eligibility criteria;

2. Lost to follow-up;

3. The patient withdraws consent for participation in the study;

DocuSign Envelope ID: A0702825-E0BD-46DB-A7D6-FEF901E11877

4. Adverse event(s) occur that put the safety of the patient at risk should they continue in the study;

5. Major protocol deviation prior to Temferon administration (the Investigator should discuss the protocol deviation with the medical monitor before withdrawing the patient);

6. Investigator decides to discontinue the patient's participation in the study;

7. Sponsor decides to discontinue the patient's participation in the study;

8. Other (e.g., death, inability to attend scheduled clinic visits, Temferon not administered, etc.).

4.2.2 Handling of Withdrawals

Patients are free to withdraw from the study or study treatment at any time upon request. Patient participation in the study may be stopped at any time at the discretion of the investigator or at the request of the Sponsor. When a patient withdraws from the study, the reason(s) for withdrawal shall be recorded by the investigator on the relevant page of the CRF. Whenever possible, all patients who withdraw from the study following ATMP administration will undergo all end-of-study assessments.

Patients who fail to return for final assessments will be contacted by the site in an attempt to have them comply with the protocol. Appropriate contact includes 2 documented phone calls followed by 1 registered letter before a patient is listed as lost to follow up. It is vital to obtain follow-up data on any patient who is withdrawn from the study and has an ongoing AE or serious AE (SAE). In every case, efforts must be made to undertake protocol-specified, safety, follow-up procedures.

4.2.3 Replacements

In the event that a patient withdraws from the study prior to Temferon administration, a replacement patient will be recruited. In the event that a patient withdraws prior to the Day +30 assessments, a replacement patient may be recruited providing adequate supply of TIA126 LV is available to produce Temferon. Discussion and agreement with Genenta Science should occur.

DocuSign Envelope ID: A0702825-E0BD-46DB-A7D6-FEF901E11877

4.2.4 Temporary suspension of the clinical study

In the event that a patient death occurs within 90 days of Temferon administration that is attributed to Temferon, further enrolment will be suspended by the Sponsor until the risk to the patient is mitigated. An independent DMC will meet on an *ad hoc* basis to review and assess the event and it will be the responsibility of the DMC to make a recommendation to the Sponsor whether enrolment to the cohort or study should be suspended.

The regulatory authority, the Ethics Committee, the Investigators, and other appropriate institutional regulatory bodies will be promptly notified by the Sponsor, if a decision to suspend enrolment is made. If enrolment is suspended, all treated patients will continue to be monitored through their completion of the study or withdrawal.

5 Treatment plan

5.1 Method of Assigning Patients to Treatment Groups

At the Screening visit, patients will be assigned an enrolment code. After confirming that a patient meets all eligibility criteria, the INCB will register the patient in the CRF as eligible for treatment with Temferon.

For Cohorts 4 and 5, which ran in parallel, alternate patients were allocated to each cohort after eligibility criteria were confirmed at Screening. For example, the first patient identified entered Cohort 4 and the next was allocated to Cohort 5.

Cohorts 6-8 will run sequentially. For each cohort, a minimum of 30 days will separate dosing of the first patient and commencement of conditioning for the second patient within the cohort. Conditioning of the third patient will occur a minimum of 30 days after the second patient received Temferon.

Progression from Cohort 6 to 7, and from Cohort 7 to 8 will be subjected to DMC recommendation. Following completion of dosing in Cohorts 7 and 8 and review of data by the DMC, an additional 3 patients will receive Temferon at a dose selected from either Cohort 7 or 8, in order to provide additional safety data before progression to Part B of the study. No staggering is foreseen in the additional 3 patients to be dosed.

5.2 HSPC mobilization and harvest

HSPCs will be obtained from mobilized peripheral blood $CD34^+$ cells. If patients do not have a suitable peripheral access, a central venous catheter will be inserted both for the purpose of leukapheresis and infusion of unmodified $CD34^+$ cells and Temferon. After mobilization, the harvesting may occur on at least two consecutive days.

The objective is for Cohorts 7 and 8 to harvest a target dose of $\geq 14 \times 10^6$ $CD34^+$ cells/kg with as few leukapheresis sessions as possible during consecutive days. There should be sufficient cells available for production of Temferon (8×10^6 $CD34^+$ cells/kg), non-manipulated cells (3×10^6 $CD34^+$ cells/kg) and a backup (2×10^6 $CD34^+$ cells/kg). Not less than 6×10^6

DocuSign Envelope ID: A0702825-E0BD-46DB-A7D6-FEF901E11877

nonmanipulated CD34+ cells/kg should be destined for co-infusion and backup, to account for cell losses (e.g., repeated QC testing) and variabilities in cell counts/volume measurements between the ATMP service provider (CMO) and the clinical cell manipulation lab.

Patients will receive lenograstim at the dose of 10μg/kg/day divided in 2 administrations (5μg/kg each) by subcutaneous injection (s.c.) for 3 days or until a sufficient number of $CD34^+$ cells is mobilized and the leukapheresis/-es has/have been concluded. Monitoring of $CD34^+$ cells in the PB will be performed. Patients will receive plerixafor before each leukapheresis. Standard leukapheresis will be performed at 5 hours following administration of plerixafor 0.24 mg/kg s.c. and shortly after the last lenograstim administration according to the $CD34^+$ cell count and in agreement with internal departmental procedures. Lenograstim and plerixafor administration will be repeated until sufficient mobilized peripheral blood (MPB) $CD34^+$ cells are harvested. It is unlikely that multiple runs of mobilization and $CD34^+$ cells selection will be required, but if necessary, the patient will undergo a second round of mobilization after at least 2 weeks from the first round. In patients who mobilise poorly additional leukapheresis may be permitted, at the discretion of the Investigator, after completion of radiotherapy should additional non-manipulated $CD34^+$ HSPCs be required for co-administration or as a backup. If the target number of cells cannot be collected after at least 2 attempts at HSPC collection, then the patient may be withdrawn from the study.

After collection, the bag containing the leukapheresis, sealed and identified, will be labelled according to local internal procedures and standards and then transferred/shipped using Sponsor approved procedures in a biohazard container [redacted] The CMO will be informed by the Investigator of the weight of the patient and the anticipated date of Temferon infusion.

The bag containing the backup cells will be labelled and cryopreserved according to local approved standards and procedures. At least 3-4 x 10^6 non-manipulated $CD34^+$ cells/kg will be returned by the CMO and cryopreserved at each center as non-manipulated apheresis product. The remaining cells will be purified by immunomagnetic purification, considering that the target $CD34^+$ cell count (excluding the backup) before any manipulation is 3-8 x 10^6 $CD34^+$ cells/kg to achieve a minimum Temferon dose of 0.5 x 10^6 $CD34^+$ cells/kg and a maximum dose of 4 x 10^6 $CD34^+$ cells/kg after purification, manipulation and transduction. The negative fraction (CD34neg) will be sent as fresh material to the center to be potentially used together with the backup, if needed.

DocuSign Envelope ID: A0702825-E0BD-46DB-A7D6-FEF901E11877

5.3 Lenograstim and plerixafor

Lenograstim and plerixafor will be provided by the local pharmacy, according to local institutional standard operating procedures.

5.4 Temferon manufacturing & characterization

5.4.1 Temferon definition

Temferon, the Drug Product (DP), can be classified as an investigational ATMP belonging to the class of the Gene Therapy Medicinal Products (GTMPs).

Temferon consists of autologous CD34$^+$-enriched HSPCs exposed to transduction with the TIA126-LV encoding for the human IFN-α2 gene. In the final formulation, prior to QP release, cells are in a cryopreservative medium (cells are resuspended at a concentration range of 2.5-10 x10^6 cells/ml in saline solution containing 7% human serum albumin [HSA] and 10% DMSO) and frozen in polypropylene cryovials supplied by Nalgene.

Upon injection of Temferon into the patient, the gene-modified HSPCs are expected to engraft into bone marrow and to differentiate into the different myeloid lineages while selectively expressing the IFN-α2 transgene in the TEMs. The properties of Temferon are therefore based on the ability of the TIA126-LV to genetically modify the autologous CD34$^+$ HSPCs.

The LV vector production as well as Temferon manufacturing and release will be performed [redacted] according to the Current GMP (cGMP), as described in the Investigator's Brochure. [redacted] has been authorized by the Italian Medicine Agency (AIFA) according to D.L.vo 211/2003 and D.L.vo. 219/2006 for the production of specific medicinal products obtained with particular technologies (i.e. cell and gene therapy) in sterile condition, and for primary packaging operations. The area dedicated to product manipulation has been built in compliance with cGMP and has been authorized by the Italian Ministry of Health according to D.L.vo 206/01 for the manipulation of genetically modified micro-organisms.

5.4.2 Drug substance and drug product manufacturing

CD34$^+$ HSPCs will be genetically modified by means of a third-generation LV to express the human IFN-α2 gene.

DocuSign Envelope ID: A0702825-E0BD-46DB-A7D6-FEF901E11877

5.4.2.1 Lentiviral vector

The TIA126-LV used for preparation of Temferon is a replication-defective third-generation pseudotyped vector. Details regarding the structure of the vector are contained in the Investigator's Brochure. From a quality perspective, each batch of LV vector will be characterized for viral particles and titer, infectivity, viral proteins, vector integrity and identity (transgene and regulatory sequences), for the presence of selected impurities related to the producer cells or the manufacturing process and for the absence of microbial contaminants (bacteria, fungi, mycoplasma and viruses) and RCL.

5.4.2.2 $CD34^+$ cell isolation and transduction

Upon arrival at the manufacturing site, total mobilized peripheral blood samples (leukapheresis) will immediately be processed to isolate $CD34^+$ cells by immunomagnetic purification. Thereafter, $CD34^+$ cells will immediately be transduced. For transduction, $CD34^+$ will be seeded (day 0) in RetroNectin®-coated bags at the target concentration of $1x10^6$ cells/ml in the CellGro serum-free medium (Cell Genix) supplemented with GMP-grade or clinical-grade cytokines (TPO 100 ng/ml, IL3 60 ng/ml, SCF 300 ng/ml, FLT3L 300 ng/ml) for 22 ± 2 hours. On day 1, $CD34^+$ cells will be transduced by adding $1x10^8$/ml Transducing Units of LV (Multiplicity of Infection 100) and incubated for 14±1 hours over-night (transduction step). On day 2, at the end of transduction, the cells will be processed for cryopreservation.

Aliquots will be retained for the Quality Control (QC) characterization (see Investigator's Brochure for detailed list of QC tests and specifications).

5.4.2.3 Cryopreservation of $CD34^+$ cells

On day 2, at the end of transduction phase, cells will be harvested, centrifuged and resuspended in CellGro medium. Cells are then re-buffered in saline solution 7% HSA, washed, centrifuged, resuspended in freezing medium constituted by saline solution, HSA 7% and DMSO 10% at a concentration of 2.5-10 x 10^6 cells/ml and cryopreserved under vapor of liquid nitrogen. This now constitutes the DP, Temferon.

DocuSign Envelope ID: A0702825-E0BD-46DB-A7D6-FEF901E11877

5.4.2.4 Temferon packaging and labelling

Temferon will be vialed in saline containing 7% HSA and 10% DMSO, in one or more sterile primary package(s) (Nalgene polypropylene cryovials) of the suitable volume. Temferon will be labelled with a unique identification code of the patient and of the product, released [redacted] according to cGMP. A secondary package, again to be labelled with a unique identification code of the patient and of the product, will be used to transfer Temferon to the center's Bone Marrow Transplant Unit.

5.4.2.5 Preparation of Temferon for intravenous infusion

Preparation of Temferon for patient administration will be performed by trained personnel at the clinical center's Bone Marrow Transplant Unit. These units hold a JACIE accreditation for cellular therapy, and the units are authorized for the manipulation of genetically modified material. Thawing and infusion of the non-manipulated HSPCs (not considered part of the DP, and therefore handled by the JACIE-certified Cell Manipulation Laboratory at each center) will be performed before Temferon infusion, according to internal standard operating procedures.

Either on the day of administration (OSR only) or prior to initiation of conditioning (PG), Temferon, frozen in cryovials, will be transferred [redacted] to the clinical center, in a liquid nitrogen dry shipper. On arrival at the Bone Marrow Transplant Unit, unless thawing of Temferon is to occur immediately, storage of the cryovials should occur at ≤-65ºC in an approved storage area for clinical grade cell and gene therapy.

Temferon dose varies according to the dose level cohort and will be expressed as the number of $CD34^+$ cells/kg body weight of the patient, as per general practice for stem cell products (including the previously infused, non-manipulated autologous HSPCs). The appropriate number of cryovials containing the target Temferon cell dose for a given patient will be immerged in a 37°C water bath. After thawing, the vial content(s) will be resuspended and aspirated into a single use 5ml syringe attached to an 18G needle and injected into a 100ml infusion bag containing normal saline and 7% human serum albumin. After reconstituting the DP in the in the infusion bag and appropriate mixing by inverting the bag several times, a 1 ml aliquot (<1% of total amount) will be removed to monitor cell number of the actually infused product (for information only, data will be available post-infusion). The diluted Temferon

product will be infused IV into the patient following the principles outlined in internal standard operating procedures. Infusion shall take no more than 15 minutes, and the infusion bag will be flushed with normal saline to recover residual Temferon from the bag and the infusion line.

5.4.2.6 Temferon supply, storage and accountability.

The handling and administration of Temferon will be recorded by Investigator or designee in the DP record log in the site file.

After Temferon infusion, the study coordinator at the site will send to Genenta Science via e-mail the documentation certifying the receipt of Temferon, injection of Temferon and waste disposal. Waste disposal should occur as specified in internal guidelines.

The Investigator is responsible for the accountability and traceability of the product.

5.5 Conditioning regimen

In the current study, all patients will receive a reduced toxicity conditioning regimen prior to administration of non-manipulated cells and Temferon. All conditioning agents will be provided by the local pharmacy, according to institutional standard operating procedures.

- BCNU & Thiotepa (Cohorts 1 to 4 and Cohort 6),
- Busulfan & Thiotepa (Cohort 5)
- Busulfan monotherapy (Cohorts 7 and 8)

On Day -6, BCNU 400 mg/m^2 will be given by IV infusion over 1 hour. On Day -5, thiotepa 5mg/kg will be given twice (once in the morning and once 12 hours later) as an IV infusion over 2 hours. In obese patients, the dose of both BCNU and thiotepa may be reduced and based on adjusted ideal body weight, according to institutional standard operating procedures.

5.5.1 Busulfan & Thiotepa (Cohort 5)

Patients will receive two doses of thiotepa 5mg/kg as an IV infusion over 2 hours, the first on Day-5 (evening) and the second on Day-4 (morning), approximately 12 hours apart from the first dose. Instructions for infusion of busulfan are listed in Section 5.5.2.

DocuSign Envelope ID: A0702825-E0BD-46DB-A7D6-FEF901E11877

5.5.2 Busulfan monotherapy (Cohorts 7 and 8)

Patients will receive a single daily intravenous busulfan dose, starting on Day -4 at 3.2 mg/kg, based on adjusted body weight (ABW) calculated according to the following formula:

- ABW = ideal body weight (IBW) + 0.4 x (measured body weight – IBW).

In underweight patients (measured body weight below the ideal body weight), the measured body weight will be used for dose calculations.

Patients will receive a total of 2-3 busulfan doses, administered by a 3-hour infusion and given every 24 hours from Day -4 to day -2. Therapeutic drug monitoring will be performed in order to target a total area under the busulfan plasma concentration curve (AUC) of 60 mg·h/L.

The pharmacokinetic samples will be collected starting Day -4 and, if required, Day -3 according to the following sampling scheme:

- pre-dosing (time 0, before busulfan infusion)
- +5 minutes after the end of busulfan infusion
- +2h after the end of busulfan infusion
- +4h after the end of busulfan infusion
- +6h after the end of busulfan infusion

After the first dose, a predicted cumulative AUC will be calculated based on the assumption that no dose adjustments will be made for subsequent doses. If the predicted cumulative AUC, based on the assumption that no additional dose adjustments will be made, is found to be between 55 and 60 mg·h/L, then the Investigator (or delegate) may use their discretion to either not modify the next dose, or to modify the dose in order to target AUC to the higher or lower end of the target range, according to clinical considerations.

If the predicted cumulative AUC is outside the target range, a dose adjustment must be performed. If there are clinical or pharmacokinetic analytical reasons to suspect that a calculated AUC value is unreliable, this should be documented in the pertinent sections of the

DocuSign Envelope ID: A0702825-E0BD-46DB-A7D6-FEF901E11877

CRF and the busulfan pharmacokinetic report, and the Investigator (or delegate) should use their judgment to determine the subsequent dose adjustment.

Dose adjustments will be performed based on the following AUC parameters:

- $mAUC_{BuD1}$ = AUC(0-∞) after the 1st dose
- $mAUC_{BuD2}$ = AUC(0-24h) after the 2nd dose (conditional, see dose adjustment procedures below)

Dose adjustment procedures:

(a) Dose adjustment for busulfan Dose 2 on Day -3, after the 1st administration (Dose 1)

A total AUC following 2 days of busulfan treatment (AUC_{BuD1+2}) is predicted based on the measured AUC after the first busulfan administration ($mAUC_{BuD1}$), as follows:

Predicted (p) $AUC_{BuD1+2} = mAUC_{BuD1}$ x2

Dose adjustment for the second Busulfan administration on day-3 (Dose 2) should be performed to target total AUC (AUC_{BuTot}) between 45 mg·h/L and 60 mg·h/L. Dose 2 is calculated according to the following formula:

Calculated Dose 2 = Dose 1 x (target $AUC_{BuTot} - mAUC_{BuD1}$) / ($mAUC_{BuD1}$)

If calculated Dose 2 results higher than 4.8 mg/kg, the dose will be split over Day -3 and Day -2, administering 50% on day -3 followed, if possible, by a second round of therapeutic drug monitoring.

(b) Dose adjustment for Dose 3 on Day -2, after the 2nd administration (Dose 2)

If a third dose (Dose 3) of busulfan administration on Day -2 is planned, therapeutic drug monitoring after administered D2 is recommended.

Predicted $AUC_{BuTot} = mAUC_{BuD1} + (mAUC_{BuD2} \times 2)$

Dose 3 adjustment may be performed, at the discretion of the investigator:

DocuSign Envelope ID: A0702825-E0BD-46DB-A7D6-FEF901E11877

Dose 3 = Dose 2 x (target AUC_{BuTot} – $mAUC_{BuD1}$ – $mAUC_{BuD2}$) / ($mAUC_{BuD2}$)

Further details of pharmacokinetic sampling, analysis and dose adjustment will be provided in the SOM.

If the decision will be to progress to Part B, therapeutic busulfan drug monitoring may be omitted, and instead patients may instead receive a fixed-dose busulfan conditioning regimen.

5.6 Infusion of non-manipulated, cryopreserved apheresis

This will be performed according to institutional practice and standard procedures for autologous stem cell transplantation.

On Day 0, at least 60 hours after completion of thiotepa infusion (Cohorts 1 to 3), patients will receive an infusion of non-manipulated stem cell support at a dose of 3 x 10^6 $CD34^+$ cells/kg.

For Cohorts 4 and 6 on Day 0, at least 60 hours after completion of thiotepa infusion, patients will receive an infusion of non-manipulated stem cell support at a dose of 2 x 10^6 $CD34^+$ cells/kg which may be adjusted according to the Temferon VCN (Table 5-1). For Cohort 5, at least 36 hours after completion of busulfan infusion (and at least 60 hours following the last thiotepa dose), patients will receive an infusion of non-manipulated stem cell support at a dose of $2x10^6$ $CD34^+$ cells/kg which may be adjusted according to the Temferon VCN (Table 5-1).

For Cohorts 7 and 8, on Day 0, at least 36 hours after completion of busulfan infusion, patients will receive an infusion of non-manipulated stem cell support at a dose of 3 x 10^6 $CD34^+$ cells/kg.

DocuSign Envelope ID: A0702825-E0BD-46DB-A7D6-FEF901E11877

5.7 Dosing of Temferon

5.7.1 Dose Levels and Dose Adjustments

Table 5-1 Dosing scheme for Temferon

Cohort	Dose Level	**Temferon** ($CD34^+$/kg)*
**Cohort 1**	**1**	0.5 x10^6
**Cohort 2**	**2**	1.0 x10^6
**Cohort 3**	**3**	2.0 x10^6
**Cohorts 4 and 5**	**3**	2.0 x10^6
**Cohort 6 and 7**	**4**	3.0 x10^6
**Cohort 8**	**5**	4.0 x10^6
* Temferon dose adjustments will be performed according to the *in-vitro* VCN indicated in the release documents.		

Note that the Temferon dose provided for injection will be at least that specified in Table 5-2 and Table 5-3 with an excess number of cells of up to 10% permitted to be administered.

Temferon dose adjustments may be required according to the VCN of the DP (Table 5-3). The dose of Temferon will be adjusted by the Sponsor, in agreement with the Principal Investigator. If necessary, the VCN will be reported to 2 decimal places to assist with dose adjustment decisions.

Table 5-2 Temferon dose adjustments will be performed according to VCN

VCN	Dose Level 1 (cells/kg)	Dose Level 2 (cells/kg)	Dose Level 3 (cells/kg)	Dose Level 4 (cells/kg)	Dose Level 5 (cells/kg)
< 0.4	0.8 x10^6	1.5 x10^6	3.0 x10^6	≥3.0 x10^6	≥4.0 x10^6
0.4-1.0	0.5 x10^6	1.0 x10^6	2.0 x10^6	3.0 x10^6	4.0 x10^6
1.1-1.5	0.4 x10^6	0.8 x10^6	1.5 x10^6	2.3 x10^6	3.0 x10^6
>1.5	0.3 x10^6	0.5 x10^6	1.0 x10^6	1.5 x10^6	2.0 x10^6

No adjustment in the supporter cell dose is required for Cohorts 7 and 8 as a minimum total $CD34^+$ cell dose > 4 x 10^6/kg is guaranteed in all circumstances.

Should the patient not have enough Temferon cells available for his planned dose level, the maximum number of available cells, but not less than 1.5 x 10^6/kg, will be infused.

The Investigators will discuss with the Sponsor the possibility of replacing the patient in the original cohort.

If the decision will be to progress to Part B, the dose of non-manipulated $CD34^+$ support cells and dose of Temferon to be evaluated will be determined from data obtained in Part A. Data from sequential patients will be reviewed in an ongoing manner to evaluate whether the dose should be modified for subsequent patients recruited according to the benefit:risk determination. Data to be used for this evaluation includes:

- Safety data according to CTCAE criteria
- Routine clinical and laboratory data
- *In vivo* engraftment of gene modified cells based on bone marrow and peripheral blood VCN
- Assessment of autoimmune manifestations attributed to use of Temferon
- Significant organ toxicities (≥grade 3 according to CTCAE v5.0) that can be ascribed to IFN exposure that are attributed to Temferon exposure
- Extra-tumor detection of IFNα gene signatures (peripheral blood, bone marrow, CSF) and IFNα concentrations (peripheral blood and CSF)
- Tumor specific criteria (imaging parameters, immunohistochemistry and IFNα gene signatures obtained from any samples available at second surgery)

5.7.2 Cohort Assignment

In Part A, the first patient assigned to the first cohort received a single IV infusion of Temferon at Dose Level 1 (Table 5-2). Beginning on the day of treatment (Day 0), the patient was monitored for safety and treatment effects during protocol-specified study visits. For each cohort, a minimum of 30 days will separate dosing of the first patient and commencement of conditioning for the second patient within a cohort. Conditioning of the third patient will occur a minimum of 30 days after the second patient received Temferon. The DMC will review the

DocuSign Envelope ID: A0702825-E0BD-46DB-A7D6-FEF901E11877

safety and efficacy data within a dosing cohort and make a recommendation about proceeding to the next dose level. Should there be insufficient Temferon engraftment in the bone marrow of the first 2 patients in a cohort (i.e. VCN < 0.07), the DMC will review the safety and efficacy data for the 2 subjects and make a recommendation about proceeding to the next dose level.

Patients were alternately allocated in Cohorts 4 and 5 and were dosed in parallel with Temferon Dose Level 3 after the DMC reviewed the safety and efficacy data of Cohort 1-3. Within each cohort, the 30 day interval was applied between Temferon dosing of a patient and commencement of conditioning for the next patient.

Patients in Cohort 6 were administered with Temferon Dose Level 4. Following DMC review of safety and efficacy data from Cohorts 1-6, a recommendation was to include Cohort 7 and 8 where the 30-day interval will continue between Temferon dosing of a patient and commencement of conditioning for the next patient.

The DMC will review the safety and efficacy data for Cohort 7 and subsequently for Cohort 8. Following their review and recommendation, an additional 3 patients will receive Temferon at a dose selected from either Cohort 7 or 8 in order to provide additional safety data before progression to Part B of the study. No staggering is foreseen in the additional 3 patients to be dosed.

5.7.3 Dose-Limiting Toxicity (DLT) and Maximum Tolerated Dose (MTD)

A DLT is defined as any CTCAE Grade 3-5 adverse event (AE) deemed by the Investigator and confirmed by the data monitoring committee (DMC) to be attributed to Temferon during the DLT observation period of 30 days after Temferon administration and will indicate that the maximum tolerated dose has been reached in Part A and that dose escalation should not occur. The MTD will correspond to the highest dose at which 1 of the 3 patients in a dosing cohort experiences a DLT.

5.8 Administration of Temferon

Before initiation of the conditioning regimen, the Investigator will review the Temferon release specifications and will confirm [redacted] the intended infusion date. Following at least 3 hours and at most 12 hours from the non-manipulated HSPC infusion on Day 0, the patient will be infused IV with Temferon. Immediately before the infusion, all the other IV supportive drugs will be interrupted.

The entire infusion procedure is expected to last 10-15 minutes.

Vital signs (blood pressure, heart rate, temperature, and oxygen saturation) and symptoms will be monitored at Day 0 prior to infusion, every ten minutes during infusion and every hour, for 6 hours after the infusion. Vital signs and symptoms will be monitored thereafter every 6 hours until 24 hours post Temferon infusion.

5.9 Treatment and Study Compliance

No treatment compliance issue is expected in this study as eligible patients will receive a single intravenous infusion of Temferon and will be monitored by the study site personnel throughout the procedure.

Patient compliance with the subsequent study visits will be assessed by the study site personnel and documented through Day +720 (or early study termination).

5.10 Overdose

An overdose is any dose of study treatment given to or taken by a patient that exceeds the dose described in the protocol.

5.10.1 Reporting of Overdose

Any overdose, with or without associated AEs, must be promptly reported to the Sponsor and the Medical Monitor. Overdoses without signs or symptoms do not need to be recorded as AEs; in case of any AEs associated with the overdose, these should be reported on relevant AE/SAE sections in the CRF.

5.10.2 Treatment of Overdose

In the event of a suspected overdose, it is recommended that supportive clinical care be instituted as considered appropriate by the Investigator, based on the patient's clinical symptoms.

5.11 Prior, Concomitant and Subsequent Therapy

All medications licensed in Italy (http://www.codifa.it/) or available under AIFA monitoring /648 law are accepted if given for treatment of AE (any grade). All supportive therapy listed in Section 5.11.1 are accepted.

Use of all concomitant medications will be recorded in the patient's CRF. The minimum requirement is that drug name and the dates of administration are to be recorded. This will include all prescription drugs, herbal products, vitamins, minerals, and over the counter medications. Any changes in concomitant medications will also be recorded in the patient's CRF.

Any concomitant medication deemed necessary for the welfare of the patient during the study may be given at the discretion of the Investigator. However, it is the responsibility of the Investigator to ensure that details regarding the medication are recorded in full in the CRF.

5.11.1 Supportive therapy

During the hospitalization for administration of Temferon and monitoring for hematological recovery, the patients will be cared for in an isolation unit. The planned supportive therapies during hospitalization are:

- Isolation unit;
- Oral decontamination and mouth care according to local standards;
- Antibacterial, antiviral, antifungal including anti Pneumocystis jirovecii prophylaxis according to local policy;
- Intravenous substitution of immunoglobulins according to local policy;
- Hyper-hydration during the conditioning regimen;
- Daily complete blood count (and results recorded in the eCRF). Erythrocyte and platelet concentrates will be transfused according to clinical need; the investigator should consider infusion of platelet concentrates should blood platelet concentration be

DocuSign Envelope ID: A0702825-E0BD-46DB-A7D6-FEF901E11877

less than 50 x 10^3/mm^3. All blood products will be leukocyte depleted and irradiated. All efforts will be made to provide HLA-matched platelets in immunized patients with documented anti-HLA antibodies.

- Antiemetic prophylaxis and pain therapy if necessary.
- CMV (PCR), EBV (PCR) viral load and aspergillus antigenemia will be determined weekly till Day +90. These data should be recorded in the eCRF. If repeatedly negative, after Day +90, the interval of testing could be prolonged. Pre-emptive therapy with Ganciclovir or Foscarnet will be administered, according to local standards, if CMV positive; pre-emptive therapy with Rituximab will be administered to EBV-positive patients after Temferon infusion in case of repeated increase of EBV viral load, according to local standards;
- Lenograstim will be considered in case of absolute neutrophil counts <1000 cells/µL, at Day +21, following bone marrow evaluation or even earlier if clinically needed;
- Parenteral nutrition will be introduced in case of severe loss of body weight or clinical decision.
- Local policy for antimicrobial treatment will be followed in case of fever or infection.
- All medications (including for prophylaxis) and supportive therapies administered during the hospitalization should be recorded in the CRF.

5.11.2 Back-up stem cell infusion and rescue transplant

In the event of failure to engraft or poor graft function, all patients will have retained a non-manipulated, autologous back-up of at least 2.0 x 10^6 $CD34^+$ cells/kg, which may be re-infused in the form of a boost or as an autologous stem cell transplant.

In the event that patients have severe/persisting side effects not satisfactorily managed by standard of care therapy, patients may undergo an autologous stem cell transplantation (ablating the genetically engineered graft with high dose melphalan or another suitable conditioning regimen) as a rescue strategy.

6 Study Assessments and Procedures

Before performing any study procedures, all potential patients will sign the ICF. Patients will have the opportunity to have any questions answered before signing the ICF. The investigator must address all questions raised by the patient. The investigator will also sign the ICF.

6.1 Schedule of Events

The Schedule of Events (SOE) to be conducted during the study is provided in Section 12.1-Appendix 1, in Table 12-1, Table 12-2 and Table 12-3 for patients recruited and managed at OSR/INCB and in Table 12-4 and Table 12-5 for patients recruited and managed at PG. The SOE lists all of the assessments and indicates with an “X” or text the visits when they are to be made. All data obtained from these assessments must be verifiable in the patient’s source documentation and are to be documented in the eCRF.

Detailed descriptions of the efficacy, safety, and exploratory procedures to be conducted during this study are provided in the following sections. Additional details, including administrative information, regarding the efficacy, safety, and exploratory procedures, will be provided in the SOM.

6.2 Assessments and Procedures

6.2.1 Eligibility Assessments

6.2.1.1 Demographic data

Demographic data are to be collected on patient’s characteristics at screening including year of birth, gender and childbearing potential.

6.2.1.2 Medical history

Relevant past medical history including date of surgery and GBM resection, date of confirmation of unmethylated MGMT promoter status, assessments of any current medical conditions must be documented in the eCRF at Screening.

DocuSign Envelope ID: A0702825-E0BD-46DB-A7D6-FEF901E11877

6.2.2 Safety Assessments

6.2.2.1 Hematologic recovery by Day +30 following Temferon infusion

Hematologic recovery is defined by the absence of grade 3/4 neutropenia or grade 3/4 thrombocytopenia, as assessed by CTCAE v5.0, or recovery of pre-treatment blood counts. Complete blood counts will be performed daily after Temferon infusion until hematological engraftment and all these data will be recorded in the eCRF. The occurrence of hematological engraftment is defined as the first of at least 3 consecutive days with a neutrophil count > 0.5 x 10^9/L and platelet count > 20 x 10^9/L (in the absence of transfusions).

6.2.2.2 Tolerability of Temferon

Short-term tolerability (0-24 hrs from Temferon infusion) will be evaluated by AE reporting (any grade) and monitoring of the systemic reactions to cell injection (i.e. fever, tachycardia, nausea and vomiting, joint pain, skin rash). The tolerability and safety of Temferon over the first 90 days following Temferon administration will be evaluated by routine clinical and laboratory surveillance (see Section 6.2.2.11, and Section 6.6), assessment of autoimmune manifestations (see Section see Section 6.2.2.11, and Section 6.2.5.5) and reporting of adverse events (see Section 6.3).

6.2.2.3 Absence of Replication Competent Lentivirus (RCL)

This will consist in the absence of positive results at ELISA for HIV p24 antigen (serum). In case of positivity, second level testing may include a) DNA PCR for VSV-G envelope (cells), b) RT-PCR for HIV-pol RNA (serum) and c) in vivo amplification in suitable animal models. The tests will be performed at Screening, Day +90, +360 and +720. The presence of anti-HIV p24 antibodies will be evaluated as part of the RCL evaluation as well as evidence of immunization.

6.2.2.4 Absence of hematologic malignancy

Clinical examination will be performed to exclude lymphadenopathy and splenomegaly, and complete blood counts, bone marrow evaluations and chest x-ray will be performed according to the SOE and as necessary based on clinical judgment to exclude myelodysplasia, acute

DocuSign Envelope ID: A0702825-E0BD-46DB-A7D6-FEF901E11877

myeloid leukemia, lymphomas and other hematologic malignancies through to Day +720 post-Temferon infusion.

6.2.2.5 Absence of abnormal clonal proliferation

First level screening (every 6 months):

Clinical evaluation, blood count with differential to detect abnormal proliferation or cytopenia, routine biochemical tests to detect abnormal organ functions, flow cytometry on peripheral blood for lymphocyte subpopulations (see Section 6.2.5.8) and qPCR analysis for measuring vector copy number.

Second level screening (every year):

Bone marrow aspirates will be performed, including morphological examination, cytogenetic analysis, VCN and detailed immune phenotyping [Granulocytes, Monocytes ($CD14^+$), Lymphocytes, $MCD2^+$ (T cells), $MCD19^+$ (B cells), $MCD27^+$, $MCD45^+$, $MCD38^+$, $MCD117^+$, $MCD56^+$, $MCD14^+$, $MCD34^+$]. Pellets and viable cells of BM and/or PB will be archived. Additional molecular tests will be performed if above tests suggest monoclonal expansion (to be performed on recent and archived samples) to study clonality and identify pro-viral integrant as well as all other appropriate imaging and laboratory tests.

Additional tests and procedures may be performed on fresh or archived samples, as appropriate, in case of abnormal findings:

1. Mutational profiling of cryopreserved BM and/or PB cells;
2. B or T cell clonality tests using flow cytometry with monoclonal antibodies for TCR Vbeta families, or molecular assays assessing B-cell Receptor (BCR) or T-cell Receptor (TCR) repertoires;
3. Establishment of cell lines;
4. RT-PCR for expression of vector and genes next to the integration sites;
5. All other appropriate imaging and laboratory tests.

6.2.2.6 Safety of the myeloid-targeted IFN-α2 transgene product

This will be evaluated from hematologic engraftment to Day +720 by clinical and laboratory surveillance, including demonstration of 1) stable blood counts and absence of cytopenias > grade 2, not related to concomitant medications; 2) absence of autoimmune manifestations requiring treatment; 3) absence of organ toxicity (> grade 2 according to CTCAE v5.0) that can be ascribed to IFN exposure; 4) absence of systemic adverse reactions (e.g. flu-like symptoms) associated with IFN-α treatment.

6.2.2.7 Bone marrow evaluation

Patients will be subject to bone marrow aspirates at Screening, Day +30, +90, +180, +360 and +720 to evaluate bone marrow dysplasia and other abnormalities (at Screening), BM VCN and the presence of an IFN-α2 gene signature in the bone marrow (BM) of Temferon-treated patients and to monitor potential Temferon hematologic toxicities.

6.2.2.8 Cerebrospinal fluid (CSF) evaluation

Patients will be subject to lumbar puncture to evaluate the presence of an IFN-α2 gene signature, cytokines and VCN in the CSF of Temferon-treated patients and to monitor potential Temferon neurological toxicities. Routine laboratory assessment of the CSF will also occur (glucose, total protein, albumin (and serum albumin), ratio albumin CSF: albumin serum, IgG CSF, IgG CSF Index (LINK), CSF cell count and cytology). Lumbar puncture will occur at Screening, Day -6, +30, +90, +180, +360 and +720. In the event that a patient, who has received Temferon, undergoes a second craniotomy for resection of GBM, then CSF will be obtained directly via the transcranial route if surgically appropriate, or by lumbar puncture immediately prior to surgery.

6.2.2.9 Pregnancy

All women of childbearing potential must undergo a serum beta-hCG test at Screening, Day -6 (pre-conditioning), Day +30, +60, +90, +120, and at all scheduled visits thereafter.

In case of pregnancy, this must be reported to the Sponsor via the Pregnancy Form within 24 hours of the Investigator becoming aware of this to the Sponsor.

DocuSign Envelope ID: A0702825-E0BD-46DB-A7D6-FEF901E11877

6.2.2.10 Vital Signs

Data on the patient's vital signs must be recorded in the CRF at all visits. Vital signs are to be obtained after the patient has rested in the sitting position for at least 5 minutes. Vital signs will include body temperature, heart rate, respiratory rate, normal oxygen saturation (SO2) and systolic/diastolic blood pressure.

6.2.2.11 Physical & Neurological Examination

The physical exam is to evaluate the major body systems in order to identify any clinically significant disease or abnormality that, in the opinion of the Investigator, would exclude a patient from the study, preclude further study participation, or be evidence of an adverse event. It includes measurement of height (cm) and body weight (kg). Height is measured at screening and at Day -6; weight is to be assessed at all scheduled visits. It also includes examination of the head, eyes, ears, nose, and throat, skin, and the endocrine, metabolic, respiratory, cardiovascular, gastrointestinal, and musculoskeletal systems. A neurological examination should also be performed at Screening, Day -6, 0, during in-patient monitoring, Day +30 and all subsequent visits. The examination where possible should include the neurologic assessment in Neuro-Oncology (NANO) scale[243].

6.2.2.12 Diagnostic imaging and instrumental tests

These include 12-lead ECG, echocardiography, lung function tests including diffusion capacity, and chest x-ray. Lung function tests and chest x-ray will be performed at Day -6 whereas ECG and echocardiography will be performed at Screening and at Day -6. These tests will be performed periodically thereafter to evaluate exclusion criteria and potential Temferon toxicities. Any significant abnormalities identified at Screening will be recorded as part of medical history. Clinically relevant new findings during the study will be recorded as adverse events.

6.2.2.13 Slit lamp examination

A slit lamp examination performed by an ophthalmologist will take place at Screening, Day -6, +90, +180, +360, +540 and +720 post Temferon infusion to monitor potential ocular toxicity of IFN-α. Both anterior and posterior chambers will be examined, as will the retina. Any

DocuSign Envelope ID: A0702825-E0BD-46DB-A7D6-FEF901E11877

significant abnormalities identified at Screening will be recorded as part of medical history. Clinically relevant new findings during the study will be recorded as adverse events.

6.2.2.14 Psychiatric assessment.

This specialty evaluation will take place at Screening, Day -6, +90, +180, +360, +540 and +720 post Temferon infusion to monitor psychiatric toxicities associated with IFN-α, including depression and suicidal ideation. Clinically relevant findings at Screening will be recorded as part of the medical history. Clinically relevant new findings during the study will be recorded as adverse events.

6.2.3 Efficacy Assessments

6.2.3.1 Presence of transduced myeloid cells in the bone marrow and peripheral blood

The presence of Temferon cells will be determined through the evaluation of the engraftment of genetically modified cells in the bone marrow and their presence in the peripheral blood.

- Engraftment will be assessed by vector-specific qPCR on bone marrow myeloid cells at Day +30, +90, +180, +360 and +720 after infusion, and will be performed at an approved laboratory for Phase 1 primary and secondary endpoint analysis at OSR.
- The presence of transduced myeloid cells in PB will be assessed by vector-specific qPCR on PB myeloid cells at Day -6, hematological engraftment, Day +30, +90, +180, +360, +720.
- The presence of transduced myeloid cells in PB may also be assessed, according to the Investigator's clinical judgement, at the time of a Serious Adverse Event.

6.2.3.2 Clinical Response Assessments

Clinical response as defined as patients achieving complete response (CR), partial response (PR), stable disease (SD) and progressive disease (PD) will be evaluated in this study. Clinical response will be determined using clinical status, corticosteroid dose changes and imaging data from a standard brain MRI protocol[244] which will include the following components:

DocuSign Envelope ID: A0702825-E0BD-46DB-A7D6-FEF901E11877

- A precontrast, 3-dimensional, isotropic, inversion recovery prepped T1-weighted gradient echo (IR-GRE) sequence;
- An axial, 2-dimensional T2-weighted fluid-attenuated inversion recovery (FLAIR) sequence obtained using a turbo-spin-echo (TSE) readout;
- An axial, 2-dimensional, 3-directional (isotropic) diffusion-weighted imaging (DWI) sequence obtained using echoplanar (EPI) or radial acquisition;
- An axial, 2-dimensional T2-weighted TSE sequence (dual echo preferred, but not required);
- A postcontrast, 3-dimensional, isotropic, T1-weighted IR-GRE sequence with matching acquisition parameters to precontrast T1-weighted images.

Additional advanced imaging sequences will be incorporated to assess brain and tumor perfusion and spectroscopy.

Brain MRI using a 3 Tesla MRI machine will be performed at Screening, Day -6, +30, +90, +120 and every 60 days up to Day +720. In the event that disease progression is suspected based on the clinical status of the patient, brain MRI will be repeated.

All brain MRI images will be reviewed and reported by up to 2 expert readers and the report will then be discussed with the oncologist. Up to 5 target lesions will be selected for quantitative evaluation and non-target lesions will be qualitatively assessed. At each subsequent visit, new lesions will be identified. Target lesion response (Complete response (CR), Partial response (PR), Stable disease (SD), Progressive disease (PD) and unable to assess (UA) will be determined and if necessary reassessed over the next 12 weeks. Non-target lesions will also be assessed for CR, PD, SD, PD and UA. Potential pseudoresponse and pseudoprogression will also be reported with further follow up and additional imaging (e.g. MR perfusion or spectroscopy) considered to enable the clinical response to be determined. Among patients who demonstrate imaging findings meeting RANO criteria for PD within six months of Temferon administration including the development of new lesions, but who are not experiencing significant neurologic decline, confirmation of radiographic progression via follow-up imaging should be sought no sooner than three months after initial radiographic evidence of PD is detected[242].

DocuSign Envelope ID: A0702825-E0BD-46DB-A7D6-FEF901E11877

The Investigator (oncologist) will review the imaging data with the expert reader, and together with data regarding the clinical/neurological status of the patient and changes in the dose of corticosteroids, will determine the clinical response of the patient using iRANO criteria[242].

PFS and OS will be evaluated regularly up to Day +720 months post-Temferon infusion. PFS is defined as time from Temferon infusion until PD or death from any cause. OS is defined as time from Temferon infusion until death of any cause.

For PFS, OS, and response duration, observation times for patients in whom the event of interest was not observed will be censored at the time last seen alive without the respective event.

6.2.3.3 Functional status

Performance status (both Eastern Cooperative Oncology Group (ECOG) and Karnofsky scores) will be assessed at Screening, Day -6, 0, during in-patient monitoring, Day +30, and at all subsequent visits, and will be recorded in the CRF. The criteria for both performance scores are listed in the SOM.

6.2.3.4 Health Related Quality of Life

Quality of life will be assessed in this study using the European Organization for Research and Treatment of Cancer (EORTC) QLQ-C30 and QLQ-BN20 questionnaires. These questionnaires are self-administered. It is important that the Investigator does not influence the patient in any way. The EORTC QLQ-C30 measures five functional scales and global quality of life. The EORTC QLQ-BN20 consists of 20 items that assess future uncertainty, visual disorder, motor dysfunction, and communication deficit. Items are presented as questions on a scale ranging from 1 = “not at all” to 4 = “very much.”. The questionnaires should be completed by the patient at Screening, Day -6, +30, and at all subsequent visits, and the scores will be recorded in the CRF. The questionnaires are included within the SOE.

6.2.4 Exploratory Assessments

Based on the results for the primary and secondary endpoints, the following evaluations may be performed in order to further characterize Temferon’s mechanism of action in humans and

DocuSign Envelope ID: A0702825-E0BD-46DB-A7D6-FEF901E11877

to correlate it with the clinical outcome. Patient samples will be collected and stored according to the requirements for the proposed exploratory endpoints.

6.2.4.1 Evaluation of tumor tissue

As part of the informed consent procedure, patients will agree to exploratory analyses on tumor samples that are routinely preserved following from prior surgery. A range of exploratory analyses may be performed on these stored samples.

These will include tumor infiltrating lymphocytes content and T cell immunorepertoire (by sequencing), characterization of infiltrating myeloid cell populations including the presence of transduced TEMs (immunohistochemistry, flow cytometry and RNA scope), and the analysis of IFN-α-stimulated genes (e.g. Ifn2a, Irf7, Oas1a, Ifit1).

Immunohistochemistry will also include proliferative and apoptotic index (e.g. Ki-67, Cleaved Caspase 3, Tunel assay), type of vascularization (e.g. CD31 or vWF), EGFR and EGFRvIII expression, and evaluation of T cells infiltrate.

Genetic characterization of the tumor will also occur including the evaluation of presence/absence of IFN-α gene (DNA Fish), mutation/deletion of PTEN, P53, IDH1/2, etc. (RNA or DNA whole-exome sequencing).

In the event of confirmed progressive disease, should patients be suitable for second surgery or for tumor biopsy, tumor tissue will be analyzed for the same parameters as at screening, but in addition methylation of the MGMT promoter will be re-evaluated.

If patients have confirmed progressive disease but are not suitable for surgery or biopsy, the Investigator will approach the patient to obtain written informed consent for a post-mortem brain sample to be acquired for exploratory analyses.

6.2.4.2 Evaluation of CSF

The CSF will be evaluated at Screening, Day -6, +30, +90, +180, +360 and +720 to assess the IFN-stimulated gene signatures over time (including Ifn2a, Irf7, Oas1a, Ifit1) and to determine the concentration of IFN-α2 (ELISA). In the event that a patient, who has received Temferon, undergoes a second craniotomy for resection of GBM, then CSF will be obtained directly via the transcranial route if surgically appropriate, or by lumbar puncture immediately prior to

DocuSign Envelope ID: A0702825-E0BD-46DB-A7D6-FEF901E11877

surgery. Furthermore, if a suitable number of cells is detected in the CSF, the presence of transduced cells will be evaluated using VCN.

Moreover, the concentration of cytokines in CSF including IL-10, etc., may also be evaluated over time.

Following collection, routine evaluation of a 1ml aliquot of CSF will occur including glucose, total protein, albumin (and serum albumin), ratio albumin CSF: albumin serum, IgG CSF, IgG CSF Index (LINK), CSF cell Count and cytology.

CSF samples will be frozen and stored for future post-hoc analyses including the possibility of RNA or DNA whole exosome sequencing, transcriptomics, proteomics and metabolomics, for future biomarker development.

6.2.4.3 Evaluation of peripheral blood

Exploratory analyses on peripheral blood will include an evaluation of the T cell immunorepertoire over time in response to standard of care (surgery and brain irradiation) and Temferon. This will be evaluated in the context of changes in the general immune composition of PB over time.

Changes in IFN-α2 concentration over time will be evaluated in PB. IFN-α2 concentration in PB may also be evaluated at the time of Serious Adverse Events, according to the Investigator's clinical judgement. Serum samples will be frozen and stored for future post-hoc analyses including the possibility of exosome sequencing, transcriptomics, proteomics and metabolomics, for future biomarker development.

An IFN-induced gene signature comprising OAS1, IRF7 and IFIT1 will be measured on PB in CD14+ and CD15+ cells by quantitative RT-PCR at the Screening visit (negative control) and at Days +30, +90, +180, +360 and +720 post Temferon infusion.

Peripheral blood for exploratory analyses will be collected at Screening, Day -6, +30, +60, +90, +120, +180, +240, +360, and at every scheduled visit thereafter including Day +720.

DocuSign Envelope ID: A0702825-E0BD-46DB-A7D6-FEF901E11877

6.2.4.4 Evaluation of bone marrow

Temferon signature will be estimated through evaluation of transgene expression and the presence of an interferon-induced gene signature:

- IFN-α2 transgene expression will be evaluated on bone marrow enriched for myeloid cells by RT-qPCR at the Screening visit (negative control) and at Days +30 and +90. This assay may also be performed at Days +180, +360 and +720 post Temferon infusion if engraftment of gene-modified cells is documented by VCN and/or IFN signature.

- An IFN-induced gene signature comprising OAS1, IRF7 and IFIT1 will be measured on BM mononuclear cells by quantitative RT-PCR at the Screening visit (negative control) and at Days +30, +90, +180, +360 and +720 post Temferon infusion. Additional evaluation of IFN-induced gene signatures on T cells (CD3) and myeloid cells ($CD33^+$) may also be performed at these timepoints dependent on the results of the analysis of BM mononuclear cells.

6.2.4.5 Clonal tracking of Temferon graft over time in different hematopoietic lineages

Integration site analysis on distinct hematopoietic lineages will be performed on peripheral blood and/or bone marrow at Screening (negative control), Day +30, +90, +180, +360 and +720, and yearly thereafter as part of the separate long-term follow-up study, as long as gene-modified cells can be detected by VCN analysis.

6.2.5 Clinical Laboratory Assessments

Clinical laboratory parameters to be measured are listed below. Analyses will be performed at an approved Phase 1 laboratory, as specified in the SOM. Laboratory results should be assessed by the Investigator as normal, abnormal and clinically significant, or abnormal and clinically insignificant.

The procedures for collection and shipment of laboratory samples are described in the SOM.

Clinical laboratory assessments should be performed as specified in the SOE (see Table 12-1 to Table 12-5)

DocuSign Envelope ID: A0702825-E0BD-46DB-A7D6-FEF901E11877

6.2.5.1 Hematology

This will include white blood count (including full differential), neutrophils, hemoglobin, red cell count, hematocrit, mean cell volume, mean cell hemoglobin, mean cell hemoglobin concentration and platelets.

6.2.5.2 Clinical chemistry

This will include plasma creatinine, estimated glomerular filtration rate (calculated with Cockroft-Gault formula), blood urea nitrogen, total and fractionated bilirubin, alanine-amino transferase (ALT), aspartate amino transferase (AST), gamma glutamyl transferase (GGT), lactate dehydrogenase (LDH), alkaline phosphatase (ALP), phosphate, albumin, creatine kinase, total protein, uric acid, calcium, glucose, sodium, potassium, magnesium, C reactive protein (CRP). Urine will also be examined for pH, protein, blood, glucose and sediment analysis.

ProBNP and troponin T will be evaluated in plasma at Day -6, and periodically after Temferon administration.

6.2.5.3 Endocrine and metabolic

Plasma will be evaluated for thyroid stimulating hormone (TSH), fT3, fT4, ACTH, cortisol, adrenocorticotropic hormone (ACTH), estradiol (female), testosterone (male), triglycerides, cholesterol (total, LDL fraction, HDL fraction).

6.2.5.4 Coagulation tests

Blood will be evaluated for prothrombin time (PT), activated partial thromboplastin time (APTT), fibrinogen, and fibrinogen degradation product (XDP).

6.2.5.5 Autoimmune screening

Serum will be used to measure anti-nucleus antibodies (ANA), (Extractable Nuclear Antigen (ENA) to be done only if ANA positive), anti-neutrophil cytoplasm antibodies (ANCA), anti-thyroglobulin (TG) and anti-thyroperoxidase (TPO) autoantibodies, Rheumatoid Factor (RF),

DocuSign Envelope ID: A0702825-E0BD-46DB-A7D6-FEF901E11877

and anti-cyclic citrullinated peptide (CCP) auto-antibodies. Additional auto-antibodies may be requested by the Investigator according to clinical symptoms and examination findings.

6.2.5.6 Serology Panel

Routine antibody assessments on serum will occur for HCV, HBV, HIV 1-2, and HBsAg.

6.2.5.7 Microbiological evaluation

Microbiological evaluation will include microscopy, culture and sensitivity evaluations for bacteria and fungi from nasal and pharyngeal swabs, stool culture for bacteria and parasites including yersinia, urine culture, blood cultures and anal swab (screening for multi-resistant germs). CMV and EBV DNA will be assessed by molecular tests on plasma. Aspergillus antigen will also be measured in the serum. The treponema pallidum hemagglutination assay will be evaluated at Screening. Nucleic acid tests for HBV, HCV and HIV will be performed prior to stem cell mobilization and leukapheresis. The presence of mycobacterium tuberculosis will be determined using the Quantiferon assay at Day -6. During the COVID-19 pandemic, the presence of the SARS-COV-2 virus will also be determined periodically according to local hospital policies.

6.2.5.8 Immunology evaluation

Serum antibodies to pathogens (EBV, CMV, HSV1-2, VZV, Toxoplasma, Mycoplasma) will be measured. Immunophenotyping will occur in peripheral blood with leukocyte subpopulations ($CD3^+$, $CD3^+CD4^+$, $CD3^+CD8^+$, Total $CD19^+$, CD3-[$CD16^+$]$^+$[$CD56^+$], CD3-($CD16^+$CD56-), CD3-(CD16-$CD56^+$), CD3-($CD16^+CD56^+$), $CD25^+$ bright, $CD25^+$ dim, $CD25^+$ bright/CD127dim-neg, $CD25^+$dim/$CD127^+$, % lymphocytes, % monocytes ($CD14^+$), and % granulocytes). For timing of evaluations see SOE (Table 12-1 to Table 12-5).

6.2.6 Emergency Assessments and Procedures during COVID-19 pandemic

The COVID-19 pandemic commenced in Italy in approximately February 2020 and regulatory guidance was issued by AIFA in March[245] and updated in April 2020[246] relating to the management of clinical trials during the pandemic. The Sponsor has incorporated this guidance and as part of a risk assessment, has assessed the potential benefits and risks of continuing the

DocuSign Envelope ID: A0702825-E0BD-46DB-A7D6-FEF901E11877

TEM-GBM_001 study and considered modifications to the follow up of patients participating in the study.

Hospitals participating in the TEM-GBM_001 study have clear policies and guidance in place which aim to protect both patients and staff against the transmission of SARS-CoV-2 virus. These local policies must be followed by hospital staff and changes to policies will be tracked by the Sponsor. Any change in local policies that may limit further recruitment or retention of patients in the study must be notified to the Sponsor's Chief Medical Officer immediately.

In addition, it is important that compliance with good clinical practice (GCP) is maintained and wherever possible risks to trial integrity is minimized. The TEM-GBM_001 study is open label in design and continued recording of data according to the protocol requirements is important. In-person visits are essential to ensure the safety of patients and integrity of the study is maintained. However, protocol deviations are likely to occur due either to local restrictions mandated by government or institutional requirements or by decisions made by the patient due to the need to self-isolate. All deviations arising as a result of the COVID-19 pandemic (with reasons provided) should be recorded in source documentation for individual patients.

The Investigator (or delegate) should discuss with the Sponsor's Chief Medical Officer (or delegate) specific cases where alternative arrangements are anticipated as a result of the COVID-19 pandemic. These include:

- Delay in anticipated apheresis dates which will impact the manufacturing of Temferon (outside the permitted treatment window specified in the protocol).
- Delay in admission of the patient to the bone marrow transplant unit (outside the permitted treatment window specified in the protocol).
- Use of alternative public laboratory facilities for patient tests (i.e. facilities not currently specified in the clinical trial application).

The Sponsor's Chief Medical Officer (or delegate) will advise on whether the alternative arrangement should occur (and be recorded in the Sponsor's Trial Master File) or whether the patient should be withdrawn from the study.

DocuSign Envelope ID: A0702825-E0BD-46DB-A7D6-FEF901E11877

All efforts will be made to retain patients, who have received Temferon, in the clinical study and to follow the specified protocol schedule of events. In the event that a patient is unable or unwilling to attend the clinical unit for a protocol mandated visit, ideally video consultation will be permitted and all adverse events (ongoing and new) and concomitant medications will be recorded in the source document. If video is not available, then telephone consultation will be permitted. If at the next visit (scheduled or unscheduled) the patient attends the clinic, investigations and procedures that were omitted previously will be reviewed and after discussion with the Sponsor's Medical Monitor, a decision will be made to either perform or omit these procedures. The agreed decision will be recorded in the source document and Sponsor Trial Master File.

Any routine investigations that may be performed in public laboratories near the patient's home, should be discussed by the Investigator with the Sponsor's Medical Monitor prospectively and the agreed decision will be recorded in the source document and Sponsor Trial Master File.

During the period of the pandemic and in view of the importance of following government instructions on the restriction of movements of individuals, on-site monitoring will be suspended and increased remote monitoring will be performed.

6.3 Adverse Events

6.3.1 Definitions of Adverse Events

6.3.1.1 Definitions of Adverse Events

The Investigator is responsible for reporting all AEs that are observed or reported during the study, regardless of their relationship to Temferon or their clinical significance.

6.3.1.2 Adverse Event

An AE is defined as any untoward medical occurrence in a patient enrolled into this study regardless of its causal relationship to Temferon. Patients should be instructed by the Investigator or delegate to contact the Investigator at any time after entry into the study if any symptoms develop.

DocuSign Envelope ID: A0702825-E0BD-46DB-A7D6-FEF901E11877

6.3.1.3 Treatment-Emergent Adverse Event (TEAE)

A TEAE is defined as any event not present before exposure to Temferon or any event already present that worsens in either intensity or frequency after exposure to Temferon.

6.3.1.4 Serious Adverse Event (SAE)

A SAE is defined as any event that:

- results in death,
- is immediately life threatening,
- requires inpatient hospitalization or prolongation of existing hospitalization,
- results in persistent or significant disability/incapacity,
- or is a congenital anomaly/birth defect.

Important medical events that may not result in death, be life threatening, or require hospitalization may be considered SAEs when, based upon appropriate medical judgment, they may jeopardize the patient or may require medical or surgical intervention to prevent one of the outcomes listed in this definition.

6.3.2 Eliciting and Documenting Adverse Events

Adverse events will be assessed from the time the patient signs the informed consent form until exit from the study.

At every study visit, patients will be asked a standard nonleading question to elicit any medically related changes in their well-being. They will also be asked if they have been hospitalized, had any accidents, used any new medications, or changed concomitant medication regimens (both prescription and over the counter medications).

In addition to patient observations, AEs identified from any study data (e.g., laboratory values, physical examination findings, electrocardiogram [ECG] changes, lung function tests, etc.) or identified from review of other documents that are relevant to patient safety will be documented on the AE page in the CRF.

DocuSign Envelope ID: A0702825-E0BD-46DB-A7D6-FEF901E11877

6.3.3 Reporting Adverse Events

All AEs reported or observed during the study will be recorded on the AE page in the CRF. Information to be collected includes drug treatment, dose, event term, time of onset, investigator-specified assessment of severity and relationship to Temferon, time of resolution of the event, seriousness, any required treatment or evaluations, and outcome. Adverse events resulting from concurrent illnesses, reactions to concurrent illnesses, reactions to concurrent medications (including conditioning regimens), or progression of disease states must also be reported. All AEs will be followed to adequate resolution. The Medical Dictionary for Regulatory Activities (MedDRA) will be used to code all AEs.

Any medical condition that is present at the time that the patient is screened but does not deteriorate should not be reported as an AE. However, if it deteriorates at any time during the study, it should be recorded as an AE.

Any AE that meets SAE criteria (Section 6.3.1.4) must be reported to Arithmos (seQure Life Sciences) immediately (i.e., within 24 hours) after the time site personnel first learn about the event. The following contact information is to be used for SAE reporting:

Arithmos srl (business unit: **seQure Life Sciences)**
[redacted]

6.3.3.1 Assessment of Severity

The severity, or intensity, of an AE refers to the extent to which an AE affects the patient's daily activities. Severity will be rated according to CTCAE v 5.0 criteria. Should the AE not be listed under CTCAE criteria (see SOM for criteria), then the intensity of the AE will be rated as mild, moderate, or severe using the criteria described in Table 6-1.

DocuSign Envelope ID: A0702825-E0BD-46DB-A7D6-FEF901E11877

Table 6-1 Assessment of AE severity

Mild:	These events require minimal or no treatment and do not interfere with the patient's daily activities.<br>An event usually transient in nature and generally not interfering with normal activities.
Moderate:	These events result in a low level of inconvenience or concern with the therapeutic measures. Moderate events may cause some interference with normal functioning.<br>An AE that is sufficiently discomforting to interfere with normal activities.
Severe:	These events interrupt a patient's usual daily activity and may require systemic drug therapy or other treatment. Severe events are usually incapacitating.<br>An AE that is incapacitating and prevents normal activities.

Changes in the severity of an AE should be documented to allow an assessment of the duration of the event at each level of intensity to be performed. Adverse events characterized as intermittent do not require documentation of onset and duration of each episode.

6.3.3.2 Assessment of Causality

The investigator's assessment of an AE's relationship to Temferon is part of the documentation process, but it is not a factor in determining what is or is not reported in the study. If there is any doubt as to whether a clinical observation is an AE, the event should be reported.

The relationship or association of Temferon in causing or contributing to the AE will be characterized using the classification and criteria described in Table 6-2.

DocuSign Envelope ID: A0702825-E0BD-46DB-A7D6-FEF901E11877

Table 6-2 Assessment of AE causality

Unrelated:	This relationship suggests that there is no association between the ATMP and the reported event.
Possible:	This relationship suggests that treatment with the ATMP caused or contributed to the AE, i.e., the event follows a reasonable temporal sequence from the time of drug administration or follows a known response pattern to the ATMP, but could also have been produced by other factors.
Probable:	This relationship suggests that a reasonable temporal sequence of the event with drug administration exists and, based upon the known pharmacological action of the drug, known or previously reported adverse reactions to the drug or class of drugs, or judgment based on the Investigator's clinical experience, the association of the event with the ATMP seems likely. The event disappears or decreases on cessation or reduction of the dose of ATMP.
Definite:	This relationship suggests that a definite causal relationship exists between drug administration and the AE, and other conditions (concurrent illness, progression/expression of disease state, or concurrent medication reaction) do not appear to explain the event. The event reappears or worsens if the ATMP is re-administered.

In addition, the potential relationship of the AE to the harvesting of autologous HSPCs, conditioning chemotherapy, or other concomitant medication should be assessed by the Investigator with details provided in the eCRF.

6.3.3.3 Exceptions

Any AE that fulfils the serious criteria but reflects progression of disease should be recorded as disease progression and not listed as an SAE. For example, hospitalization for second surgery to resect GBM, should be listed under disease progression. Similarly, other events that are attributed to disease progression by the Investigator (e.g. seizures, progressive neurological symptoms or signs, etc.) should not be listed as SAEs.

DocuSign Envelope ID: A0702825-E0BD-46DB-A7D6-FEF901E11877

6.3.3.4 Follow-Up of Patients Reporting Adverse Events

All AEs must be reported in detail on the appropriate page in the CRF and followed to satisfactory resolution, until the investigator deems the event to be chronic or not clinically significant, or until the patient is considered to be stable.

6.4 Data Monitoring Committee (DMC)

An independent DMC constituted of a minimum of 3 members (2 hematologists and 1 neuro-oncologist) and up to 5 members (3 hematologists and 2 neuro-oncologists) with relevant medical and scientific expertise will be convened before the study start. A charter describing the composition and conduct of the DMC will be issued by the Sponsor and agreed to by all DMC members prior to the DMC's initial meeting. The names of the DMC members and the charter will be sent to the ethics committee before the first patient enters the study.

The DMC will review cumulative safety and efficacy data. The primary role of the committee is to ensure that emerging safety issues are identified and communicated to the Sponsor to ensure that the safety of patients is maintained. In addition, the DMC will review data and make a recommendation regarding the safety of progressing to higher doses and additional cohorts. At completion of Part A, the DMC will make a recommendation regarding the conditioning regimen, dose of non-modified $CD34^+$ supporter cells and dose of Temferon to take forward into Part B, if any.

The dose escalation recommendation and minutes from the open session of the DMC meeting will be sent to the ethics committee, after each DMC meeting. In addition, a summary of the data reviewed by the DMC at completion of Part A, will be sent to the ethics committee before commencing administration of Temferon in Part B.

6.5 Pregnancy

Pregnancy is not regarded as an AE. However, any pregnancy that occurs to a female patient or the partner of a male patient during study participation must be reported using a clinical study pregnancy form. To ensure patient safety, each pregnancy must be reported to Genenta Science within 2 weeks of learning of its occurrence. All pregnancies must be followed by a specialist expert in the assessment of teratogenicity. The pregnancy must be followed-up to determine outcome (including spontaneous miscarriage, elective termination, normal birth, or

DocuSign Envelope ID: A0702825-E0BD-46DB-A7D6-FEF901E11877

congenital abnormality) and status of mother and child, even if the patient was discontinued from the study. Pregnancy complications and elective terminations for medical reasons must be reported as an AE or SAE. Spontaneous miscarriages must be reported as an SAE.

Any SAE occurring in association with a pregnancy, brought to the investigator's attention after the patient has completed the study, and considered by the investigator as possibly related to the study treatment, must be promptly reported to Genenta Science.

6.6 Laboratory Analyses

Any abnormal laboratory test results (hematology, clinical chemistry, or urinalysis) or other safety assessments (e.g., ECGs, radiological scans, vital sign measurements), including those that worsen from baseline, felt to be clinically significant in the medical and scientific judgment of the investigator are to be recorded as AEs or SAEs.

However, any clinically significant safety assessments that are associated with progressive disease, unless judged by the investigator to be more severe than expected for the patient's condition, are **not** to be reported as AEs or SAEs.

6.7 Sample Collections

Samples from peripheral blood (serum, plasma, cell pellets, viable cells in DMSO, DNA), CSF and tumors will be collected, processed and stored at -20°C, -80°C or in liquid nitrogen vapor at OSR and INCB, according to Institutional guidelines.

Details are provided in the SOE.

DocuSign Envelope ID: A0702825-E0BD-46DB-A7D6-FEF901E11877

7 Statistical and Analytical Plan

The study is a Phase I/IIa safety and preliminary measure of efficacy study. As such, no formal statistical analysis is required. Descriptive and exploratory analyses will be performed on all data collected in this study to gain further insight into the efficacy and safety of this gene therapy strategy.

7.1 Sample Size Calculations

The choice of sample size is arbitrary but is conventional for gene therapy studies of this type and phase.

7.2 Analysis Sets

The full analysis set (FAS) will consist of all patients who receive Temferon. The FAS will be used for presenting demographics, baseline characteristics, efficacy, the overall safety and tolerability assessments.

7.3 Description of Subgroups to be Analyzed

No subgroup analyses are planned.

7.4 Statistical Analysis Methodology

There is to be no formal statistical analysis performed. All safety and efficacy variables are to be summarized descriptively.

Continuous variables will be summarized using the mean, the standard deviation, median, minimum value, and maximum value. Categorical variables will be summarized using frequency counts and percentages. Data will be listed in data listings.

Details of the software, data summaries, methods, and data conventions are described in the study statistical analysis plan (SAP).

DocuSign Envelope ID: A0702825-E0BD-46DB-A7D6-FEF901E11877

7.4.1 Safety Analysis

AEs are to be coded using the MedDRA classification to give a preferred term (PT) and system organ class (SOC) for each event. AEs coded according to CTCAE criteria and severity will also be summarized.

Numbers and percentages of patients with AEs prior to Temferon administration and following Temferon treatment (treatment emergent adverse events) will be presented. All AEs will be listed. Tables of AEs will be presented by SOC and PT. These tables are to include overall totals for AEs within each organ class. The number of patients with an event in each classification of severity and relationship to treatment within each treatment group will be tabulated. SAEs and AEs leading to withdrawal from the study will be listed separately.

All safety parameters will be summarized by cohort (Part A) and dose of Temferon administered (Part A and B).

Adverse events attributed to conditioning will also be summarized by the type of conditioning regimen received.

Safety analyses will be presented over time from Screening up to Day 0 (prior to Temferon) and separately following administration of Temferon (treatment emergent adverse events), i.e. within first 30 days, up to Day +90, up to Day +360, and up to Day +720.

Adverse events resulting in an MTD being reached, will be listed.

7.4.2 Other Analyses

The proportion of patients will be summarized by cohort and Temferon dose, achieving hematologic recovery by Day +30 following BCNU and thiotepa, thiotepa and busulfan, or busulfan monotherapy administration from administration of non-manipulated autologous HSPC and Temferon. Hematologic recovery is defined as the first of at least 3 consecutive days with a neutrophil count > 0.5 x 10^9/L and platelet count > 20 x 10^9/L.

The presence of transduced myeloid cells in bone marrow and peripheral blood will be summarized by listing the VCN for individual patients over time and providing the mean, median, SD and range for cohorts (Part A) and by Temferon dose (Part A and B, if any).

The proportion of patients achieving persistent transduced myeloid cells in peripheral blood will be summarized. Persistence is defined as a VCN above the level of detection evident for at least 12 weeks.

The proportions of patients achieving complete response (CR), partial response (PR), stable disease (SD), and progressive disease (PD) over time following Temferon administration, will be summarized.

Overall survival at various time points up to Day +720 will be summarized.

The mean, median, SD and range for functional status over time using the ECOG and KPS will be summarized. Changes from Screening and Day -6 will be determined.

The mean, median, SD and range for EORTC QLQ C30 and BN20 scores over time will be summarized. Changes from Screening and Day -6 will be determined.

Summary tables and listings will be provided for demographics and medical history. Summary tables and listing, and changes over time will be provided for physical examination, vital signs, and safety laboratory analyses (hematology, chemistry, coagulation, immunophenotyping, microbiological and immunological examination).

All exploratory analyses will be summarized in the SAP.

7.4.3 Interim Analyses

An interim analysis will be performed once the final patient recruited in Part A of the study completes the D+30 visit.

7.5 Data Quality Assurance

The Sponsor will implement a quality assurance plan for the study which will include critical process and data identification, risk identification, risk evaluation, risk control, risk communication, risk review and risk reporting.

In the event of an assessment, audit, or inspection, the Investigator and institution must agree to grant the advisors, auditors and inspectors direct access to all relevant documents and to

DocuSign Envelope ID: A0702825-E0BD-46DB-A7D6-FEF901E11877

allocate their time and the time of their staff to discuss the conduct of the study, any findings or relevant issues and to implement any corrective and/or preventive actions.

7.6 Data Management

As part of the responsibilities assumed by participating in the study, the Investigator agrees to maintain adequate case histories for the patients treated as part of the research under this protocol. The Investigator agrees to maintain accurate CRFs and source documentation as part of the data collection. These source documents may include diary cards, laboratory reports, ECGs, etc.

Patient data are to be entered via an CRF into an electronic data capture (EDC) platform. Data provided from other sources will be received, reconciled, combined and transferred to the Sponsor at predetermined time points.

All CRF information is to be filled in. If an item is not available or is not applicable, this fact should be indicated.

Clinical data management will be performed in accordance with applicable Genenta Science standards and data cleaning procedures to ensure the integrity of the data, e.g., removing errors and inconsistencies in the data. Adverse events and concomitant medication terms will be coded using the MedDRA, and the World Health Organization (WHO) Drug Dictionary, respectively.

The CRFs (including queries and audit trails) will be sent at the end of the study to the Sponsor to be retained. Each Investigator is to receive a copy of their site-specific data in the same format to maintain as the Investigator copy.

DocuSign Envelope ID: A0702825-E0BD-46DB-A7D6-FEF901E11877

8 Ethics

8.1 Independent Ethics Committee (IEC)

Applicable local regulations and the ICH GCP guidelines require that favorable opinion be obtained from an IEC before participation of human patients in research studies in Italy. Before study onset, the protocol, informed consent, advertisements to be used for the recruitment of study patients, and any other written information regarding this study to be provided to the patient or the patient's legal guardian must be reviewed and approved by the IEC. Documentation of all IEC favorable opinions and of the IEC compliance with ICH GCP will be maintained by the site and will be available for review by the Sponsor or its designee.

All IEC positive opinion should be signed by the IEC chairman or designee and must identify the IEC name and address, the clinical protocol by title or protocol number or both, and the date a favorable opinion was granted.

The Investigator is responsible for providing written summaries of the progress and status of the study at intervals not exceeding 1 year or otherwise specified by the IEC. The Investigator must promptly supply the Sponsor or its designee, the IEC, and, where applicable, the institution, with written reports on any changes significantly affecting the conduct of the study or increasing the risk to patients.

The dose escalation recommendation and minutes from the open session of the DMC meeting will be sent to the ethics committee, after each DMC meeting. In addition, a summary of the data reviewed by the DMC at completion of Part A, will be sent to the ethics committee before commencement of Part B.

8.2 Ethical Conduct of the Study

The study will be performed in accordance with the ethical principles that have their origin in the Declaration of Helsinki, ICH E6(R2) GCP Guideline, and all applicable laws and regulations.

8.3 Patient Information and Consent

A study-specific IEC-approved ICF in compliance with applicable local regulations shall be obtained from each patient before entering the study or performing any unusual or non-routine

DocuSign Envelope ID: A0702825-E0BD-46DB-A7D6-FEF901E11877

procedure that involves risk to the patient. If the ICF is revised during the course of the study, all active participating patients must sign the revised ICF.

Before recruitment and enrolment, each prospective patient or his or her legal guardian will be given a full explanation of the study and be allowed to read the approved ICF. Once the investigator is assured that the patient/legal guardian understands the implications of participating in the study, the patient/legal guardian will be asked to give consent to participate in the study by signing the ICF.

The investigator shall retain the signed original ICF(s) and give a copy of the signed original form to the patient or legal guardian.

8.4 Minimizing risk

However important a study may be to prove or disprove the value of a treatment, participants may suffer injury as a result of inclusion in the study, even if the whole community benefits. Every effort is made to anticipate and reduce known hazards. Investigators will be fully aware before the start of a clinical study of all relevant preclinical and clinical toxicity of the drug product. In order to minimize risk in patients enrolled in clinical studies, all parties involved in the study will be properly trained and experienced in studying the patients' population, including the evaluation and management of potential adverse events.

In designing the study, every attempt has been made to minimize the number of participants and procedures, consistent with good study design. Mechanisms will be in place to ensure that a study can be rapidly terminated if an unexpected hazard is noted.

9 Investigator's Obligations

The following administrative items are meant to guide the investigator in the conduct of the study but may be patient to change based on industry and government standard operating procedures, working practice documents, or guidelines. Changes will be reported to the IEC but will not result in protocol amendments.

9.1 Confidentiality

All laboratory specimens, evaluation forms, reports, and other records will be identified in a manner designed to maintain patient confidentiality. All records will be kept in a secure storage area with limited access. Clinical information will not be released without the written permission of the patient (or the patient's legal guardian), except as necessary for monitoring and auditing by the Sponsor, its designee, the Italian regulatory authority, or the IEC.

The investigator and all employees and coworkers involved with this study may not disclose or use for any purpose other than performance of the study any data, record, or other unpublished, confidential information disclosed to those individuals for the purpose of the study. Prior written agreement from the Sponsor or its designee must be obtained for the disclosure of any said confidential information to other parties.

9.2 Financial Disclosure and Obligations

The Sponsor is not financially responsible for further testing or treatment of any medical condition that may be detected during the screening process. In addition, in the absence of specific arrangements, the Sponsor is not financially responsible for further treatment of the patient's disease.

9.3 Investigator Documentation

Prior to beginning the study, the investigator will be asked to comply with ICH E6(R2) by providing the following essential documents, including but not limited to:

- IEC favorable opinion
- Original Investigator-signed agreement page of the protocol
- Curriculum vitae (CV) for the Investigator and each sub-Investigator

DocuSign Envelope ID: A0702825-E0BD-46DB-A7D6-FEF901E11877

- IEC-approved informed consent, samples of site advertisements for recruitment for this study, and any other written information regarding this study that is to be provided to the patient or legal guardian, and
- Laboratory certifications and normal ranges for any local laboratories used by the site.

9.4 Study Conduct

The investigator agrees that the study will be conducted according to the principles of ICH E6(R2). The investigator will conduct all aspects of this study in accordance with all national, state, and local laws or regulations. Study information from this protocol will be posted on publicly available clinical trial registers before enrolment of patients begins.

9.5 Adherence to Protocol

The investigator agrees to conduct the study as outlined in this protocol in accordance with ICH E6(R2) and all applicable guidelines and regulations.

9.6 Adverse Events and Study Report Requirements

By participating in this study the investigator agrees to submit reports of SAEs according to the time line and method outlined in the protocol. In addition, the investigator agrees to submit annual reports to the study site IEC as appropriate.

9.7 Investigator's Final Report

Upon completion of the study, the investigator, where applicable, should inform the institution; the investigator/institution should provide the IEC with a summary of the study's outcome and the Sponsor and regulatory authority(ies) with any reports required.

9.8 Records Retention

Essential documents should be retained until at least 2 years after the last approval of a marketing application in an ICH region and until there are no pending or contemplated marketing applications in an ICH region or at least 2 years have elapsed since the formal discontinuation of clinical development of the investigational product. These documents

DocuSign Envelope ID: A0702825-E0BD-46DB-A7D6-FEF901E11877

should be retained for a longer period, however, if required by the applicable regulatory requirements or by an agreement with the Sponsor. It is the responsibility of the Sponsor to inform the investigator/institution as to when these documents no longer need to be retained.

9.9 Liability and Insurance

An insurance policy has been purchased by the Sponsor. It covers its legal liability for injuries caused to participating individuals and resulting from this study performed strictly in accordance with the clinical protocol as well as applicable laws and standard operating procedures. An abstract copy of the insurance policy and the insurance certificate will be archived in the Sponsor's Trial Master File (TMF).

9.10 Publications

After completion of the study, the data may be considered for reporting at a scientific meeting or for publication in a scientific journal. In these cases, the Sponsor will be responsible for these activities and will work with the investigators to determine how the manuscript is written and edited, the number and order of authors, the publication to which it will be submitted, and other related issues. The Sponsor has final approval authority over all such issues.

Data are the property of the Sponsor and cannot be published without prior authorization from the Sponsor.

DocuSign Envelope ID: A0702825-E0BD-46DB-A7D6-FEF901E11877

10 Study Management

10.1 Monitoring

The Sponsor will select a CRO or a qualified, experienced, independent contractor to perform monitoring services on behalf of the Sponsor. An agreement between the Sponsor and the 3rd party will be in place and located within the Trial Master File before the first patient is recruited into the study.

10.1.1 Clinical Study Monitor

The clinical monitor, as a representative of the Sponsor, has the obligation to follow the study closely. In doing so, the monitor will visit the Investigator and study site at periodic intervals, in addition to maintaining necessary telephone and letter contact. The monitor will maintain current personal knowledge of the study through observation, review of study records and source documentation, and discussion of the conduct of the study with the Investigator and personnel.

All aspects of the study will be carefully monitored, by the Sponsor or its designee, for compliance with applicable government regulation with respect to current GCP guideline and current standard operating procedures.

10.1.2 Inspection of Records

Investigators and institutions involved in the study will permit study-related monitoring, audits, IEC review, and regulatory inspections by providing direct access to all study records. In the event of an audit, the Investigator agrees to allow the Sponsor, representatives of the Sponsor, or a regulatory agency (e.g., AIFA, the Italian regulatory agency) access to all study records.

The Investigator should promptly notify the Sponsor of any audits scheduled by any regulatory authorities and promptly forward copies of any audit reports received to the Sponsor.

DocuSign Envelope ID: A0702825-E0BD-46DB-A7D6-FEF901E11877

10.2 Management of Protocol Amendments and Deviations

10.2.1 Modification of the Protocol

Any changes in this study, except those necessary to remove an apparent, immediate hazard to the patient, must be reviewed and approved by the Sponsor or its designee. Amendments to the protocol must be submitted in writing to the Investigator's IEC for favorable opinion before patients can be enrolled into an amended protocol.

10.2.2 Protocol Deviations

The Investigator or designee must document and explain in the patient's source documentation any deviation from the approved protocol. The Investigator may implement a deviation from, or a change of, the protocol to eliminate an immediate hazard to study patients without prior IEC and applicable regulatory agency approvals. As soon as possible after such an occurrence, the implemented deviation or change, the reasons for it, and any proposed protocol amendments should be submitted to the IEC for review and favorable opinion, to the Sponsor for agreement, and to the regulatory authorities, if required.

A deviation from the protocol is an unintended or unanticipated departure from the procedures or processes approved by the Sponsor and the IEC and agreed to by the Investigator. A significant deviation occurs when there is non-adherence to the protocol by the patient or Investigator that results in a significant, additional risk to the patient. Significant deviations can include non-adherence to inclusion or exclusion criteria, enrolment of the patient without prior Sponsor approval, or non-adherence to Italian regulations or ICH GCP guidelines, and will lead to the patient being withdrawn from the study (see Section 4.2).

Protocol deviations will be documented by the clinical monitor throughout the course of monitoring visits. Principal Investigators will be notified in writing by the monitor of deviations. The IEC should be notified of all protocol deviations in a timely manner.

10.3 Study Termination

Although the Sponsor has every intention of completing the study, the Sponsor reserves the right to discontinue the study at any time for clinical or administrative reasons.

DocuSign Envelope ID: A0702825-E0BD-46DB-A7D6-FEF901E11877

10.3.1 End of Study

The end of the study is defined as the date on which the last patient completes the last visit (includes follow-up visit).

10.3.2 Premature termination of the trial

The Sponsor and the Investigator are under obligation to monitor the progress of the study with regard to safety-relevant developments and, if necessary, initiate the premature termination of the entire study. The Sponsor/Investigator will be supported in this responsibility by the DMC, if necessary.

The study must be terminated prematurely if:

- the benefit-to-risk ratio for the patient changes markedly,
- the Sponsor/Investigator and the DMC consider that the termination of the trial is necessary,
- indications arise that the study patients' safety is no longer guaranteed,
- the questions addressed in the study can be clearly answered on the basis of results from another trial on the same patients,

If the clinical study is prematurely terminated or suspended for any reason, the Investigator should promptly inform the study patients and ensure their appropriate medical care and follow-up. Where required by the applicable regulatory requirements, the competent authority and the ethic committees will also be informed by the Sponsor.

10.4 Final Report

Whether the study is completed or prematurely terminated, the Sponsor will ensure that the clinical study reports are prepared and provided to the regulatory agency(ies) as required by the applicable regulatory requirement(s). The Sponsor will also ensure that the clinical study reports in marketing applications meet the standards of the ICH harmonised tripartite guideline (E3): Structure and content of clinical study reports.

Where required by applicable regulatory requirements, an Investigator signatory will be identified for the approval of the clinical study report. The Investigator will be provided

DocuSign Envelope ID: A0702825-E0BD-46DB-A7D6-FEF901E11877

reasonable access to statistical tables, figures, and relevant reports and will have the opportunity to review the complete study results.

Upon completion of the clinical study report, the Sponsor will provide the Investigator with the full summary of the study results. The Investigator is encouraged to share the summary results with the study patients, as appropriate. The study results will be posted on publicly available clinical trial registers.

DocuSign Envelope ID: A0702825-E0BD-46DB-A7D6-FEF901E11877

11 Reference List

1. CHMP. Strimvelis, Common name - autologous CD34+ enriched cell fraction that contains CD34+ cells transduced with retroviral vector that encodes for the human ADA cDNA sequence. http://www.ema.europa.eu/docs/en_GB/document_library/EPAR_-_Product_Information/human/003854/WC500208199.pdf.
2. Raffaele, I. S. Gene Therapy for Transfusion Dependent Beta-thalassemia (TIGET-BTHAL). *Clinicaltrials.gov* (2018).
3. Raffaele, I. S. Gene Therapy for Metachromatic Leukodystrophy (TIGET-MLD). *Clinicaltrials.gov* (2018).
4. Raffaele, I. S. Gene Therapy for Wiskott-Aldrich Syndrome (TIGET-WAS). *Clinicaltrials.gov* (2018).
5. White, M., Whittaker, R., Gándara, C. & Stoll, E. A. A Guide to Approaching Regulatory Considerations for Lentiviral-Mediated Gene Therapies. *Human Gene Therapy Methods* **28**, 163–176 (2017).
6. Burugu, S., Dancsok, A. R. & Nielsen, T. O. Emerging targets in cancer immunotherapy. *Seminars in Cancer Biology* (2017) doi:10.1016/j.semcancer.2017.10.001.
7. Khalil, D. N., Smith, E. L., Brentjens, R. J. & Wolchok, J. D. The future of cancer treatment: immunomodulation, CARs and combination immunotherapy. *Nature Reviews Clinical Oncology* **13**, 394–394 (2016).
8. Dempke, W. C. M., Fenchel, K., Uciechowski, P. & Dale, S. P. Second- and third-generation drugs for immuno-oncology treatment—The more the better? *European Journal of Cancer* **74**, 55–72 (2017).
9. De Palma, M. *et al.* Tumor-Targeted Interferon-α Delivery by Tie2-Expressing Monocytes Inhibits Tumor Growth and Metastasis. *Cancer Cell* **14**, 299–311 (2008).
10. Escobar, G. *et al.* Genetic Engineering of Hematopoiesis for Targeted IFN- Delivery Inhibits Breast Cancer Progression. *Science Translational Medicine* **6**, 217ra3-217ra3 (2014).
11. Catarinella, M. *et al.* IFNα gene/cell therapy curbs colorectal cancer colonization of the liver by acting on the hepatic microenvironment. *EMBO molecular medicine* **8**, 155–70 (2016).
12. Louis, D. N. *et al.* The 2016 World Health Organization Classification of Tumors of the Central Nervous System: a summary. *Acta neuropathologica* **131**, 803–20 (2016).
13. Ostrom, Q. T. *et al.* CBTRUS Statistical Report: Primary brain and other central nervous system tumors diagnosed in the United States in 2010-2014. *Neuro Oncol* **19**, v1–v88 (2017).
14. Malmström, A. *et al.* Temozolomide versus standard 6-week radiotherapy versus hypofractionated radiotherapy in patients older than 60 years with glioblastoma: the Nordic randomised, phase 3 trial. *The Lancet. Oncology* **13**, 916–26 (2012).

DocuSign Envelope ID: A0702825-E0BD-46DB-A7D6-FEF901E11877

15. Nava, F. *et al.* Survival effect of first- and second-line treatments for patients with primary glioblastoma: a cohort study from a prospective registry, 1997-2010. *Neuro-oncology* **16**, 719–27 (2014).
16. Olar, A. & Aldape, K. D. Using the molecular classification of glioblastoma to inform personalized treatment. *J Pathol* **232**, 165–177 (2014).
17. Ohgaki, H. & Kleihues, P. The definition of primary and secondary glioblastoma. *Clinical cancer research : an official journal of the American Association for Cancer Research* **19**, 764–72 (2013).
18. Phillips, H. S. *et al.* Molecular subclasses of high-grade glioma predict prognosis, delineate a pattern of disease progression, and resemble stages in neurogenesis. *Cancer cell* **9**, 157–73 (2006).
19. Verhaak, R. G. W. *et al.* Integrated Genomic Analysis Identifies Clinically Relevant Subtypes of Glioblastoma Characterized by Abnormalities in PDGFRA, IDH1, EGFR, and NF1. *Cancer Cell* **17**, 98–110 (2010).
20. Kleihues, P. & Ohgaki, H. Primary and secondary glioblastomas: From concept to clinical diagnosis. *Neuro-Oncology* **1**, 44–51 (1999).
21. Ohgaki, H. *et al.* Genetic pathways to glioblastoma: a population-based study. *Cancer research* **64**, 6892–9 (2004).
22. Crespo, I. *et al.* Molecular and Genomic Alterations in Glioblastoma Multiforme. *The American Journal of Pathology* **185**, 1820–1833 (2015).
23. Rong, Y., Durden, D. L., Van Meir, E. G. & Brat, D. J. 'Pseudopalisading' necrosis in glioblastoma: a familiar morphologic feature that links vascular pathology, hypoxia, and angiogenesis. *Journal of neuropathology and experimental neurology* **65**, 529–39 (2006).
24. Holland, E. C. Glioblastoma multiforme: the terminator. *Proceedings of the National Academy of Sciences of the United States of America* **97**, 6242–4 (2000).
25. Charles, N. A., Holland, E. C., Gilbertson, R., Glass, R. & Kettenmann, H. The brain tumor microenvironment. *Glia* **59**, 1169–80 (2011).
26. Hussain, S. F. *et al.* The role of human glioma-infiltrating microglia/macrophages in mediating antitumor immune responses. *Neuro-oncology* **8**, 261–79 (2006).
27. Parney, I. F., Waldron, J. S. & Parsa, A. T. Flow cytometry and in vitro analysis of human glioma-associated macrophages. Laboratory investigation. *Journal of neurosurgery* **110**, 572–82 (2009).
28. Du, R. *et al.* HIF1α Induces the Recruitment of Bone Marrow-Derived Vascular Modulatory Cells to Regulate Tumor Angiogenesis and Invasion. *Cancer Cell* **13**, 206–220 (2008).
29. Zhai, H., Heppner, F. L. & Tsirka, S. E. Microglia/macrophages promote glioma progression. *Glia* **59**, 472–85 (2011).
30. Markovic, D. S., Glass, R., Synowitz, M., Rooijen, N. van & Kettenmann, H. Microglia stimulate the invasiveness of glioma cells by increasing the activity of metalloprotease-2. *Journal of neuropathology and experimental neurology* **64**, 754–62 (2005).

DocuSign Envelope ID: A0702825-E0BD-46DB-A7D6-FEF901E11877

31. Wesolowska, A. *et al.* Microglia-derived TGF-beta as an important regulator of glioblastoma invasion--an inhibition of TGF-beta-dependent effects by shRNA against human TGF-beta type II receptor. *Oncogene* **27**, 918–30 (2008).
32. Rao, J. S. Molecular mechanisms of glioma invasiveness: the role of proteases. *Nature reviews. Cancer* **3**, 489–501 (2003).
33. Wagner, S. *et al.* Microglial/macrophage expression of interleukin 10 in human glioblastomas. *International journal of cancer* **82**, 12–6 (1999).
34. Li, W. & Graeber, M. B. The molecular profile of microglia under the influence of glioma. *Neuro-oncology* **14**, 958–78 (2012).
35. Mosser, D. M. The many faces of macrophage activation. *Journal of leukocyte biology* **73**, 209–12 (2003).
36. Gordon, S. Alternative activation of macrophages. *Nature reviews. Immunology* **3**, 23–35 (2003).
37. Mantovani, A., Germano, G., Marchesi, F., Locatelli, M. & Biswas, S. K. Cancer-promoting tumor-associated macrophages: new vistas and open questions. *European journal of immunology* **41**, 2522–5 (2011).
38. Tsutsui, S. *et al.* Macrophage infiltration and its prognostic implications in breast cancer: the relationship with VEGF expression and microvessel density. *Oncology reports* **14**, 425–31 (2005).
39. Shimura, S. *et al.* Reduced infiltration of tumor-associated macrophages in human prostate cancer: association with cancer progression. *Cancer research* **60**, 5857–61 (2000).
40. Lapa, C. *et al.* Tumor-associated macrophages in glioblastoma multiforme-a suitable target for somatostatin receptor-based imaging and therapy? *PloS one* **10**, e0122269 (2015).
41. Nishie, A. *et al.* Macrophage infiltration and heme oxygenase-1 expression correlate with angiogenesis in human gliomas. *Clin Cancer Res* **5**, 1107–1113 (1999).
42. Engler, J. R. *et al.* Increased microglia/macrophage gene expression in a subset of adult and pediatric astrocytomas. *PLoS One* **7**, e43339 (2012).
43. Komohara, Y. *et al.* Importance of direct macrophage-tumor cell interaction on progression of human glioma. *Cancer Sci* **103**, 2165–2172 (2012).
44. Gabrusiewicz, K. *et al.* Glioblastoma-infiltrated innate immune cells resemble M0 macrophage phenotype. *JCI Insight* **1**, (2016).
45. Stupp, R. *et al.* Radiotherapy plus concomitant and adjuvant temozolomide for glioblastoma. *The New England journal of medicine* **352**, 987–96 (2005).
46. Athanassiou, H. *et al.* Randomized Phase II Study of Temozolomide and Radiotherapy Compared With Radiotherapy Alone in Newly Diagnosed Glioblastoma Multiforme. *Journal of Clinical Oncology* **23**, 2372–2377 (2005).
47. Karacetin, D., Okten, B., Yalcin, B. & Incekara, O. Concomitant temozolomide and radiotherapy versus radiotherapy alone for treatment of newly diagnosed glioblastoma multiforme. *Journal of B.U.ON. : official journal of the Balkan Union of Oncology* **16**, 133–7.

DocuSign Envelope ID: A0702825-E0BD-46DB-A7D6-FEF901E11877

48. Kocher, M. *et al.* Randomized Study of Postoperative Radiotherapy and Simultaneous Temozolomide without Adjuvant Chemotherapy for Glioblastoma. *Strahlentherapie und Onkologie* **184**, 572–579 (2008).
49. Szczepanek, D. *et al.* Efficacy of concomitant and adjuvant temozolomide in glioblastoma treatment. A multicentre randomized study. *Neurologia i neurochirurgia polska* **47**, 101–8.
50. Feng, E., Sui, C., Wang, T. & Sun, G. Temozolomide with or without Radiotherapy in Patients with Newly Diagnosed Glioblastoma Multiforme: A Meta-Analysis. *European Neurology* **77**, 201–210 (2017).
51. Hegi, M. E. *et al.* Correlation of O ^{6} -Methylguanine Methyltransferase (MGMT) Promoter Methylation With Clinical Outcomes in Glioblastoma and Clinical Strategies to Modulate MGMT Activity. *Journal of Clinical Oncology* **26**, 4189–4199 (2008).
52. Gorlia, T. *et al.* Nomograms for predicting survival of patients with newly diagnosed glioblastoma: prognostic factor analysis of EORTC and NCIC trial 26981-22981/CE.3. *The Lancet. Oncology* **9**, 29–38 (2008).
53. Olson, R. A., Brastianos, P. K. & Palma, D. A. Prognostic and predictive value of epigenetic silencing of MGMT in patients with high grade gliomas: a systematic review and meta-analysis. *Journal of neuro-oncology* **105**, 325–35 (2011).
54. Wick, W. *et al.* Temozolomide chemotherapy alone versus radiotherapy alone for malignant astrocytoma in the elderly: the NOA-08 randomised, phase 3 trial. *The Lancet. Oncology* **13**, 707–15 (2012).
55. University of Pennsylvania. CART-EGFRvIII + Pembrolizumab in GBM - Full Text View - ClinicalTrials.gov. https://clinicaltrials.gov/ct2/show/NCT03726515?term=03726515&rank=1.
56. Washington University School of Medicine. Neoantigen-based Personalized Vaccine Combined With Immune Checkpoint Blockade Therapy in Patients With Newly Diagnosed, Unmethylated Glioblastoma - Full Text View - ClinicalTrials.gov. https://clinicaltrials.gov/ct2/show/NCT03422094?term=03422094&rank=1.
57. M.D. Anderson Cancer Center. EDO-S101 for MGMT Unmethylated Glioblastoma (nGBM) - Full Text View - ClinicalTrials.gov. https://clinicaltrials.gov/ct2/show/NCT03452930?term=03452930&rank=1.
58. New York University School of Medicine. Nivolumab, Ipilimumab, and Short-course Radiotherapy in Adults With Newly Diagnosed, MGMT Unmethylated Glioblastoma - Full Text View - ClinicalTrials.gov. https://clinicaltrials.gov/ct2/show/NCT03367715?term=03367715&rank=1.
59. BeiGene USA. A Study Assessing BGB-290 With Radiation and/or Temozolomide (TMZ) in Subjects With Newly Diagnosed or Recurrent Glioblastoma - Full Text View - ClinicalTrials.gov. https://clinicaltrials.gov/ct2/show/NCT03150862?term=03150862&rank=1.
60. BMS. An Investigational Immuno-therapy Study of Nivolumab Compared to Temozolomide, Each Given With Radiation Therapy, for Newly-diagnosed Patients With Glioblastoma (GBM, a Malignant Brain Cancer) - Full Text View -

DocuSign Envelope ID: A0702825-E0BD-46DB-A7D6-FEF901E11877

ClinicalTrials.gov. https://clinicaltrials.gov/ct2/show/NCT02617589?term=02617589&rank=1.

61. Jue, T. R. *et al.* Veliparib in combination with radiotherapy for the treatment of MGMT unmethylated glioblastoma. *Journal of translational medicine* **15**, 61 (2017).
62. Lee, A. *et al.* Patterns of care and outcomes of chemoradiation versus radiation alone for MGMT promoter unmethylated glioblastoma. *Clinical neurology and neurosurgery* **170**, 127–131 (2018).
63. Herrlinger, U. *et al.* Bevacizumab Plus Irinotecan Versus Temozolomide in Newly Diagnosed O6-Methylguanine-DNA Methyltransferase Nonmethylated Glioblastoma: The Randomized GLARIUS Trial. *Journal of clinical oncology : official journal of the American Society of Clinical Oncology* **34**, 1611–9 (2016).
64. Wick, W. *et al.* Phase II Study of Radiotherapy and Temsirolimus versus Radiochemotherapy with Temozolomide in Patients with Newly Diagnosed Glioblastoma without MGMT Promoter Hypermethylation (EORTC 26082). *Clinical cancer research : an official journal of the American Association for Cancer Research* **22**, 4797–4806 (2016).
65. de Visser, K. E., Eichten, A. & Coussens, L. M. Paradoxical roles of the immune system during cancer development. *Nature Reviews Cancer* **6**, 24–37 (2006).
66. Condeelis, J. & Pollard, J. W. Macrophages: Obligate Partners for Tumor Cell Migration, Invasion, and Metastasis. *Cell* **124**, 263–266 (2006).
67. De Palma, M., Murdoch, C., Venneri, M. A., Naldini, L. & Lewis, C. E. Tie2-expressing monocytes: regulation of tumor angiogenesis and therapeutic implications. *Trends in immunology* **28**, 519–24 (2007).
68. De Palma, M. & Naldini, L. Role of haematopoietic cells and endothelial progenitors in tumour angiogenesis. *Biochimica et biophysica acta* **1766**, 159–66 (2006).
69. Shojaei, F., Zhong, C., Wu, X., Yu, L. & Ferrara, N. Role of myeloid cells in tumor angiogenesis and growth. *Trends in cell biology* **18**, 372–8 (2008).
70. De Palma, M., Venneri, M. A., Roca, C. & Naldini, L. Targeting exogenous genes to tumor angiogenesis by transplantation of genetically modified hematopoietic stem cells. *Nature medicine* **9**, 789–95 (2003).
71. De Palma, M. *et al.* Tie2 identifies a hematopoietic lineage of proangiogenic monocytes required for tumor vessel formation and a mesenchymal population of pericyte progenitors. *Cancer cell* **8**, 211–26 (2005).
72. Ahn, G.-O. & Brown, J. M. Matrix Metalloproteinase-9 Is Required for Tumor Vasculogenesis but Not for Angiogenesis: Role of Bone Marrow-Derived Myelomonocytic Cells. *Cancer Cell* **13**, 193–205 (2008).
73. Mantovani, A. & Sica, A. Macrophages, innate immunity and cancer: balance, tolerance, and diversity. *Current Opinion in Immunology* **22**, 231–237 (2010).
74. Fantin, A. *et al.* Tissue macrophages act as cellular chaperones for vascular anastomosis downstream of VEGF-mediated endothelial tip cell induction. *Blood* **116**, 829–40 (2010).
75. Pucci, F. *et al.* A distinguishing gene signature shared by tumor-infiltrating Tie2-expressing monocytes, blood "resident" monocytes, and embryonic

DocuSign Envelope ID: A0702825-E0BD-46DB-A7D6-FEF901E11877

macrophages suggests common functions and developmental relationships. *Blood* **114**, 901–14 (2009).

76. Venneri, M. A. *et al.* Identification of proangiogenic TIE2-expressing monocytes (TEMs) in human peripheral blood and cancer. *Blood* **109**, 5276–5285 (2007).
77. Schauer, D. *et al.* Intermediate monocytes but not TIE2-expressing monocytes are a sensitive diagnostic indicator for colorectal cancer. *PloS one* **7**, e44450 (2012).
78. Atanasov, G. *et al.* Prognostic significance of TIE2-expressing monocytes in hilar cholangiocarcinoma. *Journal of Surgical Oncology* **114**, 91–98 (2016).
79. Fiorcari, S. *et al.* Ibrutinib modifies the function of monocyte/macrophage population in chronic lymphocytic leukemia. *Oncotarget* **7**, 65968–65981 (2016).
80. Cattin, S. *et al.* Bevacizumab specifically decreases elevated levels of circulating KIT+CD11b+ cells and IL-10 in metastatic breast cancer patients. *Oncotarget* **7**, 11137–50 (2016).
81. Campanelli, R. *et al.* Tie2 Expressing Monocytes in the Spleen of Patients with Primary Myelofibrosis. *PLOS ONE* **11**, e0156990 (2016).
82. He, Y.-F. *et al.* Tie2-Expressing Monocytes Are Associated with Identification and Prognoses of Hepatitis B Virus Related Hepatocellular Carcinoma after Resection. *PloS one* **10**, e0143657 (2015).
83. Matsubara, T. *et al.* TIE2-expressing monocytes as a diagnostic marker for hepatocellular carcinoma correlates with angiogenesis. *Hepatology* **57**, 1416–1425 (2013).
84. Patel, A. S. *et al.* TIE2-expressing monocytes/macrophages regulate revascularization of the ischemic limb. *EMBO Molecular Medicine* **5**, 858–869 (2013).
85. Wang, X. *et al.* Crosstalk between TEMs and endothelial cells modulates angiogenesis and metastasis via IGF1-IGF1R signalling in epithelial ovarian cancer. *British Journal of Cancer* **117**, 1371–1382 (2017).
86. Calcinotto, A. *et al.* Modifications of the mouse bone marrow microenvironment favor angiogenesis and correlate with disease progression from asymptomatic to symptomatic multiple myeloma. *Oncoimmunology* **4**, e1008850 (2015).
87. Panchabhai, S. *et al.* Tumor-associated macrophages and extracellular matrix metalloproteinase inducer in prognosis of multiple myeloma. *Leukemia* **30**, 951–4 (2016).
88. Zheng, Y. *et al.* Macrophages are an abundant component of myeloma microenvironment and protect myeloma cells from chemotherapy drug-induced apoptosis. *Blood* **114**, 3625–3628 (2009).
89. Suyanı, E. *et al.* Tumor-associated macrophages as a prognostic parameter in multiple myeloma. *Annals of hematology* **92**, 669–77 (2013).
90. Ibberson, M. *et al.* TIE-2 and VEGFR kinase activities drive immunosuppressive function of TIE-2-expressing monocytes in human breast tumors. *Clinical cancer research : an official journal of the American Association for Cancer Research* **19**, 3439–49 (2013).

DocuSign Envelope ID: A0702825-E0BD-46DB-A7D6-FEF901E11877

91. Gentner, B. *et al.* Identification of Hematopoietic Stem Cell-Specific miRNAs Enables Gene Therapy of Globoid Cell Leukodystrophy. *Science Translational Medicine* **2**, 58ra84-58ra84 (2010).
92. Chiriaco, M. *et al.* Dual-regulated lentiviral vector for gene therapy of X-linked chronic granulomatosis. *Molecular therapy : the journal of the American Society of Gene Therapy* **22**, 1472–1483 (2014).
93. Galli, R. *et al.* Isolation and characterization of tumorigenic, stem-like neural precursors from human glioblastoma. *Cancer Res* **64**, 7011–7021 (2004).
94. Mazzoleni, S. *et al.* Epidermal growth factor receptor expression identifies functionally and molecularly distinct tumor-initiating cells in human glioblastoma multiforme and is required for gliomagenesis. *Cancer Res* **70**, 7500–7513 (2010).
95. Wong, M. L., Prawira, A., Kaye, A. H. & Hovens, C. M. Tumour angiogenesis: its mechanism and therapeutic implications in malignant gliomas. *J Clin Neurosci* **16**, 1119–1130 (2009).
96. Brem, S., Cotran, R. & Folkman, J. Tumor angiogenesis: a quantitative method for histologic grading. *J Natl Cancer Inst* **48**, 347–356 (1972).
97. Reiss, Y., Machein, M. R. & Plate, K. H. The role of angiopoietins during angiogenesis in gliomas. *Brain Pathol* **15**, 311–317 (2005).
98. Ding, H. *et al.* Expression and hypoxic regulation of angiopoietins in human astrocytomas. *Neuro Oncol* **3**, 1–10 (2001).
99. Zadeh, G. *et al.* Targeting the Tie2/Tek receptor in astrocytomas. *Am J Pathol* **164**, 467–476 (2004).
100. Lee, O. H. *et al.* Expression of the receptor tyrosine kinase Tie2 in neoplastic glial cells is associated with integrin beta1-dependent adhesion to the extracellular matrix. *Mol Cancer Res* **4**, 915–926 (2006).
101. Murdoch, C., Tazzyman, S., Webster, S. & Lewis, C. E. Expression of Tie-2 by human monocytes and their responses to angiopoietin-2. *J Immunol* **178**, 7405–7411 (2007).
102. Winkler, F. *et al.* Kinetics of vascular normalization by VEGFR2 blockade governs brain tumor response to radiation: role of oxygenation, angiopoietin-1, and matrix metalloproteinases. *Cancer Cell* **6**, 553–563 (2004).
103. Zitvogel, L., Galluzzi, L., Kepp, O., Smyth, M. J. & Kroemer, G. Type I interferons in anticancer immunity. *Nature Reviews Immunology* **15**, 405–414 (2015).
104. Rizza, P., Moretti, F., Capone, I. & Belardelli, F. Role of type I interferon in inducing a protective immune response: perspectives for clinical applications. *Cytokine & growth factor reviews* **26**, 195–201 (2015).
105. El-Baky, N. A. & Redwan, E. M. Therapeutic alpha-interferons protein: structure, production, and biosimilar. *Preparative biochemistry & biotechnology* **45**, 109–27 (2015).
106. Ezekowitz, R. A., Mulliken, J. B. & Folkman, J. Interferon alfa-2a therapy for life-threatening hemangiomas of infancy. *The New England journal of medicine* **326**, 1456–63 (1992).
107. Escudier, B. *et al.* Renal cell carcinoma: ESMO Clinical Practice Guidelines for diagnosis, treatment and follow-up†. *Annals of Oncology* **27**, v58–v68 (2016).

DocuSign Envelope ID: A0702825-E0BD-46DB-A7D6-FEF901E11877

108. Dummer, R. *et al.* Cutaneous melanoma: ESMO Clinical Practice Guidelines for diagnosis, treatment and follow-up. *Annals of Oncology* **23**, vii86–vii91 (2012).
109. Öberg, K., Knigge, U., Kwekkeboom, D., Perren, A. & ESMO Guidelines Working Group. Neuroendocrine gastro-entero-pancreatic tumors: ESMO Clinical Practice Guidelines for diagnosis, treatment and follow-up. *Annals of oncology : official journal of the European Society for Medical Oncology* **23 Suppl 7**, vii124-30 (2012).
110. Kirkwood, J. Cancer immunotherapy: the interferon-alpha experience. *Seminars in oncology* **29**, 18–26 (2002).
111. Friedman, R. M. Clinical uses of interferons. *British journal of clinical pharmacology* **65**, 158–62 (2008).
112. Sidky, Y. A. & Borden, E. C. Inhibition of angiogenesis by interferons: effects on tumor- and lymphocyte-induced vascular responses. *Cancer research* **47**, 5155–61 (1987).
113. Pfeffer, L. M. Biologic activities of natural and synthetic type I interferons. *Seminars in oncology* **24**, S9-63-S9-69 (1997).
114. Dunn, G. P., Koebel, C. M. & Schreiber, R. D. Interferons, immunity and cancer immunoediting. *Nature reviews. Immunology* **6**, 836–48 (2006).
115. Parker, B. S., Rautela, J. & Hertzog, P. J. Antitumour actions of interferons: implications for cancer therapy. *Nature reviews. Cancer* **16**, 131–44 (2016).
116. Gessani, S., Conti, L., Del Cornò, M. & Belardelli, F. Type I interferons as regulators of human antigen presenting cell functions. *Toxins* **6**, 1696–723 (2014).
117. Hervas-Stubbs, S. *et al.* Direct Effects of Type I Interferons on Cells of the Immune System. *Clinical Cancer Research* **17**, 2619–2627 (2011).
118. Fuertes, M. B., Woo, S.-R., Burnett, B., Fu, Y.-X. & Gajewski, T. F. Type I interferon response and innate immune sensing of cancer. *Trends in immunology* **34**, 67–73 (2013).
119. Belardelli, F., Ferrantini, M., Proietti, E. & Kirkwood, J. M. Interferon-alpha in tumor immunity and immunotherapy. *Cytokine & growth factor reviews* **13**, 119–34 (2002).
120. Brassard, D. L., Grace, M. J. & Bordens, R. W. Interferon-alpha as an immunotherapeutic protein. *Journal of leukocyte biology* **71**, 565–81 (2002).
121. Blankenstein, T. The role of tumor stroma in the interaction between tumor and immune system. *Current opinion in immunology* **17**, 180–6 (2005).
122. Willimsky, G. & Blankenstein, T. The adaptive immune response to sporadic cancer. *Immunological Reviews* **220**, 102–112 (2007).
123. Theofilopoulos, A. N., Baccala, R., Beutler, B. & Kono, D. H. TYPE I INTERFERONS (α/β) IN IMMUNITY AND AUTOIMMUNITY. *Annual Review of Immunology* **23**, 307–335 (2005).
124. Boëthius, J., Blomgren, H., Collins, V. P., Greitz, T. & Strander, H. The effect of systemic human interferon-alpha administration to patients with glioblastoma multiforme. *Acta neurochirurgica* **68**, 239–51 (1983).
125. Hirakawa, K. *et al.* Effect of human leukocyte interferon on malignant brain tumors. *Cancer* **51**, 1976–81 (1983).

DocuSign Envelope ID: A0702825-E0BD-46DB-A7D6-FEF901E11877

126. Nakagawa, Y. *et al.* Local administration of interferon for malignant brain tumors. *Cancer treatment reports* **67**, 833–5 (1983).
127. Nagai, M. & Arai, T. Clinical effect of interferon in malignant brain tumours. *Neurosurgical review* **7**, 55–64 (1984).
128. Mahaley, M. S. *et al.* Immunobiology of primary intracranial tumors. *Journal of Neurosurgery* **63**, 719–725 (1985).
129. Obbens, E. A. *et al.* Phase I clinical trial of intralesional or intraventricular leukocyte interferon for intracranial malignancies. *Journal of neuro-oncology* **3**, 61–7 (1985).
130. Hamada, H., Asakura, T., Maeda, Y., Yokoyama, S. & Niiro, M. [A study on the direct antitumoral effect of interferon-alpha on human glioma]. *Gan to kagaku ryoho. Cancer & chemotherapy* **13**, 464–71 (1986).
131. Jereb, B., Petric, J., Lamovec, J., Skrbec, M. & Soss, E. Intratumor application of human leukocyte interferon-alpha in patients with malignant brain tumors. *American journal of clinical oncology* **12**, 1–7 (1989).
132. Yoshida, J., Kajita, Y., Wakabayashi, T. & Sugita, K. Long-term follow-up results of 175 patients with malignant glioma: importance of radical tumour resection and postoperative adjuvant therapy with interferon, ACNU and radiation. *Acta neurochirurgica* **127**, 55–9 (1994).
133. Buckner, J. C. *et al.* Phase II evaluation of recombinant interferon alpha and BCNU in recurrent glioma. *Journal of neurosurgery* **82**, 430–5 (1995).
134. Dillman, R. O. *et al.* Interferon alpha-2a and external beam radiotherapy in the initial management of patients with glioma: a pilot study of the National Biotherapy Study Group. *Cancer biotherapy* **10**, 265–71 (1995).
135. Brandes, A. A. *et al.* Phase II trial with BCNU plus alpha-interferon in patients with recurrent high-grade gliomas. *American journal of clinical oncology* **20**, 364–7 (1997).
136. Rajkumar, S. V *et al.* Phase I evaluation of radiation combined with recombinant interferon alpha-2a and BCNU for patients with high-grade glioma. *International journal of radiation oncology, biology, physics* **40**, 297–302 (1998).
137. Vaquero, J. & Martínez, R. Intratumoral immunotherapy with interferon-alpha and interleukin-2 in glioblastoma. *Neuroreport* **3**, 981–3 (1992).
138. Jereb, B. *et al.* Addition of IFN-alpha to treatment of malignant brain tumors. *Acta oncologica (Stockholm, Sweden)* **33**, 651–4 (1994).
139. Olson, J. J. *et al.* Phase I analysis of BCNU-impregnated biodegradable polymer wafers followed by systemic interferon alfa-2b in adults with recurrent glioblastoma multiforme. *Journal of Neuro-Oncology* **90**, 293–299 (2008).
140. Groves, M. D. *et al.* Two phase II trials of temozolomide with interferon-α2b (pegylated and non-pegylated) in patients with recurrent glioblastoma multiforme. *British Journal of Cancer* **101**, 615–620 (2009).
141. Dillman, R. O. *et al.* Interferon-alpha2a and 13-cis-retinoic acid with radiation treatment for high-grade glioma. *Neuro-oncology* **3**, 35–41 (2001).
142. Buckner, J. C. *et al.* A phase III study of radiation therapy plus carmustine with or without recombinant interferon-alpha in the treatment of patients with newly diagnosed high-grade glioma. *Cancer* **92**, 420–33 (2001).

DocuSign Envelope ID: A0702825-E0BD-46DB-A7D6-FEF901E11877

143. Olson, J. J. *et al.* Correlation of the response of recurrent malignant gliomas treated with interferon alpha with tumor interferon alpha gene content. *Int J Oncol* **25**, 419–427 (2004).
144. James, C. D. *et al.* Chromosome 9 deletion mapping reveals interferon alpha and interferon beta-1 gene deletions in human glial tumors. *Cancer Res* **51**, 1684–1688 (1991).
145. James, C. D., He, J., Collins, V. P., Allalunis-Turner, M. J. & Day 3rd, R. S. Localization of chromosome 9p homozygous deletions in glioma cell lines with markers constituting a continuous linkage group. *Cancer Res* **53**, 3674–3676 (1993).
146. Olopade, O. I. *et al.* Molecular analysis of deletions of the short arm of chromosome 9 in human gliomas. *Cancer Res* **52**, 2523–2529 (1992).
147. Bello, M. J. *et al.* Molecular and cytogenetic analysis of chromosome 9 deletions in 75 malignant gliomas. *Genes Chromosomes Cancer* **9**, 33–41 (1994).
148. Negrini, S., Gorgoulis, V. G. & Halazonetis, T. D. Genomic instability--an evolving hallmark of cancer. *Nature reviews. Molecular cell biology* **11**, 220–8 (2010).
149. Rizvi, N. A. *et al.* Cancer immunology. Mutational landscape determines sensitivity to PD-1 blockade in non-small cell lung cancer. *Science (New York, N.Y.)* **348**, 124–8 (2015).
150. Chen, D. S. & Mellman, I. Elements of cancer immunity and the cancer-immune set point. *Nature* **541**, 321–330 (2017).
151. Cancer Genome Atlas Research Network, R. *et al.* Comprehensive genomic characterization defines human glioblastoma genes and core pathways. *Nature* **455**, 1061–8 (2008).
152. Brennan, C. W. *et al.* The somatic genomic landscape of glioblastoma. *Cell* **155**, 462–77 (2013).
153. Alexandrov, L. B. *et al.* Signatures of mutational processes in human cancer. *Nature* **500**, 415–21 (2013).
154. Finocchiaro, G., Langella, T., Corbetta, C. & Pellegatta, S. Hypermutations in gliomas: a potential immunotherapy target. *Discovery medicine* **23**, 113–120 (2017).
155. Derer, A., Frey, B., Fietkau, R. & Gaipl, U. S. Immune-modulating properties of ionizing radiation: rationale for the treatment of cancer by combination radiotherapy and immune checkpoint inhibitors. *Cancer immunology, immunotherapy : CII* **65**, 779–86 (2016).
156. Golden, E. B. & Apetoh, L. Radiotherapy and immunogenic cell death. *Seminars in radiation oncology* **25**, 11–7 (2015).
157. De Palma, M. *et al.* Tumor-Targeted Interferon-α Delivery by Tie2-Expressing Monocytes Inhibits Tumor Growth and Metastasis. *Cancer Cell* **14**, 299–311 (2008).
158. Deininger, M. H., Meyermann, R. & Schluesener, H. J. The allograft inflammatory factor-1 family of proteins. *FEBS Lett* **514**, 115–121 (2002).
159. Costa, B. *et al.* A Set of Cell Lines Derived from a Genetic Murine Glioblastoma Model Recapitulates Molecular and Morphological Characteristics of Human Tumors. *Cancers* **13**, 230 (2021).

DocuSign Envelope ID: A0702825-E0BD-46DB-A7D6-FEF901E11877

160. Birocchi, F. *et al.* Targeted inducible delivery of immunoactivating cytokines reprograms glioblastoma microenvironment and inhibits growth in mouse models. *Sci Transl Med* **14**, eabl4106 (2022).
161. Aiuti, A. *et al.* Lentiviral Hematopoietic Stem Cell Gene Therapy in Patients with Wiskott-Aldrich Syndrome. *Science* **341**, 1233151–1233151 (2013).
162. Biffi, A. *et al.* Lentiviral hematopoietic stem cell gene therapy benefits metachromatic leukodystrophy. *Science (New York, N.Y.)* **341**, 1233158 (2013).
163. Sessa, M. *et al.* Lentiviral haemopoietic stem-cell gene therapy in early-onset metachromatic leukodystrophy: an ad-hoc analysis of a non-randomised, open-label, phase 1/2 trial. *Lancet (London, England)* **388**, 476–87 (2016).
164. Ailles, L. *et al.* Molecular evidence of lentiviral vector-mediated gene transfer into human self-renewing, multi-potent, long-term NOD/SCID repopulating hematopoietic cells. *Molecular therapy : the journal of the American Society of Gene Therapy* **6**, 615–26 (2002).
165. Biffi, A. *et al.* Gene therapy of metachromatic leukodystrophy reverses neurological damage and deficits in mice. *The Journal of clinical investigation* **116**, 3070–82 (2006).
166. Capotondo, A. *et al.* Safety of arylsulfatase A overexpression for gene therapy of metachromatic leukodystrophy. *Human gene therapy* **18**, 821–36 (2007).
167. Cartier, N. *et al.* Hematopoietic stem cell gene therapy with a lentiviral vector in X-linked adrenoleukodystrophy. *Science (New York, N.Y.)* **326**, 818–23 (2009).
168. Guenechea, G. *et al.* Transduction of human CD34+ CD38- bone marrow and cord blood-derived SCID-repopulating cells with third-generation lentiviral vectors. *Molecular therapy : the journal of the American Society of Gene Therapy* **1**, 566–73 (2000).
169. Guenechea, G., Gan, O. I., Dorrell, C. & Dick, J. E. Distinct classes of human stem cells that differ in proliferative and self-renewal potential. *Nature Immunology* **2**, 75–82 (2001).
170. Miyoshi, H., Smith, K. A., Mosier, D. E., Verma, I. M. & Torbett, B. E. Transduction of human CD34+ cells that mediate long-term engraftment of NOD/SCID mice by HIV vectors. *Science (New York, N.Y.)* **283**, 682–6 (1999).
171. Montini, E. *et al.* Hematopoietic stem cell gene transfer in a tumor-prone mouse model uncovers low genotoxicity of lentiviral vector integration. *Nature biotechnology* **24**, 687–96 (2006).
172. Montini, E. *et al.* The genotoxic potential of retroviral vectors is strongly modulated by vector design and integration site selection in a mouse model of HSC gene therapy. *Journal of Clinical Investigation* **119**, 964–975 (2009).
173. Biffi, A. *et al.* Lentiviral vector common integration sites in preclinical models and a clinical trial reflect a benign integration bias and not oncogenic selection. *Blood* **117**, 5332–9 (2011).
174. Lieberman, F. Glioblastoma update: molecular biology, diagnosis, treatment, response assessment, and translational clinical trials. *F1000Research* **6**, 1892 (2017).

DocuSign Envelope ID: A0702825-E0BD-46DB-A7D6-FEF901E11877

175. Taal, W. *et al.* Single-agent bevacizumab or lomustine versus a combination of bevacizumab plus lomustine in patients with recurrent glioblastoma (BELOB trial): a randomised controlled phase 2 trial. *The Lancet. Oncology* **15**, 943–53 (2014).
176. Erdem-Eraslan, L. *et al.* Identification of Patients with Recurrent Glioblastoma Who May Benefit from Combined Bevacizumab and CCNU Therapy: A Report from the BELOB Trial. *Cancer research* **76**, 525–34 (2016).
177. Seystahl, K., Wick, W. & Weller, M. Therapeutic options in recurrent glioblastoma--An update. *Critical reviews in oncology/hematology* **99**, 389–408 (2016).
178. Escobar, G. *et al.* Genetic Engineering of Hematopoiesis for Targeted IFN- Delivery Inhibits Breast Cancer Progression. *Science Translational Medicine* **6**, 217ra3-217ra3 (2014).
179. Adair, J. E. *et al.* Extended survival of glioblastoma patients after chemoprotective HSC gene therapy. *Sci Transl Med* **4**, 133ra57 (2012).
180. Ferreri, A. J. M. *et al.* Whole-brain radiotherapy or autologous stem-cell transplantation as consolidation strategies after high-dose methotrexate-based chemoimmunotherapy in patients with primary CNS lymphoma: results of the second randomisation of the International Extranodal L. *The Lancet Haematology* **4**, e510–e523 (2017).
181. Montemurro, M. *et al.* Primary central nervous system lymphoma treated with high-dose methotrexate, high-dose busulfan/thiotepa, autologous stem-cell transplantation and response-adapted whole-brain radiotherapy: results of the multicenter Ostdeutsche Studiengruppe Hämato-Onkologie OSHO-53 phase II study. *Annals of Oncology* **18**, 665–671 (2007).
182. Kiefer, T. *et al.* Long-term follow-up of high-dose chemotherapy with autologous stem-cell transplantation and response-adapted whole-brain radiotherapy for newly diagnosed primary CNS lymphoma: results of the multicenter Ostdeutsche Studiengruppe Hamatologie und Onkologie OSHO-53 phase II study. *Ann. Oncol.* **23**, 1809–1812 (2012).
183. Young, P. A. *et al.* Durable Survival Outcomes in Primary and Secondary Central Nervous System Lymphoma After High-dose Chemotherapy and Autologous Stem Cell Transplantation Using a Thiotepa, Busulfan, and Cyclophosphamide Conditioning Regimen. *Clin Lymphoma Myeloma Leuk* **20**, 468–479 (2020).
184. Biffi, A. Hematopoietic Stem Cell Gene Therapy for Storage Disease: Current and New Indications. *Mol Ther* **25**, 1155–1162 (2017).
185. Morgan, R. A., Gray, D., Lomova, A. & Kohn, D. B. Hematopoietic Stem Cell Gene Therapy: Progress and Lessons Learned. *Cell Stem Cell* **21**, 574–590 (2017).
186. Shaw, P., Shizuru, J., Hoenig, M., Veys, P., & IEWP-EBMT. Conditioning Perspectives for Primary Immunodeficiency Stem Cell Transplants. *Front Pediatr* **7**, 434 (2019).
187. Aiuti, A. *et al.* Gene Therapy for Immunodeficiency Due to Adenosine Deaminase Deficiency. *New England Journal of Medicine* **360**, 447–458 (2009).
188. Ferrua, F. *et al.* Lentiviral haemopoietic stem/progenitor cell gene therapy for treatment of Wiskott-Aldrich syndrome: interim results of a non-randomised, open-label, phase 1/2 clinical study. *Lancet Haematol* **6**, e239–e253 (2019).

DocuSign Envelope ID: A0702825-E0BD-46DB-A7D6-FEF901E11877

189. Thompson, A. A. *et al.* Gene Therapy in Patients with Transfusion-Dependent β-Thalassemia. *The New England journal of medicine* **378**, 1479–1493 (2018).
190. Kohn, D. B. *et al.* Lentiviral gene therapy for X-linked chronic granulomatous disease. *Nat. Med.* **26**, 200–206 (2020).
191. Aiuti, A. *et al.* Lentiviral Hematopoietic Stem Cell Gene Therapy in Patients with Wiskott-Aldrich Syndrome. *Science* **341**, 1233151–1233151 (2013).
192. Bernardo, M. E. & Aiuti, A. The Role of Conditioning in Hematopoietic Stem-Cell Gene Therapy. *Human Gene Therapy* **27**, 741–748 (2016).
193. Giralt, S. *et al.* Reduced-Intensity Conditioning Regimen Workshop: Defining the Dose Spectrum. Report of a Workshop Convened by the Center for International Blood and Marrow Transplant Research. *Biology of Blood and Marrow Transplantation* **15**, 367–369 (2009).
194. Capotondo, A. *et al.* Brain conditioning is instrumental for successful microglia reconstitution following hematopoietic stem cell transplantation. *Proc Natl Acad Sci U S A* **109**, 15018–15023 (2012).
195. Wilkinson, F. L. *et al.* Busulfan conditioning enhances engraftment of hematopoietic donor-derived cells in the brain compared with irradiation. *Mol. Ther.* **21**, 868–876 (2013).
196. Peters, W. P. *et al.* Clinical and pharmacologic effects of high dose single agent busulfan with autologous bone marrow support in the treatment of solid tumors. *Cancer Res.* **47**, 6402–6406 (1987).
197. Chongsathidkiet, P. *et al.* Sequestration of T cells in bone marrow in the setting of glioblastoma and other intracranial tumors. *Nat. Med.* **24**, 1459–1468 (2018).
198. Gupta, K. & Burns, T. C. Radiation-Induced Alterations in the Recurrent Glioblastoma Microenvironment: Therapeutic Implications. *Front Oncol* **8**, 503 (2018).
199. Bethge, W. A. *et al.* Haploidentical allogeneic hematopoietic cell transplantation in adults with reduced-intensity conditioning and CD3/CD19 depletion: Fast engraftment and low toxicity. *Experimental Hematology* **34**, 1746–1752 (2006).
200. Bacigalupo, A. *et al.* Unmanipulated haploidentical bone marrow transplantation and post-transplant cyclophosphamide for hematologic malignanices following a myeloablative conditioning: an update. *Bone Marrow Transplantation* **50**, S37–S39 (2015).
201. Duléry, R. *et al.* Thiotepa, Busulfan, and Fludarabine Conditioning Regimen in T Cell-Replete HLA-Haploidentical Hematopoietic Stem Cell Transplantation. *Biol. Blood Marrow Transplant.* **25**, 1407–1415 (2019).
202. Fagnoni, F. F. *et al.* T-cell dynamics after high-dose chemotherapy in adults: elucidation of the elusive CD8+ subset reveals multiple homeostatic T-cell compartments with distinct implications for immune competence. *Immunology* **106**, 27–37 (2002).
203. Muranski, P. *et al.* Increased intensity lymphodepletion and adoptive immunotherapy—how far can we go? *Nat Clin Pract Oncol* **3**, 668–681 (2006).
204. Crow, Y. J. Type I interferonopathies: mendelian type I interferon up-regulation. *Curr Opin Immunol* **32**, 7–12 (2015).

DocuSign Envelope ID: A0702825-E0BD-46DB-A7D6-FEF901E11877

205. Cesani, M. *et al.* Shedding of clinical-grade lentiviral vectors is not detected in a gene therapy setting. *Gene therapy* **22**, 496–502 (2015).

206. Visigalli, I. *et al.* Preclinical Testing of the Safety and Tolerability of Lentiviral Vector-Mediated Above-Normal Alpha-L-Iduronidase Expression in Murine and Human Hematopoietic Cells Using Toxicology and Biodistribution Good Laboratory Practice Studies. *Human gene therapy* **27**, 813–829 (2016).

207. CHMP. COMMITTEE FOR MEDICINAL PRODUCTS FOR HUMAN USE (CHMP) GUIDELINE ON NON-CLINICAL TESTING FOR INADVERTENT GERMLINE TRANSMISSION OF GENE TRANSFER VECTORS DRAFT AGREED BY SAFETY WORKING PARTY September 2005 DRAFT AGREED BY GENE THERAPY WORKING PARTY October 2. http://www.emea.europa.eu (2005).

208. EMEA. ICH Considerations General Principles to Address the Risk of Inadvertent Germline Integration of Gene Therapy Vectors. http://www.emea.europa.eu (2006).

209. Ikawa, M. *et al.* Restoration of spermatogenesis by lentiviral gene transfer: Offspring from infertile mice. *Proceedings of the National Academy of Sciences* **99**, 7524–7529 (2002).

210. Park, F. Lentiviral vectors: are they the future of animal transgenesis? *Physiological Genomics* **31**, 159–173 (2007).

211. Lund, A. H. *et al.* Genome-wide retroviral insertional tagging of genes involved in cancer in Cdkn2a-deficient mice. *Nature Genetics* **32**, 160–165 (2002).

212. Suzuki, T. *et al.* New genes involved in cancer identified by retroviral tagging. *Nature Genetics* **32**, 166–174 (2002).

213. Uren, A. G., Kool, J., Berns, A. & van Lohuizen, M. Retroviral insertional mutagenesis: past, present and future. *Oncogene* **24**, 7656–7672 (2005).

214. Hacein-Bey-Abina, S. *et al.* Insertional oncogenesis in 4 patients after retrovirus-mediated gene therapy of SCID-X1. *The Journal of clinical investigation* **118**, 3132–42 (2008).

215. Howe, S. J. *et al.* Insertional mutagenesis combined with acquired somatic mutations causes leukemogenesis following gene therapy of SCID-X1 patients. *The Journal of clinical investigation* **118**, 3143–50 (2008).

216. Braun, C. J. *et al.* Gene therapy for Wiskott-Aldrich Syndrome—Long-term reconstitution and clinical benefits, but increased risk for leukemogenesis. *Rare Diseases* **2**, e947749 (2014).

217. Ott, M. G. *et al.* Correction of X-linked chronic granulomatous disease by gene therapy, augmented by insertional activation of MDS1-EVI1, PRDM16 or SETBP1. *Nature Medicine* **12**, 401–409 (2006).

218. Ott, M. G. *et al.* Advances in the treatment of Chronic Granulomatous Disease by gene therapy. *Current gene therapy* **7**, 155–61 (2007).

219. Recchia, A. *et al.* Retroviral vector integration deregulates gene expression but has no consequence on the biology and function of transplanted T cells. *Proceedings of the National Academy of Sciences* **103**, 1457–1462 (2006).

220. Ryu, B. Y. *et al.* An experimental system for the evaluation of retroviral vector design to diminish the risk for proto-oncogene activation. *Blood* **111**, 1866–75 (2008).

DocuSign Envelope ID: A0702825-E0BD-46DB-A7D6-FEF901E11877

221. Modlich, U. *et al.* Insertional Transformation of Hematopoietic Cells by Self-inactivating Lentiviral and Gammaretroviral Vectors. *Molecular Therapy* **17**, 1919–1928 (2009).
222. Modlich, U. *et al.* Leukemia induction after a single retroviral vector insertion in Evi1 or Prdm16. *Leukemia* **22**, 1519–28 (2008).
223. Montini, E. *et al.* The genotoxic potential of retroviral vectors is strongly modulated by vector design and integration site selection in a mouse model of HSC gene therapy. *Journal of Clinical Investigation* **119**, 964–975 (2009).
224. Biffi, A. *et al.* Lentiviral Hematopoietic Stem Cell Gene Therapy Benefits Metachromatic Leukodystrophy. *Science* **341**, 1233158–1233158 (2013).
225. Sessa, M. *et al.* Lentiviral haemopoietic stem-cell gene therapy in early-onset metachromatic leukodystrophy: an ad-hoc analysis of a non-randomised, open-label, phase 1/2 trial. *Lancet (London, England)* **388**, 476–87 (2016).
226. Biasco, L. *et al.* In Vivo Tracking of Human Hematopoiesis Reveals Patterns of Clonal Dynamics during Early and Steady-State Reconstitution Phases. *Cell Stem Cell* **19**, 107–119 (2016).
227. Montini, E. *et al.* Hematopoietic stem cell gene transfer in a tumor-prone mouse model uncovers low genotoxicity of lentiviral vector integration. *Nature biotechnology* **24**, 687–96 (2006).
228. Cesana, D. *et al.* Whole transcriptome characterization of aberrant splicing events induced by lentiviral vector integrations. *Journal of Clinical Investigation* **122**, 1667–1676 (2012).
229. Gentner, B. *et al.* Stable knockdown of microRNA in vivo by lentiviral vectors. *Nature methods* **6**, 63–6 (2009).
230. Gentner, B. & Naldini, L. Exploiting microRNA regulation for genetic engineering. *Tissue Antigens* **80**, 393–403 (2012).
231. Lechman, E. R. *et al.* Attenuation of miR-126 activity expands HSC in vivo without exhaustion. *Cell stem cell* **11**, 799–811 (2012).
232. Nucera, S. *et al.* miRNA-126 Orchestrates an Oncogenic Program in B Cell Precursor Acute Lymphoblastic Leukemia. *Cancer Cell* **29**, 905–921 (2016).
233. Lechman, E. R. *et al.* miR-126 Regulates Distinct Self-Renewal Outcomes in Normal and Malignant Hematopoietic Stem Cells. *Cancer Cell* **29**, 214–228 (2016).
234. de Haan, G. & Lazare, S. S. Aging of hematopoietic stem cells. *Blood* **131**, 479–487 (2018).
235. Moog, R. Adverse events in peripheral progenitor cell collection: a 7-year experience. *Journal of hematotherapy & stem cell research* **10**, 675–80 (2001).
236. Linnemann, B. Management of Complications Related to Central Venous Catheters in Cancer Patients: An Update. *Seminars in Thrombosis and Hemostasis* **40**, 382–394 (2014).
237. Morris, C. *et al.* Should the standard dimethyl sulfoxide concentration be reduced? Results of a European Group for Blood and Marrow Transplantation prospective noninterventional study on usage and side effects of dimethyl sulfoxide. *Transfusion* **54**, 2514–22 (2014).

DocuSign Envelope ID: A0702825-E0BD-46DB-A7D6-FEF901E11877

238. CHMP. ANNEX I SUMMARY OF PRODUCT CHARACTERISTICS (Intron3A).
239. Schenk-Braat, E. A. M., van Mierlo, M. M. K. B., Wagemaker, G., Bangma, C. H. & Kaptein, L. C. M. An inventory of shedding data from clinical gene therapy trials. *The journal of gene medicine* **9**, 910–21 (2007).
240. FDA CBER. Guidance for Industry: Gene Therapy Clinical Trials – Observing Subjects for Delayed Adverse Events. http://www.fda.gov/cber/guidelines.htm. (2006).
241. CHMP. *COMMITTEE FOR MEDICINAL PRODUCTS FOR HUMAN USE (CHMP) GUIDELINE ON FOLLOW-UP OF PATIENTS ADMINISTERED WITH GENE THERAPY MEDICINAL PRODUCTS AGREED BY GENE THERAPY WP April 2008 AGREED BY PHARMACOVIGILANCE WP March 2008 ADOPTION BY CHMP FOR RELEASE FOR CONS*. (2008).
242. Okada, H. *et al.* Immunotherapy response assessment in neuro-oncology: a report of the RANO working group. *The Lancet Oncology* **16**, e534–e542 (2015).
243. Nayak, L. *et al.* The Neurologic Assessment in Neuro-Oncology (NANO) scale: a tool to assess neurologic function for integration into the Response Assessment in Neuro-Oncology (RANO) criteria. *Neuro-Oncology* **19**, 625–635 (2017).
244. Ellingson, B. M. *et al.* Consensus recommendations for a standardized Brain Tumor Imaging Protocol in clinical trials. *Neuro-oncology* **17**, 1188–98 (2015).
245. Gestione degli studi clinici in Italia in corso di emergenza COVID-19 (coronavirus disease 19) (12/03/2020). https://aifa.gov.it/-/gestione-degli-studi-clinici-in-italia-in-corso-di-emergenza-covid-19-coronavirus-disease-19-.
246. Gestione degli studi clinici in Italia in corso di emergenza COVID-19 (07/04/2020). https://aifa.gov.it/-/gestione-degli-studi-clinici-in-italia-in-corso-di-emergenza-covid-19-coronavirus-disease-19-07-04-2020-.

DocuSign Envelope ID: A0702825-E0BD-46DB-A7D6-FEF901E11877

12 Appendices

12.1 Appendix: Schedule of Events

DocuSign Envelope ID: A0702825-E0BD-46DB-A7D6-FEF901E11877

Table 12-1 Schedule of Events (Screening to Day +29): INCB/OSR (Cohorts 1-6)

[redacted]

[redacted]

Footnotes:

[redacted]

Table 12-2 Schedule of Events (Screening to Day +29): INCB/OSR (Cohorts 7 and 8)

[redacted]

DocuSign Envelope ID: A0702825-E0BD-46DB-A7D6-FEF901E11877

[redacted]

Footnotes:

[redacted]

[redacted]

Table 12-3 Schedule of Events (Day +30 to end of study): INCB/OSR

[redacted]

Footnotes:

[redacted]

Table 12-4 Schedule of Events (Screening to Day +29): PG

[redacted]

[redacted]

Footnotes:

[redacted]

DocuSign Envelope ID: A0702825-E0BD-46DB-A7D6-FEF901E11877

[redacted]

DocuSign Envelope ID: A0702825-E0BD-46DB-A7D6-FEF901E11877

Table 12-5 Schedule of Events (Day +30 to end of study): PG

DocuSign Envelope ID: A0702825-E0BD-46DB-A7D6-FEF901E11877

[redacted]

DocuSign Envelope ID: A0702825-E0BD-46DB-A7D6-FEF901E11877

Footnotes:

[redacted]

12.2 Summary of Prior Protocol Amendments

TEM-GBM_001 Clinical Protocol Amendment 1
SUMMARY OF CHANGES

This protocol (Version 1.2 of 29th October 2018) replaces previous version (Version 1.1 of 11 June 2018).
This amendment includes a number of administrative and clarification changes to the protocol including:

- To modify the contact information for safety reporting (from Product Life Group in the UK to seQure, a specialized pharmacovigilance provider based in Italy).
- To reorganize the schedule of events in order to clarify the assessments being performed at the clinical centers involved in the study
- To correct typographical errors identified in the protocol
- Corrections have been made to the immunophenotype panels which had been incorrectly listed
- Greater clarity is provided on the assessments to be performed

Other administrative changes or corrections have also been performed, as detailed below.

(Note: added text is ***bold in italic***; deleted text is ~~strikethrough)~~

Location	Rationale	New (Version 1.2 of 29th October 2018)
Headers throughout the document, signature pages, page 2, page 37	Version update	***Amendment V1.2 of 29th October 2018*** ~~v1.1 of 11th June 2018~~
Contact List<br>(Page 2)	The contact information for safety reporting has been modified from Product Life Group to seQure, a specialized pharmacovigilance provider based in Italy.	Serious Adverse Event (SAE) Contact Information:<br>~~Product Life Group~~<br>~~Cowley Road~~<br>~~Milton~~<br>~~Cambridge~~<br>~~UK~~

DocuSign Envelope ID: A0702825-E0BD-46DB-A7D6-FEF901E11877

Location	Rationale	New (Version 1.2 of 29th October 2018)
		[redacted]<br><br>**seQure Life Sciences srl**<br>[redacted]
Synopsis (page 28)	Correction of incorrect assessments listed	Absence of abnormal clonal proliferation will be assessed every 6 months with clinical evaluation, blood count with differential to detect abnormal proliferation or cytopenia, routine biochemical tests to detect abnormal organ functions~~, serum immunoglobulin and protein serum electrophoresis for the presence of altered or monoclonal immunoglobulin components~~, flow cytometry on peripheral blood for lymphocyte subpopulations ~~(CD2, CD3, CD4, CD8, CD56, CD16), TCR (alpha/beta), TCR (gamma/delta), CD19, Ig-kappa, Ig-lambda, flow cytometry with monoclonal antibodies for TCR V-Beta families~~, **and** qPCR analysis for measuring vector copy number. Every year, bone marrow aspirates will be performed, including morphological examination, cytogenetic analysis, VCN and detailed immune phenotyping.
Section 1.6.2.7 (page 71)	Clarification on when samples for RCL assessment will be collected	The patients will be followed to exclude the possible development of RCL infection (see above). Samples will be collected **at Screening**, before treatment (at Day -6) and at Day +30, +90, +180, +360 and +720 and then yearly thereafter, as suggested by FDA guidelines (Guidance for Industry, November 2006)[240].

DocuSign Envelope ID: A0702825-E0BD-46DB-A7D6-FEF901E11877

Location	Rationale	New (Version 1.2 of 29th October 2018)
Section 5.7.2 (page 94)	Incorrect reference listed	In Part A, the first patient assigned to the first cohort will receive a single IV infusion of Temferon at Dose Level 1 (~~Error! Reference source not found.~~**Table 5-1**).
Section 6.2.2.5 (Page 101)	Correction in list of immunophenotype markers for flow cytometry	First level screening (every 6 months):<br>Clinical evaluation, blood count with differential to detect abnormal proliferation or cytopenia, routine biochemical tests to detect abnormal organ functions, ~~serum immunoglobulin and protein serum electrophoreses for the presence of altered or monoclonal immunoglobulin components,~~ flow cytometry on peripheral blood for lymphocyte subpopulations (~~CD2,~~ CD3, CD4, CD8, **CD16**, CD56, CD16, ~~TCR (alpha/beta), TCR (gamma/delta),~~ CD19, **CD25**, **CD127**, ~~Igkappa, Ig-lambda, flow cytometry with monoclonal antibodies for TCR V Beta families,~~ **and** qPCR analysis for measuring vector copy number.<br><br>Second level screening (every year):<br>Bone marrow aspirates will be performed, including morphological examination, cytogenetic analysis, VCN and detailed immune phenotyping. ~~Cytogenetic analyses on peripheral blood lymphocytes (karytotype) will also be performed.~~ Pellets and viable cells of BM and/or PB will be archived. Additional molecular tests will be performed if above tests suggest monoclonal expansion (to be performed on recent and archived samples) to study clonality and identify pro-viral integrant as well as all other appropriate imaging and laboratory tests.
Section 6.2.2.8 (page 102)	Clarification that cytokines are to be measured in the CSF	Patients will be subject to lumbar puncture to evaluate the presence of an IFN-α2 gene signature, **cytokines** and VCN in the CSF of Temferon-treated patients and to monitor potential Temferon neurological toxicities

DocuSign Envelope ID: A0702825-E0BD-46DB-A7D6-FEF901E11877

Location	Rationale	New (Version 1.2 of 29th October 2018)
Section 6.2.2.10 (Page 102)	Vital signs are assessed at all scheduled visits	Data on the patient's vital signs must be recorded in the CRF at ~~Screening, Day -6, 0, +2, +30, and~~ all ~~subsequent~~ visits.
Section 6.2.2.11 (Page 102 & 103)	Clarification on when height, weight and neurological examinations should be performed	It includes measurement of height (cm) and body weight (kg). Height is ~~only~~ measured at screening **and at Day -6**; weight is to be assessed at **all scheduled visits except Day 0**. ~~Day -6, +90, +180, and +540.~~ It also includes examination of the head, eyes, ears, nose, and throat, skin, and the endocrine, metabolic, respiratory, cardiovascular, gastrointestinal, and musculoskeletal systems. A neurological examination should also be performed at Screening, Day -6, 0, ~~+2~~**during in-patient monitoring**, **Day** +30 and all subsequent visits. The examination **where possible** should include the neurologic assessment in Neuro-Oncology (NANO) scale[206].
Section 6.2.2.12 (page 103)	Clarification that some organ imaging and functional tests will be performed at either Screening and/or Day -6	**Lung function tests and chest x-ray will be performed at Day -6 whereas ECG and echocardiography will be performed at Screening and at Day -6.** These ~~are to be done at screening and Day -6, and~~**tests will be performed** periodically thereafter to evaluate exclusion criteria and potential Temferon toxicities.
Section 6.2.3.1 (page 104)	Grammatical corrections. Assessment of transduced myeloid cells in peripheral blood to occur at Day -6	The presence of Temferon cells will be determined through **the** evaluation of **the** engraftment of genetically modified cells in the bone marrow and **their** presence in the peripheral blood.<br>• Engraftment will be assessed by vector-specific qPCR on bone marrow myeloid cells at Day +30, +90, +180, +360 and +720 after infusion, and will be performed at an approved laboratory for Phase 1 primary and secondary endpoint analysis at OSR.<br>• The presence of transduced myeloid cells in PB will be assessed by vector-specific qPCR on PB myeloid cells at **Day -6,** +30, +90, +180, +360, +720.

Location	Rationale	New (Version 1.2 of 29th October 2018)
Section 6.2.3.3 (Page 105)	Clarification when ECOG and Karnofsky status will be assessed on the day of Temferon infusion and during hospitalization for hematological monitoring	Performance status (both Eastern Cooperative Oncology Group (ECOG) and Karnofsky scores) will be assessed at Screening, Day -6, **0, during in-patient monitoring, Day** +30, and at all subsequent visits, and will be recorded in the CRF
Section 6.2.3.4 (page 106)	Typographical error	The questionnaires should be completed by the patient at Screening, Day -6, +30, and at all subsequent visits, and the scores will be recorded in the CRF. The questionnaires are included within the SO~~M~~E.
Section 6.2.4.2 (page 107)	Remove duplicated information	Moreover, the concentration of cytokines in CSF including IL-10, ~~IFN-α,~~ etc., may also be evaluated over time
Section 6.2.4.3 (Page 108)	Blood for exploratory analyses will not be taken during the in-hospital monitoring for hematological recovery	Peripheral blood for exploratory analyses will be collected at Screening, Day -6, ~~+1, +2,~~ +30, +60, +90, +120, +180, +240, +360, and at every scheduled visit thereafter including Day +720.
Section 6.2.5.1 (Page 108)	Include all routine haematology analyses	This will include white blood count (including full differential), neutrophils, hemoglobin, **red cell count,** hematocrit, **mean cell volume, mean cell hemoglobin, mean cell hemoglobin concentration** and platelets.
Section 6.2.5.2 (Page 108 and 109)	Complete list of routine clinical chemistry analyses provided and clarification that urinary glucose will also be assessed as part of routine urine test	This will include plasma creatinine, estimated glomerular filtration rate (calculated with Cockroft-Gault formula), **blood** urea **nitrogen**, total and fractionated bilirubin, alanine-amino transferase (ALT), aspartate amino transferase (AST), gamma glutamyl transferase (GGT), lactate dehydrogenase (LDH), **alkaline phosphatase (ALP), phosphate, albumin, creatine kinase, total protein, uric acid, calcium**, glucose, sodium, potassium, magnesium, C reactive protein (CRP). Urine will also be examined for pH, protein, blood, **glucose** and sediment analysis.
Section 6.2.5.7 (Page 109,110)	Clarification that screening for treponema pallidum will be performed and that HBV, HCV and HIV nucleic acid tests will be performed prior to stem	**The treponema pallidum hemagglutination assay will be evaluated at Screening. Nucleic acid tests for HBV, HCV and HIV will be performed prior to stem cell mobilization and leukapheresis. The presence of**

Location	Rationale	New (Version 1.2 of 29th October 2018)
	cell mobilization. Quanteferon assay will also be performed.	**mycobacterium tuberculosis will be determined using the Quantiferon assay at Day -6.**
Section 6.2.5.8 (page 110)	Inclusion of mycoplasma antibodies to be measured. Incorrect subpopulations of leukocytes were specified.	Serum antibodies to pathogens (EBV, CMV, HSV1-2, VZV, Toxoplasma, **Mycoplasma**) will be measured. Immunophenotyping will occur in peripheral blood with ~~lymphocyte~~ **leukocyte** subpopulations **(CD3+, CD3+CD4+, CD3+CD8+, Total CD19+, CD3-[CD16+]+[CD56+], CD3-(CD16+CD56-), CD3-(CD16-CD56+), CD3-(CD16+CD56+), CD25+ bright, CD25+ dim, CD25+ bright/CD127dim-neg, CD25+dim/CD127+, % lymphocytes, % monocytes (CD14+), and % granulocytes**). ~~CD2, CD45, CD4, CD3, CD8, CD19, CD20, CD16, CD56, CD14, TCR (alpha/beta), TCR (gamma/delta), Ig-kappa, Ig-lambda, CD34, CD45RA, CD45RO, CD25, CD45RO, CD27, s-IgM, s-IgD, CD62L, DR percentage and absolute count.~~ For timing of evaluations see SOE (Table 12 1).
Section 6.3.3 (Page 111, 112)	Change in pharmacovigilance provider	Any AE that meets SAE criteria (Section 6.3.1.4) must be reported to ~~Product Life Group~~ se**Qure Life Sciences** immediately (i.e., within 24 hours)<br><br>[redacted]<br><br>[redacted]
Section 6.7 (page 116)	Some samples may be stored at at -20°C.<br>Correction of typographical error	Samples from peripheral blood (serum, plasma, cell pellets, viable cells in DMSO, ~~cell lines,~~ DNA), CSF and tumors will be collected, **processed** and stored at -**20°C**, -80°C or in liquid nitrogen vapor at OSR and INCB, according to Institutional guidelines.<br>Details are provided in the SO~~M~~E.

Location	Rationale	New (Version 1.2 of 29th October 2018)
Section 7.4.2 (Page119)	Correct error in QOL scores analysis	The mean, median, SD and range for EORTC QLQ **C30 and** BN20 **scores** over time ~~using the ECOG and KPS~~ will be summarized. Changes from Screening and Day -6 will be determined.
Section 12.1 (pages 142-145)	Schedule of events has been redesigned to provide greater clarity on the assessments and investigations that should be performed at each visit and at each center. Simplification of the in-patient monitoring period (Day +1 to +29) on the SOE.	See new schedule of events Table 12.1 and Table 12.2

TEM-GBM_001 Clinical Protocol Amendment 2
SUMMARY OF CHANGES

This protocol (Version 2.0 of 5th February 2019) replaces previous version (Version 1.2 of 29th October 2018).

This amendment includes a number of substantial, administrative and clarification changes to the protocol including:

- Omission of temozolomide from the treatment regimen during the 6 weeks of radiotherapy
- Maintain blood platelet concentrations of at least 50 x 10^3/mm^3 during the hospitalization period post Temferon administration
- Alignment with approved changes to methods and procedures included in a parallel Temferon study in patients with multiple myeloma (EudraCT 2018-001741-14)
- To correct typographical errors identified in the protocol

Other administrative changes or corrections have also been performed, as detailed below.

(Note: added text is ***bold in italic***; deleted text is ~~strikethrough)~~

Location	Rationale	New (Version 2.0 of 5th February 2019)
Headers throughout the document, signature pages, page 2	Version update	***Amendment V2.0 of 5th February 2019***~~1.2 of 29th October 2018~~
Synopsis, page 22<br><br>Section 4.1.1. page 85	Multiple methods of contraception required for women<br><br>(Alignment with approved Temferon protocol EudraCT 2018-001741-14)	Inclusion criteria:<br><br>Women of child-bearing potential enrolled in the study must have a negative pregnancy test at screening and agree to use ***two distinct*** acceptable methods of contraception during the trial.
Synopsis,page 33	Erroneous text inserted into previous document	The safety of the myeloid-targeted IFN-α2 transgene product will be evaluated from hematologic engraftment to Day +720 by clinical and laboratory surveillance, including demonstration of 1) stable blood counts and absence of

Location	Rationale	New (Version 2.0 of 5th February 2019)
Section 6.2.2.6 page 106		cytopenias > grade 2, not related to ~~myeloma progression or~~ concomitant medications;
Synopsis, page 34<br><br>Section 5.2, page 93	Clarification on timing of lenograstim and plerixafor administration<br><br>(Alignment with approved Temferon protocol EudraCT 2018-001741-14)	Synopsis<br>~~Lenograstim 10 µg/kg/day (divided in 2 doses) s.c. for 3 days. CD34+ cell counts will be checked daily during leukapheresis from day 3. Plerixafor 0.24 mg/kg s.c. at 12h before commencement of leukapheresis~~<br>***Lenograstim 10 µg/kg/day divided in 2 administrations (5 µg/kg each) by subcutaneous injection (s.c.) for 3 days or until a sufficient number of CD34+ cells is mobilized and the leukapheresis/-es has/have been concluded. Monitoring of CD34+ cells in the PB will be performed.***<br><br>***Plerixafor 0.24 mg/kg s.c. 5h before commencement of leukapheresis.***<br><br>***If required, another leukapheresis will be performed the day after, preceded by a second plerixafor dose 5 hrs before.***<br><br>Section 5.2<br>~~Lenograstim 10 µg/kg/day (divided in 2 doses) will be administered subcutaneously for 3 days. Plerixafor 0.24 mg/kg will be administered subcutaneously at 8-10 hours before commencement of leukapheresis. CD34+ cell counts will be checked daily during leukapheresis from day 3.~~<br>***Patients will receive lenograstim at the dose of 10µg/kg/day divided in 2 administrations (5µg/kg each) by subcutaneous injection (s.c.) for 3 days or until a sufficient number of CD34⁺ cells is mobilized and the leukapheresis/-es has/have been concluded. Monitoring of CD34⁺ cells in the PB will be performed. Patients will receive plerixafor before each leukapheresis.*** Standard leukapheresis will be performed ***at 5 hours following administration***

DocuSign Envelope ID: A0702825-E0BD-46DB-A7D6-FEF901E11877

Location	Rationale	New (Version 2.0 of 5th February 2019)
		of plerixafor 0.24 mg/kg s.c. and shortly after the last lenograstim administration according to the $CD34^+$ cell count and in agreement with internal procedure of the OSR Stem Cells Program.
Synopsis – ATMP, Dosage and Route of Administration, page 35<br><br>Section 5.7.1, page 98	To clarify that Temferon dose adjustments based on in vitro VCN will be performed by Genenta Science in agreement with the Principal Investigator<br><br>(Alignment with approved Temferon protocol EudraCT 2018-001741-14)	Temferon dose adjustments will be performed by the ~~CMO~~ ***Sponsor, in agreement with the Principal Investigator***, according to VCN in the DP. ~~The adjusted dose will be released to the Investigator for administration~~
Synopsis, many pages<br><br>Section 1.2.3, page 50<br>Section 1.4, page 63<br>Section 3.1, page 82<br>Section 3.4.3, page 85	Following discussion with the data monitoring committee, a recommendation was made to omit temozolomide (TMZ) from the standard of care regimen during the 6 weeks of radiotherapy. This is justified as GBM patients with unmethylated MGMT have no benefit from treatment with TMZ compared to use of radiotherapy alone, and the potential for additive toxicity particularly from a hematological perspective means that it is justified to omit this from the treatment regimen. Recently completed and ongoing studies in GBM patients with unmethylated MGMT have omitted TMZ from the treatment regimen.	Synopsis<br>In Part A, the primary objective is to evaluate the safety and tolerability of 3 escalating doses of Temferon in up to 9 patients with GBM who have an unmethylated MGMT promoter, following first line radiotherapy ~~and temozolomide treatment~~.<br><br>In Part B, the primary objective is to evaluate the safety and tolerability of a single dose, as identified from Part A, of Temferon in 12 patients with GBM and unmethylated MGMT, following first line radiotherapy ~~and temozolomide treatment~~.<br><br>Study phases<br>3. HSPC harvesting (within 4 weeks following Screening), which will occur before standard of care treatment with radiotherapy ~~and TMZ~~ administered for 6 weeks.

DocuSign Envelope ID: A0702825-E0BD-46DB-A7D6-FEF901E11877

Location	Rationale	New (Version 2.0 of 5th February 2019)
		4. Temferon production phase, following HSPC harvest and up to Temferon release for clinical use by qualified person (QP). This will occur while standard of care treatment with radiotherapy ~~and TMZ~~ occurs.<br><br>Section 1.2.3<br>Unmethylated MGMT promoter status is identified in approximately 60% of GBM but despite the evidence suggesting that this group of patients do not benefit from adjuvant therapy with TMZ, the lack of alternative options means that these patients often receive TMZ ***in routine clinical practice. This approach may also be associated with harm, with worse outcomes in some patients with unmethylated MGMT compared to treating with radiotherapy alone[14,51,54]. Adding a new drug or agent on top of the previously established combined modality regimen may cause undue toxicity or drug interaction, thus requiring dose reduction and treatment with potentially subtherapeutic doses. Therefore, in both recently published as well as in currently ongoing clinical studies in GBM patients with unmethylated MGMT, the standard approach is to omit TMZ in groups receiving investigational medicinal products[55-64].***<br><br>Section 1.4<br>Use of adjuvant TMZ has not been shown to be of benefit to this subgroup of ***patients and in some cases may be harmful.***<br><br>Administering Temferon following surgical resection of the tumor and completion of radiotherapy ~~and TMZ~~ may be the optimal timepoint for direct inhibition of residual tumor cell growth, inhibition of neo-angiogenesis, enhanced recruitment and activation of innate immune cells, and recruitment

Location	Rationale	New (Version 2.0 of 5th February 2019)
		and activation of T-cells, in order to maximize the inhibition of disease progression<br><br>Section 2.1<br>In Part A, the primary objective is to evaluate the safety and tolerability of 3 escalating doses of Temferon in up to 9 patients with GBM who have an unmethylated MGMT promoter, following first line radiotherapy ~~and temozolomide treatment~~.<br>In Part B, the primary objective is to evaluate the safety and tolerability of a single dose, as identified from Part A, of Temferon in 12 patients with GBM and unmethylated MGMT, following first line radiotherapy ~~and temozolomide treatment~~.<br><br>Section 3.1<br>A standard of care regimen of radiotherapy ~~and TMZ~~ lasting approximately 6 weeks, will then take place ~~at INCB~~.<br><br>Section 3.4.3<br>Following HSPC harvest, patients will receive ~~standard of care~~ treatment with radiotherapy ~~and~~ ***but without*** TMZ for approximately 6 weeks
Section 1.6.2.7, page 75<br><br>Section 6.2.2.3, page 105	The EMA guideline CHMP/BWP/2458/03 defines the best assays for the determination of RCL. Between these, the detection of HIV p24 antigen in serum by ELISA is identified as a validated and sensitive test (it is routinely used in the clinic to determine HIV positivity in cases of suspected viral infection by the wild type lentivirus). In light of the	Section 1.6.2.7<br>The patients will be followed to exclude the possible development of RCL infection (see above). Samples will be collected at Screening, ~~before treatment (at Day -6)~~ and at Day ~~+30,~~ +90, ~~+180,~~ +360 and +720 and ~~then~~ yearly thereafter, as suggested by FDA ***and EMA*** guidelines (Guidance for Industry, November 2006; ***Guideline on follow-up of patients administered with gene therapy medicinal products, EMEA/CHMP/GTWP/60436/2007***)[205,206] In case

DocuSign Envelope ID: A0702825-E0BD-46DB-A7D6-FEF901E11877

Location	Rationale	New (Version 2.0 of 5th February 2019)
	above, it is proposed in this amendment to use only p24 ELISA assay to assess for RCL, in line with clinical practice. In the case of reconfirmed positivity of this test, further analyses will be performed, including PCR on genomic DNA for VSV-G gene and RT-PCR for RNA-Pol (HIV). In case of further positivity reconfirmed for at least one of these tests, an amplification test in vivo may be conducted in suitable animal models.<br><br>(Alignment with approved Temferon protocol EudraCT 2018-001741-14)	the samples collected during the first two years result always negative for the screening tests (***ELISA for*** HIV p24 ***antigen in serum***), ~~and vesicular stomatitis virus- G DNA and HIV-pol RNA,~~ the subsequent yearly samples will be stored, but not analysed. ~~If one of the above-mentioned tests will result positive, the screening evaluations will be repeated at the next planned follow-up visit; in the case two of the three screening test will result positive, a confirmatory RCL culture tests will be performed~~.<br><br><u>Section 6.2.2.3</u><br>This will consist in the absence of positive results at ELISA for HIV p24 antigen (serum). ***In case of positivity, second level testing may include a) DNA PCR for VSV-G envelope (cells), b) RT-PCR for HIV-pol RNA (serum) and c) in vivo amplification in suitable animal models.*** The tests will be performed at Screening, ~~Day -6,~~ Day +90, +360 and +720. ~~If one of the tests listed above is positive, the three RCL evaluations will be repeated at the next planned follow-up visit; in the case two of the three screening test are positive, a confirmatory RCL will be performed.~~ The presence of anti-HIV p24 antibodies will be evaluated as part of the RCL evaluation as well as evidence of immunization.
Section 6.2.2.8 page 109<br><br>Section 6.2.4.2 page 113<br><br>Schedule of events, Footnote 15	Routine evaluation of CSF includes white cell count, protein and glucose concentrations	<u>Section 6.2.2.8</u><br>Patients will be subject to lumbar puncture to evaluate the presence of an IFN-α2 gene signature, cytokines and VCN in the CSF of Temferon-treated patients and to monitor potential Temferon neurological toxicities. ***Routine laboratory assessment of the CSF will also occur (white cell count, protein and glucose).*** Lumbar puncture will occur at Screening, Day -6, +30, +90, +180, +360 and +720.

Location	Rationale	New (Version 2.0 of 5th February 2019)
		Section 6.2.4.2<br>***Following collection, routine evaluation of a 1ml aliquot of CSF will occur including white cell count, protein and glucose concentrations.***<br><br>Schedule of events footnote 15<br>15. ***Routine evaluation should include white cell count, protein and glucose.*** 4ml of CSF will be stored in the biobank
Section 5.11.1, page 102	Following discussion with the data monitoring committee, it was recommended that during hospitalization following administration of Temferon, blood platelet concentrations ideally be maintained ≥ 50 x 10^3/mm^3 in order to reduce the risk of intracranial hemorrhage.	Erythrocyte and platelet concentrates will be transfused according to clinical need; ***the investigator should consider infusion of platelet concentrates should blood platelet concentration be less than 50 x 10^3/mm^3***. All blood products will be leukocyte depleted and irradiated. All efforts will be made to provide HLA-matched platelets in immunized patients with documented anti-HLA antibodies.
Section 6.2.2.5, page 108	Provide consistency within the document and more detail regarding the assessments for abnormal clonal proliferation<br><br>(Alignment with approved Temferon protocol EudraCT 2018-001741-14)	First level screening (every 6 months):<br>Clinical evaluation, blood count with differential to detect abnormal proliferation or cytopenia, routine biochemical tests to detect abnormal organ functions, flow cytometry on peripheral blood for lymphocyte subpopulations ~~(CD3, CD4, CD8, CD16, CD56, CD16, CD19, CD25, CD127, s,~~ ***see Section 6.2.5.8)*** and qPCR analysis for measuring vector copy number.<br><br>Second level screening (every year):<br>Bone marrow aspirates will be performed, including morphological examination, cytogenetic analysis, VCN and detailed immune phenotyping ***(Granulocytes, Monocytes (CD14+), Lymphocytes, MCD2+ (T cells), MCD19+ (B cells), MCD45+, MCD38+, MCD117+, MCD56+, MCD14+, MCD34+)***. Pellets and viable cells of BM and/or PB will be archived.

Location	Rationale	New (Version 2.0 of 5th February 2019)
		Additional molecular tests will be performed if above tests suggest monoclonal expansion (to be performed on recent and archived samples) to study clonality and identify pro-viral integrant as well as all other appropriate imaging and laboratory tests.<br><br>***Additional tests and procedures may be performed on fresh or archived samples, as appropriate, in case of abnormal findings:***<br>***1. Mutational profiling of cryopreserved BM and/or PB cells;***<br>***2. B or T cell clonality tests using flow cytometry with monoclonal antibodies for TCR Vbeta families, or molecular assays assessing B-cell Receptor (BCR) or T-cell Receptor (TCR) repertoires;***<br>***3. Establishment of cell lines;***<br>***4. RT-PCR for expression of vector and genes next to the integration sites;***<br>***5. All other appropriate imaging and laboratory tests.***
Section 6.2.4.4, page 114	Greater detail provided on the assessments for IFN-α gene signature in the bone marrow.<br><br>(Alignment with approved Temferon protocol EudraCT 2018-001741-14)	~~Exploratory analyses on bone marrow will include the evaluation of IFN-stimulated gene signatures over time (Screening, +30, +90, +180, +360 and +720).~~<br>***Temferon signature will be estimated through evaluation of transgene expression and the presence of an interferon-induced gene signature:***<br>• ***IFN-α2 transgene expression will be evaluated on bone marrow enriched for myeloid cells by RT-qPCR at the Screening visit (negative control) and at Days +30 and +90. This assay may also be performed at Days +180, +360 and +720 post Temferon infusion if engraftment of gene-modified cells is documented by VCN and/or IFN signature.***

DocuSign Envelope ID: A0702825-E0BD-46DB-A7D6-FEF901E11877

Location	Rationale	New (Version 2.0 of 5th February 2019)
		• ***An IFN-induced gene signature comprising OAS1, IRF7 and IFIT1 will be measured on BM mononuclear cells by quantitative RT-PCR at the Screening visit (negative control) and at Days +30, +90, +180, +360 and +720 post Temferon infusion. Additional evaluation of IFN-induced gene signatures on T cells (CD3) and myeloid cells (CD33+) may also be performed at these timepoints dependent on the results of the analysis of BM mononuclear cells.***
Section 6.2.4.5, page 115	Greater detail provided on clonal tracking via integration site analysis provided in text<br><br>(Alignment with approved Temferon protocol EudraCT 2018-001741-14)	***6.2.4.5 Clonal tracking of Temferon graft over time in different hematopoietic lineages***<br><br>***Integration site analysis on distinct hematopoietic lineages will be performed on PB and/or BM at Screening (negative control), Day +30, 90, 360 and 720, and yearly thereafter as part of the separate long-term follow-up study, as long as gene-modified cells can be detected by VCN analysis.***
Schedule of Events, pages 148-151	Correction of missing information and typographical errors	Footnote 2: All brain MRI procedures will be conducted at INCB<br><br>Footnote 16. Bone marrow evaluation will include morphology and immunophenotype. Karyotype and clonogenic progenitors will also be assessed at Screening, Day +***30***, 90, ***180***, 360 and 720.

DocuSign Envelope ID: A0702825-E0BD-46DB-A7D6-FEF901E11877

TEM-GBM_001 Clinical Protocol Amendment 3
SUMMARY OF CHANGES

This protocol (Version 3.0 of 27th April 2020) replaces previous version (Version 2.0 of 5th February 2019).

This amendment includes several substantial, administrative and clarification changes to the protocol including:

- Instructions on the follow up of patients during the COVID-19 pandemic
- Clarification on the required interval between patients in each cohort in Part A
- Instruct that peripheral blood be collected for the measurement of VCN at the time of hematological engraftment
- Clarify that dose adjustments may use data based on VCN expressed to 2 decimal places
- Permit the assessment of IFN-α concentrations and VCN in peripheral blood in the event of Serious Adverse Events, should these be clinically indicated
- Clarification on the routine analysis to be performed on cerebrospinal fluid and CSF collection at time of second surgery
- To correct typographical errors identified in the protocol and schedule of events

Other administrative changes or corrections have also been performed, as detailed below.

(Note: added text is ***bold in italic***; deleted text is ~~strikethrough)~~

Location	Rationale	New (Version 3.0 of 27th April 2020)
Headers throughout the document, signature pages, page 2	Version update	***Amendment V3.0 of 27th April 2020*** ~~V2.0 of 5th February 2019~~
Synopsis & Section 5.7.2.	Clarification that in Part A, conditioning for the 2nd and 3rd patients in each cohort should not commence until the preceding patient reaches the Day+30 visit	For each cohort, a minimum of 30 days will separate dosing of the first ***patient*** and ***commencement of conditioning for the*** second patient within a cohort. ***Conditioning*** ~~Dosing~~ of the third patient will occur a minimum of 30 ~~60~~ days after the ***second*** ~~first~~ patient received Temferon.

DocuSign Envelope ID: A0702825-E0BD-46DB-A7D6-FEF901E11877

Location	Rationale	New (Version 3.0 of 27th April 2020)
Section 5.7.1	Availability of VCN expressed to 2DP where the value falls on the thresholds for dose adjustment	***If necessary, the VCN will be reported to 2 decimal places to assist with dose adjustment decisions.***
Section 6.2.2.5	CD27+ accidentally omitted from previous list	Bone marrow aspirates will be performed, including morphological examination, cytogenetic analysis, VCN and detailed immune phenotyping [Granulocytes, Monocytes (CD14+), Lymphocytes, MCD2+ (T cells), MCD19+ (B cells), ***MCD27***+, MCD45+, MCD38+, MCD117+, MCD56+, MCD14+, MCD34+].
Sections 6.2.2.8, 6.2.4.2 & Schedule of Events	Clarification of routine analysis of CSF	Patients will be subjected to lumbar puncture to evaluate the presence of an IFN-α2 gene signature, cytokines and VCN in the CSF of Temferon-treated patients and to monitor potential Temferon neurological toxicities. Routine laboratory assessment of the CSF will also occur (***glucose, total protein, albumin (and serum albumin), ratio albumin CSF: albumin serum, IgG CSF, IgG CSF Index (LINK), CSF cell count and cytology*** ~~white cell count, protein and glucose~~). Lumbar puncture will occur at Screening, Day -6, +30, +90, +180, +360 and +720. ***In the event that a patient, who has received Temferon, undergoes a second craniotomy for resection of GBM, then CSF will be obtained directly via the transcranial route if surgically appropriate, or by lumbar puncture immediately prior to surgery.***<br><br>Following collection, routine evaluation of a 1ml aliquot of CSF will occur including ***glucose, total protein, albumin (and serum albumin), ratio albumin CSF: albumin serum, IgG CSF, IgG CSF Index (LINK), CSF cell count and cytology*** ~~white cell count, protein and glucose~~.

DocuSign Envelope ID: A0702825-E0BD-46DB-A7D6-FEF901E11877

Location	Rationale	New (Version 3.0 of 27th April 2020)
Section 6.2.2.8, 6.2.4.2 & Schedule of Events	CSF to be collected at the time of second surgery for resection of progressive GBM	The CSF will be evaluated at Screening, Day -6, +30, +90, +180, +360 and +720 to assess the IFN-stimulated gene signatures over time (including Ifn2a, Irf7, Oas1a, Ifit1) and to determine the concentration of IFN-α2 (ELISA). ***In the event that a patient, who has received Temferon, undergoes a second craniotomy for resection of GBM, then CSF will be obtained directly via the transcranial route if surgically appropriate, or by lumbar puncture immediately prior to surgery.*** Furthermore, if a suitable number of cells is detected in the CSF, the presence of transduced cells will be evaluated using VCN.
Section 6.2.3.1 & Schedule of Events table	Clarification that VCN in peripheral blood mononuclear and myeloid lineage will be evaluated at the time of hematological recovery and also may be performed at the time of an SAE	• The presence of transduced myeloid cells in PB will be assessed by vector-specific qPCR on PB myeloid cells at Day -6, ***hematological engraftment, Day*** +30, +90, +180, +360, +720.<br>• ***The presence of transduced myeloid cells in PB may also be assessed, according to the Investigator's clinical judgement, at the time of a Serious Adverse Event.***
Section 6.2.4.3	Clarification that IFN-α2 concentrations in PB may be assessed at the time of an SAE	Changes in IFN-α2 concentration over time will be evaluated in PB. ***IFN-α2 concentration in PB may also be evaluated at the time of Serious Adverse Events, according to the Investigator's clinical judgement.*** Serum samples will be frozen and stored for future post-hoc analyses including the possibility of exosome sequencing, transcriptomics, proteomics and metabolomics, for future biomarker development.

DocuSign Envelope ID: A0702825-E0BD-46DB-A7D6-FEF901E11877

Location	Rationale	New (Version 3.0 of 27th April 2020)
Section 6.2.5.7	Requirement to periodically test patients for the presence of SARS-COV-2 according to local hospital policies	Nucleic acid tests for HBV, HCV and HIV will be performed prior to stem cell mobilization and leukapheresis. The presence of mycobacterium tuberculosis will be determined using the Quantiferon assay at Day -6. ***During the COVID-19 pandemic, the presence of the SARS-COV-2 virus will also be determined periodically according to local hospital policies.***
Section 6.2.6	New section providing instructions on the assessment and follow-up of patients during the COVID-19 pandemic	***The COVID-19 pandemic commenced in Italy in approximately February 2020 and regulatory guidance was issued by AIFA in March[245] and updated in April 2020[246] relating to the management of clinical trials during the pandemic. The Sponsor has incorporated this guidance and as part of a risk assessment, has assessed the potential benefits and risks of continuing the TEM-GBM_001 study and considered modifications to the follow-up of patients participating in the study.***<br><br>***Hospitals participating in the TEM-GBM_001 study have clear policies and guidance in place which aim to protect both patients and staff against the transmission of SARS-CoV-2 virus. These local policies must be followed by hospital staff and changes to policies will be tracked by the Sponsor. Any change in local policies that may limit further recruitment or retention of patients in the study must be notified to the Sponsor's Chief Medical Officer immediately.***<br><br>***In addition, it is important that compliance with good clinical practice (GCP) is maintained and wherever possible risks to trial integrity is minimized. The TEM-GBM_001 study is open label in design and continued recording of data according to the protocol requirements is important. In-person visits are essential to ensure the safety of patients and integrity of the study is maintained. However,***

Location	Rationale	New (Version 3.0 of 27th April 2020)
		protocol deviations are likely to occur due either to local restrictions mandated by government or institutional requirements or by decisions made by the patient due to the need to self-isolate. All deviations arising as a result of the COVID-19 pandemic (with reasons provided) should be recorded in source documentation for individual patients.<br><br>***The Investigator (or delegate) should discuss with the Sponsor's Chief Medical Officer (or delegate) specific cases where alternative arrangements are anticipated as a result of the COVID-19 pandemic. These include:***<br><br>• ***Delay in anticipated apheresis dates which will impact the manufacturing of Temferon (outside the permitted treatment window specified in the protocol).***<br><br>• ***Delay in admission of the patient to the bone marrow transplant unit (outside the permitted treatment window specified in the protocol).***<br><br>• ***Use of alternative public laboratory facilities for patient tests (i.e. facilities not currently specified in the clinical trial application).***<br><br>***The Sponsor's Chief Medical Officer (or delegate) will advise on whether the alternative arrangement should occur (and be recorded in the Sponsor's Trial Master File) or whether the patient should be withdrawn from the study.***<br><br>***All efforts will be made to retain patients, who have received Temferon, in the clinical study and to follow the specified protocol schedule of events. In the event that a patient is unable or unwilling to attend the clinical unit for a protocol mandated visit, ideally video consultation will be permitted and all adverse events***

DocuSign Envelope ID: A0702825-E0BD-46DB-A7D6-FEF901E11877

Location	Rationale	New (Version 3.0 of 27th April 2020)
		(ongoing and new) and concomitant medications will be recorded in the source document. If video is not available, then telephone consultation will be permitted. If at the next visit (scheduled or unscheduled) the patient attends the clinic, investigations and procedures that were omitted previously will be reviewed and after discussion with the Sponsor's Medical Monitor, a decision will be made to either perform or omit these procedures. The agreed decision will be recorded in the source document and Sponsor Trial Master File.<br><br>***Any routine investigations that may be performed in public laboratories near the patient's home, should be discussed by the Investigator with the Sponsor's Medical Monitor prospectively and the agreed decision will be recorded in the source document and Sponsor Trial Master File.***<br><br>***During the period of the pandemic and in view of the importance of following government instructions on the restriction of movements of individuals, on-site monitoring will be suspended and increased remote monitoring will be performed.***

TEM-GBM_001 Clinical Protocol Amendment 4
SUMMARY OF CHANGES

This protocol (Version 4.0 of 17th July 2020) replaces previous version (Version 3.0 of 27th April 2020).

This amendment includes several substantial, administrative and clarification changes to the protocol including:

- Recruitment and management of study patients in three centers in Italy (Ospedale San Raffaele, Istituto Neurologico Carlo Besta, Policlinico Universitario Fondazione Agostino Gemelli)
- Addition of 2 cohorts to Part A in order to evaluate the safety and hematologic engraftment associated with two conditioning regimens (BCNU and thiotepa, or busulfan and thiotepa) when administered prior to intravenous infusion of 2 x 10^6 non-modified $CD34^+$ cells/kg and Temferon Dose Level 3
- Exclusion at Screening from the study of patients with a known hypersensitivity to busulfan
- Permit additional leukapheresis to be performed after radiotherapy is completed in order to harvest sufficient non-modified $CD34^+$ cells to be used for administration prior to Temferon or as a backup

Other administrative changes or corrections have also been performed, as detailed below.

(Note: added text is ***bold in italic***; deleted text is ~~strikethrough)~~

Location	Rationale	New (Version 4.0 of 17th July 2020)
Headers throughout the document, signature pages, page 2	Version update	***Amendment V4.0 of 17th July 2020*** ~~V3.0 of 27th April 2020~~
Synopsis, Section 1.5	Rationale for busulfan and thiotepa conditioning regimen	The need for Temferon transduced cells to engraft in bone marrow requires a conditioning regimen. ~~Key~~ ***Ideally the*** requirements for such a regimen include ***achieving rapid and stable engraftment of transduced HSPCs with long term differentiation of myeloid progeny and mobilisation to the tumor tissue of TEMs carrying the IFN-α2***

Location	Rationale	New (Version 4.0 of 17th July 2020)
		transgene, a direct cytotoxic effect on glioma cells, and minimizing the risk of complete myeloablation which is associated with potentially significant risks relating to infection, anemia, bleeding and end organ damage. A number of options have been considered including high dose carmustine (BCNU) as utilized in a previous gene therapy study in GBM[179], a combination of carmustine and thiotepa[180] ***or busulfan and thiotepa***[181–183] as utilized in primary central nervous system lymphoma, ***busulfan as used in many completed and ongoing gene therapy studies***[163,184–190], and other low intensity conditioning regimens [191,192] ~~including the combination with thiotepa~~.<br><br>***Once the safety and tolerability of increasing doses of Temferon has been assessed using carmustine and thiotepa as a conditioning regimen, an alternative conditioning approach using busulfan, instead of carmustine, in combination with thiotepa will be evaluated in a cohort of 3 patients (Cohort 5). The aim is to evaluate whether conditioning with busulfan (instead of carmustine) in combination with thiotepa may result in a more stable engraftment of Temferon cells that ultimately leads to a greater number and potentially longer term differentiation of transduced myeloid progenitors into IFN-α2 expressing TEMs that migrate into the residual tumor. Busulfan has direct effects on myeloid cell turnover in the brain and may potentiate engraftment of HSPC derived cells in the brain[194,195]. Importantly, historical data confirm that, even at doses higher than those proposed for this study, busulfan when administered as a monotherapy for conditioning, has greater specificity for myeloid precursors with relative sparing of lymphocytes[196]. Recent findings from ongoing gene therapy studies support this observation (Dr. Bernhard Gentner – Personal Communication) and this is an important consideration for achieving efficacy with Temferon taking into account the known sequestration of T cells in the bone marrow in GBM[197]. Using therapeutic drug monitoring (TDM), complete myeloablation may be avoided by targeting busulfan exposure to achieve an area under the plasma concentration curve (AUC) of 40-60 mg.h/L. Busulfan will be administered IV 4 days prior to Temferon at a dose of 3.2 mg/kg ideal body weight, with a further TDM adjusted dose administered 24 hours (and if necessary 48 hours) later.***<br><br>***The combination of busulfan and thiotepa for conditioning is important particularly when considering early homing of Temferon derived TEMs to the brain tumor in tandem with the pro-inflammatory tumor environment that is likely to exist after radiotherapy[198]. An engraftment peak of transduced progenitors at Day +30 could be beneficial and early engraftment has typically been associated with inclusion of thiotepa as part of conditioning regimens[199–201]. Another argument in favor of inclusion of thiotepa as part of the conditioning regimen is that pre-***

Location	Rationale	New (Version 4.0 of 17th July 2020)
		formed tumor-specific CD8+ T cells may undergo homeostatic expansion following mild lymphopenia [202,203]. Although a reduced lymphocyte repertoire and low CD4+ count post-thiotepa may impair in the short-term the de novo generation of tumor-specific T cells, at this point the relative clinical importance of these two thiotepa associated effects on T cells remains unclear in the context of Temferon treatment for GBM. Clinical safety with the combination of thiotepa and busulfan is well established not only in the field of haploidentical HSCT[199–201] but also historically for cerebral lymphoma[181–183], where higher doses have been administered than those proposed for inclusion in this study.<br><br>***Cohorts 4 and 5 will,*** ~~Should a potentially efficacious in vivo dose not be reached in dose cohort 3 (i.e. achieving a VCN≥0.7 in bone marrow at Day +30),~~ in the absence of ***2 or more dose-limiting toxicities in Cohort 3***, ~~a fourth dose cohort~~ employ~~ing~~ 2.0 x 10^6 Temferon cells/kg with a reduced number of non-manipulated CD34+ cells (2.0 x 10^6/kg) ~~will be considered~~ (i.e. 50% of the total dose of infused CD34+ cells). ***Cohort 4 will receive conditioning using carmustine and thiotepa, whereas Cohort 5 will receive conditioning using busulfan and thiotepa.***
Section 1.6.2.5	Risk of infusion reactions with carmustine included	Carmustine (BCNU) and thiotepa will be utilized as a conditioning regimen ***in Cohorts 1- 4*** prior to infusion of non-manipulated supporter cells and Temferon. Very common adverse reactions that may occur with a single 400 mg/m2 dose of BCNU include myelosuppression, ataxia, dizziness, headache, transient conjunctival flushing and blurred vision, retinal hemorrhages, hypotension (due to alcohol content of diluent), phlebitis, nausea and vomiting, and transient hyperpigmentation of the skin associated with accidental skin contact. ***Infusion reactions have also been reported.***
Section 1.6.2.5	Risks associated with busulfan included	***Busulfan and thiotepa will be utilized as a conditioning regimen in Cohort 5 prior to infusion of non-manipulated supporter cells and Temferon. Very common adverse reactions that may occur with busulfan include myelosuppression and infections, which reflect the immune status of patients during conditioning. Gastrointestinal symptoms such as nausea and vomiting, stomatitis, and diarrhea have also been reported very commonly. Hepatic veno-occlusive disease, acute respiratory distress syndrome and seizures have also been reported as serious adverse reactions. Impaired fertility commonly occurs after use of busulfan.***

DocuSign Envelope ID: A0702825-E0BD-46DB-A7D6-FEF901E11877

Location	Rationale	New (Version 4.0 of 17th July 2020)
		Further information for both BCNU, and thiotepa and busulfan can be found in the respective Summary of Product Characteristics included in the SOM.
Synopsis, Section 2.1	Purpose of addition of 2 cohorts (4 and 5) included in the primary objectives	In Part A, the primary objective is to evaluate the safety and tolerability of 3 escalating doses of Temferon in up to 9 patients with GBM who have an unmethylated MGMT promoter, following first line radiotherapy. ***Two additional cohorts will each include 3 patients, and will evaluate the safety, tolerability and long-term hematologic engraftment after 2 different conditioning regimens, followed by Temferon Dose 3 when 2x10^6 non-manipulated CD34+cells/kg are co-infused.***
Synopsis, Section 2.4	Secondary endpoints updated to achieve consistency with the additional conditioning regimen included	• The proportion of patients achieving hematologic recovery by day +30 following ~~BCNU and thiotepa administration from~~ the administration of ~~un-~~*non*-manipulated autologous HSPC and Temferon. Hematologic recovery is defined as the first of at least 3 consecutive days with a neutrophil count >0.5 x 10^9/L and platelet count >20 x 10^9/L.<br>• ***Assess the tolerability and safety of two conditioning regimens (BCNU and thiotepa, or busulfan and thiotepa), up to Day +30 following administration of Temferon, in patients with GBM.***<br>• Evaluate persistent transduced myeloid cells in PB as assessed by VCN. Persistence is defined as a VCN above the level of detection evident for at least 12 weeks. ***Persistence according to the type of conditioning regimen used, will also be evaluated.***
Synopsis, Sections 3.1, 4.1, 5.2, 5.3, 5.4.2.4, 5.4.2.5,	Inclusion of 3rd clinical center (Policlinico Gemelli). Changes in numerous sections to ensure consistency and clarity	This is a non-randomized, open label, ~~two-center~~ **multicenter**, phase I/IIa, therapeutic-exploratory, dose-escalation, prospective study…….<br>The study will recruit and follow-up patients at a specialist neurosurgical and neuro-oncology units ~~(Istituto Neurologico Carlo Besta, INCB) in Milan,~~ Italy. Administration of Temferon and hematological follow up will take place at ~~a~~ specialist hematology and bone marrow transplantation units ~~at OSR~~ in ~~Milan,~~ Italy.<br><br>Potentially eligible patients will be identified ~~at INCB~~ immediately after surgical resection of GBM once the MGMT promoter methylator status is known. Once written, informed consent is obtained, and screening procedures have been completed, harvesting of HSPCs will occur ~~at OSR~~. A standard of care regimen of radiotherapy lasting approximately

DocuSign Envelope ID: A0702825-E0BD-46DB-A7D6-FEF901E11877

Location	Rationale	New (Version 4.0 of 17th July 2020)
		6 weeks, will then take place. During this time, Temferon manufacturing will occur [redacted] Following completion of radiotherapy, patients will be admitted to ~~OSR~~ ***the bone marrow transplant unit*** for receipt of a conditioning regimen which ***in Cohort 1-4*** consist~~ings~~ of BCNU and thiotepa, ***and in Cohort 5 consists of busulfan and thiotepa (the choice of conditioning for Part B will be determined following completion of Part A)***. This will be followed by administration of non-manipulated HSPCs and Temferon. In-patient monitoring ***on the bone marrow transplant unit*** ~~at OSR~~ will occur until hematological recovery occurs. Thereafter, regular follow-up of patients will occur up to 2 years (+720 days) with the majority of assessments and procedures ~~performed at INCB~~ ***being neuro-oncological and with some hematological*** assessments/procedures ~~at OSR~~.<br><br>Approximately 21 patients will be treated and followed up at two centers in Milan ***and a center in Rome***. INCB is a specialist referral center for GBM in Italy and therefore patients may be resident outside the Milan or Lombardy region. ~~Patients will be assigned to study treatment only if they meet all the inclusion criteria and none of the exclusion criteria.~~ ***OSR is the Italian leading center for rare disease gene therapy, and the OSR neurosurgical team may also identify patients with GBM. Policlinico Universitario Fondazione Agostino Gemelli (PG) in Rome has been selected as this center has both neurosurgical and transplant hematology expertise for this study. Patients will be assigned to study treatment only if they meet all the inclusion criteria and none of the exclusion criteria.***
Synopsis, Section 3.1, Section 3.2 Intervention scheme figure, Section 3.3, 5.1	Study design considerations for Cohort 4 and 5, and reduction of number of patients to include in Part B	In Part A of the study, ***the first*** 3 cohorts of 3 patients will receive escalating doses of Temferon. ***Cohorts 4 and 5, which will recruit and run in parallel, will each include 3 patients, and patients will receive the same dose of Temferon as in Cohort 3, but they will receive a lower dose of non-manipulated CD34+ cells compared to Cohorts 1 to 3. Furthermore, 2 different conditioning regimens will be utilized in Cohorts 4 (BCNU and thiotepa) and 5 (busulfan and thiotepa)***. On completion of Part A, ***the conditioning regimen, dose of non-manipulated CD34+ cells and*** a single dose of Temferon will be selected to be studied in up to a further ~~12~~ 6 patients in Part B. Criteria for study eligibility are the same for both Part A and Part B.<br><br>5. Temferon treatment phase: Once the Investigator receives confirmation that Temferon is available to administer to the patient, the conditioning regimen ~~of BCNU will be administered to the patient on Day -6, followed~~

DocuSign Envelope ID: A0702825-E0BD-46DB-A7D6-FEF901E11877

Location	Rationale	New (Version 4.0 of 17th July 2020)
		~~by thiotepa on Day -5~~ ***will be administered at Baseline (Cohorts 1 to 4: BCNU will be administered to the patient on Day -6, followed by thiotepa on Day -5; Cohort 5: Thiotepa on Day -5 (evening) and -4 (morning), busulfan on Day -4 (evening) to Day -2).*** Non-manipulated HSPCs and Temferon will be infused on Day 0
Synopsis, Section 3.4.3, Section 5.2	If insufficient CD34+ cells are obtained at the first leukapheresis for the backup, this may be repeated at the Investigator's discretion after the radiotherapy has completed	Patients will undergo HSPC mobilization with lenograstim and plerixafor and harvest by leukapheresis, as per institutional practice. The harvested cells will be selected for the CD34+ marker to enrich for HSPCs. The mobilization target is ≥10 x10^6 CD34+ cells/kg, of which 3-7 x10^6 CD34++cells/kg will be used for Temferon production, while a minimum of 3-4 x10^6 CD34+ cells/kg will be frozen as non-manipulated apheresis product. ***In patients who mobilise poorly additional leukapheresis may be permitted, at the discretion of the Investigator, after completion of radiotherapy should additional non-manipulated CD34+ HSPCs be required for co-administration or as a backup.*** If the target number of cells cannot be collected after at least 2 attempts at HSPC collection, then the patient may be withdrawn from the study.
Synopsis, Section 3.4.5	Description of the Baseline visit and how thiotepa and busulfan will be administered	At least 7 to 28 days following completion of radiotherapy, a number of assessments should be completed prior to administration of conditioning to ensure the patient is still eligible for the study. A time window of up to 14 days prior to ~~Day -6~~***Baseline visit (defined as the first day of conditioning)*** has been provided to ensure these assessments are performed (See Table 12 1).<br><br>Experimental treatment (~~Day -6~~ ***Baseline*** through Day 0) will consist of reduced intensity conditioning; ***Cohorts 1-4*** with BCNU (Day -6, 400mg/m2 IV) and thiotepa (Day -5, two doses of 5mg/kg IV, once in the morning and once 12 hours later); ***Cohort 5 with thiotepa (Day -5 evening, 5mg/kg IV, Day -4 morning, 5mg/kg IV), and busulfan (Day -4 evening, busulfan 3.2 mg/kg ideal body weight IV, with TDM performed to adjust, if necessary, the dose of busulfan to administer on Day -3 and/or, if required, on Day -2.***

DocuSign Envelope ID: A0702825-E0BD-46DB-A7D6-FEF901E11877

Location	Rationale	New (Version 4.0 of 17th July 2020)
Synopsis, Section 4.1.2	Exclusion of patients if they have a known hypersensitivity to busulfan	• Known hypersensitivity to carmustine (or any other nitrosurea), thiotepa, ***busulfan***, lenograstim, plerixafor, or any excipients used in these products.
Section 5.4.2.5	Instructions for preparation of Temferon updated to include requirements for a clinical center outside Milan	Preparation of Temferon for patient administration will be performed by trained personnel at ~~UTMO in the Ospedale San Raffaele~~ the clinical center's ***Bone Marrow Transplant Unit***. ***These units*** hold a JACIE accreditation for cellular therapy, and the ***units*** ~~is~~ ***are*** authorized for the manipulation of genetically modified material. Thawing and infusion of the non-manipulated HSPCs (not considered part of the DP, and therefore handled by the JACIE-certified ~~OSR~~ Cell Manipulation ***Laboratory at each center***) will be performed before Temferon infusion, according to internal standard operating procedures ~~(IOS-TMO 309)~~.<br><br>***Either*** on the day of administration ***(OSR only) or prior to initiation of conditioning (PG)***, Temferon, frozen in cryovials, will be transferred [redacted] to the ~~OSR Cell Manipulation Lab/TE~~ ***clinical center***, ~~in accordance with IOG 345 guidelines,~~ in a liquid nitrogen dry shipper. ***On arrival at the Bone Marrow Transplant Unit, unless thawing of Temferon is to occur immediately, storage of the cryovials should occur at ≤-65°C in an approved storage area for clinical grade cell and gene therapy.*** ~~Specifically, Temferon will be released to the Investigator or another member of the clinical research team designated by the Investigator, and then transferred in a dry shipper biohazard container to the clinical ward for injection, according to the OSR Stem Cells Program internal operating procedures.~~
Section 5.5 and subsections	Information on BCNU and thiotepa, and busulfan and thiotepa provided. In obese patients, the dose of both BCNU and thiotepa may be reduced and based on	See Section 5.5.1 and Section 5.5.2 for extensive new wording

DocuSign Envelope ID: A0702825-E0BD-46DB-A7D6-FEF901E11877

Location	Rationale	New (Version 4.0 of 17th July 2020)
	adjusted ideal body weight, according to institutional standard operating procedures. Detailed instructions provided on administration of busulfan and calculation of busulfan doses using therapeutic drug monitoring	
Section 5.6	Details provided on the dose and requirements for infusion of non-manipulated CD34+ cells	***On Day 0, at least 60 hours after completion of thiotepa infusion (Cohorts 1 to 3), patients will receive an infusion of non-manipulated stem cell support at a dose of 3x10⁶ CD34⁺ cells/kg.***<br><br>***For Cohort 4, on Day 0, at least 60 hours after completion of thiotepa infusion, patients will receive an infusion of non-manipulated stem cell support at a dose of 2x10⁶ CD34⁺ cells/kg which may be adjusted according to the Temferon VCN (Table 5-1). For Cohort 5, at least 36 hours after completion of busulfan infusion (and at least 60hr following the last thiotepa dose), patients will receive an infusion of non-manipulated stem cell support at a dose of 2x10⁶ CD34⁺ cells/kg which may be adjusted according to the Temferon VCN (Table 5-1).***

<table>
<tr><th>Location</th><th>Rationale</th><th>New (Version 4.0 of 17th July 2020)</th></tr>
<tr><td></td><td></td><td>Supporter non-manipulated $CD34^+$ cells for Cohorts 4 and 5 to be infused
<table>
<tr><th>Temferon VCN</th><th>Supporter $CD34^+$ cells (cells/kg)</th></tr>
<tr><td>< 0.4</td><td>$\geq 1.5 \times 10^6$</td></tr>
<tr><td>0.4-1.0</td><td>$\geq 2.0 \times 10^6$</td></tr>
<tr><td>1.1-1.5</td><td>$\geq 2.5 \times 10^6$</td></tr>
<tr><td>>1.5</td><td>$\geq 3.0 \times 10^6$</td></tr>
</table></td></tr>
<tr><td>Section 5.7.1</td><td>Table describing the dosing scheme for Temferon has been simplified to accommodate Cohort 4 and 5</td><td>Dosing scheme for Temferon
<table>
<tr><th></th><th></th><th>Temferon ($CD34^+$/kg)*</th></tr>
<tr><td>Cohort 1</td><td>Dose level 1</td><td>0.5×10^6</td></tr>
<tr><td>Cohort 2</td><td>Dose level 2</td><td>1×10^6</td></tr>
<tr><td>Cohort 3</td><td>Dose level 3</td><td>2×10^6</td></tr>
<tr><td>Cohorts 4 and 5</td><td>Dose level 3</td><td>2×10^6</td></tr>
<tr><td colspan="3">* Temferon dose adjustments will be performed according to the in-vitro VCN indicated in the release documents.</td></tr>
</table></td></tr>
<tr><td>Synopsis, Section 5.7.1.</td><td>DMC will agree conditioning regimen, dose of non-modified CD34+ cells and dose of Temferon for Part B</td><td>The conditioning regimen, dose of non-manipulated CD34+ support cells and dose of Temferon to be evaluated in Part B will be determined from data obtained in Part A.</td></tr>
</table>

Location	Rationale	New (Version 4.0 of 17th July 2020)
Section 5.2, 5.7.2	Cohort 4 and 5 assignment	***For Cohorts 4 and 5 which will run in parallel, alternate patients will be allocated to each cohort after eligibility criteria have been confirmed at Screening. For example, the first patient identified will enter Cohort 4 and the next will be allocated to Cohort 5.***<br><br>***Cohorts 4 and 5 will start dosing Temferon Dose Level 3 in parallel after the DMC has reviewed the safety and efficacy data within Cohort 3, which also evaluates Temferon Dose Level 3. Alternate patients will be allocated to Cohort 4 and 5 after eligibility criteria have been confirmed at Screening. For example, the first patient identified will enter Cohort 4 and the next will be allocated to Cohort 5. Within each cohort, the 30 day interval will continue between Temferon dosing of a patient and commencement of conditioning for the next patient. The DMC will review the safety and efficacy data for Cohorts 4 and 5 and make a recommendation about proceeding to Part B.***
Section 6.1, Appendix 1 SOE	Updated SOE to incorporate centers outside OSR and INCB, cohorts 4 and 5	The Schedule of Events (SOE) to be conducted during the study is provided in Section 12.1-Appendix 1, in Table 12-1 and Table 12-2 ***for patients recruited and managed at OSR/INCB and in Table 12-3 and Table 12-4 for patients recruited and managed at PG.***
Section 6.4	DMC to advise on progression to Cohorts 4 and 5 and to advise on dosing plan for Part B	The DMC will review cumulative safety and efficacy data. The primary role of the committee is to ensure that emerging safety issues are identified and communicated to the Sponsor to ensure that the safety of patients is maintained. In addition, the DMC will review data and make a recommendation regarding the safety of progressing to higher doses ***and additional cohorts. At completion of Part A, the DMC will make a recommendation regarding the conditioning regimen, dose of non-modified CD34+ supporter cells and dose of Temferon to take forward into Part B.***<br><br>***The dose escalation recommendation and minutes from the open session of the DMC meeting will be sent to the ethics committee, after each DMC meeting. In addition, a summary of the data reviewed by the DMC at completion of Part A, will be sent to the ethics committee before*** ~~commencement~~ ***commencing*** ~~ement~~ ***administration of Temferon in*** ~~of~~ Part B.

DocuSign Envelope ID: A0702825-E0BD-46DB-A7D6-FEF901E11877

Location	Rationale	New (Version 4.0 of 17th July 2020)
Section 7.4.1	Adverse events attributed to conditioning will be summarized	***Adverse events attributed to conditioning will also be summarized by the type of conditioning regimen received.***

TEM-GBM_001 Clinical Protocol Amendment 4.1
SUMMARY OF CHANGES

This protocol (Version 4.1 of 14th December 2021) replaces previous version (Version 4.0 of 17th July 2020).

This amendment includes several substantial, administrative changes to the protocol:

- Prof Gaetano Finocchiaro has been appointed as Coordinating Principal Investigator based at Ospedale San Raffaele, Milan
- Dr. Marica Eoli has been appointed as the new Principal Investigator for Istituto Neurologico Carlo Besta
- [redacted]

Location	Rationale	New (Version 4.1 of 14th December 2020)
Headers throughout the document, signature pages, page 2	Version update	***Amendment V4.1 of 14th December 2020*** ~~Amendment V4.0 of 17th July 2020~~
Protocol Contributors, page 3	Investigators update – Prof. Gaetano Finocchiaro	***Coordinating*** Principal investigator***, based at Ospedale San Raffaele,*** responsible for advising on the study design, eligibility criteria, neuro-oncology endpoints, and clinical monitoring from a neurological perspectiv*e*
Protocol Contributors, page 3	Investigators update – Dr. Marica Eoli	***Principal investigator at Istituto Neurologico Carlo Besta and responsible for identification and management of potentially eligible patients***
Throughout the document	CMO Name Change	[redacted]

TEM-GBM_001 Clinical Protocol Amendment 4.2
SUMMARY OF CHANGES

This protocol (Version 4.2 of 05th July 2021) replaces previous version (Version 4.1 of 14th December 2020).

This amendment includes several substantial, administrative and clarification changes to the protocol including:

- Change of the Medical Monitor
- Addition of 2 cohorts to Part A in order to continue dose escalation to Dose Level 4 (Cohort 6) and Dose Level 5 (Cohort 7), while administering the conditioning regimen as in Cohorts 1-4 (BCNU and thiotepa) and intravenous infusion of 2 x 10^6 non-modified $CD34^+$ cells/kg and Temferon Dose Level 4

Other administrative changes or corrections have also been performed, as detailed below.

(Note: added text is ***bold in italic***; deleted text is ~~strikethrough)~~

Location	Rationale	New (Version 4.2 of 05th July 2021)
Headers throughout the document, signature pages, page 2	Version update	***Amendment V4.2 of 05th July 2021*** ~~Amendment V4.1 of 14th December 2020~~
Medical Monitor, page 2	Medical Monitor update: [redacted]	[redacted]<br>***Medical Monitor***<br>***Genenta Science***<br>[redacted]
Protocol Contributor, page 3	Medical Monitor update: [redacted]	***Medical Monitor*** and responsible for protocol development including study design, eligibility criteria, endpoints, schedule of events and procedures, safety monitoring

Location	Rationale	New (Version 4.2 of 05th July 2021)
Declaration of Investigator, page 7	The Investigator Brochure version was updated	Version 4.0, dated 9th July 2021
Version History, page 8	The changes incorporated in v4.2 were listed	***Protocol amendment (Changes in medical monitor and study outline); See Summary of Changes Page 9***
TEM-GBM_001 Clinical Protocol Amendment 6	The changes incorporated in v4.2 were listed	This protocol (***Version 4.2 of 05th July 2021***) replaces previous version (***Version 4.1 of 14th December 2020***).<br>This amendment includes the following substantial, ~~administrative~~ changes to the protocol:<br>• ***Change of medical monitor*** [redacted]<br>• ***Addition of 2 cohorts, each of 3 patients, to Part A. Both cohorts will utilize the same conditioning regimen of BCNU and thiotepa and the infusion of 2 x 10^6 non-modified $CD34^+$ cells/kg. Patients in Cohort 6 will receive 3 x 10^6 transduced cells/kg, while patients in Cohort 7 will receive 4 x 10^6 transduced cells/kg..***
Synopsis, Objectives	The section was modified to incorporate the additional cohorts to Part A and Dose Levels 4 and 5	~~In Part A, t~~The primary objective ***of TEM-GBM_001 study*** is to evaluate the safety and tolerability of ***5*** escalating doses of Temferon, in up to ***21*** patients with GBM ***and*** an unmethylated MGMT promoter, following first line radiotherapy. ***Patients are assigned to 7 different cohorts, each including 3 patients. The <u>first 3 cohorts</u> studied Temferon dose escalation up to Dose Level 3 (2 x 10^6 CD34+ cells/kg). Cohorts 4 and 5 were infused with Temferon Dose Level 3, reducing the number of non-manipulated supporter cells to 2x10^6 CD34+ cells/kg and evaluating*** the safety, tolerability and long-term hematologic engraftment after 2 different conditioning regimens ***(BCNU & thiotepa vs Busulfan & thiothepa, respectively). Dose-limiting toxicities have not been observed in the 9 patients that were treated at Temferon Dose Level 3, while engraftment of genetically-engineered cells stabilised around the 10% mark, with little difference between the 2 conditioning regimens. Based on these observations and the possibility that higher engraftment may translate into a higher likelihood of therapeutic efficacy, the study will enrol two additional Temferon Dose Levels, both conditioned with the BCNU & Thiotepa regimen used for Cohort 1-4.***<br><br>***Cohorts 6 and 7 will recruit in sequence 3 patients each who will be administered the same conditioning regimen of BCNU and thiotepa and an infusion of 2 x 10^6 non-modified $CD34^+$ cells/kg. Patients in Cohort 6 will receive 3 x 10^6 transduced cells/kg (Dose Level 4), while patients in Cohort 7 will receive 4 x 10^6 transduced cells/kg (Dose Level 5).***<br><br>~~In Part B, the primary objective *will be* to evaluate the safety and tolerability of a single dose, as identified from Part A, of Temferon in 6 patients with GBM and unmethylated MGMT, following first line radiotherapy.~~

Location	Rationale	New (Version 4.2 of 05th July 2021)
Synopsis, Study Design	The section was modified to incorporate the additional cohorts to Part A and Dose Levels 4 and 5 and delete the reference to Part B	***Patients in Cohorts 6 and 7 will be administered the same conditioning regimen of BCNU and thiotepa and an infusion of 2 x 10^6 non-modified $CD34^+$ cells/kg. Patients in Cohort 6 will receive 3 x 10^6 transduced cells/kg (Dose Level 4), while patients in Cohort 7 will receive 4 x 10^6 transduced cells/kg (Dose Level 5). In-patient monitoring will occur until hematological recovery occurs.***<br><br>Escalating doses of Temferon, ***from Dose 1 to 5, were and will be administered to cohorts of 3 patients each as follows:***<br><br>• ***Dose 1 – Cohort 1 (Completed)***<br>• ***Dose 2 – Cohort 2 (Completed)***<br>• ***Dose 3 – Cohorts 3 and 4, 5 (Completed)***<br>• ***Dose 4 – Cohort 6***<br>• ***Dose 5- Cohort 7***<br><br>***Cohorts 1 to 3 recruited and ran sequentially, while*** Cohorts 4 and 5 recruited and ran in parallel. ***Cohorts 6 and 7 will recruit and run sequentially.***<br><br>***Cohorts 1 to 3 received 3 x 10^6 non-modified $CD34^+$ cells/kg, while Cohorts 4 and 5 received 2 x 10^6 non-modified $CD34^+$ cells/kg. Cohorts 6 and 7 will receive 2 x 10^6 non-modified $CD34^+$ cells/kg as Cohorts 4 and 5.***<br><br>***Patients in Cohorts 6 and 7 will receive intravenous infusion of 3.0 x 10^6 Temferon cells/kg ($CD34^+$/kg) and 4.0 x 10^6 Temferon cells/kg ($CD34^+$/kg), respectively.***<br><br>~~On completion of Part A, the conditioning regimen, dose of non-manipulated $CD34^+$ cells and a single dose of Temferon will be selected to be studied in the up to a further 6 patients in Part B of the study.~~<br><br>~~Criteria for study eligibility are the same for both Part A and Part B.~~
Synopsis, Study Phases	The section was modified to incorporate the additional cohorts	Cohorts 1 to 4 ***and Cohorts 6 and 7***: BCNU will be administered to the patient on Day -6, followed by thiotepa on Day -5;

Location	Rationale	New (Version 4.2 of 05th July 2021)
Synopsis, Exploratory Endpoints	One endpoint was better clarified	Immunohistochemistry characteristics of tumors at first surgery and following repeat surgery including proliferative and apoptotic index, type of vascularization, EGFR and EGFRvIII expression, evaluation of T cells and myeloid cells infiltrate ***including the presence of transduced Tie-2 expressing monocytes***, and presence/absence of IFN-α gene expression
Synopsis, Safety Assessments	The section was modified to incorporate the additional cohorts	Safety assessments of the conditioning regimens utilized (Cohorts 1-4 ***and Cohort 6-7*** thiotepa and BCNU, Cohorts 5 busulfan and thiotepa) will include ~~adverse events~~ ***AE*** reporting and routine clinical and laboratory surveillance performed during the admission to the bone marrow transplant unit for conditioning and administration of Temferon.
Synopsis, ATMP, Dosage, and Route of Administration	The section was modified to incorporate the additional cohorts and better describe the cohorts.	**HSPC mobilization**<br><br>**(…)** Mobilization target ***for Cohorts 6 and 7*** is ≥ ***12*** x 10^6 leukocytes/kg, of which a minimum of ***7*** x10^6 $CD34^+$ cells/kg will be used for Temferon production, while a minimum of ***5*** x 10^6 leukocytes/kg will be frozen as non-manipulated apheresis product.<br><br>**Experimental treatment** with conditioning and infusion of Temferon:<br><br>*Cohorts 1 – 4* ***and Cohort 6-7****:*<br><br>On Day -6, BCNU (carmustine) 400 mg/m^2 will be given by IV infusion over 1 hour.<br><br>On Day -5, thiotepa 5mg/kg will be given twice (once in the morning and once 12 hours later) as an IV infusion over 2 hours.<br><br>*Non-manipulated $CD34^+$ HSPCs and Temferon Infusion:*<br><br>On Day 0, ~~at least 60 hours after completion of thiotepa infusion (Cohorts 1 to 3),~~ patients ~~will~~ receive an infusion of non-manipulated stem cell support at a dose of 3x10^6 $CD34^+$ cells/kg.<br><br>• ***Cohorts 1 to 3 were administered, at least 60 hours after completion of thiotepa infusion, with a dose of 3x10^6 $CD34^+$ of non-manipulated cells/kg.***<br>• Cohort 4 ***was*** administered, at least 60 hours after completion of thiotepa infusion, with a dose of 2x10^6 $CD34^+$ of non-manipulated cells/kg.

<table>
<tr><th>Location</th><th>Rationale</th><th>New (Version 4.2 of 05th July 2021)</th></tr>
<tr><td></td><td></td><td>

- Cohort 5 ***was*** administered, at least 36 hours after completion of busulfan infusion, with a dose of 2x10^6 CD34$^+$ of non-manipulated cells/kg.
- ***Cohort 6 and 7 will be administered, at least 60 hours after completion of thiotepa infusion, with a dose of 2x10^6 CD34$^+$ of non-manipulated cells/kg.***

</td></tr>
<tr><td>Synopsis, ATMP, Dosage, and Route of Administration</td><td>The section was modified to incorporate Dose levels 4 and 5</td><td>

- ***Dose level 4 (Cohort 6): 3.0 x 10^6 CD34+ cells/kg body weight;***
- ***Dose level 5 (Cohort 7): 4.0 x 10^6 CD34+ cells/kg body weight.***

</td></tr>
<tr><td>Synopsis, ATMP, Dosage, and Route of Administration</td><td>The Table was modified to incorporate Dose Levels 4 and 5</td><td>
<table>
<tr><th>VCN</th><th>Dose Level 1 (cells/kg)</th><th>Dose Level 2 (cells/kg)</th><th>Dose Level 3 (cells/kg)</th><th>Dose Level 4 (cells/kg)</th><th>Dose Level 5 (cells/kg)</th></tr>
<tr><td>< 0.4</td><td>0.8 x10^6</td><td>1.5 x10^6</td><td>3.0 x10^6</td><td>≥3.0 x10^6</td><td>≥4.0 x10^6</td></tr>
<tr><td>0.4-1.0</td><td>0.5 x10^6</td><td>1.0 x10^6</td><td>2.0 x10^6</td><td>3.0 x10^6</td><td>4.0 x10^6</td></tr>
<tr><td>1.1-1.5</td><td>0.4 x10^6</td><td>0.8 x10^6</td><td>1.5 x10^6</td><td>2.3 x10^6</td><td>3.0 x10^6</td></tr>
<tr><td>>1.5</td><td>0.3 x10^6</td><td>0.5 x10^6</td><td>1.0 x10^6</td><td>1.5 x10^6</td><td>2.0 x10^6</td></tr>
</table>
</td></tr>
<tr><td>Synopsis, ATMP, Dosage, and Route of Administration</td><td>The section was modified to address adjustment in the supporter cell dose for Cohorts 6 and 7</td><td>

No adjustment in the supporter cell dose is required for Cohorts 6 and 7 as a minimum total CD34+ cell dose > 4x10^6/kg is guaranteed in all circumstances.

Should the patient not have enough Temferon cells available for his planned dose level, the maximum number of available cells, but not less than 1.5x10^6/kg, will be infused.

The Investigators will discuss with the Sponsor the possibility of replacing the patient in the original cohort.

</td></tr>
<tr><td>Synopsis, ATMP, Dosage, and Route of Administration</td><td>The section was modified to add Cohorts 6 and 7 and to provide topics to be decided for Part B</td><td>

Patients in Cohorts 6 and 7 will utilize the same conditioning regimen (BCNU and thiotepa) and will be administered with the same dose of unmodified cells as in Cohorts 4 and 5.

If the decision will be to progress to Part B, ~~the conditioning regimen,~~ the dose of Temferon to be evaluated will be determined from data obtained in Part A.

</td></tr>
</table>

Location	Rationale	New (Version 4.2 of 05th July 2021)
Synopsis, Sample Size	The sample size for Part A was modified. The number of patients for Part B was deleted.	Part A: up to ~~15~~***21*** patients (up to 3 patients per cohort)<br><br>Part B: ~~up 6 patients21~~ ***sample size will be defined once the data generated in Part A will be analysed***
Section 1.5 Dosage justification for Temferon	The section was modified to incorporate the additional cohorts to Part A and Doses Level 4 and 5	Page 64<br><br>Therefore the objectives for this phase I/IIa dose escalation study are to determine the safety and tolerability of ~~3~~***5*** escalating doses of Temferon in the context of conditioning regimens used in bone marrow transplantation and a fixed dose of non-manipulated HSPCs, and to assess the biological activity of Temferon.<br><br>Page 65<br><br>~~Once the safety and tolerability of increasing doses of Temferon has been assessed using carmustine and thiotepa as a conditioning regimen, a~~An alternative conditioning approach using busulfan, instead of carmustine, in combination with thiotepa ***was*** evaluated in Cohort 5. The aim ***was*** to evaluate whether conditioning with busulfan ***(Cohort 5)*** (instead of carmustine, ***Cohorts 1-4***) in combination with thiotepa may result in a more stable engraftment of Temferon cells that ultimately leads to a greater number and potentially longer term differentiation of transduced myeloid progenitors into IFN-α2 expressing TEMs that migrate into the residual tumor.<br><br>Page 66<br><br>As this is a first-in-man study for GBM, patients ***in Cohorts 1-3*** will receive stem cell support using $3x10^6$ non-manipulated $CD34^+$ cells/kg body weight transplanted before Temferon, sufficient to allow full hematologic recovery following the conditioning regimen with either carmustine and thiotepa, or busulfan and thiotepa.<br><br>Page 67<br><br>***Based on the hematologic recovery and the in vivo engraftment observed in patients of Cohorts 4 and 5, patients in Cohorts 6 and 7 will receive 3.0 x 10^6 Temferon cells/kg and 4.0 x 10^6 Temferon cells/kg, respectively, with the same number of non-manipulated CD34^+ cells (2.0 x 10^6/kg) (i.e. respectively the 60% and 67% of the total dose of infused CD34^+ cells). Considering that data from Cohorts 4 and 5 suggest that the two tested conditioning regimens are***

Location	Rationale	New (Version 4.2 of 05th July 2021)
		equivalent; patients treated with BCNU show better VCN results; and BCNU is a more appropriate drug for GBM; the conditioning regimen in both Cohorts 6 and 7 will be carmustine and thiotepa.
Section 1.6.2.5 Risks related to autologous HSPC collection, conditioning chemotherapy and autologous HSPC infusion	The section was modified to specify the conditioning regimens for Cohorts 6 and 7	Carmustine (BCNU) and thiotepa will be utilized as a conditioning regimen in Cohorts 1- 4 ***and 6-7*** prior to infusion of non-manipulated supporter cells and Temferon. (…)
Section 2.1 Primary Objectives	The section was modified to incorporate the additional cohorts to Part A and Dose Levels 4 and 5 and delete the number of patients for Part B	~~In Part A, t~~The primary objective of ***TEM-GBM*** is to evaluate the safety and tolerability of ***5*** escalating doses of Temferon in up to ***21*** patients with GBM who have an unmethylated MGMT promoter, following first line radiotherapy. ***Cohorts 1-3 Cohorts each included 3 patients and evaluated the safety, tolerability and long-term hematologic engraftment after a conditioning regimen with BCNU and thiotepa, followed by Temferon Dose Level 1-3. Cohorts 4 and 5,*** each included 3 patients and evaluated the safety, tolerability and long-term hematologic engraftment after 2 different conditioning regimens ***(BCNU and thiotepa and thiotea and busulfan),*** followed by Temferon Dose Level 3. ***Cohorts 6 and 7 will receive the same conditioning regimen as of Cohorts 1-4, BCNU and thiotepa, and the same dose of unmodified cells as of Cohorts 4 and 5, i.e. 2x10^6 non-manipulated CD34$^+$cells/kg. Patients in Cohort 6 will receive Temferon Dose Level 4, while patients in Cohort 7 will receive Dose Level 5.***<br><br>~~In Part B, the primary objective is to evaluate the safety and tolerability of a single dose, as identified from Part A, of Temferon in 6 patients with GBM and unmethylated MGMT, following first line radiotherapy.~~
Section 2.5 Exploratory Endpoints	One endpoint was better clarified	Immunohistochemistry characteristics of tumors at first surgery and following repeat surgery including proliferative and apoptotic index (Ki-67, Cleaved Caspase 3, Tunel assay), type of vascularization (CD31 or vWF), EGFR and EGFRvIII expression, evaluation of T cells and myeloid cells infiltrate ***including the presence of transduced Tie-2 expressing monocytes,*** and presence/absence of IFN gene expression.
Section 3.1 Study Design	The section was modified to	Page 84

Location	Rationale	New (Version 4.2 of 05th July 2021)
	incorporate the additional cohorts and delete the number of patients for Part B	***The conditioning regimen in Cohorts 6 and 7 will be the same as in Cohorts 1-4, with BCNU and thiotepa.***<br><br>Page 84<br><br>***Escalating doses of Temferon from Dose 1 to 5 were and will be administered to cohorts of 3 patients each as follows:***<br><br>• ***Dose 1 – Cohort 1 (Completed)***<br>• ***Dose 2 – Cohort 2 (Completed)***<br>• ***Dose 3 – Cohort 3 and 4, 5 (Completed)***<br>• ***Dose 4 – Cohort 6***<br>• ***Dose 5 - Cohort 7***<br><br>Page 85<br><br>Furthermore, ***patients in Cohort 5 received a*** different conditioning regimen, busulfan and thiotepa, ***compared to patients enrolled in Cohort 1-4***.<br><br>***Cohorts 6 and 7, which will recruit and run sequentially, will each include 3 patients. The conditioning regimen in Cohorts 6 and 7 will be the same as in Cohorts 1-4 (BCNU and thiotepa). The dose of Temferon in Cohort 6 will be 3 x 10^6 per Kg body weight, while the dose of Temferon in Cohort 7 will be 4 x 10^6 per Kg body weight.***<br><br>~~On completion of Part A, the conditioning regimen, dose of non manipulated $CD34^+$ cells, and a single dose of Temferon will be selected to be studied in Part B. Criteria for study eligibility are the same for both Part A and Part B.~~

<table>
<tr>
<td>Section 3.2 Intervention Scheme</td>
<td>The scheme was modified to incorporate the additional cohorts and Dose Levels 4 and 5</td>
<td>

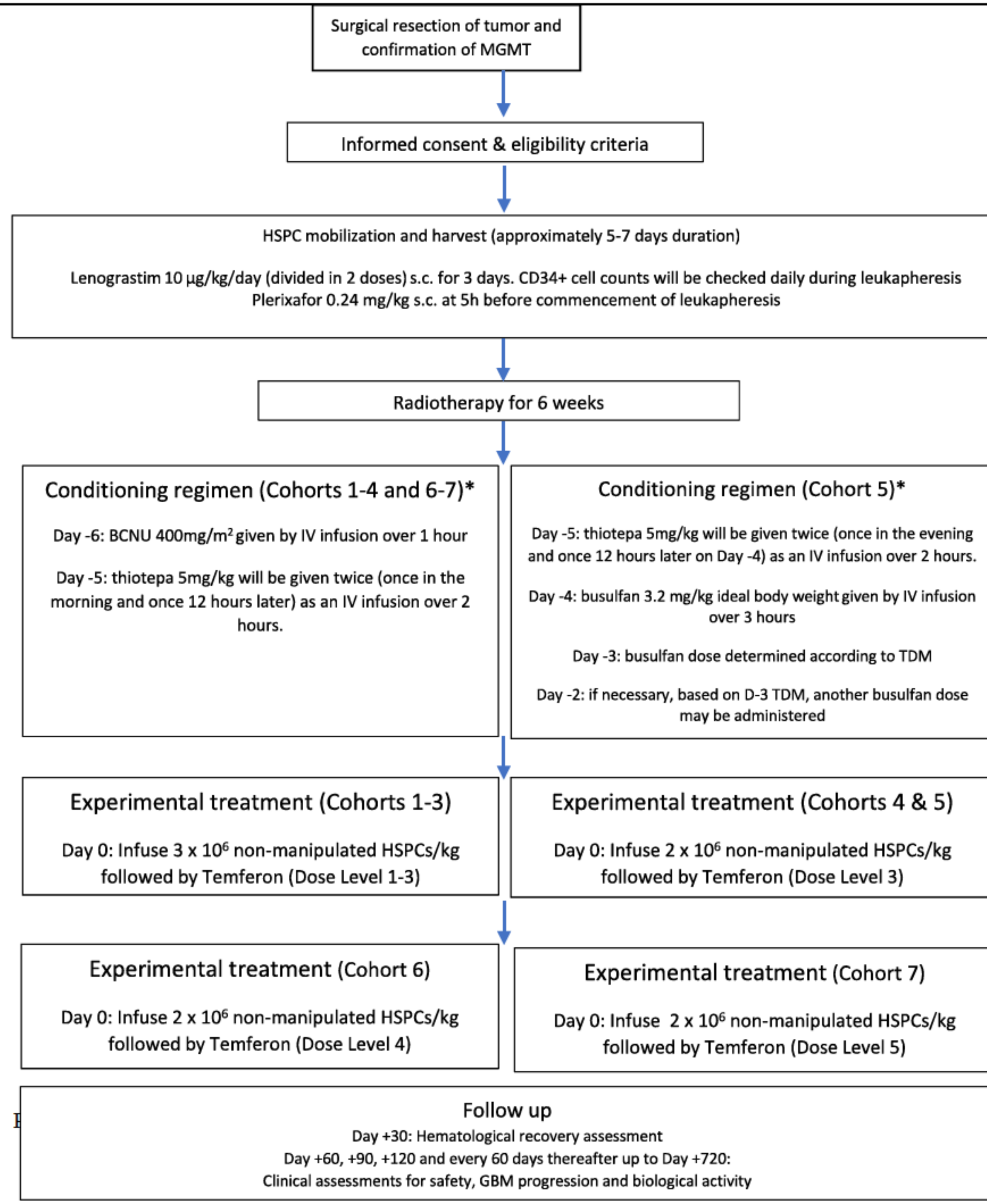

*Conditioning regimen, dose of non-manipulated cells and dose of Temferon for Part B will be discussed and agreed following completion of Part A with the DMC

</td>
</tr>
</table>

Location	Rationale	New (Version 4.2 of 05th July 2021)
Section 3.4 Study Phases	The section was modified to incorporate the additional cohorts	*Temferon treatment phase:* Once the Investigator receives confirmation that Temferon is available to administer to the patient, the conditioning regimen will be administered at Baseline (Cohorts 1 to 4 ***and Cohort 6 and 7***: BCNU will be administered to the patient on Day -6, followed by thiotepa on Day -5;
Section 3.4.3 HSPC harvest	The specifics for mobilization for Cohorts 6 and 7 were added	The mobilization target for Cohorts 6 and 7 is ≥ ***12*** x 10^6 $CD34^+$ cells/kg, of which ***7*** x 10^6 $CD34^+$ cells/kg will be used for Temferon production, while a minimum of ***5*** x 10^6 $CD34^+$ cells/kg will be frozen as non-manipulated apheresis product.
Section 3.4.5 Temferon treatment	The section was modified to incorporate the additional cohorts	Cohorts 1-4 ***and Cohort 6 and 7*** with BCNU (Day -6, 400mg/m^2 IV) and thiotepa (Day -5, two doses of 5mg/kg IV, once in the morning and once 12 hours later);
Section 5.1 Method of Assigning Patients to Treatment Groups	The section was modified to incorporate additional cohorts	***Cohorts 6 and 7 will run sequentially. For each cohort, a minimum of 30 days will separate dosing of the first patient and commencement of conditioning for the second patient within the cohort. Conditioning of the third patient will occur a minimum of 30 days after the second patient received Temferon.***<br><br>***Progression to Cohort 7 will be subjected to DMC recommendation.***
Section 5.2 HSPC mobilization and harvest	The specifics for mobilization for Cohorts 6 and 7 were added	The objective is to harvest ***for Cohorts 6 and 7*** a target dose of ≥***12*** x 10^6 $CD34^+$ cells/kg with as few leukapheresis sessions as possible during consecutive days. There should be sufficient cells available for production of Temferon (7 x 10^6 $CD34^+$ cells/kg), non-manipulated cells (***7*** x 10^6 $CD34^+$ cells/kg) and a backup (***2*** x 10^6 $CD34^+$ cells/kg).
Section 5.5.1 BCNU & Thiotepa	The title of the section was modified to incorporate additional cohorts	BCNU & Thiotepa (Cohorts 1 to 4 ***and Cohort 6 and 7***)

<table>
<tr><th>Location</th><th>Rationale</th><th>New (Version 4.2 of 05th July 2021)</th></tr>
<tr><td>Section 5.6 Infusion of non-manipulated, cryopreserved apheresis</td><td>The section was modified to incorporate additional cohorts</td><td>For Cohorts 4, 6 and 7, on Day 0, at least 60 hours after completion of thiotepa infusion, patients will receive an infusion of non-manipulated stem cell support at a dose of $2x10^6$ $CD34^+$ cells/kg which may be adjusted according to the Temferon VCN (Table 5-1). For Cohorts 5 and 7, at least 36 hours after completion of busulfan infusion (…)</td></tr>
<tr><td>Section 5.6 Infusion of non-manipulated, cryopreserved apheresis</td><td>Table 5 1 was deleted</td><td>
<table>
<tr><th>~~Temferon VCN~~</th><th>~~Supporter $CD34^+$ cells (cells/kg)~~</th></tr>
<tr><td>~~<0.4~~</td><td>~~≥ 1.5 x10^6~~</td></tr>
<tr><td>~~0.4-1.0~~</td><td>~~≥ 2.0 x10^6~~</td></tr>
<tr><td>~~1.1-1.5~~</td><td>~~≥ 2.5 x10^6~~</td></tr>
<tr><td>~~>1.5~~</td><td>~~≥ 3.0 x10^6~~</td></tr>
</table>
</td></tr>
<tr><td>Section 5.7.1 Dose Levels and Dose Adjustments, Tables 5-2 and 5-3</td><td>The tables were modified to incorporate Dose Levels 4 and 5</td><td>
<table>
<tr><th>Cohort</th><th>Dose Level</th><th>Temferon ($CD34^+$/kg)*</th></tr>
<tr><td>Cohort 1</td><td>1</td><td>0.5 x10^6</td></tr>
<tr><td>Cohort 2</td><td>2</td><td>1.0 x10^6</td></tr>
<tr><td>Cohort 3</td><td>3</td><td>2.0 x10^6</td></tr>
<tr><td>Cohorts 4 and 5</td><td>3</td><td>2.0 x10^6</td></tr>
<tr><td>Cohort 6</td><td>4</td><td>3.0 x10^6</td></tr>
<tr><td>Cohort 7</td><td>5</td><td>4.0 x10^6</td></tr>
<tr><td colspan="3">* Temferon dose adjustments will be performed according to the in-vitro VCN indicated in the release documents.</td></tr>
</table>
</td></tr>
</table>

<table>
<tr><th>Location</th><th>Rationale</th><th>New (Version 4.2 of 05th July 2021)</th></tr>
<tr><td></td><td></td><td>
Dosing scheme for Temferon

Temferon dose adjustments will be performed according to VCN
<table>
<tr><th>VCN</th><th>Dose Level 1 (cells/kg)</th><th>Dose Level 2 (cells/kg)</th><th>Dose Level 3 (cells/kg)</th><th>Dose Level 4 (cells/kg)</th><th>Dose Level 5 (cells/kg)</th></tr>
<tr><td>< 0.4</td><td>0.8 x10^6</td><td>1.5 x10^6</td><td>3.0 x10^6</td><td>≥3.0 x10^6</td><td>≥4.0 x10^6</td></tr>
<tr><td>0.4-1.0</td><td>0.5 x10^6</td><td>1.0 x10^6</td><td>2.0 x10^6</td><td>3.0 x10^6</td><td>4.0 x10^6</td></tr>
<tr><td>1.1-1.5</td><td>0.4 x10^6</td><td>0.8 x10^6</td><td>1.5 x10^6</td><td>2.3 x10^6</td><td>3.0 x10^6</td></tr>
<tr><td>>1.5</td><td>0.3 x10^6</td><td>0.5 x10^6</td><td>1.0 x10^6</td><td>1.5 x10^6</td><td>2.0 x10^6</td></tr>
</table>
</td></tr>
<tr><td>Section 5.7.1 Dose Levels and Dose Adjustments</td><td>The section was modified to address the adjustment in supporter cells for Cohorts 6 and 7</td><td>No adjustment in the supporter cell dose is required for Cohorts 6 and 7 as a minimum total CD34+ cell dose > 4x10^6/kg is guaranteed in all circumstances.

Should the patient not have enough Temferon cells available for his planned dose level, the maximum number of available cells, but not less than 1.5x10^6/kg, will be infused.

<u>The Investigators will discuss with the Sponsor the possibility of replacing the patient in the original cohort.</u></td></tr>
<tr><td>Section 5.7.2 Cohort Assignment</td><td>The section was modified to incorporate additional cohorts</td><td>Patients were alternately allocated in Cohorts 4 and 5 and were dosed in parallel with Temferon Dose Level 3 after the DMC reviewed the safety and efficacy data of Cohort 1-3.

Patients in Cohort 6 will be administered with Temferon Dose Level 4 after the DMC has reviewed the safety and efficacy data of Cohorts 1-5; patients in Cohort 7 will be administered with Temferon Dose Level 5 after the DMC has reviewed the safety and efficacy data of Cohorts 1-6; Within Cohorts 6 and 7, the 30- day interval will continue between Temferon dosing of a patient and commencement of conditioning for the next patient.

The DMC will review the safety and efficacy data for Cohort 7 and make a recommendation whether to proceed to Part B.</td></tr>
<tr><td>Section 6.2.4.5 Clonal tracking of Temferon graft over time</td><td>The section was modified to add Day+180 timepoint</td><td>Integration site analysis on distinct hematopoietic lineages will be performed on peripheral blood and/or bone marrow at Screening (negative control), Day +30, 90, +180, 360 and 720, and yearly thereafter (…)</td></tr>
</table>

Location	Rationale	New (Version 4.2 of 05th July 2021)
in different hematopoietic lineages		
Section 7.4.2 Other Analyses	The conditioning regimens were better indicated	The proportion of patients will be summarized by cohort and Temferon dose, achieving hematologic recovery by Day +30 following BCNU and thiotepa or ***thiotepa and*** busulfan administration from administration of non-manipulated autologous HSPC and Temferon.
Section 12.1 Appendix: Schedule of Events	The Schedule of Events was modified to incorporate the additional cohorts and the Day+180 timepoint for Integration Site Analysis. The tables were divided in 2 to facilitate the reading.	

TEM-GBM_001 Clinical Protocol Amendment 5.0
SUMMARY OF CHANGES

This protocol (Version 5.0 of 21st October 2022) replaces previous version (Version 4.2 of 5th July 2021).

This amendment includes the following substantial changes to the protocol:

- Two cohorts to use busulfan monotherapy, each of 3 patients, in Part A. Both cohorts will utilize the same conditioning regimen of busulfan and the infusion of 3 x 10^6 non-modified $CD34^+$ cells/kg. Patients in Cohort 7 will receive 3 x 10^6 transduced cells/kg, while patients in Cohort 8 will receive 4 x 10^6 transduced cells/kg. Following completion of the DMC review of data from both Cohorts 7 and 8, the DMC will advise on dose escalation (after Cohort 7) or dosing of an additional 3 subjects.

Other administrative changes or corrections have also been performed, as detailed below.

(Note: added text is ***bold in italic***; deleted text is ~~strikethrough)~~

Location	Rationale	New (Version 5.0 of 21st October 2022)
Headers throughout the document, signature pages, page 2	Version update	***Amendment V5.0 of 21st October 2022*** ~~Amendment V4.2 of 5th July 2021~~
Medical Monitor, page 2	Medical Monitor update: [redacted]	[redacted]<br>Medical Monitor<br>Genenta Science<br>[redacted]
Protocol Contributor, page 3	Medical Monitor update: [redacted]	***Medical Monitor*** and responsible for protocol development including study design, eligibility criteria, endpoints, schedule of events and procedures, safety monitoring
SAE reporting, page 2<br><br>Section 6.3.3, page 131	Change in name of PV vendor	[redacted]

Location	Rationale	New (Version 5.0 of 21st October 2022)
Synopsis: Objectives<br><br>Section 2.1, page 82	Change in number of subjects in study to accommodate for additional cohort 8, rationale for change in conditioning to busulfan monotherapy	The primary objective of TEM-GBM_001 study is to evaluate the safety and tolerability of 5 escalating doses of Temferon, in up to ~~21~~ ***27*** patients with GBM and an unmethylated MGMT promoter, following first line radiotherapy. Patients are assigned to ~~7~~ ***8*** different cohorts, each including 3 patients.<br><br>***Cohort 6 patients received Temferon Dose Level 4 (3 x 10^6 CD34+ cells/kg) and non-manipulated supporter cells to 2x10^6 CD34+ cells/kg which were administered after BCNU and thiotepa conditioning.***<br><br>Dose-limiting toxicities have not been observed in the ~~9~~ ***15*** patients that were treated ~~at~~ ***up to*** Temferon Dose Level ~~3~~***4***, ~~, while engraftment of genetically-engineered cells stabilised around the 10% mark, with little difference between the 2 conditioning regimens. Based on these observations and the possibility that higher engraftment may translate into a higher likelihood of therapeutic efficacy, the study will enrol two additional Temferon Dose Levels, both conditioned with the BCNU & Thiotepa regimen used for Cohort 1-4.~~ ***However, serious adverse events (CTCAE≥ Grade 3) attributed to the conditioning regimen have occurred associated with hematologic complications (febrile neutropenia, secondary graft failure), venous thromboembolism, pneumonia and other infections. The majority of patients have also received dexamethasone during the post-transplant period as a treatment for radiotherapy associated cerebral edema. The combination of glioblastoma, brain irradiation, conditioning chemotherapy and dexamethasone has therefore contributed to complications resulting from immunosuppression.***<br><br>***In order to mitigate these recognised risks, the conditioning chemotherapy regimen will be modified in two additional cohorts to utilise busulfan monotherapy (total busulfan AUC target 60 mg·h/L). Thiotepa will be omitted. In addition, patients utilizing dexamethasone >4mg qd within 20 days of Temferon administration will undergo frequent medical review over 3 weeks to reduce dexamethasone requirements to ≤4mg qd.***<br><br>Cohorts ~~6~~ ***7*** and ~~7~~ ***8*** will recruit 3 patients each who will be administered ~~the same conditioning regimen of BCNU and thiotepa~~ ***busulfan*** and an infusion of ~~2~~ ***3*** x 10^6 non-modified CD34+ cells/kg. Patients in Cohort ~~6~~ ***7*** will receive 3 x 10^6 transduced cells/kg (Dose Level 4), while patients in Cohort ~~7~~ ***8*** will receive 4 x 10^6 transduced cells/kg (Dose Level 5).

Location	Rationale	New (Version 5.0 of 21st October 2022)
		Following completion of dosing in Cohorts 7 and 8 and review of data by the DMC, an additional 3 patients will receive Temferon at a dose selected from either Cohort 7 or 8 in order to provide additional safety data before progression to Part B of the study.
Synopsis: Study design<br><br>Section 3.1, page 86-87	Description of Cohorts 7 and 8 and need for additional 3 subjects to confirm safety in final cohort	This is a non-randomized, open label, multicenter, phase I/IIa, therapeutic-exploratory, dose-escalation, prospective study, involving a single injection of Temferon, an investigational ATMP consisting of autologous CD34+-enriched HSPCs exposed to transduction with a 3rd generation, vesicular stomatitis virus G (VSV-G) pseudo-typed lentiviral vector driving myeloid-specific IFN-α2 expression, which will be administered to up to 2~~1~~*7* patients affected by GBM who have an unmethylated MGMT promoter.<br><br>***Patients in Cohort 6 received BCNU and thiotepa conditioning and an infusion of 2 x 10^6 non-modified CD34+ cells/kg and 3 x 10^6 transduced cells/kg (Dose Level 4).*** Patients in Cohorts ~~6~~ *7* and ~~7~~ ***8 will receive busulfan monotherapy as conditioning and an infusion of 3 x 10^6 non-modified CD34+ cells/kg***. ***Patients in Cohort 7 will receive*** ~~receive BCNU and thiotepa conditioning and an infusion of 2 x 106 non-modified CD34+ cells/kg. Patients in Cohort 6 will receive~~ 3 x 10^6 transduced cells/kg (Dose Level 4), while patients in Cohort *8*~~7~~ will receive 4 x 10^6 transduced cells/kg (Dose Level 5).<br><br>Escalating doses of Temferon, from Dose 1 to 5, were and will be administered to cohorts of 3 patients each as follows:<br>• Dose 1 – Cohort 1 (Completed)<br>• Dose 2 – Cohort 2 (Completed)<br>• Dose 3 – Cohorts 3 and 4, 5 (Completed)<br>• Dose 4 – Cohort 6 ***(Completed), and 7***<br>• Dose 5 -- Cohort ~~7~~*8*<br><br>Cohorts 1 to 3 recruited and ran sequentially, while Cohorts 4 and 5 recruited and ran in parallel. Cohorts 6 ***ran sequentially after Cohorts 4 and 5.*** ***Cohorts*** ~~and~~ 7 and 8 will recruit and run sequentially.<br><br>Cohorts 6 ~~and 7 will~~ received 2 x 10^6 non-modified CD34+ cells/kg. **Cohorts 7 and 8 will receive 3 x 10^6 non-modified CD34+ cells/kg** as in Cohorts ~~4 and 5~~1 to 3.

Location	Rationale	New (Version 5.0 of 21st October 2022)
		Patients in Cohorts ~~6~~7 and 8~~7~~ will receive intravenous infusion of 3.0 x 10^6 Temferon cells/kg (CD34+/kg) and 4.0 x 10^6 Temferon cells/kg (CD34+/kg), respectively.<br><br>***Following completion of dosing in Cohorts 7 and 8 and review of data by the DMC, an additional 3 patients will receive Temferon at a dose selected from either Cohort 7 or 8 in order to provide additional safety data before progression to Part B of the study.***
Synopsis: Study phases, Safety Assessments<br><br>Section 3.4, page 89	Busulfan monotherapy as conditioning for Cohorts 7 and 8	5. Temferon treatment phase: Once the Investigator receives confirmation that Temferon is available to administer to the patient, the conditioning regimen will be administered at Baseline (Cohorts 1 to 4 and Cohorts ~~6~~ ~~and 7~~: BCNU will be administered to the patient on Day -6, followed by thiotepa on Day -5; Cohort 5: Thiotepa on Day -5 and -4, Busulfan Day -4 to Day-2; ***Cohorts 7 and 8: Busulfan Day -4 to Day-2***). Non-manipulated HSPCs and Temferon will be infused on Day 0.
Synopsis: secondary endpoints. Safety (Part A only)<br><br>Section 2.4, page 83	Include busulfan monotherapy as one of the conditioning regimens and extend time of interest for endpoint to Day +45 days	• Assess the tolerability and safety of ~~two~~***three*** conditioning regimens (BCNU and thiotepa, ~~or~~ busulfan and thiotepa, ***or busulfan monotherapy***), up to Day +~~3~~**45** following administration of Temferon, in patients with GBM.
Synopsis: exploratory endpoints	Make synopsis consistent with text in Section 2.5	• Characterization of IFN-α gene signatures in bone marrow ***and peripheral blood*** over time.
Synopsis: Safety assessments	Emphasise need to record all haematology data collected after Temferon infusion until hematologic engraftment in the CRF	Complete blood counts will be performed daily after Temferon infusion until hematological engraftment ***and these data will be recorded in the eCRF.***

Location	Rationale	New (Version 5.0 of 21st October 2022)
Synopsis: ATMP, Dosage and Route of Administration<br><br>Section 3.4.3<br><br>Section 5.2, page 100	Mobilization target for Cohorts 7 and 8	Mobilization target for Cohorts ~~6,~~ ***7 and 8*** ~~and 7~~ is ≥ 12 x 10^6 leukocytes/kg, of which a minimum of 7 x10^6 CD34+ cells/kg will be used for Temferon production, while a minimum of ~~5~~ ***6*** x 10^6 leukocytes/kg will be frozen as non-manipulated apheresis product.<br><br>***Not less than 6x10^6 non-manipulated CD34+ cells/kg should be destined for co-infusion and backup, to account for cell losses (e.g., repeated QC testing) and variabilities in cell counts/volume measurements between the CMO and the clinical cell manipulation lab.***
Synopsis: ATMP, Dosage and Route of Administration<br><br>Section 3.4.5 Page 91-92	Management of dexamethasone prior to conditioning	Administration of Temferon will only occur once radiotherapy has completed ***and providing the daily requirements for dexamethasone (or another corticosteroid) is less than or equal to 4mg daily. In the event that dexamethasone (or equivalent) >4mg qd is required, the Investigator will have a period of 3 weeks from the time of receipt of the post-radiotherapy/baseline brain MRI images to control cerebral edema or manage clinical symptoms with alternative approved therapies (bevacizumab is prohibited) with the aim of reducing corticosteroid requirements to a dexamethasone (or equivalent) dose ≤4mg qd. In the event that this dose target cannot be reached, the Investigators will review the case with the Sponsor in order to discuss and agree the rationale for continuation of the patient in the study.***
Synopsis: ATMP, Dosage and Route of Administration	Therapeutic drug monitoring in Cohorts 7 and 8	***Cohort 7 and 8:***<br>***On Day -4, in the evening, busulfan 3.2 mg/kg ideal body weight will be given by IV infusion over 3 hours. Therapeutic drug monitoring of busulfan will be performed as described above for Cohort 5. Pharmacokinetic blood sampling will occur pre-dosing, 5 minutes after the end of busulfan infusion, and +2 hours, +4 hours and +6 hours after the end of the busulfan infusion. The pharmacokinetic busulfan data will be calculated. The total busulfan AUC target is 60 mg·h/L. The busulfan dose on Day -3 may be adjusted and/or an additional dose of busulfan may be infused on Day -2 in order to ensure drug exposure within the target range***
Synopsis: ATMP, Dosage and Route of Administration	Dose of non-manipulated CD34+	• ***Cohorts 7 and 8 will be administered, at least 36 hours after completion of busulfan infusion, with a dose of 3x10^6 CD34+ of non-manipulated cells/kg***

Location	Rationale	New (Version 5.0 of 21st October 2022)
Section 5.6, page 108	cells to infuse in Cohorts 7 and 8	No adjustment in the supporter cell dose is required for Cohorts 6, ***7 or 8*** ~~and 7~~ as a minimum total CD34+ cell dose > 4x10^6/kg is guaranteed in all circumstances.
Synopsis: ATMP, Dosage and Route of Administration	Busulfan monotherapy and addition of 3 patients once completion of DMC review of data for Cohorts 7/8 occurs	***Patients in Cohorts 7 and 8 will use busulfan monotherapy as conditioning and will receive the same dose of unmodified cells as in Cohorts 1-3.***<br><br>***Prior to progression to Part B, an additional 3 patients will receive busulfan monotherapy as conditioning, the same dose of unmodified cells as in Cohorts 1-3, 7 and 8, and a dose of Temferon selected from Cohort 7 or Cohort 8.***
Synopsis: Sample size	Clarity on number of patients and cohorts in Part A	Part A: up to ~~21~~ ***27*** patients (~~7~~ 8 cohorts, up to 3 patients per cohort, ***and an additional 3 patients prior to progression to Part B***)
Section 1.3.5, page 63	Updated preclinical data from orthotopic GBM model	***Upon orthotopic transplantation into C57Bl/6, mGB2 cells gave rise to high-grade gliomas that faithfully recapitulated the histopathological features, invasiveness and immune cell infiltration characteristic of certain types of human glioblastoma[159]. In this mGB2 mouse model, IFN-a delivery by TEMs delayed or suppressed GBM growth, whereas in the GL261 preclinical model, low circulating amounts of IFN-a were in sufficient to elicit antitumor responses. The anti-tumor activity was exerted through the reprogramming of the myeloid compartment associated with a shift in the M2 to M1 macrophage balance in favor of pro-inflammatory macrophages (M1) with the IFN-a mediated angiogenesis inhibition. Furthermore, there was activation of the adaptive and innate immune response as showed by the reduced percentage of exhausted CD8+ T cells, the immunoglobulin switch from IgM to IgG, the activation of tumor-infiltrating DCs, a positive enrichment of M1 genes and negative enrichment for M2 genes[160].***
Section 1.5, page 68	Explanation for change in study design, steroid dose prior to conditioning and busulfan monotherapy	Dose-limiting toxicities have not been observed in the 9 patients that were treated at Temferon Dose Level 3, while engraftment of genetically-engineered cells stabilised around the 10% mark, with little difference between the 2 conditioning regimens. Based on these observations and the possibility that higher engraftment may translate into a higher likelihood of therapeutic efficacy, the study ~~will~~ ***enrolled*** an ~~two~~ additional Temferon Dose Level~~s~~, ~~both~~ conditioned with the BCNU & Thiotepa regimen used for Cohorts 1-4. Patients in Cohorts 6 ~~and 7 will~~ received 3.0 x 10^6 Temferon cells/kg

Location	Rationale	New (Version 5.0 of 21st October 2022)
		(Dose Level 4) ~~and 4.0 x 106 Temferon cells/kg (Dose Level 5), respectively~~, with the same number of non-manipulated CD34+ cells (2.0 x 106/kg) (i.e. ~~respectively the~~ 60% ~~and 67%~~ of the total dose of infused CD34+ cells). ***No dose limiting toxicity was observed in this Cohort. However, review of safety data from all 6 cohorts showed that serious adverse events (CTCAE≥ Grade 3) attributed to the conditioning regimen have occurred associated with hematologic complications (e.g. febrile neutropenia, secondary graft failure), venous thromboembolism, pneumonia and other infections. The majority of patients have also received dexamethasone during the post-transplant period as a treatment for radiotherapy associated cerebral edema. The combination of glioblastoma, brain irradiation, conditioning chemotherapy and dexamethasone has therefore contributed to complications resulting from immunosuppression.***<br><br>***In order to mitigate these recognised risks, the conditioning chemotherapy regimen will be modified in two additional cohorts (Cohorts 7 and 8) to utilise busulfan monotherapy (total busulfan AUC target 60 mg·h/L). Thiotepa will be omitted. In addition, patients utilizing dexamethasone >4mg qd within 20 days of Temferon administration will undergo frequent medical review over 3 weeks to reduce dexamethasone requirements to ≤4mg qd.***<br><br>***Cohorts 7 and 8 will recruit 3 patients each who will be administered busulfan and an infusion of 3 x 10^6 non-modified CD34+ cells/kg. Patients in Cohort 7 will receive 3 x 10^6 transduced cells/kg (Dose Level 4), while patients in Cohort 8 will receive 4 x 10^6 transduced cells/kg (Dose Level 5) (i.e. 50% and 57%, respectively, of the total dose of infused CD34+ cells).***
Section 1.6.2.5, page 76	Explanation for change in study design, steroid dose prior to conditioning and busulfan monotherapy	***Following review of safety data from Cohorts 1-6, serious adverse events (CTCAE≥ Grade 3) attributed to the conditioning regimen have occurred associated with hematologic complications (febrile neutropenia, secondary graft failure), venous thromboembolism, pneumonia and other infections. The majority of patients have also received dexamethasone during the post-transplant period as a treatment for radiotherapy associated cerebral edema. The combination of glioblastoma, brain irradiation, conditioning chemotherapy and dexamethasone has therefore contributed to complications resulting from immunosuppression.***<br><br>***In order to mitigate these recognised risks, and following discussion and agreement with the Data Monitoring Committee (DMC), the conditioning chemotherapy regimen will be modified in two additional cohorts (Cohorts 7 and 8) to utilise busulfan monotherapy (total busulfan AUC target 60***

Location	Rationale	New (Version 5.0 of 21st October 2022)
		mg·h/L). Thiotepa will be omitted. In addition, patients utilizing dexamethasone >4mg qd within 20 days of Temferon administration will undergo frequent medical review over 3 weeks to reduce dexamethasone requirements to ≤4mg qd.
Section 3.2. Intervention Scheme, page 88	Updated conditioning (busulfan monotherapy) for use in Cohorts 7 and 8	See page 88 for updated figure
Section 3.4.6. page 92<br><br>Section 5.11.1, page 114<br><br>Sction 6.2.2.1, page 116	Emphasise need for daily blood count from D+1 to day of discharge from transplant unit	***It is essential that daily complete blood counts are performed from D+1 until the day of discharge from the transplant unit following ASCT and these data are recorded in the eCRF.***
Section 4.1, page 94	Patient number updated	Approximately ~~21~~ ***27*** patients will be treated in Part A and followed up at two centers in Milan (OSR and INCB). A center ~~and a center~~ in Rome ~~(~~**will participate for** ~~the~~ Part B of the study~~, if any~~).
Section 5.1, page 100	Details on Cohort 7 and 8	Cohorts 6-***8*** ~~and 7~~ will run sequentially<br><br>Progression ~~to Cohort 7~~ ***from Cohort 6 to 7, and from Cohort 7 to 8*** will be subjected to DMC recommendation. ***Following completion of dosing in Cohorts 7 and 8 and review of data by the DMC, an additional 3 patients will receive Temferon at a dose selected from either Cohort 7 or 8 in order to provide additional safety data before progression to Part B of the study. No staggering is foreseen in the additional 3 patients to be dosed.***

<table>
<tr><th>Location</th><th>Rationale</th><th>New (Version 5.0 of 21st October 2022)</th></tr>
<tr><td>Section 5.5, page 105</td><td>Addition of busulfan monotherapy as conditioning regimen</td><td>- BCNU & Thiotepa (Cohorts 1 to 4 and Cohort~~s~~ 6 ~~and 7~~),
- Busulfan & Thiotepa (Cohort 5)
- Busulfan monotherapy (Cohorts 7 and 8)</td></tr>
<tr><td>Section 5.5.2, page 106 - 108</td><td>Detailed instructions on busulfan infusion and Pk monitoring. Moved from previous section 5.5.1 to 5.5.2</td><td>Text unchanged except for:
If the decision will be to progress to Part B, ~~based on pharmacokinetic and clinical data, in the event that the combination of busulfan and thiotepa is selected for conditioning,~~ therapeutic busulfan drug monitoring may be omitted, and instead patients may instead receive a fixed-dose busulfan ~~+ thiotepa conditioning~~ regimen.</td></tr>
<tr><td>Table 5-1, page 109</td><td>Updated information for Cohorts 7 and 8 with corresponding dose levels</td><td>
<table>
<tr><th>Cohort</th><th>Dose Level</th><th>Temferon (CD34+/kg)*</th></tr>
<tr><td>Cohort 1</td><td>1</td><td>0.5 x10^6</td></tr>
<tr><td>Cohort 2</td><td>2</td><td>1.0 x10^6</td></tr>
<tr><td>Cohort 3</td><td>3</td><td>2.0 x10^6</td></tr>
<tr><td>Cohorts 4 and 5</td><td>3</td><td>2.0 x10^6</td></tr>
<tr><td>Cohort 6 and 7</td><td>4</td><td>3.0 x10^6</td></tr>
<tr><td>Cohort 8</td><td>5</td><td>4.0 x10^6</td></tr>
<tr><td colspan="3">* Temferon dose adjustments will be performed according to the in-vitro VCN indicated in the release documents.</td></tr>
</table>
</td></tr>
<tr><td>Section 5.7.2, page 111</td><td>Explanation for change to Cohort 7, addition of Cohort 8, and an additional 3 patients prior to Part B</td><td>Patients in Cohort 6 ~~will be~~ were administered with Temferon Dose Level 4. Following DMC review of safety and efficacy data from Cohorts 1-6 a recommendation was to include Cohort 7 and 8 ~~after the DMC has reviewed the safety and efficacy data of Cohorts 1-5; patients in Cohort 7 will be administered with Temferon Dose Level 5 after the DMC has reviewed the safety and efficacy data of Cohorts 1-6; Within Cohorts 6 and 7,~~where the 30- day interval will continue between Temferon dosing of a patient and commencement of conditioning for the next patient.</td></tr>
</table>

Location	Rationale	New (Version 5.0 of 21st October 2022)
		The DMC will review the safety and efficacy data for Cohort 7 ***and subsequently for Cohort 8. Following their review and recommendation, an additional 3 patients will receive Temferon at a dose selected from either Cohort 7 or 8 in order to provide additional safety data before progression to Part B of the study.*** ~~and make a recommendation whether to proceed to Part B~~. ***No staggering is foreseen in the additional 3 patients to be dosed.***
Section 5.11.1 page 113	Emphasise importance of recording CBC, microbiology and concomitant medications from ASCT admission in the CRF	• ***Daily complete blood count (and results recorded in the CRF).*** Erythrocyte and platelet concentrates will be transfused according to clinical need; the investigator should consider infusion of platelet concentrates should blood platelet concentration be less than 50 x 103/mm3. All blood products will be leukocyte depleted and irradiated. All efforts will be made to provide HLA-matched platelets in immunized patients with documented anti-HLA antibodies.<br><br>• CMV (PCR), EBV (PCR) viral load and aspergillus antigenemia will be determined weekly till Day +90. ***These data should be recorded in the eCRF.***<br><br>• ***All medications (including for prophylaxis) and supportive therapies administered during the hospitalization should be recorded in the eCRF.***
Section 6.1, page 115<br><br>Section 6.2.5, page 125<br><br>Section 6.2.5.8, page 127	Update cross-reference to Tables	***Table 12-1, Table 12-2, Table 12-3, Table 12-4 and Table 12-5***
Section 6.2.4.3, page, page 124	Clarification on the IFN induced genes to be evaluated	***An IFN-induced gene signature comprising OAS1, IRF7 and IFIT1 will be measured on PB in CD14+ and CD15+ cells by quantitative RT-PCR at the Screening visit (negative control) and at Days +30, +90, +180, +360 and +720 post Temferon infusion.***

Location	Rationale	New (Version 5.0 of 21st October 2022)
Section 6.3.3. 2. page 132	Clarity on attributing relationship of non-IMP factors to AEs	***In addition, the potential relationship of the AE to the harvesting of autologous HSPCs, conditioning chemotherapy, or other concomitant medication should be assessed by the Investigator with details provided in the CRF.***
Section 7.4.2, page 137	Inclusion of busulfan monotherapy for further analysis	The proportion of patients will be summarized by cohort and Temferon dose, achieving hematologic recovery by Day +30 following BCNU and thiotepa, ~~or~~ thiotepa and busulfan, ***or busulfan monotherapy*** administration from administration of non-manipulated autologous HSPC and Temferon.
Section 7.4.3, page 138	Inclusion of an interim analysis at end of Part A	~~No interim analyses are planned~~ ***An interim analysis will be performed once the final patient recruited in Part A of the study completes the D+30 visit.***
Table 12-2, page 167-169	SoE for Cohorts 7 and 8 from Screening to D+30	See relevant page for detail
