# Supplementary material for: Tumor-targeted interferon-α gene therapy for glioblastoma: a phase 1 trial
Source: Nat Med. 2026 Jun 1;32(6):2216–26. doi: 10.1038/s41591-026-04419-1 (PMC13768172; doi:10.1038/s41591-026-04419-1)
Supplement: Supplementary file 4 [file 41591_2026_4419_MOESM9_ESM.pdf]

5′ tggaagggctaattcactcccaacgaagacaagatctgcttttttgcttgtactgg 55
3′ accttcccgattaagtgagggttgcttctgttctagacgaaaaaacgaacatgacc

SIN 5' LTR (ΔU3)

gtctctctggttagaccagatctgagcctgggagctctctggctaactagggaac 110
cagagagaccaatctggtctagactcggaccctcgagagaccgattgatcccttg

SIN 5' LTR (ΔU3)

ccactgcttaagcctcaataaagcttgccttgagtgcttcaagtagtgtgtgccc 165
ggtgacgaattcggagttatttcgaacggaactcacgaagttcatcacacacggg

SIN 5' LTR (ΔU3)

gtctgttgtgtgactctggtaactagagatccctcagacccttttagtcagtgtg 220
cagacaacacactgagaccattgatctctagggagtctgggaaaatcagtcacac

SIN 5' LTR (ΔU3)

gaaaatctctagcagtggcgcccgaacagggacctgaaagcgaaagggaaaccag 275
cttttagagatcgtcaccgcgggcttgtccctggactttcgctttccctttggtc

SIN 5' LTR (ΔU3)

agctctctcgacgcaggactcggcttgctgaagcgcgcacggcaagaggcgaggg 330
tcgagagagctgcgtcctgagccgaacgacttcgcgcgtgccgttctccgctccc

HIV-1 Ψ

gcggcgactggtgagtacgccaaaaattttgactagcggaggctagaaggagaga 385
cgccgctgaccactcatgcggtttttaaaactgatcgcctccgatcttcctctct

HIV-1 Ψ

gatgggtgcgagagcgtcagtattaagcgggggagaattagatcgcgatgggaaa 440
ctacccacgctctcgcagtcataattcgccccctcttaatctagcgctaccttt

HIV-1 Ψ

aaattcggttaaggccagggggaaagaaaaaatataaattaaaacatatagtatg 495
tttaagccaattccggtccccctttcttttttatatttaattttgtatatcatac

ggcaagcagggagctagaacgattcgcagttaatcctggcctgttagaaacatca 550
ccgttcgtccctcgatcttgctaagcgtcaattaggaccggacaatctttgtagt

```
gaaggctgtagacaaatactgggacagctacaaccatcccttcagacaggatcag
                                                          605
cttccgacatctgtttatgaccctgtcgatgttggtagggaagtctgtcctagtc

aagaacttagatcattatataatacagtagcaaccctctattgtgtgcatcaaag
                                                          660
ttcttgaatctagtaatatattatgtcatcgttgggagataacacacgtagtttc

gatagagataaaagacaccaaggaagctttagacaagatagaggaagagcaaaac
                                                          715
ctatctctattttctgtggttccttcgaaatctgttctatctccttctcgttttg

aaaagtaagaccaccgcacagcaagcggccgctgatcttcagacctggaggagga
                                                          770
ttttcattctggtggcgtgtcgttcgccggcgactagaagtctggacctcctcct

gatatgagggaccaattgtggccagacgattccttaactcgtgttacacctgcag
                                                          825
ctatactccctggttaacaccggtctgctaaggaattgagcacaatgtggacgtc
                        TIE2 enhancer

aatgagttttagatctagctgtgacctcttccccagccccaccccattgtccc
                                                          880
ttactcaaaatctagatcgacactggagaaggggtcggggtggggtaacaggg
TIE2 enhancer

cttgtgtgccttcaggaatctgatcattcttctcctgctccttcccaaaggct
                                                          935
gaacacacggaagtccttagactagtaagaagagaggacgaggaagggtttccga
TIE2 enhancer

gcaggagcaggtgtgaagacgtggatgtgccagatgcagagtcctgacacttttc
                                                          990
cgtcctcgtccacacttctgcacctacacggtctacgtctcaggactgtgaaaag
TIE2 enhancer

aacacatctgcatattagaggaagtacataccattgcttggtggtttcatgtct
                                                          1045
ttgtgtagacgtataatctccttcatgtatgggtaacgaaccaccaaagtacaga
TIE2 enhancer

aatgtggtatgagtgtgacaaagagagggagaaaatttggactagccaaagaagc
                                                          1100
ttacaccatactcacactgtttctctccctctttaaacctgatcggtttcttcg
TIE2 enhancer

cagtcaggcgtggggtttgaagggcatcgtgggcggctgtcatttgctctctgct
                                                          1155
gtcagtccgcaccccaaacttcccgtagcacccgccgacagtaaacgagagacga
TIE2 enhancer
```

```
tgtcacagccccttgcccagggcttgaccagtgaggtgtatgtgctggtcacacc  1210
acagtgtcggggaacgggtcccgaactggtcactccacatacacgaccagtgtgg
TIE2 enhancer

catctcagcagatctgtcagctttcccgcttttgttaaagggtgatatcatgctt  1265
gtagagtcgtctagacagtcgaaagggcgaaaacaatttcccactatagtacgaa
TIE2 enhancer

cctgggggagcactggaagacaatgctcggccactttcctccagatacaatagg  1320
ggacccccctcgtgaccttctgttacgagccggtgaaaggaggtctatgttatcc
TIE2 enhancer

cggagtcaggaaggcagtattgacattgctggggctggggaggcactcactgctc  1375
gcctcagtccttccgtcataactgtaacgaccccgacccctccgtgagtgacgag
TIE2 enhancer

tgcggccgtcagatggtgaaccagcttaaccttggcacacagggcctgggttgtg  1430
acgccggcagtctaccacttggtcgaattggaaccgtgtgtcccggacccaacac
TIE2 enhancer

caaggcgtctggctgcagagccaaaggggactccaccctggggacaggagtgctt  1485
gttccgcagaccgacgtctcggtttcccctgaggtgggaccccctgtcctcacgaa
TIE2 enhancer

tagacatctgggaatctgggatgggcttcaaattctgatccctgtgtcagaaaca  1540
atctgtagacccttagaccctacccgaagtttaagactagggacacagtctttgt
TIE2 enhancer

accacaaaacaataagagtaccagtaataacaaaaatgactaccctaggttgaat  1595
tggtgttttgttattctcatggtcattattgtttttactgatgggatccaactta
TIE2 enhancer

gcctttatgtgccaagtgtcaattggagaagtgaattatataaatataaagtagt  1650
cggaaatacacggttcacagttaacctcttcacttaatatatttatatttcatca
TIE2 enhancer

aaaaattgaaccattaggagtagcacccaccaaggcaaagagaagagtggtgcag  1705
tttttaacttggtaatcctcatcgtgggtggttccgtttctcttctcaccacgtc
```

```
agagaaaaagagcagtgggaataggagctttgttccttgggttcttgggagcag   1760
tctctttttctcgtcacccttatcctcgaaacaaggaacccaagaaccctcgtc
                        [RRE]

caggaagcactatgggcgcagcctcaatgacgctgacggtacaggccagacaatt   1815
gtccttcgtgatacccgcgtcggagttactgcgactgccatgtccggtctgttaa
[RRE]

attgtctggtatagtgcagcagcagaacaatttgctgagggctattgaggcgcaa   1870
taacagaccatatcacgtcgtcgtcttgttaaacgactcccgataactccgcgtt
[RRE]

cagcatctgttgcaactcacagtctggggcatcaagcagctccaggcaagaatcc   1925
gtcgtagacaacgttgagtgtcagaccccgtagttcgtcgaggtccgttcttagg
[RRE]

tggctgtggaaagatacctaaaggatcaacagctcctggggatttggggttgctc   1980
accgacacctttctatggatttcctagttgtcgaggacccctaaaccccaacgag
[RRE]

tggaaaactcatttgcaccactgctgtgccttggaatgctagttggagtaataaa   2035
accttttgagtaaacgtggtgacgacacggaaccttacgatcaacctcattattt

tctctggaacagatttggaatcacacgacctggatggagtgggacagagaaatta   2090
agagaccttgtctaaaccttagtgtgctggacctacctcaccctgtctctttaat

acaattacacaagcttaatacactccttaattgaagaatcgcaaaaccagcaaga   2145
tgttaatgtgttcgaattatgtgaggaattaacttcttagcgttttggtcgttct

aaagaatgaacaagaattattggaattagataaatgggcaagtttgtggaattgg   2200
tttcttacttgttcttaataaccttaatctatttacccgttcaaacaccttaacc

tttaacataacaaattggctgtggtatataaaattattcataatgatagtaggag   2255
aaattgtattgtttaaccgacaccatatattttaataagtattactatcatcctc

gcttggtaggtttaagaatagtttttgctgtactttctatagtgaatagagttag   2310
cgaaccatccaaattcttatcaaaaacgacatgaaagatatcacttatctcaatc

gcagggatattcaccattatcgtttcagacccacctcccaaccccgaggggaccc   2365
cgtccctataagtggtaatagcaaagtctgggtggagggttggggctcccctggg
```

gacaggcccgaaggaatagaagaagaaggtggagagagagacagagacagatcca
ctgtccgggcttccttatcttcttcttccacctctctctctgtctctgtctaggt
2420

ttcgattagtgaacggatctcgacggtatcggttaacttttaaaagaaaaggggg
aagctaatcacttgcctagagctgccatagccaattgaaaattttcttttccccc
2475
cPPT/CTS

gattggggggtacagtgcaggggaaagaatagtagacataatagcaacagacata
ctaacccccccatgtcacgtcccctttcttatcatctgtattatcgttgtctgtat
2530
cPPT/CTS

caaactaaagaattacaaaaacaaattacaaaaattcaaaattttatcgatcacg
gtttgatttcttaatgtttttgtttaatgtttttaagttttaaaatagctagtgc
2585
cPPT/CTS

agactagcctcgagtcacacctgcaaactggaaacattaattggttcttaagatc
tctgatcggagctcagtgtggacgtttgacctttgtaattaaccaagaattctag
2640
TIE2 promoter

atcatcgacgtgataaaacctgggacagaaattagtcaagactagctgcatctgc
tagtagctgcactattttggaccctgtctttaatcagttctgatcgacgtagacg
2695
TIE2 promoter

cttttcctctggtgggtaggaaaaggaggagtataatgatttcctcaggcatgaa
gaaaaggagaccacccatccttttcctcctcatattactaaaggagtccgtactt
2750
TIE2 promoter

ggtcgatgatgagcaaagtgtatactctctaatctaatgtcataattcatattgt
ccagctactactcgtttcacatatgagagattagattacagtattaagtataaca
2805
TIE2 promoter

ggagtaattatctggataagtgtagggtctctgacctcattctagatattgtaca
cctcattaatagacctattcacatcccagagactggagtaagatctataacatgt
2860
TIE2 promoter

ttccatggctattttcattttggtccatgaactctctttgctctcatgagcacca
aaggtaccgataaaagtaaaaccaggtacttgagagaaacgagagtactcgtggt
2915
TIE2 promoter

ttttatcccaatctaatcctgtatgtttgtgttttacacagattagttttaa 2970
aaaaatagggttagattaggacatacaaacacaaaatgtgtctaatcaaaatt
TIE2 promoter

atgttatatataatttgcttctgaaacaccattgctcaatgactaccaaatcttt 3025
tacaatatatattaaacgaagactttgtggtaacgagttactgatggtttagaaa
TIE2 promoter

ctcattaccaaaatccttctatgccaacttcttcaagaaatttgatcacctttag 3080
gagtaatggttttaggaagatacggttgaagaagttctttaaactagtggaaatc
TIE2 promoter

atgaattgttaatgaaaattaaagctatagccggcaacatgggtatctttgggct 3135
tacttaacaattactttaatttcgatatcggccgttgtacccatagaaacccga
TIE2 promoter

aatggccaaccaacaggccatctgtgtgaaagaaaacaggctaacaattttggac 3190
ttaccggttggttgtccggtagacacactttcttttgtccgattgttaaaacctg
TIE2 promoter

tctggtctcttggggctacattgagcattgacctcaccggtgctcactgaaatta 3245
agaccagagaaccccgatgtaactcgtaactggagtggccacgagtgactttaat
TIE2 promoter

attgcttttcaggttgtatttctcatcacggaaaccttcttctcccaattcaaa 3300
taacgaaaagtccaacataaaagagtagtgcctttggaagaagagggttaagttt
TIE2 promoter

ccatgtgggttaaaatgagaaaacaaaagccaaaacggcttcccacacccaaaag 3355
ggtacacccaattttactcttttgttttcggttttgccgaagggtgtgggttttc
TIE2 promoter

ctccttctgtcagagatcccagtagccccgggagagctgttagaagtctgagaag 3410
gaggaagacagtctctagggtcatcggggccctctcgacaatcttcagactcttc
TIE2 promoter

gattggtcatcatcgcataccatacataggtggagggcttgttattctcagtttc 3465
ctaaccagtagtagcgtatggtatgtatccacctcccgaacaataagagtcaaag
TIE2 promoter

```
ccgcctatgagaggatacccctattgtttctgaaaatgctgaccgggacccacac   3520
ggcggatactctcctatggggataacaaagactttacgactggccctgggtgtg
[TIE2 promoter]

ttccaacaaaaattcctctgcccctacagcagcagcaaaagcagcagcagaagca   3575
aaggttgtttttaaggagacggggatgtcgtcgtcgttttcgtcgtcgtcttcgt
[TIE2 promoter]

acagcaacagataagtgttttgatgaattgcgagatggatagggcttgagtgccc   3630
tgtcgttgtctattcacaaaactacttaacgctctacctatcccgaactcacggg
[TIE2 promoter]

ccagccctgctgataccaaatgcctttaagatacagcctttcccatcctaatcta   3685
ggtcgggacgactatggtttacggaaattctatgtcggaaagggtaggattagat
[TIE2 promoter]

caaaggaaacaggaaaaaggaacttaaaactccctgtgctcagacagaaatgaga   3740
gtttcctttgtcctttttccttgaattttgagggacacgagtctgtctttactct
[TIE2 promoter]

ctgttacagcctgcttctgtgctgttccttcttgcctctaacttgtaaacaagac   3795
gacaatgtcggacgaagacacgacaaggaagaacggagattgaacatttgttctg
[TIE2 promoter]

gtagtaggacgatgctaatggaaagtcacaaaccgctgggtttttgaaaggatcc   3850
catcatcctgctacgattacctttcagtgtttggcgacccaaaaactttcctagg
[TIE2 promoter]

tagactcgagcggccgccactgtgctggatatctgcagaattgcccttatggcct   3905
atctgagctcgccggcggtgacacgacctatagacgtcttaacgggaataccgga
1
Met Ala
[IFN-α2b >]

tgacctttgctttactggtggccctcctggtgctcagctgcaagtcaagctgctc   3960
actggaaacgaaatgaccaccgggaggaccacgagtcgacgttcagttcgacgag
5 10 15 20
Leu Thr Phe Ala Leu Leu Val Ala Leu Leu Val Leu Ser Cys Lys Ser Ser Cys Ser
[IFN-α2b >]
```

tgtgggctgtgatctgcctcaaacccacagcctgggtagcaggaggaccttgatg 4015
acacccgacactagacggagtttgggtgtcggacccatcgtcctcctggaactac

25 30 35
Val Gly Cys Asp Leu Pro Gln Thr His Ser Leu Gly Ser Arg Arg Thr Leu Met
IFN-α2b

ctcctggcacagatgaggagaatctctcttttctcctgcttgaaggacagacatg 4070
gaggaccgtgtctactcctcttagagagaaaagaggacgaacttcctgtctgtac

40 45 50 55
Leu Leu Ala Gln Met Arg Arg Ile Ser Leu Phe Ser Cys Leu Lys Asp Arg His
IFN-α2b

actttggatttccccaggaggagtttggcaaccagttccaaaaggctgaaaccat 4125
tgaaacctaaaggggtcctcctcaaaccgttggtcaaggttttccgacttttggta

60 65 70 75
Asp Phe Gly Phe Pro Gln Glu Glu Phe Gly Asn Gln Phe Gln Lys Ala Glu Thr Ile
IFN-α2b

ccctgtcctccatgagatgatccagcagatcttcaatctcttcagcacaaaggac 4180
gggacaggaggtactctactaggtcgtctagaagttagagaagtcgtgtttcctg

80 85 90
Pro Val Leu His Glu Met Ile Gln Gln Ile Phe Asn Leu Phe Ser Thr Lys Asp
IFN-α2b

tcatctgctgcttgggatgagaccctcctagacaaattctacactgaactctacc 4235
agtagacgacgaaccctactctgggaggatctgtttaagatgtgacttgagatgg

95 100 105 110
Ser Ser Ala Ala Trp Asp Glu Thr Leu Leu Asp Lys Phe Tyr Thr Glu Leu Tyr
IFN-α2b

agcagctgaatgacctggaagcctgtgtgatacagggggtgggggtgacagagac 4290
tcgtcgacttactggaccttcggacacactatgtcccccacccccactgtctctg

115 120 125 130
Gln Gln Leu Asn Asp Leu Glu Ala Cys Val Ile Gln Gly Val Gly Val Thr Glu Thr
IFN-α2b

tcccctgatgaaggaggactccattctggctgtgaggaaatacttccaaagaatc 4345
aggggactacttcctcctgaggtaagaccgacactcctttatgaaggtttcttag

135 140 145
Pro Leu Met Lys Glu Asp Ser Ile Leu Ala Val Arg Lys Tyr Phe Gln Arg Ile
IFN-α2b

actctctatctgaaagagaagaaatacagcccttgtgcctgggaggttgtcagag 4400
tgagagatagactttctcttctttatgtcgggaacacggaccctccaacagtctc

150 155 160 165
Thr Leu Tyr Leu Lys Glu Lys Lys Tyr Ser Pro Cys Ala Trp Glu Val Val Arg
IFN-α2b

```
cagaaatcatgagatcttttctttgtcaacaaacttgcaagaaagtttaagaag    4455
gtctttagtactctagaaaagaaacagttgtttgaacgttctttcaaattcttc
          170                 175                 180                 185
Ala Glu Ile Met Arg Ser Phe Ser Leu Ser Thr Asn Leu Gln Glu Ser Leu Arg Ser
IFN-α2b

taaggaatgaaagggcaattccaccacactggactagtggatccgtcgacaatca    4510
attccttactttcccgttaaggtggtgtgacctgatcacctaggcagctgttagt
Lys Glu *
IFN-α2b
mWPRE

acctctggattacaaaatttgtgaaagattgactggtattcttaactatgttgct    4565
tggagacctaatgttttaaacactttctaactgaccataagaattgatacaacga
mWPRE

cctttacgctatgtggatacgctgctttaatgcctttgtatcatgctattgctt    4620
ggaaaatgcgatacacctatgcgacgaaattacggaaacatagtacgataacgaa
mWPRE

cccgtatggctttcattttctcctccttgtataaatcctggttgctgtctcttta    4675
gggcataccgaaagtaaaagaggaggaacatatttaggaccaacgacagagaaat
mWPRE

tgaggagttgtggcccgttgtcaggcaacgtggcgtggtgtgcactgtgtttgct    4730
actcctcaacaccgggcaacagtccgttgcaccgcaccacacgtgacacaaacga
mWPRE

gacgcaacccccactggttggggcattgccaccacctgtcagctcctttccggga    4785
ctgcgttgggggtgaccaaccccgtaacggtggtggacagtcgaggaaaggccct
mWPRE

ctttcgctttccccctccctattgccacggcggaactcatcgccgcctgccttgc    4840
gaaagcgaaagggggagggataacggtgccgccttgagtagcggcggacggaacg
mWPRE

ccgctgctggacaggggctcggctgttgggcactgacaattccgtggtgttgtcg    4895
ggcgacgacctgtccccgagccgacaacccgtgactgttaaggcaccacaacagc
mWPRE
```

```
gggaaatcatcgtcctttccttggctgctcgcctgtgttgccacctggattctgc        4950
ccctttagtagcaggaaaggaaccgacgagcggacacaacggtggacctaagacg
[                         mWPRE                        ]

gcgggacgtccttctgctacgtcccttcggccctcaatccagcggaccttccttc        5005
cgccctgcaggaagacgatgcagggaagccgggagttaggtcgcctggaaggaag
[                         mWPRE                        ]

ccgcggcctgctgccggctcgagcattattactcacggtacgacgtagcattatt        5060
ggcgccggacgacggccgagctcgtaataatgagtgccatgctgcatcgtaataa
[mWPRE]                [  miR-126-3p recognition elements  ]

actcacggtacgaggtacctttaagaccaatgacttacaaggcagctgtagatct        5115
tgagtgccatgctccatggaaattctggttactgaatgttccgtcgacatctaga
[           ]
miR-126-3p recognition elements

tagccacttttttaaaagaaaaggggggactggaagggctaattcactcccaacga        5170
atcggtgaaaaattttcttttcccccctgaccttcccgattaagtgagggttgct
                                   [    3' LTR (ΔU3)    ]

agacaagatctgcttttgcttgtactgggtctctctggttagacc   3'
                                                   5216
tctgttctagacgaaaaacgaacatgacccagagagaccaatctgg (P)  5'
[              3' LTR (ΔU3)                  ]
```
